# Acridine–Isoxazole and Acridine–Azirine Hybrids: Synthesis, Photochemical Transformations in the UV/Visible Radiation Boundary Region, and Anticancer Activity

**DOI:** 10.3390/molecules29071538

**Published:** 2024-03-29

**Authors:** Ekaterina E. Galenko, Mikhail S. Novikov, Alexander S. Bunev, Alexander F. Khlebnikov

**Affiliations:** 1 Institute of Chemistry, St. Petersburg State University, 7/9 Universitetskaya Naberezhnaya, St. Petersburg 199034, Russia; ekaterina.e.galenko@gmail.com (E.E.G.); m.novikov@spbu.ru (M.S.N.); 2 Medicinal Chemistry Center, Togliatti State University, Togliatti 445020, Russia; a.s.bunev@gmail.com

**Keywords:** acridines, isoxazoles, azirines, heterocyclic hybrids, photochemical reactions, cytotoxicity

## Abstract

Easy-to-handle *N*-hydroxyacridinecarbimidoyl chloride hydrochlorides were synthesized as convenient nitrile oxide precursors in the preparation of 3-(acridin-9/2-yl)isoxazole derivatives via 1,3-dipolar cycloaddition with terminal alkynes, 1,1-dichloroethene, and acrylonitrile. Azirines with an acridin-9/2-yl substituent attached directly or via the 1,2,3-triazole linker to the azirine C2 were also synthesized. The three-membered rings of the acridine–azirine hybrids were found to be resistant to irradiation in the UV/visible boundary region, despite their long-wave absorption at 320–420 nm, indicating that the acridine moiety cannot be used as an antenna to transfer light energy to generate nitrile ylides from azirines for photoclick cycloaddition. The acridine–isoxazole hybrids linked at the C9–C3 or C2–C3 atoms under blue light irradiation underwent the addition of such hydrogen donor solvents, such as, toluene, *o*-xylene, mesitylene, 4-chlorotoluene, THF, 1,4-dioxane, or methyl *tert*-butyl ether (MTBE), to the acridine system to give the corresponding 9-substituted acridanes in good yields. The synthesized acridine–azirine, acridine–isoxazole, and acridane–isoxazole hybrids exhibited cytotoxicity toward both all tested cancer cell lines (HCT 116, MCF7, and A704) and normal cells (WI-26 VA4).

## 1. Introduction

Acridine derivatives exhibit a number of biological activities, including anticancer, antimicrobial, antibiotic, antiacetylcholinesterase, antileukemic, antiprotozoal, neuroleptic, anti-dementia, telomerase inhibitory, and many others [1,2,3,4,5]. A stimulating strategy in drug discovery to maximize efficacy, minimize side effects, and combat drug resistance is the synthesis of hybrid molecules that include a combination of two biologically relevant heterocyclic moieties that act on different targets [6]. In particular, a number of molecular hybrids [7,8,9,10,11,12,13,14,15] containing an acridine moiety have been prepared because of efforts to develop new therapeutic agents. Thus, tacrine–acridine [8], cyclopentaquinoline–acridine [9], and acridine–flavone hybrids [10] are potentially useful for treating Alzheimer’s disease, quinoline–acridine and pyrrolidine–acridine–artemisinin hybrids are antimalarials [11,12,13], neocryptolepine–acridine hybrids exhibit antiproliferative activity [6,14], and thiophene–acridine hybrids demonstrate antitumor activity [3,15]. Another very important class of N-heterocyclic compound is isoxazoles because they have a wide spectrum of biological activity used as base for developing of several commercially available drugs [16,17,18,19,20,21,22,23,24,25,26]. Some biologically active hybrids of isoxazoles with various heterocycles have been synthesized and studied [20,21,22,23,24,25,26,27]. Meanwhile, not many acridines containing an isoxazole moiety have been obtained so far. Thus, 9-(4-isoxazolylanilino)acridines with antibacterial and larvicidal activity were synthesized from corresponding chalcones [28]. In an attempt to at least partially fill the gap in the synthesis of acridine–isoxazole hybrids, we report a simple approach to the synthesis of various isoxazole–acridine and azirine–acridine hybrids, in which these heterocycles are directly linked, as well as some of their photochemical reactions under irradiation in the UV/visible boundary region, allowing the easy synthesis of acridan–isoxazole hybrids. The anticancer activity of a number of synthesized acridine/acridan-substituted isoxazoles and acridine-substituted azirines is also reported.

## 2. Results and Discussion

**Scheme 1:**
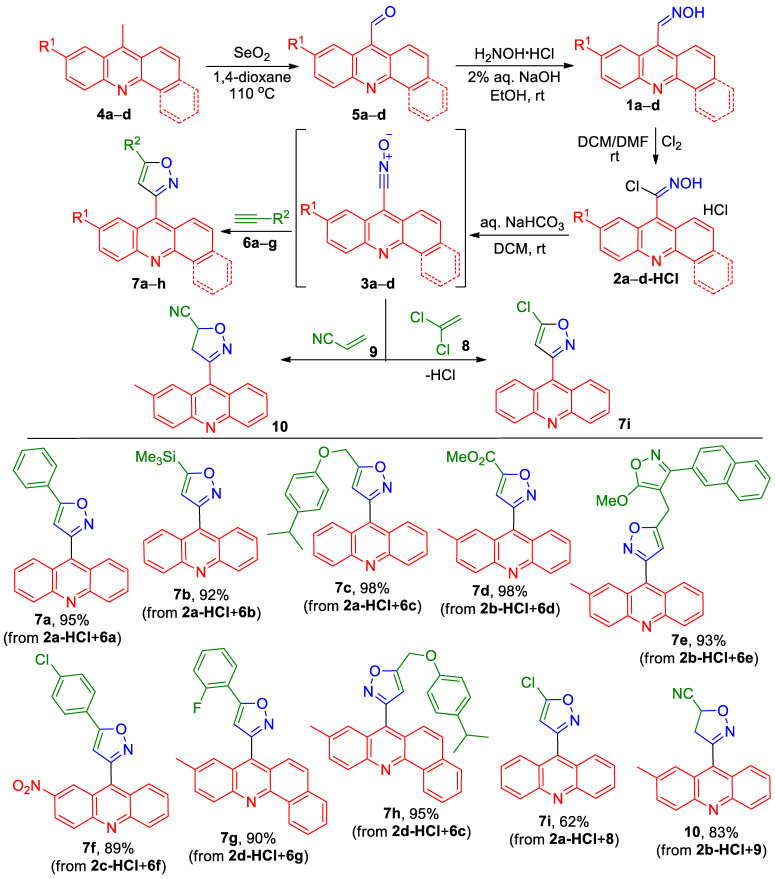
Synthesis of acridinyl-substituted isoxazole derivatives **7a**–**i**, **10**.

The only known (acridin-9-yl)isoxazole, ethyl 3-(acridin-9-yl)-5-methylisoxazole-4-carboxylate, was prepared from acridine-9-carbaldehyde oxime **1a** by converting it into unstable *N*-hydroxyacridine-9-carbimidoyl chloride **2a** under the action of *N*-chlorosuccinimide, followed by immediate treatment with triethylamine and ethyl 3-(pyrrolidin-1-yl)but-2-enoate [29]. We were unsuccessful in our attempts to apply the procedure proposed in this work for generating acridinyl-substituted nitrile oxide to obtain derivatives of 9-(isoxazolyl)acridines through cycloaddition to alkenes and acetylenes. In all experiments, the only compound isolated was the hydrolysis product of *N*-hydroxyacridine-9-carbimidoyl chloride **2a** to acridine-9-carboxylic acid. Since the synthesis of *N*-hydroxyacridine-9-carbimidoyl chloride **2a** and its application described in the work [29] have never been applied by other authors, unlike the anthracene analogue [30], we decided to find another, more reliable approach. It was found that the use of easy-to-handle *N*-hydroxyacridine-9-carbimidoyl chloride hydrochlorides **2-HCl**, instead of the unstable and moisture-sensitive *N*-hydroxyacridine-9-carbimidoyl chloride **2a**, allowed for the cycloaddition of acridinyl-substituted nitrile oxides **3** to acetylenes and alkenes to be carried out at a high yield (Scheme 1). These compounds were easily prepared in good to excellent yields by the oxidation of 9-methylacridines **4a**–**c** or 7-methylbenzo[*c*]acridine **4d** with SeO_2_, the condensation of aldehydes **5a**–**d** with hydroxylamine to give oximes **1a**–**d**, followed by chlorination with Cl_2_. The reaction of nitrile oxides **3a**–**d**, generated from compounds **2a**–**d-HCl** in a two-phase system DCM/sat. aq. NaHCO_3_, with alkynes **6a**–**g** gave 3-(acridin-9-yl)isoxazoles **7a**–**h** in 90–98% yields. The cycloaddition of nitrile oxide **3a**, generated from compounds **2a-HCl**, to 1,1-dichloroethene **8** accompanied the dehydrochlorination of intermediate 5,5-dichloro-4,5-dihydroisoxazoles and gave acridinyl-substituted 5-chloroizoxazole **7i** in a 62% yield (Scheme 1). The cycloaddition of nitrile oxide **3b** to acrylonitrile **9** afforded 3-(2-methylacridin-9-yl)-4,5-dihydroisoxazole-5-carbonitrile **10** in an 83% yield (Scheme 1).

**Scheme 2:**
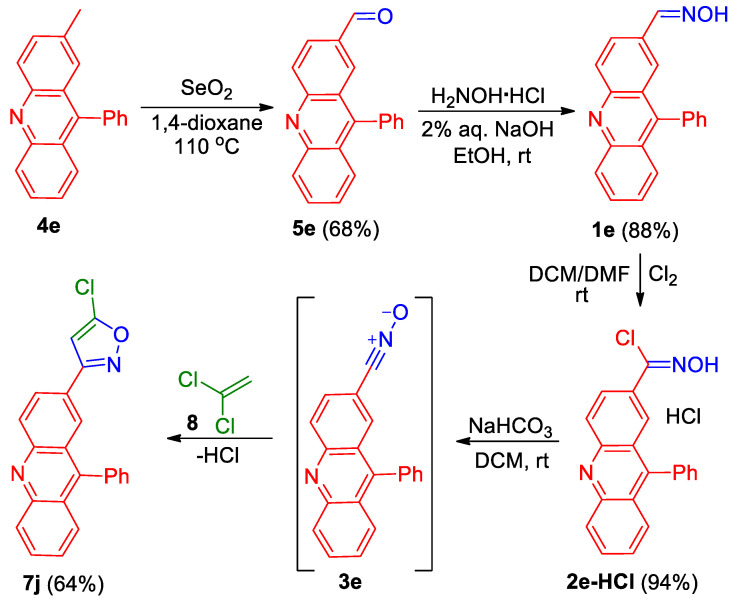
Synthesis of 3-(acridin-2-yl)isoxazole **7j**.

Analogously, by the cycloaddition of nitrile oxide **3e**, generated from compound **2e-HCl**, to 1,1-dichloroethene **8,** 3-(acridin-2-yl)isoxazole **7j** was prepared in a 64% yield (Scheme 2).

**Scheme 3:**
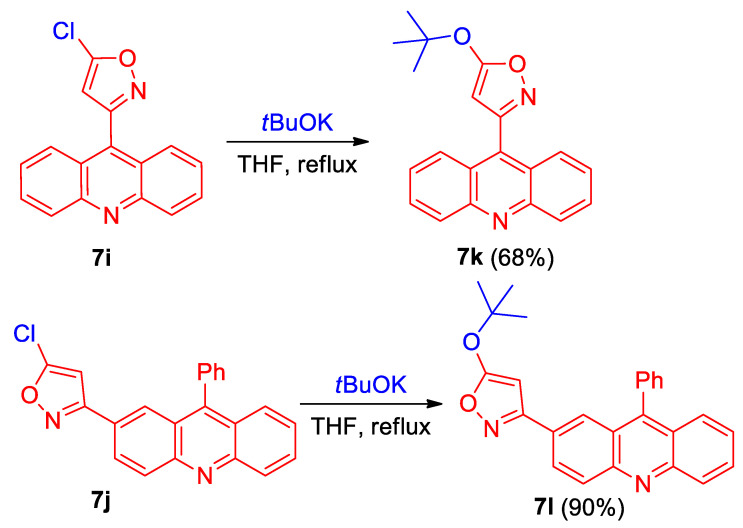
Synthesis of acridinyl-substituted isoxazoles **7k** and **l**.

Acridinyl-substituted 5-chloroisoxazoles **7i**,**j** can be used for the preparation of other derivatives by the substitution of chlorine with O-nucleophiles (Scheme 3). Thus, the reaction of 5-chloroisoxazoles **7i**,**j** with *t*BuOK gives 5-(*tert*-butoxy)isoxazoles **7k**,**l** in a 68% and 90% yield, respectively (Scheme 3).

Isoxazoles with a heteroatom substituent at C5 have found wide application as convenient precursors of 2*H*-azirine-2-carboxylic acid derivatives, versatile building blocks for organic synthesis [31]. A very large number of structurally diverse azirines have been synthesized to date, including those which are of interest for biorthogonal chemistry [32], the chemistry of natural compounds [33], and medicine [34], but azirines with acridinyl substituents are still unknown in the literature. 

**Scheme 4:**
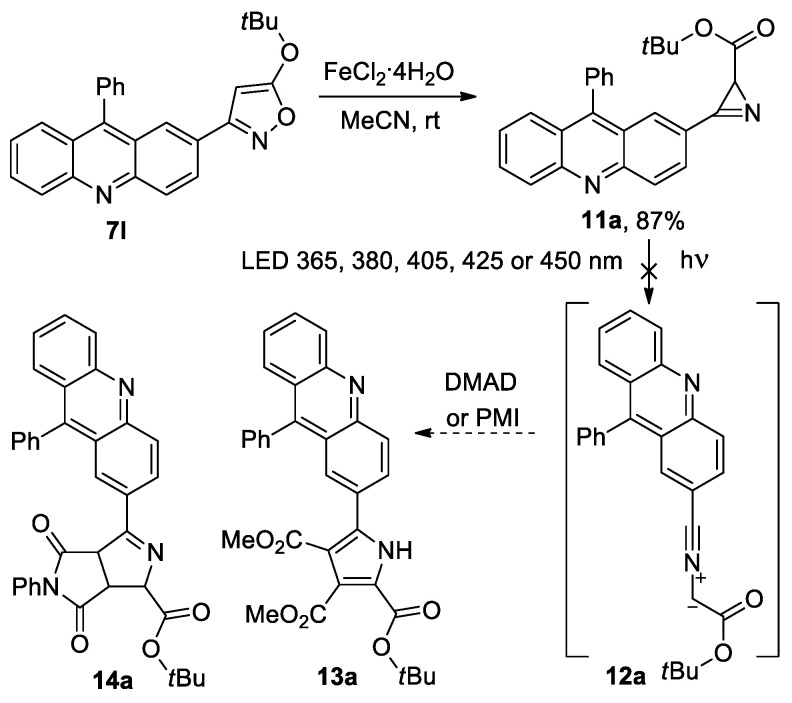
Synthesis of acridinyl-substituted azirine **11a**.

Having acridinyl-substituted isoxazoles at our disposal, we decided to prepare acridinyl-substituted azirines in order to evaluate the possibility of their use in photoclick cycloaddition [35,36,37,38]. The authors of the work [35] synthesized 3-(pyren-1-yl)-2*H*-azirine, which absorbs light in the 400 nm region, allowing for the activation of an efficient cycloaddition reaction for azirine-based ligation, which is potentially suitable for the bioorthogonal conjugation of polymers and peptides using low-energy visible light sources. According to them, such azirine allows for avoiding handling problems during synthesis or sample preparation, and the pyrene group is not only critical for light absorption, but is also incorporated into the carbon backbone of the cycloadducts, thereby providing additional desirable functions such as an integrated fluorescent marker or an anchor for π-π stacking. We assumed that the involvement of acridine-substituted azirines in photoinitiated cycloaddition reactions, using low-energy visible light sources, cannot be ruled out, since the acridinyl substituent provides light absorbance in the UV/visible radiation boundary region (385–405 nm) [39,40]. Moreover, acridines bind to DNA and RNA by intercalation, and their fluorescence could also be of interest in the case of the implementation of the discussed cycloaddition reaction. Azirine **11a** was obtained by the isoxazole–azirine isomerization [31] of isoxazole **7l** catalyzed by FeCl_2_·4H_2_O at an 87% yield (Scheme 4).

**Scheme 5:**
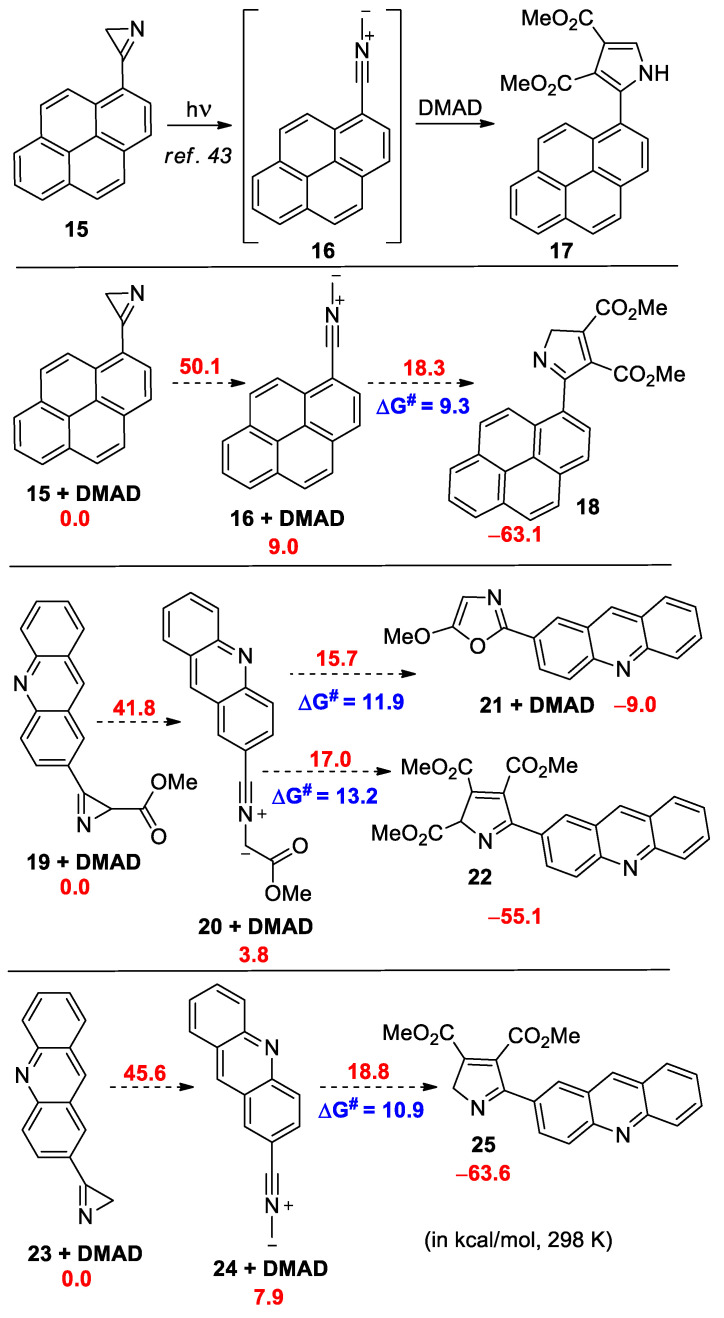
Relative Gibbs free energies for the transformations of pyrenyl- and acridinyl-substituted nitrile ylides (in kcal/mol, 298 K, DFT B3LYP-D3/6-311+G(d,p) level with SMD model for MeCN).

In the UV spectrum of azirine **11a**, there is a long-wave absorption band in the region of 320–420 nm (see Figure S3 in the Supporting Information), therefore, its photolysis in the presence of DMAD or *N*-phenylmaleimide (PMI) (20 equiv.) was carried out in acetonitrile using LED 365, 380, 405, 425, or 450 nm, as well with white light LED (380−760 nm). However, under all the conditions tested, nitrile ylide **12a**, which was expected to be formed by photolysis [35,41,42], did not produce a cycloaddition product **13a** with DMAD or **14a** with PMI, and the starting azirine was isolated unchanged. The same result was also obtained during the photolysis of isoxazole **7l** in the presence of DMAD or PMI, although it is known that azirines and nitrile ylides can successively be formed during the photolysis of isoxazoles [42]. Meanwhile, the photolysis of 3-(pyren-1-yl)-2*H*-azirine **15**, which has a long-wave absorption band in the region of 360–400 nm, using LED (410–420 nm) in acetonitrile in the presence of DMAD gave, via nitrile ylide **16**, cycloaddition product **17** with a yield of 75% [35] (Scheme 5). In an attempt to find the reason for this difference in the reactivity of azirines **15** and **11a**, we performed DFT calculations (the B3LYP-D3/6-311+G(d,p) level of theory with SMD model for MeCN, see Supporting Information for details) of the cycloaddition of the nitrile ylides formed from azirine **15** and model azirines **19** and **23** to DMAD (Scheme 5). 

From the calculation results, it follows that, although the barrier for the cycloaddition of nitrile ylide **20** to DMAD is higher than the barrier for the cycloaddition of nitrile ylide **16**, it is still low enough to be easily overcome at room temperature (ΔG^#^ 13.2 vs. 9.3 kcal/mol). At the same time, nitrile ylide **24**, without a methoxycarbonyl substituent, has a very similar barrier to its cycloaddition to DMAD (ΔG^#^ 10.9 vs. 9.3 kcal/mol). A significant difference between the chemical behavior of nitrile ylides **16** and **20** is that, in the case of nitrile ylide **20,** there is an additional possibility of its transformation into oxazole **21** through a lower energy barrier than that for cycloaddition. Although the formation of oxazole type **21** was not detected during the photolysis of azirine **11a**, we decided to synthesize azirine **28** to ensure that the presence of an additional conversion channel in the case of nitrile ylide **12a** was critical. 

**Scheme 6:**
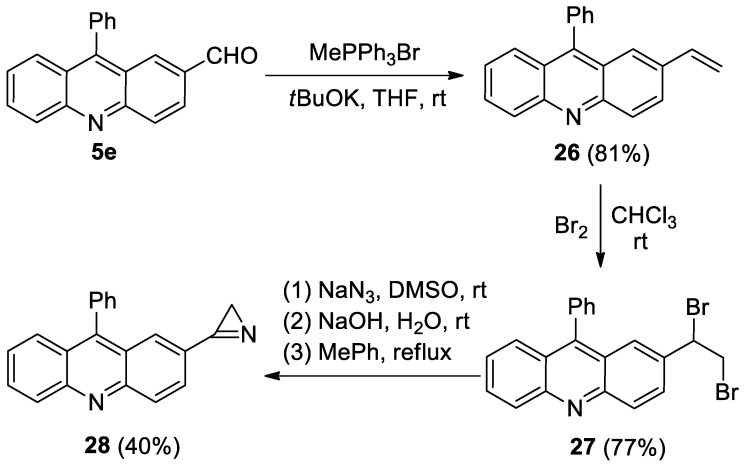
Synthesis of acridinyl-substituted azirine **7j**.

Azirine **28** was prepared the usual way from aldehyde **5e** (Scheme 6). However, the photolysis of azirine **28** in acetonitrile, in the presence of DMAD or PMI (20 equiv.) using LED 365, 380, or 405 nm, led to the same result as the photolysis of azirine **11a**: the starting azirine was isolated unchanged.

**Scheme 7:**
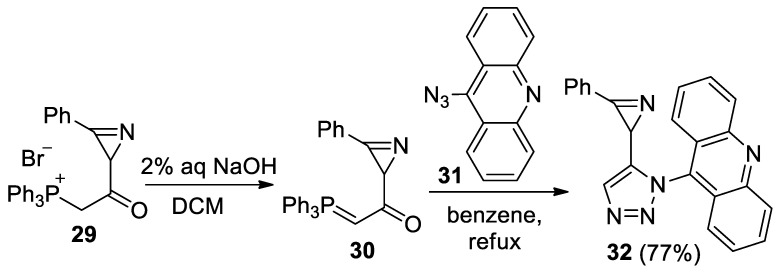
Synthesis of acridinyl-substituted azirine **32**.

Azirine **32**, linked to acridine by a triazole bridge, was also synthesized by the reaction of 1-(3-phenyl-2*H*-azirin-2-yl)-2-(triphenylphosphoranylidene)ethanone **30** with 9-azidoacridine **31**, according to the procedure developed in the work [43], at a 77% yield (Scheme 7). However, the photolysis of azirine **32** in acetonitrile, in the presence of DMAD or PMI (20 equiv.) using LED 365, 380, 405, or 425 nm, led to the same result as the photolysis of azirine **11a**: the starting azirine was isolated unchanged. 

One of the possible reasons for the different behavior of pyrenyl-substituted azirine **15** and acridinyl-substituted azirines **11a**,**28**,**32** during photolysis in the presence of DMAD may be the fast relaxation of the singlet excited state of acridinyl-substituted azirines through intersystem crossing into a triplet excited state, characteristic of acridine systems in acetonitrile [44]. This may prevent the energy required to break the azirine C2–C3 bond from being transferred from the excited acridine moiety to the azirine moiety and block, therefore, the formation of nitrile ylide from azirine.

**Scheme 8:**
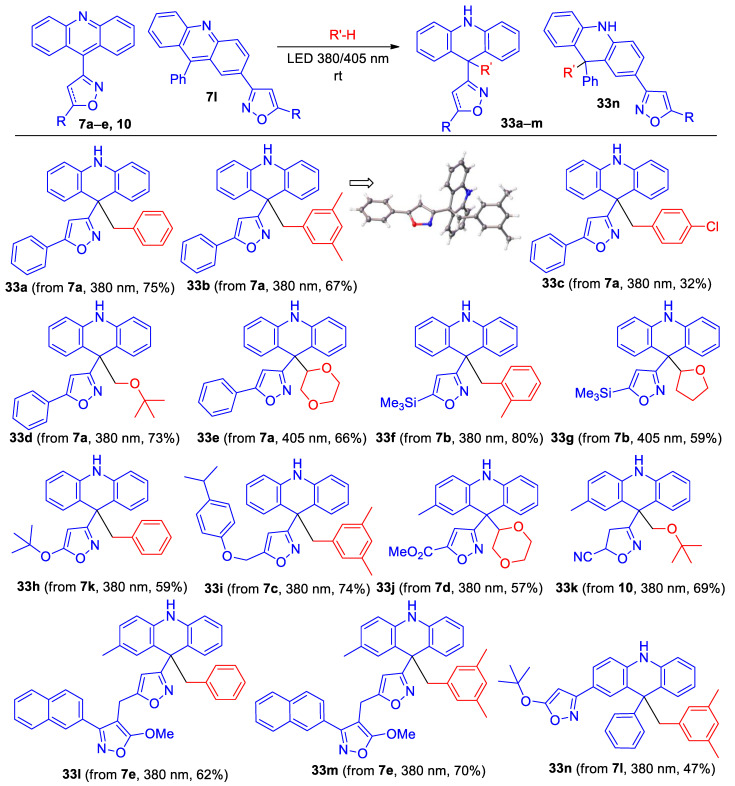
Synthesis of 9,9-disubstituted acridanes **33a**–**n**.

It was previously found that the photolysis of 9-phenylacridine in toluene using a high-pressure mercury lamp leads to 9-benzyl-9-phenyl-9,10-dihydroacridine (49%) [45] through the probable formation and coupling of 9-phenyl-9,10-dihydroacridine-9-yl and benzyl radicals [45,46,47]. To expand the range of isoxazole-substituted acridine derivatives, we decided to carry out the photolysis of isoxazolyl- and azirinyl-substiuted acridines in the presence of hydrogen donor solvents, but by using LED light in the UV/visible radiation boundary region (385–405 nm) instead of with a high-pressure mercury lamp. The irradiation of azirine **28** in toluene under these conditions gave a complex mixture of products, probably because free radicals can open the azirine ring [48]. Fortunately, the irradiation of isoxazolylacridines **7** using LED 385 or 405 nm in the presence of toluene, mesitylene, *o*-xylene, 4-chlorotoluene, THF, MTBE, and 1,4-dioxane gave 9,9-disubstituted acridanes **33a**–**n**, in good yields in most cases, while the reaction with tetrahydrothiophene led to unstable products (Scheme 8).

Note that nitro-substituted derivative **7f** reacts very slowly, and long-term irradiation leads to its decomposition. All new compounds were characterized by ^1^H, ^13^C NMR, and HRMS methods. The structure of acridane **33b** was also confirmed by single-crystal X-ray diffraction analysis.

**Figure 1:**
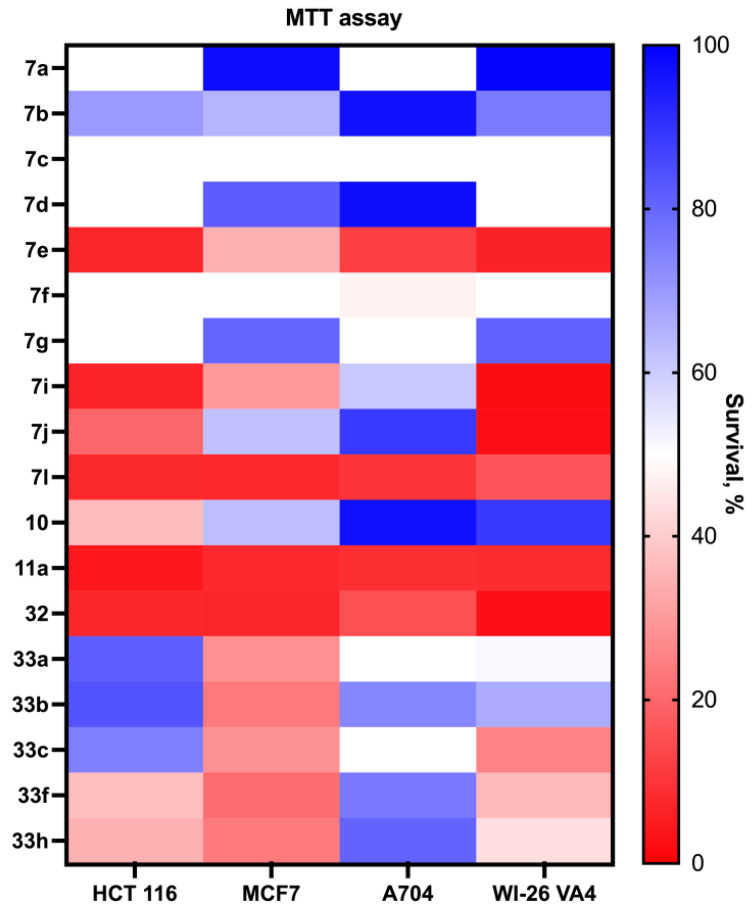
The cytotoxicity of acridine–azirine, acridine–isoxazole, and acridane–isoxazole hybrids.

The evaluation of the antiproliferative activity of the synthesized compounds, including acridine–azirine hybrids, acridine–isoxazoles, and acridane–isoxazole hybrids, was conducted using the MTT assay with a compound concentration of 30 μM over a 72 h period. This comprehensive assessment was performed against a diverse set of cancer cell lines such as HCT 116 (colon cancer), MCF7 (breast cancer), and A704 (kidney cancer), as well as against normal fibroblast cells (WI-26 VA4) to determine the compounds’ selectivity. The findings revealed that the majority of the compounds demonstrated antiproliferative activity, impacting both cancerous and normal cell lines. Notably, derivatives containing the isoxazole moiety, especially compounds **7g**,**i** and **7l**, were identified as having significant cytotoxic activity (Figure 1). However, the lack of pronounced selectivity between the cancerous and normal cells highlights an area for future refinement to improve the possible therapeutic efficacy of these compounds.

## 3. Materials and Methods

### 3.1. General Instrumentation

Melting points were determined on a melting point apparatus. ^1^H (400 MHz) and ^13^C (100 MHz) spectra were recorded on a Bruker AVANCE 400 spectrometer (Billerica, MA, USA) in CDCl_3_ or DMSO-*d*_6_. Chemical shifts (δ) are reported in parts per million downfield from tetramethylsilane (TMS, *δ* = 0.00). ^1^H NMR spectra were calibrated according to the residual peak of H-analogues of CDCl_3_ (7.26 ppm), DMSO-*d_6_* (2.50 ppm), and C_6_D_6_ (7.16 ppm). For all new compounds, ^13^C{^1^H} and ^13^C DEPT-135 spectra were recorded and calibrated according to the peak of CDCl_3_ (77.00 ppm), DMSO-*d*_6_ (39.51 ppm), and C_6_D_6_ (128.06 ppm). Electrospray ionization (ESI) mass spectra were recorded on a Bruker MaXis mass spectrometer, HRMS-ESI-QTOF. Single-crystal X-ray data were collected by the means of “XtaLAB Synergy” diffractometer (Rigaku Oxford Diffraction, Akishima, Japan). Crystallographic data for the structure **33b** (CCDC 2328401) were deposited with the Cambridge Crystallographic Data Centre. Thin-layer chromatography (TLC) was conducted on aluminum sheets with 0.2 mm silica gel and a fluorescent indicator. The physical and spectral data of 7,9-dimethylbenz[*c*]acridine **4b** [49], 9-methyl-2-nitroacridine **4c [50]**, 2-methylacridine-9-carbaldehyde **5a** [51], acridine-9-carbaldehyde oxime **1a** [29], (2-oxo-2-(3-phenyl-2*H*-azirin-2-yl)ethyl)triphenylphosphonium bromide **29 [52]**, and 9-azidoacridine **31 [53]**, prepared according to the published procedures, were in agreement with previously reported values.

### 3.2. General Experimental Procedures

#### 3.2.1. General Procedure A (GP-A) for the Preparation of Chlorooxime Hydrochlorides **2-HCl**

A suspension of oxime **1** in DCM/DMF 20:1 (*v*/*v*) was bubbled with chlorine gas at rt for 0.5–1.5 h (the suspension changed from a brick (orange) color to bright yellow or orange). The reaction mixture was stirred at rt for 1 d in a closed reaction vessel and diluted with DCM, and the product was filtered, washed with DCM, and dried in air.

#### 3.2.2. General Procedure B (GP-B) for the Preparation of Isoxazoles **7**

Chlorooxime hydrochloride **2-HCl** (1 equiv.), excess of appropriate acetylene or alkene, and DCM/sat. aq. NaHCO_3_ 2:1 (*v*/*v*) were placed in round-bottom flask with a plastic stopper. The suspension was shaken well until the reaction mixture became lighter in color and the chloroxime hydrochloride began to dissolve. Then, the reaction mixture was vigorously stirred at rt overnight. The layers were separated and the water layer was extracted with DCM. The combined organic layers were washed with water and brine, dried over Na_2_SO_4_, and after the evaporation of the solvent, the product was purified by chromatography on silica gel.

#### 3.2.3. General Procedure C (GP-C) for the Reaction of Isoxazoles **7a**–**e**,**l** and **10** with Hydrogen Donor Solvents

Isoxazoles in an appropriate hydrogen donor solvent (when reacting with 4-chlorotoluene, α,α,α-trifluorotoluene was added as a co-solvent) were irradiated using LED 385 or 405 nm at rt (TLC control). The solvent was evaporated and the residue was purified by chromatography on silica gel.

#### 3.2.4. Specific Procedures and Characterization

*2-Methyl-9-phenylacridine* (**4e**). Compound **4e** was prepared according to the published procedure [54] from 4-methyl-*N*-phenylaniline (1.83 g, 10 mmol), benzoic acid (2.44 g, 20 mmol), and anhydrous ZnCl_2_ (2.72 g, 20 mmol) at 220 °C overnight to give a pure product of 1.62 g (60% yield), after column chromatography on silica (light petroleum/ethyl acetate, 5:1–1:1, (*v*/*v*)) as a light yellow solid: mp 110–111 °C (ethyl acetate); ^1^H NMR (400 MHz, CDCl_3_) *δ* 8.26 (d, 1H, *J* = 8.8 Hz), 8.18 (d, 1H, *J* = 8.9 Hz), 7.73 (ddd, 1H, *J* = 8.5, 6.5, 1.3 Hz), 7.66 (dd, 1H, *J* = 8.8, 1.3 Hz), 7.67–7.58 (m, 4H), 7.45–7.38 (m, 4H), 2.46 (s, 3H); ^13^C{^1^H} NMR (100 MHz, CDCl_3_) *δ* 148.3 (C), 147.8 (C), 146.0 (C), 136.2 (C), 135.5 (C), 132.9 (CH), 130.5 (CH), 129.6 (CH), 129.5 (CH), 129.4 (CH), 128.5 (CH), 128.2 (CH), 126.8 (CH), 125.5 (CH), 125.3 (C), 125.2 (C), 124.8 (CH), 22.0 (CH_3_); HRMS (ESI) *m/z* [M + H]^+^ calcd for C_20_H_16_N^+^ 270.1277, found 270.1280.
*2-Nitroacridine-9-carbaldehyde* (**5c**). Compound **5c** was prepared according to the published procedure [55] from 9-methyl-2-nitroacridine **4c** (325 mg, 1.35 mmol) and SeO_2_ (160 mg, 1.43 mmol) in 1,4-dioxane (5 mL) at 115 °C (bath temperature) to give a pure product of 214 mg (62% yield), after column chromatography on silica (light petroleum/ethyl acetate, 1:1–0:1, (*v*/*v*)) as a brown-orange solid: mp 182–184 °C (ethyl acetate); ^1^H NMR (400 MHz, DMSO-*d*_6_) *δ* 11.52 (s, 1H), 9.78 (d, 1H, *J* = 2.6 Hz), 8.94 (d, 1H, *J* = 8.8 Hz), 8.55 (dd, 1H, *J* = 9.4, 2.7 Hz), 8.44 (d, 1H, *J* = 9.6 Hz), 8.34 (d, 1H, *J* = 8.7 Hz), 8.09–8.06 (m, 1H), 7.92–7.88 (m, 1H); ^13^C{^1^H} NMR (100 MHz, DMSO-*d*_6_) *δ* 195.1 (CH), 150.9 (C), 148.9 (C), 146.3 (C), 136.0 (C), 132.6 (CH), 131.8 (CH), 130.1 (CH), 129.7 (CH), 124.4 (C), 124.1 (CH), 123.2 (CH), 122.9 (CH), 120.4 (C); HRMS (ESI) *m/z* [M + H]^+^ calcd for C_14_H_9_N_2_O_3_^+^ 253.0608, found 253.0599.
*9-Methylbenzo[c]acridine-7-carbaldehyde* (**5d**). Compound **5d** was prepared according to the published procedure [55] from 7,9-dimethylbenzo[*c*]acridine **4d** (1.5 g, 7.24 mmol) and SeO_2_ (843 mg, 7.6 mmol) in 1,4-dioxane (50 mL) at 115 °C (bath temperature) to give a pure product of 1.32 g (82% yield), after column chromatography on silica (light petroleum/ethyl acetate, 1:1–0:1, (*v*/*v*)) as an orange solid: mp 165–166 °C (ethyl acetate); ^1^H NMR (400 MHz, CDCl_3_) *δ* 11.44 (d, 1H, *J* = 6.7 Hz), 9.50–9.47 (m, 1H), 8.49 (d, 1H, *J* = 4.5 Hz), 8.45–8.41 (m, 1H), 8.33 (dd, 1H, *J* = 8.7, 5.0 Hz), 7.88–7.73 (m, 4H), 7.69 (dt, 1H, *J* = 9.3, 2.2 Hz), 2.64 (s, 3H); ^13^C{^1^H} NMR (100 MHz, CDCl_3_) *δ* 193.9 (CH), 146.9 (C), 146.4 (C), 138.8 (C), 132.7 (C), 132.2 (CH), 131.4 (C), 131.0 (C), 130.8 (CH), 130.3 (CH), 129.2 (CH), 127.79 (CH), 127.75 (CH), 125.2 (CH), 123.6 (C), 123.1 (C), 121.8 (CH), 120.3 (CH), 22.4 (CH_3_); HRMS (ESI) *m*/*z* [M + H]^+^ calcd for C_19_H_14_NO^+^ 272.1070, found 272.1065.
*9-Phenylacridine-2-carbaldehyde* (**5e**). Compound **5e** was prepared according to the published procedure [55] from 2-methyl-9-phenylacridine **4e** (1.0 g, 3.7 mmol) and SeO_2_ (4.3 g, 38.7 mmol, 6 portions every 12 h) 1,4-dioxane (35 mL) at 115 °C (bath temperature) to give a pure product of 714 mg (68% yield), after column chromatography on silica (light petroleum/ethyl acetate, 1:1–0:1, (*v*/*v*)) as a light yellow solid: mp 185–186 °C (ethyl acetate); ^1^H NMR (400 MHz, CDCl_3_) *δ* 10.02 (d, 1H, *J* = 0.8 Hz), 8.32 (td, 2H, *J* = 9.6, 0.8 Hz), 8.23–8.20 (m, 2H), 7.77–7.75 (m, 1H), 7.68–7.64 (m, 3H), 7.52–7.47 (, 3H); ^13^C{^1^H} NMR (CDCl_3_, 100 MHz) *δ* 191.5 (CH), 150.5 (C), 150.4 (C), 149.9 (C), 135.4 (CH), 134.9 (C), 133.7 (C), 131.5 (CH), 131.1 (CH), 130.4 (CH), 129.8 (CH), 129.0 (CH), 128.7 (CH), 127.2 (CH), 126.5 (CH), 125.7 (CH), 125.5 (C), 124.3 (C); HRMS (ESI) *m/z* [M + H]^+^ calcd for C_20_H_14_NO^+^ 284.1070, found 284.1062.
*2-Methylacridine-9-carbaldehyde oxime* (**1b**). Compound **1b** was prepared according to the published procedure [29] from acridinecarbaldehyde **5b** (1.0 g, 4.5 mmol) and H_2_NOH·HCl (628 mg, 9 mmol) in EtOH (20 mL) and 2% aq. NaOH (2 mL) to give a pure product of 885 mg (83% yield), after filtration as a brick-yellow solid: mp 196–197 °C (EtOH/H_2_O); ^1^H NMR (400 MHz, DMSO-*d*_6_) *δ* 12.35 (s, 1H), 9.28 (s, 1H), 8.55 (d, 1H, *J* = 8.8 Hz), 8.31–8.30 (m, 1H), 8.19 (d, 1H, *J* = 8.6 Hz), 8.12 (d, 1H, *J* = 8.9 Hz), 7.89 (ddd, 1H, *J* = 8.4, 6.5, 1.4 Hz), 7.77 (dd, 1H, *J* = 8.9, 1.9 Hz), 7.68 (ddd, 1H, *J* = 8.2, 6.6, 1.3 Hz), 2.56 (s, 1H); ^13^C{^1^H} NMR (100 MHz, DMSO-*d*_6_) *δ* 146.3 (br. s, C), 145.8 (br. s, C), 145.1 (CH), 136.8 (C), 135.1 (br. s, C), 133.9 (CH), 130.7 (CH), 128.1 (br. s, CH), 127.9 (br. s, CH), 126.9 (CH), 125.8 (CH), 123.64 (CH), 123.61 (C), 123.56 (C), 21.7 (CH_3_); HRMS (ESI) *m/z* [M + H]^+^ calcd for C_15_H_13_N_2_O^+^ 237.1022, found 237.1017.
*2-Nitroacridine-9-carbaldehyde oxime* (**1c**). Compound **1c** was prepared according to the published procedure [29] from acridinecarbaldehyde **5c** (200 mg, 0.8 mmol) and H_2_NOH·HCl (138 mg, 2 mmol) in EtOH (5 mL) and 2% aq. NaOH (0.5 mL) to give a pure product of 162 mg (76% yield), after filtration as a yellow-green solid: mp 202–214 °C (dec., EtOH/H_2_O); ^1^H NMR (400 MHz, DMSO-*d*_6_) *δ* 12.67 (s, 1H), 9.65 (d, 1H, *J* = 2.5 Hz), 9.44 (s, 1H), 8.56 (d, 1H, *J* = 8.8 Hz), 8.49 (dd, 1H, *J* = 9.5, 2.7 Hz), 8.34 (d, 1H, *J* = 9.5 Hz), 8.24 (d, 1H, *J* = 8.6 Hz), 8.01 (t, 1H, *J* = 7.7 Hz), 7.77 (t, 1H, *J* = 7.7 Hz); ^13^C{^1^H} NMR (100 MHz, DMSO-*d*_6_) *δ* 150.0 (C), 148.7 (C), 145.0 (C), 144.5 (CH), 141.7 (CH), 137.7 (C), 132.0 (CH), 131.4 (CH), 129.5 (CH), 127.5 (CH), 124.9 (CH), 124.0 (CH), 122.3 (CH), 120.9 (C); HRMS (ESI) *m/z* [M + H]^+^ calcd for C_14_H_10_N_3_O_3_^+^ 268.0717, found 268.0719.
*9-Methylbenzo[c]acridine-7-carbaldehyde oxime* (**1d**). Compound **1d** was prepared according to the published procedure [29] from acridinecarbaldehyde **5d** (935 mg, 3.5mmol) and H_2_NOH·HCl (600 mg, 8.6 mmol) in EtOH (20 mL) and 2% aq. NaOH (2 mL) to give a pure product of 984 mg (99% yield), after filtration as a brick-orange solid: mp 237–238 °C (EtOH/H_2_O); ^1^H NMR (400 MHz, DMSO-*d*_6_) *δ* 12.02 (br. s, 1H), 9.42–9.40 (m, 1H), 9.23 (s, 1H), 8.31–8.27 (m, 3H), 8.02–8.00 (m, 1H), 7.90 (d, 1H, *J* = 9.5 Hz), 7.83–7.78 (m, 3H), 2.61 (s, 3H); ^13^C{^1^H} NMR (100 MHz, DMSO-*d*_6_) *δ* 145.5 (C), 145.2 (CH), 145.0 (C), 136.9 (C), 133.9 (C), 133.0 (C), 132.9 (CH), 130.3 (C), 129.5 (CH), 129.0 (CH), 128.3 (CH), 128.0 (CH), 127.6 (CH), 124.8 (CH), 124.2 (C), 123.8 (CH), 122.9 (CH), 122.3 (C), 21.7 (CH_3_); HRMS (ESI) *m/z* [M + H]^+^ calcd for C_19_H_15_N_2_O^+^ 287.1179, found 287.1170.
*9-Phenylacridine-2-carbaldehyde oxime* (**1e**). Compound **1e** was prepared according to the published procedure [29] from acridinecarbaldehyde **5e** (693 mg, 2.5 mmol) and H_2_NOH·HCl (425 mg, 6.1 mmol) in EtOH (20 mL) and 2% aq. NaOH (2 mL) to give a pure product of 639 mg (88% yield), after filtration as a bright yellow solid: mp 212–215 °C (dec., EtOH/H_2_O); ^1^H NMR (400 MHz, DMSO-*d*_6_) *δ* 11.71 (br. s, 1H), 8.62–8.42 (m, 3H0, 8.34 (s, 1H), 8.24–8.16 (m, 1H), 7.92–7.90 (m,1H), 7.81–7.73 (m, 5H), 7.61–7.57 (m, 2H); ^13^C{^1^H} NMR (100 MHz, DMSO-*d*_6_) *δ* 155.8 (br. s, C), 147.2 (CH), 142.1 (br. s, C), 141.4 (br. s, C), 135.2 (br. s, CH), 133.3 (C), 132.4 (br. s, C), 132.3 (CH), 130.0 (CH), 129.8 (CH), 128.9 (CH), 128.0 (CH), 127.6 (CH), 125.5 (CH), 125.2 (C), 124.9 (C), 123.1 (br. s, CH), 122.3 (br. s, CH); HRMS (ESI) *m/z* [M + H]^+^ calcd for C_20_H_15_N_2_O^+^ 299.1179, found 299.1183.
*N-Hydroxyacridine-9-carbimidoyl chloride hydrochloride* (**2a-HCl**). Compound **2a-HCl** was prepared according to the general procedure GP-A from oxime **1a** (2.0 g, 9 mmol) in DCM (30 mL) and DMF (3 mL) to give a pure product of 1.84 g (70% yield), after filtration as a bright yellow solid: mp 226–230 °C (dec., DCM); ^1^H NMR (400 MHz, DMSO-d_6_) *δ* 13.29 (s, 1H), 8.42 (d, 2H, *J* = 8.7 Hz), 8.14–8.07 (m, 4H), 7.89–7.85 (m, 2H), 4.46 (br. s); ^13^C{^1^H} NMR (100 MHz, DMSO-d_6_) *δ* 144.9 (C), 140.4 (C), 133.6 (CH), 128.7 (CH), 127.4 (C), 125.8 (CH), 125.2 (CH), 123.6 (C); HRMS (ESI) *m/z* [M − Cl]^+^ calcd for C_14_H_10_ClN_2_O^+^ 257.0476, found 257.0482.
*N-Hydroxy-2-methylacridine-9-carbimidoyl chloride hydrochloride* (**2b-HCl**). Compound **2b-HCl** was prepared according to the general procedure GP-A from oxime **1b** (842 mg, 2.3 mmol) in DCM (20 mL) and DMF (2 mL) to give a pure product of 838 mg (83% yield), after filtration as a bright yellow-orange solid: mp 231–232 °C (dec., DCM); ^1^H NMR (400 MHz, DMSO-*d*_6_) *δ* 13.33 (s, 1H), 8.46–8.44 (m, 2H), 8.39 (d, 1H, *J* = 9.0 Hz), 8.12–8.08 (m, 2H), 7.99 (dd, 1H, *J* = 8.9, 1.9 Hz), 7.90–7.86 (m, 2H), 7.61 (s, 3H), 5.81 (br. s); ^13^C{^1^H} NMR (100 MHz, DMSO-*d*_6_) *δ* 143.2 (C), 142.8 (C), 140.0 (C), 138.8 (C), 136.6 (CH), 133.3 (CH), 128.4 (CH), 127.0 (C), 124.9 (CH), 124.6 (CH), 124.4 (CH), 123.6 (C), 123.6 (C), 122.8 (CH), 21.4 (CH_3_); HRMS (ESI) *m/z* [M − Cl]^+^ calcd for C_14_H_20_ClN_2_O^+^ 257.0476, found 257.0482.
*N-Hydroxy-2-nitroacridine-9-carbimidoyl chloride hydrochloride* (**2c-HCl**). Compound **2c-HCl** was prepared according to the general procedure GP-A from oxime **1c** (155 mg, 0.6 mmol) in DCM (3 mL) and DMF (0.3 mL) to give a pure product of 136 mg (69% yield), after filtration as an orange-brown solid: mp 229–230 °C (dec., DCM); ^1^H NMR (400 MHz, DMSO-*d*_6_) *δ* 13.47 (s, 1H), 8.86 (t, 1H, *J* = 2.0 Hz), 8.55 (dt, 1H, *J* = 9.4, 2.4 Hz), 8.45 (dd, 1H, *J* = 9.4, 2.2 Hz), 8.32 (d, 1H, *J* = 8.7 Hz), 8.12 (d, 1H, *J* = 8.8 Hz), 8.10–8.06 (m, 1H), 7.88 (dd, 1H, *J* = 8.7, 6.7 Hz), 7.51–6.61 (br. s); ^13^C{^1^H} NMR (100 MHz, DMSO-*d*_6_) *δ* 150.2 (C), 148.4 (C), 145.6 (C), 139.9 (C), 133.3 (CH), 131.7 (CH), 129.6 (CH), 129.4 (CH), 127.4 (C), 125.1 (CH), 123.9 (C), 123.7 (CH), 122.0 (CH), 121.5 (C); HRMS (ESI) *m/z* [M − Cl]^+^ calcd for C_14_H_9_ClN_3_O_3_^+^ 302.0327, found 302.0320.
*N-Hydroxy-9-methylbenzo[c]acridine-7-carbimidoyl chloride hydrochloride* (**2d-HCl**). Compound **2d-HCl** was prepared according to the general procedure GP-A from oxime **1d** (855 mg, 3 mmol) in DCM (30 mL) and DMF (3 mL) to give a pure product of 908 mg (85% yield), after filtration as a bright yellow solid: mp 267–272 °C (dec., DCM); ^1^H NMR (400 MHz, DMSO-*d*_6_) *δ* 13.07 (s, 1H), 9.39–9.37 (m, 1H), 8.31 (d, 1H, *J* = 8.7 Hz), 8.07–8.04 (m, 1H), 8.01 (d, 1H, *J* = 9.4 Hz), 7.86–7.76 (m, 5H), 5.61–5.16 (br. s), 2.61 (s, 3H); ^13^C{^1^H} NMR (100 MHz, DMSO-*d*_6_) *δ* 146.0 (C), 145.6 (C), 138.0 (C), 135.1 (C), 133.3 (CH), 133.0 (C), 130.5 (C), 129.78 (CH), 129.75 (CH), 129.5 (CH), 128.8 (C), 128.4 (CH), 128.1 (CH), 124.6 (CH), 123.8 (C), 122.6 (CH), 122.2 (C), 121.8 (CH), 21.7 (CH_3_); HRMS (ESI) *m/z* [M − Cl]^+^ calcd for C_19_H_14_ClN_2_O^+^ 321.0789, found 321.0801.
*N-Hydroxy-9-phenylacridine-2-carbimidoyl chloride hydrochloride* (**2e-HCl**). Compound **2e-HCl** was prepared according to the general procedure GP-A from oxime **1e** (623 mg, 2.1 mmol) in DCM (20 mL) and DMF (2 mL) to give a pure product of 721 mg (94% yield), after filtration as a bright yellow solid: mp 228–229 °C (dec., DCM); ^1^H NMR (400 MHz, DMSO-*d*_6_) *δ* 12.94 (s, 1H), 8.63–8.56 (m, 3H), 8.27–8.23 (m, 1H), 8.16 (d, 1H, *J* = 1.9 Hz), 7.83 (d, 1H, *J* = 3.9 Hz), 7.79–7.75 (m, 3H), 7.64–7.62 (m, 2H); ^13^C{^1^H} NMR (100 MHz, DMSO-*d*_6_) *δ* 156.7 (C), 142.0 (C), 141.8 (C), 135.9 (CH), 134.4 (C), 133.1 (C), 132.1 (CH), 131.1 (C), 130.0 (CH), 130.0 (CH), 128.9 (CH), 128.3 (CH), 127.7 (CH), 125.8 (CH), 125.3 (C), 124.4 (C), 123.0 (CH), 122.3 (CH); HRMS (ESI) *m/z* [M + H]^+^ calcd for C_20_H_14_ClN_2_O^+^ 333.0789, found 333.0777.
*3-(Acridin-9-yl)-5-phenylisoxazole* (**7a**). Compound **7a** was prepared according to the general procedure GP-B from chlorooxime **2a-HCl** (171 mg, 0.58 mmol) and phenylacetylene **6a** (298 mg, 2.92 mmol, 5 eq.) in DCM (10 mL) and sat. aq. NaHCO_3_ (5 mL) to give a pure product of 178 mg (95% yield), after column chromatography on silica (light petroleum/ethyl acetate, 6:1, (*v*/*v*)) as a bright yellow solid: mp 258–260 °C (light petroleum/ethyl acetate); ^1^H NMR (400 MHz, CDCl_3_): *δ* 8.32 (d, 2H, *J* = 8.7 Hz), 8.01 (d, 2H, *J* = 8.7 Hz), 7.96–7.94 (m, 2H), 7.83 (ddd, 2H, *J* = 8.5, 6.6, 1.4 Hz), 7.58–7.53 (m, 5H), 6.85 (s, 1H); ^13^C{^1^H} NMR (100 MHz, CDCl_3_) *δ* 170.8 (C), 159.4 (C), 148.7 (C), 133.7 (C), 130.8 (CH), 130.3 (CH), 129.9 (CH), 129.2 (CH), 127.0 (C), 126.8 (CH), 126.1 (CH), 125.8 (CH), 125.0 (C), 102.6 (CH); HRMS (ESI) *m/z* [M + H]^+^ calcd for C_29_H_23_N_2_O^+^ 415.1805, found 415.1799.
*3-(Acridin-9-yl)-5-(trimethylsilyl)isoxazole* (**7b**). Compound **7b** was prepared according to the general procedure GP-B from chlorooxime **2a-HCl** (255 mg, 0.87 mmol) and trimethylsilylacetylene **6b** (1.94 g, 17.4 mmol, 20 eq.) in DCM (15 mL) and sat. NaHCO_3_ aq. (8 mL) to give a pure product of 256 mg (92% yield), after column chromatography on silica (light petroleum/ethyl acetate, 6:1, (*v*/*v*)) as a bright yellow solid: mp 151–152 °C (light petroleum/ethyl acetate); ^1^H NMR (400 MHz, CDCl_3_) *δ* 8.29 (dt, 2H, *J* = 8.8, 1.0 Hz), 7.87 (ddd, 2H, *J* = 8.7, 1.4, 0.7 Hz), 7.80 (ddd, 2H, *J* = 8.9, 6.6, 1.4 Hz), 7.52 (ddd, 2H, *J* = 8.7, 6.6, 1.2 Hz), 6.74 (s, 1H), 0.49 (s, 9H); ^13^C{^1^H} NMR (100 MHz, CDCl_3_) *δ* 179.1 (C), 156.9 (C), 148.6 (C), 134.1 (C), 130.2 (CH), 129.8 (CH), 126.5 (CH), 125.9 (CH), 125.1 (C), 115.4 (CH), −1.8 (CH_3_); HRMS (ESI) *m/z* [M + H]^+^ calcd for C_19_H_19_N_2_OSi^+^ 319.1261, found 319.1258.
*3-(Acridin-9-yl)-5-((4-isopropylphenoxy)methyl)isoxazole* (**7c**). Compound **7c** was prepared according to the general procedure GP-B from chlorooxime **2a-HCl** (155 mg, 0.51 mmol) and 1-isopropyl-4-(prop-2-yn-1-yloxy)benzene **6c** (446 mg, 2.56 mmol, 5 eq.) in DCM (10 mL) and sat. NaHCO_3_ aq. (5 mL) to give a pure product of 198 mg (98% yield), after column chromatography on silica (light petroleum/ethyl acetate, 10:1, (*v*/*v*)) as a bright yellow solid: mp 94–95 °C (light petroleum/ethyl acetate); ^1^H NMR (400 MHz, CDCl_3_) *δ* 8.30 (d, 2H, *J* = 8.8 Hz), 7.91 (d, 2H, *J* = 8.7 Hz), 7.83–7.79 (m, 2H), 7.56–7.52 (m, 2H), 7.21 (d, 2H, *J* = 8.2 Hz), 6.99 (d, 2H, *J* = 8.2 Hz), 6.67 (s, 1H), 5.37 (s, 2H), 2.90 (hept, 1H, *J* = 7.0 Hz), 1.25 (d, 6H, *J* = 7.0 Hz); ^13^C{^1^H} NMR (100 MHz, CDCl_3_) *δ* 169.2 (C), 158.9 (C), 155.8 (C), 148.6 (C), 142.7 (C), 133.3 (C), 130.3 (CH), 129.9 (CH), 127.6 (CH), 126.8 (CH), 125.7 (CH), 124.9 (C), 114.8 (CH), 106.4 (CH), 61.8 (CH_2_), 33.3 (CH), 24.1 (CH_3_); HRMS (ESI) *m/z* [M + H]^+^ calcd for C_26_H_23_N_2_O_2_^+^ 395.1754, found 395.1752.
*Methyl 3-(2-methylacridin-9-yl)isoxazole-5-carboxylate* (**7d**). Compound **7d** was prepared according to the general procedure GP-B from chlorooxime **2b-HCl** (330 mg, 1.1 mmol) and methyl propiolate **6d** (452 mg, 5.4 mmol, 5 eq.) in DCM (20 mL) and sat. NaHCO_3_ aq. (10 mL) to give a pure product of 179 mg (52% yield), after column chromatography on silica (light petroleum/ethyl acetate, 6:1, (*v*/*v*)) as a yellow solid: mp 169–170 °C (light petroleum/ethyl acetate); ^1^H NMR (400 MHz, CDCl_3_) *δ* 8.28 (dd, 1H, *J* = 9.1, 1.2 Hz), 8.20 (d, 1H, *J* = 8.9 Hz), 7.80–776 (m, 2H), 7.65 (dd, 1H, *J* = 8.9, 1.6 Hz), 7.55–7.51 (m, 2H), 7.25 (s, 1H), 4.08 (s, 3H), 2.52 (s, 3H); ^13^C{^1^H} NMR (100 MHz, CDCl_3_) *δ* 160.9 (C), 159.6 (C), 157.0 (C), 148.0 (C), 147.6 (C), 137.3 (C), 133.3 (CH), 130.5 (C), 130.0 (CH), 129.8 (CH), 129.7 (CH), 127.0 (CH), 125.0 (CH), 124.8 (C), 124.8 (C), 123.1 (CH), 112.3 (CH), 53.1 (CH_3_), 22.1 (CH_3_); HRMS (ESI) *m/z* [M + H]^+^ calcd for C_19_H_15_N_2_O_3_^+^ 319.1077, found 319.1076.
*5-Methoxy-4-((3-(2-methylacridin-9-yl)isoxazol-5-yl)methyl)-3-(naphthalen-2-yl)isoxazole* (**7e**). Compound **7e** was prepared according to the general procedure GP-B from chlorooxime **2b-HCl** (150 mg, 0.49 mmol) and 5-methoxy-3-(naphthalen-2-yl)-4-(prop-2-yn-1-yl)isoxazole **6e** (296 mg, 1.12 mmol, 2.3 eq.) in DCM (20 mL) and sat. NaHCO_3_ aq. (10 mL) to give a pure product of 227 mg (93% yield), after column chromatography on silica (light petroleum/ethyl acetate, 20:1–6:1, (*v*/*v*)) as a bright yellow solid: mp 157–158 °C (light petroleum/ethyl acetate); ^1^H NMR (400 MHz, CDCl_3_) *δ* 8.21 (d, 1H, *J* = 8.7 Hz), 8.14 (d, 1H, *J* = 8.7 Hz), 8.13 (s, 1H), 7.96 (d, 1H, *J* = 8.5 Hz), 7.92–7.89 (m, 2H), 7.78 (dd, 1H, *J* = 8.5, 1.8 Hz), 7.70 (ddd, 1H, *J* = 8.1, 6.7, 1.3 Hz), 7.61–7.52 (m, 5H), 7.24–7.20 (m, 1H), 6.23 (s, 1H), 4.25 (s, 3H), 4.18 (s, 2H), 2.40 (s, 3H); ^13^C{^1^H} NMR (100 MHz, CDCl_3_) *δ* 171.3 (C), 170.4 (C), 164.3 (C), 159.0 (C), 147.8 (C), 147.4 (C), 136.7 (C), 133.8 (C), 133.1 (CH), 133.0 (C), 132.2 (C), 129.6 (CH), 129.6 (CH), 129.4 (CH), 128.8 (CH), 128.5 (CH), 127.8 (CH), 127.6 (CH), 127.2 (CH), 126.8 (CH), 126.7 (C), 126.4 (CH), 125.3 (CH), 124.9 (C), 124.8 (C), 124.6 (CH), 123.5 (CH), 105.2 (CH), 85.9 (CH), 58.1 (CH_3_), 22.0 (CH_3_), 20.1 (CH_2_); HRMS (ESI) *m/z* [M + H]^+^ calcd for C_32_H_24_N_3_O_3_^+^ 498.1812, found 498.1807.
*5-(4-Chlorophenyl)-3-(2-nitroacridin-9-yl)isoxazole* (**7f**). Compound **7f** was prepared according to the general procedure GP-B from chlorooxime **2c-HCl** (150 mg, 0.44 mmol) and 1-chloro-4-ethynylbenzene **6f** (305 mg, 2.2 mmol, 5 eq.) in DCM (20 mL) and sat. aq. NaHCO_3_ (10 mL) to give a pure product of 159 mg (89% yield), after column chromatography on silica (light petroleum/ethyl acetate, 6:1–0:1, (*v*/*v*)) as a bright yellow solid: mp 232–233 °C (ethyl acetate); ^1^H NMR (400 MHz, DMSO-*d*_6_) *δ* 8.90 (d, 1H, *J* = 2.5 Hz), 8.55 (dd, 1H, *J* = 9.5, 2.5 Hz), 8.48 (d, 1H, *J* = 9.5 Hz), 8.36 (d, 1H, *J* = 8.8 Hz), 8.11–8.05 (m, 5H), 7.79 (dd, 1H, *J* = 8.7, 6.6 Hz), 7.71–7.69 (m, 2H); ^13^C{^1^H} NMR (CDCl_3_, 100 MHz) *δ* 169.3 (C), 158.0 (C), 149.9 (C), 148.4 (C), 145.1 (C), 136.4 (C), 135.5 (C), 132.4 (CH), 131.5 (CH), 129.4 (CH), 129.1 (CH), 128.2 (CH), 127.5 (CH), 125.6 (CH), 124.9 (C), 124.4 (C), 122.9 (CH), 122.8 (CH), 122.0 (C), 103.9 (CH); HRMS (ESI) *m/z* [M + H]^+^ calcd for C_22_H_13_ClN_3_O_3_^+^ 402.0640, found 402.0635.
*5-(2-Fluorophenyl)-3-(9-methylbenzo[c]acridin-7-yl)isoxazole* (**7g**). Compound **7g** was prepared according to the general procedure GP-B from chlorooxime **2d-HCl** (170 mg, 0.48 mmol) and 1-ethynyl-2-fluorobenzene **6g** (230 mg, 1.9 mmol, 4 eq.) in DCM (10 mL) and sat. NaHCO_3_ aq. (5 mL) to give a pure product of 174 mg (90% yield), after column chromatography on silica (light petroleum/ethyl acetate, 6:1, (*v*/*v*)) as a brick-yellow solid: mp 234–235 °C (ethyl acetate); ^1^H NMR (CDCl_3_, 400 MHz) *δ* 9.56 (d, 1H, *J* = 7.9 Hz), 8.36–8.34 (m, 1H), 8.21 (td, 1H, *J* = 7.6, 1.8 Hz), 7.86 (dd, 1H, *J* = 7.6, 1.5 Hz), 7.82–7.78 (m, 1H), 7.76–7.68 (m, 5H), 7.52 (tdd, 1H, *J* = 7.5, 5.1, 1.8 Hz), 7.40 (td, 1H, *J* = 7.5, 1.2 Hz), 7.29–7.25 (m, 1H), 7.06 (d, 1H, *J* = 3.8 Hz), 2.55 (s, 3H); ^13^C{^1^H} NMR (CDCl_3_, 100 MHz) *δ* 164.5 (d, C, *J* = 2.5 Hz), 160.1 (C), 159.3 (d, C, *J* = 253.6 Hz), 146.6 (C), 146.0 (C), 136.9 (C), 133.3 (C), 132.4 (CH), 132.1 (d, CH, *J* = 8.6 Hz), 131.55 (C), 131.48 (C), 129.9 (CH), 129.0 (CH), 128.6 (CH), 127.84 (CH), 127.82 (CH), 127.5 (CH), 125.4 (C), 125.3 (CH), 124.9 (d, CH, *J* = 3.6 Hz), 123.8 (CH), 123.6 (C), 122.9 (CH), 116.4 (d, CH, *J* = 21.0 Hz), 115.6 (d, C, *J* = 12.3 Hz), 106.7 (d, CH, *J* = 11.3 Hz), 22.3 (CH_3_); HRMS (ESI) *m/z* [M + H]^+^ calcd for C_27_H_18_FN_2_O^+^ 405.1398, found 405.1392.
*5-((4-Isopropylphenoxy)methyl)-3-(9-methylbenzo[c]acridin-7-yl)isoxazole* (**7h**). Compound **7h** was prepared according to the general procedure GP-B from chlorooxime **2d-HCl** (150 mg, 0.58 mmol) and 1-isopropyl-4-(prop-2-yn-1-yloxy)benzene **6c** (370 mg, 2.1 mmol, 5 eq.) in DCM (10 mL) and sat. NaHCO_3_ aq. (5 mL) to give a pure product of 157 mg (95% yield), after column chromatography on silica (light petroleum/ethyl acetate, 10:1, (*v*/*v*)) as a bright yellow solid: mp 169–170 °C (light petroleum/ethyl acetate); ^1^H NMR (400 MHz, CDCl_3_) *δ* 9.58 (br. s, 1H), 8.38 (br. s, 1H), 7.87–7.85 (m, 1H), 7.82–7.72 (m, 2H), 7.69 (d, 2H, *J* = 9.2 Hz), 7.63–7.61 (m, 2H), 7.24–7.20 (m, 2H), 7.02–6.98 (m, 2H), 6.66 (s, 1H), 5.39 (s, 2H), 2.91 (hept, 1H, *J* = 7.0 Hz), 2.55 (s, 3H), 1.25 (d, 6H, *J* = 7.0 Hz); ^13^C{^1^H} NMR (100 MHz, CDCl_3_) *δ* 169.1 (C), 159.3 (C), 155.9 (C), 146.6 (C), 146.0 (C), 142.6 (C), 136.9 (C), 133.3 (C), 132.4 (CH), 131.6 (C), 131.3 (C), 129.9 (CH), 129.1 (CH), 128.6 (CH), 127.8 (CH), 127.6 (CH), 127.5 (CH), 125.4 (C), 125.3 (CH), 123.7 (CH), 123.5 (C), 122.9 (CH), 114.8 (CH), 106.4 (CH), 61.9 (CH_2_), 33.3 (CH), 24.2 (CH_3_), 22.1 (CH_3_); HRMS (ESI) *m/z* [M + H]^+^ calcd for C_31_H_27_N_2_O_2_^+^ 459.2068, found 459.2061.
*3-(Acridin-9-yl)-5-chloroisoxazole* (**7i**). Compound **7i** was prepared according to the general procedure GP-B from chlorooxime **2a-HCl** (365 mg, 0.58 mmol) and 1,1-dichloroethylene **8** (2 mL, 24.9 mmol, 20 eq.) in DCM (20 mL) and sat. NaHCO_3_ aq. (10 mL) to give a pure product of 218 mg (62% yield), after column chromatography on silica (light petroleum/ethyl acetate, 6:1, (*v*/*v*)) as a bright yellow solid: mp 181–182 °C (light petroleum/ethyl acetate); ^1^H NMR (400 MHz, CDCl_3_) *δ* 8.31 (dt, 2H, *J* = 8.8, 1.0 Hz), 7.91 (dt, 2H, *J* = 8.8, 1.0 Hz), 7.83 (ddd, 2H, *J* = 8.8, 6.6, 1.4 Hz), 7.52 (ddd, 2H, *J* = 8.7, 6.6, 1.2 Hz), 6.52 (s, 1H); ^13^C{^1^H} NMR (100 MHz, CDCl_3_) *δ* 160.9 (C), 155.9 (C), 148.6 (C), 132.1 (C), 130.3 (CH), 130.0 (CH), 127.1 (CH), 125.3 (CH), 124.7 (C), 104.5 (CH); HRMS (ESI) *m/z* [M + H]^+^ calcd for C_16_H_10_ClN_2_O^+^ 291.0477, found 291.0470.
*5-Chloro-3-(9-phenylacridin-2-yl)isoxazole* (**7j**). Compound **7j** was prepared according to the general procedure GP-B from chlorooxime **2d-HCl** (620 mg, 1.7 mmol) and 1,1-dichloroethylene **8** (2.7 mL, 33.6 mmol, 20 eq.) in DCM (30 mL) and sat. aq. NaHCO_3_ (15 mL) to give a pure product of 381 mg (64% yield), after column chromatography on silica (light petroleum/ethyl acetate, 6:1, (*v*/*v*)) as a bright yellow solid: mp 159–160 °C (light petroleum/ethyl acetate); ^1^H NMR (400 MHz, CDCl_3_) *δ* 8.36 (d, 1H, *J* = 9.0 Hz), 8.29 (d, 1H, *J* = 8.8 Hz), 8.23–8.20 (m, 1H), 8.01 (d, 1H, *J* = 1.9 Hz), 7.82 (ddd, 1H, *J* = 8.6, 6.6, 1.4 Hz), 7.72 (dd, 1H, *J* = 8.8, 1.3 Hz), 7.68–7.61 (m, 3H), 7.49–7.45 (m, 3H), 6.41 (s, 1H); ^13^C{^1^H} NMR (CDCl_3_, 100 MHz) *δ* 163.7 (C), 155.3 (C), 149.5 (C), 149.0 (C), 148.2 (C), 135.2 (C), 130.8 (CH), 130.7 (CH), 130.4 (CH), 129.7 (CH), 128.8 (CH), 128.7 (CH), 127.3 (CH), 127.0 (CH), 126.2 (CH), 125.8 (CH), 125.5 (C), 125.4 (C), 124.6 (C), 99.6 (CH); HRMS (ESI) *m/z* [M + H]^+^ calcd for C_22_H_14_ClN_2_O^+^ 357.0789, found 357.0785.
*3-(Acridin-9-yl)-5-(tert-butoxy)isoxazole* (**7k**). Compound **7k** was prepared according to the published procedure [56] from chloroisoxazole **7i** (165 mg, 0.59 mmol) and *t*BuOK (100 mg, 0.88 mmol) in THF (6 mL) to give a pure product of 128 mg (68% yield), after column chromatography on silica (light petroleum/ethyl acetate, 8:1, (*v*/*v*)) as a colorless solid: mp 166–167 °C (light petroleum/ethyl acetate); ^1^H NMR (400 MHz, CDCl_3_) *δ* 8.28 (dt, 2H, *J* = 8.9, 0.9 Hz), 8.02 (dt, 2H, *J* = 8.7, 1.1 Hz), 7.80 (ddd, 2H, *J* = 8.7, 6.6, 1.4 Hz), 7.55 (ddd, 2H, *J* = 8.7, 6.6, 1.2 Hz), 1.64 (s, 9H); ^13^C{^1^H} NMR (CDCl_3_, 100 MHz) *δ* 172.2 (C), 160.6 (C), 148.7 (C), 134.3 (C), 130.2 (CH), 129.8 (CH), 126.6 (CH), 125.8 (CH), 124.8 (C), 87.3 (CH), 85.7 (C), 28.4 (CH_3_); HRMS (ESI) *m/z* [M + H]^+^ calcd for C_20_H_19_N_2_O_2_^+^ 319.1441, found 319.1438.
*5-(tert-Butoxy)-3-(9-phenylacridin-2-yl)isoxazole* (**7l**). Compound **7l** was prepared according to the published procedure [56] from chloroisoxazole **7j** (320 mg, 0.9 mmol) and *t*BuOK (152 mg, 1.35 mmol) in THF (10 mL) to give a pure product of 318 mg (90% yield), after column chromatography on silica (light petroleum/ethyl acetate, 10:1, (*v*/*v*)) as a light yellow solid: mp 114–115 °C (light petroleum/ethyl acetate); ^1^H NMR (400 MHz, CDCl_3_) *δ* 8.34 (d, *J* = 9.1 Hz, 1H), 8.29 (d, 1H, *J* = 8.6 Hz), 8.21 (dd, 1H, *J* = 9.1, 1.9 Hz), 8.04 (dd, 1H, *J* = 1.9, 0.7 Hz), 7.79 (ddd, 1H, *J* = 8.7, 6.5, 1.4 Hz), 7.71 (ddd, 1H, *J* = 8.8, 1.4, 0.7 Hz), 7.66–7.60 (m, 3H), 7.48–7.43 (m, 3H), 5.58 (s, 1H), 1.52 (s, 9H); ^13^C{^1^H} NMR (CDCl_3_, 100 MHz) *δ* 172.2 (C), 163.4 (C), 149.2 (C), 149.0 (C), 148.0 (C), 135.4 (C), 130.5 (CH), 130.4 (CH), 129.7 (CH), 128.63 (CH), 128.57 (CH), 127.7 (CH), 127.0 (C), 126.9 (CH), 126.0 (CH), 125.5 (C), 125.0 (CH), 124.7 (C), 85.3 (C), 82.6 (CH), 28.4 (CH_3_); HRMS (ESI) *m/z* [M + H]^+^ calcd for C_26_H_23_N_2_O_2_^+^ 395.1754, found 395.1753.
*3-(2-Methylacridin-9-yl)-4,5-dihydroisoxazole-5-carbonitrile* (**10**). Compound **10** was prepared according to the general procedure GP-B from chlorooxime **2b-HCl** (150 mg, 0.49 mmol) and acrylonitrile **9** (518 mg, 9.8 mmol, 20 eq.) in DCM (20 mL) and sat. NaHCO_3_ aq. (10 mL) to give a pure product of 116 mg (83% yield), after column chromatography on silica (light petroleum/ethyl acetate, 6:1–0:1, (*v*/*v*)) as a bright yellow solid: mp 217–218 °C (ethyl acetate); ^1^H NMR (CDCl_3_, 400 MHz) *δ* 8.28 (d, 1H, *J* = 8.8 Hz), 8.21–8.19 (m, 1H), 7.92 (d, 1H, *J* = 8.8 Hz), 7.82 (ddd, 1H, *J* = 8.4, 6.7, 1.4 Hz), 7.70–7.67 (m, 2H), 7.64 (dd, 1H, *J* = 8.2, 6.7, 1.2 Hz), 5.65 (dd, 1H, *J* = 10.7, 4.8 Hz), 3.93 (dd, 1H, *J* = 17.6, 10.7 Hz), 3.81 (dd, 1H, *J* = 17.6, 4.8 Hz), 2.61 (s, 3H); ^13^C{^1^H} NMR (CDCl_3_, 100 MHz) *δ* 154.6 (C), 147.8 (C), 147.5 (C), 138.0 (C), 133.6 (CH), 130.2 (CH), 130.1 (CH), 129.9 (CH), 129.5 (C), 127.6 (CH), 124.2 (C), 124.2 (C), 124.0 (CH), 122.1 (CH), 117.0 (C), 66.8 (CH), 46.2 (CH_2_), 22.2 (CH_3_); HRMS (ESI) *m/z* [M + H]^+^ calcd for C_18_H_14_N_3_O^+^ 288.1131, found 288.1125.
*tert-Butyl 3-(9-phenylacridin-2-yl)-2H-azirine-2-carboxylate* (**11a**). A mixture of isoxazole **7l** (123 mg, 0.31 mmol) and FeCl_2_·4H_2_O (6.2 mg, 0.03 mmol) was stirred in MeCN (10 mL) at rt overnight. After the evaporation of the solvent, the product was filtered through a pad of silica (light petroleum/ethyl acetate, 10:1, (*v*/*v*)) to give pure product **11a** at 107 mg (87% yield) as a yellow solid: mp 155–156 °C (light petroleum/ethyl acetate); ^1^H NMR (400 MHz, CDCl_3_) *δ* 8.42 (d, 1H, *J* = 8.9 Hz), 8.31 (d, 1H, *J* = 8.7 Hz), 8.21–8.17 (m, 2H), 7.89–7.85 (m,1H), 7.77–7.75 (m, 1H), 7.67–7.58 (m, 3H), 7.57–7.46 (m, 2H), 7.44–7.41 (m, 1H), 2.77 (s, 1H), 1.43 (s, 9H); ^13^C{^1^H} NMR (CDCl_3_, 100 MHz) *δ* 170.7 (C), 159.0 (C), 150.4 (C), 149.7 (C), 149.6 (C), 134.8 (C), 132.6 (CH), 131.6 (CH), 131.4 (CH), 130.4 (CH), 130.3 (CH), 129.9 (CH), 129.0 (CH), 128.8 (CH), 128.6 (CH), 128.1 (CH), 127.2 (CH), 126.6 (CH), 125.6 (C), 124.5 (C), 119.8 (C), 81.7 (C), 30.9 (CH), 28.0 (CH_3_); HRMS (ESI) *m/z* [M + H]^+^ calcd for C_26_H_23_N_2_O_2_^+^ 395.1754, found 395.1753.
*9-Phenyl-2-vinylacridine* (**26**). Compound **26** was prepared according to the published procedure [57] from aldehyde **5e** (400 mg, 1.4 mmol), methyltriphenylphosphonium bromide (1.51 g, 4.2 mmol) and *t*BuOK (475 mg, 4.2 mmol) in THF (25 mL) at rt for 48 h to give a pure product pf 397 mg (81% yield), after column chromatography on silica (light petroleum/ethyl acetate, 10:1, (*v*/*v*)) as a bright yellow solid: mp 108–109 °C (light petroleum/ethyl acetate); ^1^H NMR (400 MHz, CDCl_3_) *δ* 8.24 (dd, 2H, *J* = 12.2, 8.9 Hz), 7.98 (dd, 1H, *J* = 9.2, 2.0 Hz), 7.75 (ddd, 1H, *J* = 8.5, 6.6, 11.5 Hz), 7.67 (dd, 1H, *J* = 8.8, 1.3 Hz), 7.65–7.58 (m, 3H), 7.52 (d, 1H, *J* = 2.0 Hz), 7.46–7.40 (m, 3H), 6.78 (dd, 1H, *J* = 17.6, 10.9 Hz), 5.83 (d, 1H, *J* = 17.6 Hz), 5.32 (d, 1H, *J* = 10.9 Hz); ^13^C{^1^H} NMR (CDCl_3_, 100 MHz) *δ* 148.8 (C), 148.6 (C), 147.0 (C), 136.5 (CH), 135.8 (C), 134.6 (C), 130.5 (CH), 129.94 (CH), 129.89 (CH), 129.6 (CH), 128.5 (CH), 128.4 (CH), 127.1 (CH), 126.8 (CH), 125.7 (CH), 125.5 (C), 125.3 (CH), 125.2 (C), 115.1 (CH_2_); HRMS (ESI) *m/z* [M + H]^+^ calcd for C_21_H_16_N^+^ 282.1277, found 282.1290.
*2-(1,2-Dibromoethyl)-9-phenylacridine* (**27**). Compound **27** was prepared according to the published procedure [58] from styrene **26** (320 mg, 1.14 mmol) and Br_2_ (0.7 mL, 1.4 mmol0) in CHCl_3_ (5 mL) at 0 °C for 30 min to give a pure product of 387 mg (77% yield), after column chromatography on silica (light petroleum/ethyl acetate, 10:1, (*v*/*v*)) as a bright yellow solid: mp 154–155 °C (light petroleum/ethyl acetate); ^1^H NMR (400 MHz, CDCl_3_) *δ* 8.34 (d, 1H, *J* = 9.1 Hz), 8.29 (d, 1H, *J* = 8.8 Hz), 7.84–7.79 (m, 2H), 7.71 (d, 1H, *J* = 8.8 Hz), 7.72–7.60 (m, 4H), 7.47–7.43 (m, 3H), 5.22 (dd, 1H, *J* = 10.6, 5.5 Hz), 4.11–4.01 (m, 2H); ^13^C{^1^H} NMR (CDCl_3_, 100 MHz) *δ* 149.0 (br. s, C), 148.3 (br. s, C), 135.3 (C), 130.9 (br. s, C), 130.6 (br. s, CH), 130.5 (CH), 130.4 (CH), 129.4 (br. s, CH), 128.7 (CH), 128.6 (C), 128.6 (CH), 128.3 (br. s, CH), 126.9 (CH), 126.3 (CH), 126.1 (CH), 125.5 (C), 124.3 (C), 51.1 (CH), 34.3 (CH_2_)*;* HRMS (ESI) *m/z* [M + H]^+^ calcd for C_21_H_16_Br_2_N^+^439.9644, found 439.9648.
*2-(2H-Azirin-3-yl)-9-phenylacridine* (**28**). Compound **28** was prepared according to the published procedure [59] from dibromide **27** (380 mg, 0.86 mmol), NaN_3_ (84 mg, 1.3 mmol), and NaOH (40 mg, 1 mmol) in DMSO (2 mL) and toluene (5 mL) to give a pure product of 102 mg (40% yield), after column chromatography on silica (light petroleum/ethyl acetate, 10:1, (*v*/*v*)) as a yellow-brown solid: mp 192–193 °C (light petroleum/ethyl acetate); ^1^H NMR (400 MHz, CDCl_3_) *δ* 8.41 (d, 1H, *J* = 9.0 Hz), 8.32 (d, 1H, *J* = 8.8 Hz), 8.26 (dd, 1H, *J* = 8.9, 1.8 Hz), 8.20 (d, 1H, *J* = 1.6 Hz), 7.86 (ddd, 1H, *J* = 8.5, 6.5, 1.4 Hz), 7.75 (d, 1H, *J* = 8.6 Hz), 7.68–7.62 (m, 3H), 7.52–7.47 (m, 3H), 1.82 (s, 2H); ^13^C{^1^H} NMR (CDCl_3_, 100 MHz) *δ* 165.7 (C), 150.0 (C), 149.7 (C), 149.4 (C), 135.0 (C), 131.6 (CH), 131.3 (CH), 131.0 (CH), 130.4 (CH), 129.7 (CH), 128.9 (CH), 128.7 (CH), 127.9 (CH), 127.2 (CH), 126.5 (CH), 125.6 (C), 124.6 (C), 122.8 (C), 20.5 (CH_2_); HRMS (ESI) *m/z* [M + H]^+^ calcd for C_21_H_15_N_2_^+^ 295.1230, found 295.1238.
*9-(5-(3-Phenyl-2H-azirin-2-yl)-1H-1,2,3-triazol-1-yl)acridine* (**32**). Compound **32** was prepared according to the published procedure [55] from phosphonium salt **29** (250 mg, 0.5 mmol) and azide **31** (165 mg, 0.75 mmol) in benzene (10 mL) for 4 h to give a pure product of 139 mg (77% yield), after column chromatography on silica (light petroleum/ethyl acetate, 8:1–3:1, (*v*/*v*)) as a beige solid: mp 194–196 °C (dec., light petroleum/ethyl acetate); ^1^H NMR (400 MHz, CDCl_3_) *δ* 8.30 (d, 1H, *J* = 8.8 Hz), 8.23 (d, 1H, *J* = 8.8 Hz), 7.89–7.85 (m, 1H), 7.84–7.80 (m, 1H), 7.82 (s, 1H), 7.66–7.58 (m, 2H), 7.51–7.47 (m, 1H), 7.38 (d, 2H, *J* = 9.2 Hz), 7.32–7.28 (m, 4H), 2.86 (s, 1H); ^13^C{^1^H} NMR (CDCl_3_, 100 MHz) *δ* 161.4 (C), 149.2 (C), 149.1 (C), 141.4 (C), 136.1 (C), 133.7 (CH), 132.3 (CH), 130.8 (CH), 130.7 (CH), 129.76 (CH), 129.74 (CH), 129.1 (CH), 129.0 (CH), 128.4 (CH), 128.2 (CH), 123.2 (C), 123.2 (C), 122.6 (CH), 122.4 (CH), 122.0 (C), 23.1 (CH); HRMS (ESI) *m/z* [M + H]^+^ calcd for C_23_H_16_N_5_^+^ 362.1400, found 362.1399.
*3-(9-Benzyl-9,10-dihydroacridin-9-yl)-5-phenylisoxazole* (**33a**). Compound **33a** was prepared according to the general procedure GP-C from isoxazole **7a** (34 mg, 0.105 mmol) in toluene (4 mL) at 380 nm for 50 min to give a pure product of 33 mg (75% yield), after column chromatography on silica (light petroleum/ethyl acetate, 15:1, (*v*/*v*)) as a colorles solid: mp 143–144 °C (light petroleum/ethyl acetate); ^1^H NMR (400 MHz, CDCl_3_) *δ* 7.76–7.73 (m, 2H), 7.467.40 (m, 3H), 7.12–7.04 (m, 5H), 6.94–6.90 (m, 1H), 6.88–6.84 (m, 1H), 6.45–6.43 (m, 2H), 6.32–6.30 (m, 2H), 6.23 (s, 1H), 5.65 (s, 1H), 3.60 (s, 2H); ^13^C{^1^H} NMR (CDCl_3_, 100 MHz) *δ* 170.7 (C), 169.4 (C), 138.5 (C), 137.0 (C), 130.7 (CH), 129.9 (CH), 129.3 (CH), 128.8 (CH), 128.0 (CH), 127.5 (C), 126.9 (CH), 125.83 (CH), 125.77 (CH), 122.0 (C), 120.4 (CH), 113.2 (CH), 101.0 (CH), 50.6 (CH_2_), 48.2 (C); HRMS (ESI) *m/z* [M + H]^+^ calcd for C_9_H_23_N_2_O^+^ 415.1805, found 415.1810.
*3-(9-(3,5-Dimethylbenzyl)-9,10-dihydroacridin-9-yl)-5-phenylisoxazole* (**33b**). Compound **33b** was prepared according to the general procedure GP-C from isoxazole **7a** (52 mg, 0.16 mmol) in mesitylene (10 mL) at 380 nm for 90 min to give a pure product of 48 mg (67% yield), after column chromatography on silica (light petroleum/ethyl acetate, 15:1, (*v*/*v*)) as a light yellow solid: mp 187–188 °C (light petroleum/ethyl acetate); ^1^H NMR (400 MHz, CDCl_3_) *δ* 7.73 (dd, 2H, *J* = 7.7, 2.0 Hz), 7.44–7.38 (m, 3H), 7.10–7.06 (m, 4H), 6.84 (t, 2H, *J* = 7.5 Hz), 6.68 (br. s, 1H), 6.41 (dd, 2H, *J* = 8.3, 1.2 Hz), 6.23 (s, 1H), 5.86 (s, 2H), 5.64 (br. s, 1H), 3.47 (s, 2H), 1.98 (s, 6H); ^13^C{^1^H} NMR (CDCl_3_, 100 MHz) *δ* 170.6 (C), 169.3 (C), 138.7 (C), 136.7 (C), 136.0 (C), 129.9 (CH), 129.3 (CH), 128.8 (CH), 128.6 (CH), 127.8 (CH), 127.6 (C), 127.2 (CH), 125.8 (CH), 122.2 (C), 120.3 (CH), 112.9 (CH), 101.1 (CH), 50.7 (CH_2_), 48.3 (C), 21.0 (CH_3_); HRMS (ESI) *m/z* [M + H]^+^ calcd for C_31_H_27_N_2_O^+^ 443.2118, found 443.2124.
*3-(9-(4-Chlorobenzyl)-9,10-dihydroacridin-9-yl)-5-phenylisoxazole* (**33c**). Compound **33c** was prepared according to the general procedure GP-C from isoxazole **7a** (60 mg, 0.19 mmol) and 4-chlorotoluene (2.36 g, 18.6 mmol, 100 eq.) in α,α,α-trifluorotoluene (12 mL) at 380 nm for 40 h to give a pure product of 27 mg (32% yield), after column chromatography on silica (light petroleum/ethyl acetate, 25:1, (*v*/*v*)) as a light yellow solid: mp 183–184 °C (light petroleum/ethyl acetate); ^1^H NMR (400 MHz, C_6_D_6_) *δ* 7.36–7.34 (m, 2H), 7.10–7.09 (m, 2H), 6.97–6.92 (m, 5H), 6.84–6.81 (m, 2H), 6.770–6.66 (m, 2H), 6.21–6.19 (m, 2H), 6.03–6.00 (m, 2H), 5.97 (s, 1H), 4.92 (br. s, 1H), 3.71 (s, 2H); ^13^C{^1^H} NMR (C_6_D_6_, 100 MHz) *δ* 170.8 (C), 169.9 (C), 138.9 (C), 136.3 (C), 132.5 (CH), 132.4 (C), 129.9 (CH), 129.7 (CH), 128.9 (CH), 128.4 (CH), 128.2 (CH), 127.5 (CH), 126.1 (CH), 122.4 (C), 120.9 (CH), 113.6 (CH), 101.4 (CH), 50.6 (CH_2_), 48.6 (C); HRMS (ESI) *m/z* [M + Na]^+^ calcd for C_29_H_21_ClN_2_NaO^+^ 471.1235, found 471.1236.
*3-(9-(tert-Butoxymethyl)-9,10-dihydroacridin-9-yl)-5-phenylisoxazole* (**33d**). Compound **33d** was prepared according to the general procedure GP-C from isoxazole **7a** (60 mg, 0.19 mmol) in MTBE (7 mL) at 380 nm for 2.5 h to give a pure product of 56 mg (73% yield), after column chromatography on silica (light petroleum/ethyl acetate, 20:1, (*v*/*v*)) as a colorless solid: mp 180–181 °C (light petroleum/ethyl acetate); ^1^H NMR (400 MHz, C_6_D_6_): *δ* 7.36–7.33 (m, 2H), 7.24–7.22 (m, 2H), 7.05–7.01 (m, 2H), 6.96–6.93 (m, 3H), 6.77–6.73 (m, 2H), 6.37–6.34 (m, 2H), 6.03 (s,1H), 5.62–5.60 (br. s, 1H), 4.32 (s, 2H), 0.87 (s, 9H); ^13^C NMR (100 MHz, C_6_D_6_): *δ* 169.5 (C), 169.2 (C), 139.4 (C), 130.0 (CH), 129.7 (CH), 128.9 (CH), 128.4 (CH), 128.0 (CH), 126.1 (CH), 122.7 (C), 120.6 (CH), 113.6 (CH), 101.4 (CH), 73.0 (C), 71.2 (CH_2_), 48.4 (C), 27.3 (CH_3_); HRMS (ESI) *m/z* [M + H]^+^ calcd for C_27_H_27_N_2_O_2_^+^ 411.2064, found 411.2067.
*3-(9-(1,4-Dioxan-2-yl)-9,10-dihydroacridin-9-yl)-5-phenylisoxazole* (**33e**). Compound **33e** was prepared according to the general procedure C from isoxazole **7a** (50 mg, 0.16 mmol) in 1,4-dioxane (5 mL) at 405 nm for 90 min to give a pure product pf 52 mg (66% yield), after column chromatography on silica (light petroleum/ethyl acetate, 20:1, (*v*/*v*)) as a light brown semi-solid; NMR spectra indicate that the product is a mixture of two diastereomers in a ~1:1 ratio; ^1^H NMR (400 MHz, CDCl_3_): *δ* 7.72–7.68 (m, 2H), 7.42–7.36 (m, 3H), 7.22–7.18 (m, 1H), 7.14–7.11 (m, 2H), 7.04–7.02 (m, 1H), 6.87–6.71 (m, 4H), 6.27 (br. S, 1H), 6.12 (s, 1H), 4.46–4.43 (m, 1H), 3.84–3.79 (m, 2H), 3.64–3.54 (m, 2H), 3.37–3.31 (m, 1H), 3.24–3.19 (m, 1H); ^13^C NMR (100 MHz, CDCl_3_): *δ* 168.9 ©, 168.3 (br. s, C), 138.4 (br. s, C), 138.3 (C), 132.2 (br. s, CH), 129.9 (CH), 129.6 (br. s, CH), 128.8 (CH), 128.5 (br. s, CH), 128.3 (br. s, CH), 127.5 (C), 125.7 (CH), 120.7 (br. s, CH), 120.1 (br. s, CH), 119.6 (br. s, C), 119.1 (br. s, C), 113.7 (br. s, CH), 113.3 (br. s, CH), 101.2 (CH), 81.2 (CH), 67.7 (CH_2_), 67.3 (CH_2_), 66.2 (CH_2_), 48.7 (br. s, C); HRMS (ESI) *m/z* [M + H]^+^ calcd for C_26_H_23_N_2_O_3_+ 411.1703, found 411.1700.
*3-(9-(2-Methylbenzyl)-9,10-dihydroacridin-9-yl)-5-(trimethylsilyl)isoxazole* (**33f**). Compound **33f** was prepared according to the general procedure GP-C from isoxazole **7b** (57 mg, 0.18 mmol) in *o*-xylene (8 mL) at 380 nm for 60 min to give a pure product of 61 mg (80% yield), after column chromatography on silica (light petroleum/ethyl acetate, 10:1, (*v*/*v*)) as a light yellow solid: mp 187–188 °C (light petroleum/ethyl acetate); ^1^H NMR (400 MHz, CDCl_3_) *δ* 7.09 (td, 2H, *J* = 7.7, 1.5 Hz), 6.96–6.92 (m, 3H), 6.85–6.81 (m, 3H), 6.67 (td, 1H, *J* = 7.5, 1.5 Hz), 6.42 (d, 2H, *J* = 7.9 Hz), 6.12 (s, 1H), 6.08 (dd, 1H, *J* = 7.7, 1.4 Hz), 5.65 (br. s, 1H), 3.62 (s, 2H), 1.48 (s, 3H), 0.32 (s, 9H); ^13^C NMR (100 MHz, CDCl_3_): *δ* 177.5 (C), 168.3 (C), 139.0 (C), 138.0 (C), 135.3 (C), 131.7 (CH), 129.6 (CH), 129.4 (CH), 127.8 (CH), 125.9 (CH), 124.2 (CH), 122.6 (C), 120.4 (CH), 114.1 (CH), 113.2 (CH), 48.1 (C), 47.2 (CH_2_), 18.8 (CH_3_), −1.9 (CH_3_); HRMS (ESI) *m/z* [M + Na]^+^ calcd for C_27_H_28_N_2_NaOSi^+^ 447.1863, found 447.1868.
*3-(9-(Tetrahydrofuran-2-yl)-9,10-dihydroacridin-9-yl)-5-(trimethylsilyl)isoxazole* (**33g**). Compound **33g** was prepared according to the general procedure GP-C from isoxazole **7b** (100 mg, 0.31 mmol) in THF (10 mL) at 405 nm for 3 h to give a pure product of 72 mg (59% yield), after column chromatography on silica (light petroleum/ethyl acetate, 20:1, (*v*/*v*)) as a colorless solid: mp 79–80 °C (light petroleum/ethyl acetate); NMR spectra indicate that the product is a mixture of two diastereomers in a ~1:1 ratio; ^1^H NMR (400 MHz, CDCl_3_): *δ* 7.16–7.05 (m, 3H), 6.95–6.92 (m, 1H), 6.82–6.75 (m, 2H), 6.2–6.68 (m, 2H), 6.23 (br. s, 1H), 6.05 (s, 1H), 4.86 (t, 1H, *J* = 7.2 Hz), 3.75 (td, 1H, *J* = 7.4, 4.8 Hz), 3.36 (q, 1H, 7.4 Hz), 1.73–1.69 (m, 2H), 1.33–1.23 (m, 2H); ^13^C NMR (100 MHz, CDCl_3_): *δ* 176.9 (C), 167.0 (C), 138.8 (C), 138.2 (C), 132.1 (CH), 129.7 (CH), 128.1 (CH), 127.9 (CH), 125.1 (C), 121.2 (C), 120.2 (CH), 120.0 (CH), 119.9 (C), 115.4 (C), 114.1 (CH), 113.4 (CH), 112.9 (CH), 86.2 (CH), 69.2 (CH_2_), 49.8 (C), 27.7 (CH_2_), 25.7 (CH_2_), −1.9 (CH_3_); HRMS (ESI) *m/z* [M + Na]^+^ calcd for C_23_H_26_N_2_NaO_2_Si^+^ 413.1656, found 413.1658.
*3-(9-Benzyl-9,10-dihydroacridin-9-yl)-5-(tert-butoxy)isoxazole* (**33h**). Compound **33h** was prepared according to the general procedure GP-C from isoxazole **7k** (41 mg, 0.13 mmol) in toluene (5 mL) at 380 nm for 90 min to give a pure product of 31 mg (59% yield), after column chromatography on silica (light petroleum/ethyl acetate, 20:1, (*v*/*v*)) as a colorless solid: mp 161–162 °C (light petroleum/ethyl acetate); ^1^H NMR (400 MHz, CDCl_3_) *δ* 7.11–7.00 (m, 5H), 6.90–6.82 (m, 4H), 6.38 (dd, 2H, *J* = 7.9, 1.2 Hz), 6.26–6.23 (m, 2H), 5.58 (s, 1H), 5.00 (s, 1H), 3.45 (s, 2H), 1.43 (s, 9H); ^13^C NMR (100 MHz, C_6_D_6_) *δ* 172.0 (C), 171.8 (C), 138.9 (C), 137.8 (C), 131.3 (CH), 129.8 (CH), 128.4 (CH), 128.4 (CH), 127.9 (CH), 127.3 (CH), 126.2 (CH), 122.9 (C), 120.7 (CH), 113.5 (CH), 85.7 (CH), 83.7 (C), 50.7 (CH_2_), 49.2 (C), 27.9 (CH_3_); HRMS (ESI) *m/z* [M + H]^+^ calcd for C_27_H_27_N_2_O_2_^+^ 411.2067, found 411.2070.
*3-(9-(3,5-Dimethylbenzyl)-9,10-dihydroacridin-9-yl)-5-((4-isopropylphenoxy)methyl)isoxazole* (**33i**). Compound **33i** was prepared according to the general procedure GP-C from isoxazole **7k** (55 mg, 0.14 mmol) in mesitylene (10 mL) at 380 nm for 90 min to give a pure product of 53 mg (74% yield), after column chromatography on silica (light petroleum/ethyl acetate, 20:1, (*v*/*v*)) as a colorless solid: mp 167–168 °C (light petroleum/ethyl acetate); ^1^H NMR (400 MHz, CDCl_3_) *δ* 7.15–7.12 (m, 2H), 7.08–7.04 (m, 2H), 6.98 (d, 2H, *J* = 7.6 Hz), 6.87–6.81 (m, 4H), 6.67 (s, 1H), 6.40–6.38 (m, 2H), 6.04 (s, 1H), 5.83 (s, 2H), 5.60 (s, 1H), 5.08 (s, 2H), 3.42 (s, 2H), 2.85 (hept, 1H, *J* = 6.9 Hz), 1.97 (s, 6H), 1.22 (d, 6H, *J* = 6.9 Hz); ^13^C NMR (100 MHz, CDCl_3_) *δ* 169.9 (C), 167.6 (C), 156.1 (C), 142.3 (C), 138.7 (C), 136.7 (C), 136.0 (C), 129.2 (CH), 128.7 (CH), 127.8 (CH), 127.4 (CH), 127.2 (CH), 122.2 (C), 120.3 (CH), 114.9 (CH), 113.0 (CH), 104.8 (CH), 61.9 (CH_2_), 50.7 (CH_2_), 48.3 (C), 33.3 (CH), 24.1 (CH_3_), 20.9 (CH_3_); HRMS (ESI) *m/z* [M + H]^+^ calcd for C_35_H_35_N_2_O_2_^+^ 515.2693, found 515.2692.
*Methyl 3-(9-(1,4-dioxan-2-yl)-2-methyl-9,10-dihydroacridin-9-yl)isoxazole-5-carboxylate* (**33j**). Compound **33j** was prepared according to the general procedure GP-C from isoxazole **7d** (90 mg, 0.28 mmol) in 1,4-dioxane (20 mL) at 380 nm for 2 h to givea pure product of 65 mg (57% yield), after column chromatography on silica (light petroleum/MTBE, 3:1, (*v*/*v*)) as a light yellow solid: mp 79–81 °C (light petroleum/MTBE); NMR spectra indicate that the product is a mixture of two diastereomers in a ~1:1 ratio; ^1^H NMR (400 MHz, C_6_D_6_) *δ* 7.31–7.29 (m, 0.5 H), 7.05–7.00 (m, 0.5 H), 6.99–6.96 (m, 1H), 6.89–6.86 (m, 0.5 H), 6.78–6.74 (m, 1H), 6.64–6.59 (m, 0.5 H), 6.49 (s, 0.5 H), 6.48 (s, 0.5 H), 6.30–6.24 (m, 1H), 6.21–6.14 (m, 1H), 5.44 (s, 0.5 H), 5.43 (s, 0.5 H), 4.78 (dd, 0.5 H, *J* = 10.2, 2.3 Hz), 4.72 (dd, 0.5 H, *J* = 10.2, 2.2 Hz), 3.91–3.86 (m, 1H), 3.70–3.60 (m, 1H), 3.44–3.37 (m, 2H), 3.14 (s, 1.5 H), 3.14 (s, 1.5 H), 3.16–3.10 (m, 1H), 3.06–3.00 (m, 1H), 2.04 (s, 1.5 H), 1.92 (s, 1.5 H); ^13^C NMR (100 MHz, C_6_D_6_) *δ* 168.99 (C), 168.96 (C), 160.1 (C), 157.04 (C), 157.03 (C), 139.1 (C), 136.7 (C), 136.5 (C), 132.8 (CH), 132.5 (CH), 130.3 (C), 129.78 (CH), 129.75 (CH), 129.6 (CH), 129.5 (CH), 128.66 (CH), 128.62 (CH), 128.4 (CH), 120.8 (CH), 120.3 (CH), 119.6 (C), 119.5 (C), 119.3 (C), 119.1 (C), 114.3 (CH), 114.1 (CH), 113.9 (CH), 113.7 (CH), 111.48 (CH), 111.46 (CH), 82.1 (CH), 81.9 (CH), 67.9 (CH_2_), 67.72 (CH_2_), 67.66 (CH_2_), 66.16 (CH_2_), 66.12 (CH_2_), 51.8 (CH_3_), 49.33 (C), 49.25 (C), 30.2 (C), 20.9 (CH_3_), 20.7 (CH_3_); HRMS (ESI) *m/z* [M + H]^+^ calcd for C_23_H_25_N_2_O_5_^+^ 407.1601, found 407.1601.
*3-(9-(tert-Butoxymethyl)-2-methyl-9,10-dihydroacridin-9-yl)-4,5-dihydroisoxazole-5-carbonitrile* (**33k**). Compound **33k** was prepared according to the general procedure GP-C from isoxazole **10** (60 mg, 0.18 mmol) in MTBE (20 mL) at 380 nm for 2 h to give a pure product of 47 mg (69% yield), after column chromatography on silica (light petroleum/MTBE, 1:1, (*v*/*v*)) as a light brow oil; NMR spectra indicate that the product is a mixture of two diastereomers in a ~1:1 ratio; ^1^H NMR (400 MHz, C_6_D_6_) *δ* 7.23–7.19 (m, 1 H), 7.03–6.91 (m, 2 H), 6.84–6.78 (m, 1.5 H), 6.73–6.69 (m, 0.5 H), 6.26–6.19 (m, 2H), 5.38 (s, 0.5 H), 5.37 (s, 0.5 H), 4.07–3.94 (m, 3H), 2.51–2.27 (m, 2H), 2.21 (s, 1.5 H), 2.08 (s, 1.5 H), 0.77 (s, 4.5 H), 0.77 (s, 4.5 H); ^13^C NMR (100 MHz, C_6_D_6_) *δ* 161.1 (C), 160.9 (C), 139.9 (C), 139.4 (C), 137.3 (C), 136.9 (C), 129.6 (C), 129.4 (CH), 129.3 (CH), 128.63 (CH), 128.56 (CH), 128.45 (CH), 128.42 (CH), 128.39 (CH), 121.1 (CH), 120.3 (CH), 120.0 (C), 119.8 (C), 119.2 (C), 119.1 (C), 117.9 (C), 117.7 (C), 114.1 (CH), 114.0 (CH), 113.94 (CH), 113.90 (CH), 73.0, 71.68 (CH_2_), 71.63 (CH_2_), 65.9 (CH), 65.8 (CH), 48.6, 48.5, 42.0 (CH_2_), 27.1 (CH_3_); HRMS (ESI) *m/z* [M + H]^+^ calcd for C_23_H_26_N_3_O_2_^+^ 376.2020, found 376.2013.
*4-((3-(9-Benzyl-2-methyl-9,10-dihydroacridin-9-yl)isoxazol-5-yl)methyl)-5-methoxy-3-(naphthalen-2-yl)isoxazole* (**33l**). Compound **33l** was prepared according to the general procedure GP-C from isoxazole **7e** (50 mg, 0.10 mmol) in toluene (10 mL) at 380 nm for 5h to give a pure product of 37 mg (62% yield), after column chromatography on silica (light petroleum/ethyl acetate, 20:1, (*v*/*v*)) as a colorless solid: mp 157–158 °C (light petroleum/ethyl acetate); ^1^H NMR (400 MHz, CDCl_3_) *δ* 7.91 (s, 1H), 7.86–7.84 (m, 2H), 7.71 (d, 1H, *J* = 8.0 Hz), 7.61 (d, 1H, *J* = 8.6 Hz), 7.54 (t, 1H, *J* = 7.3 Hz), 7.49 (t, 1H, *J* = 7.4 Hz), 7.02 (d, 1H, *J* = 7.8 Hz), 6.98 (d, 1H, *J* = 8.4 Hz), 6.89–6.78 (m, 5H), 6.622 (t, 1H, *J* = 7.7 Hz), 6.34 (d, 1H, *J* = 7.9 Hz), 6.29 (d, 1H, *J* = 8.0 Hz), 6.24 (d, 2H, *J* = 7.6 Hz), 5.65 (s, 1H), 5.50 (s, 1H), 4.12 (s, 3H), 3.85 (s, 2H), 3.51–3.43 (m, 2H), 2.13 (s, 3H); ^1^H NMR (400 MHz, C_6_D_6_) *δ* 8.03 (s, 1H), 7.83–7.81 (m, 1H), 7.58–7.56 (m, 2H), 7.53–7.51 (m, 1H), 7.28–7.20 (m, 2H), 7.03–6.98 (m, 2H), 6.92–6.84 (m, 4H), 6.75–6.73 (m, 1H), 6.57–6.53 (m, 1H), 6.50–6.47 (m, 2H), 5.98–5.94 (m, 2H), 5.55 (s, 1H), 4.87 (s, 1H), 4.87 (s, 1H), 3.89–3.79 (m, 2H), 3.48 (s, 2H), 3.33 (s, 3H), 1.93 (s, 3H); ^13^C NMR (100 MHz, C_6_D_6_) *δ* 170.7 ©, 170.6 (C), 170.4 (C), 164.5 (C), 139.0 (C), 137.8 (C), 136.6 (C), 134.2 (C), 133.6 (C), 131.2 (CH), 129.8 (CH), 129.68 (CH), 129.66 (C), 129.02 (CH), 128.9 (CH), 128.8 (CH), 128.4 (CH), 128.2 (CH), 128.0 (CH), 127.9 (CH), 127.8 (C), 127.3 (CH), 127.1 (CH), 126.7 (CH), 126.3 (CH), 125.5 (CH), 122.5 (C), 120.3 (CH), 113.6 (CH), 113.4 (C), 103.6 (CH), 86.8 (C), 57.4 (CH_3_), 51.2 (CH_2_), 48.7 (C), 20.7 (CH_3_), 20.1 (CH_2_); HRMS (ESI) *m/z* [M + H]^+^ calcd for C_39_H_32_N_3_O_3_^+^ 590.2438, found 590.2424.
*4-((3-(9-(3,5-Dimethylbenzyl)-2-methyl-9,10-dihydroacridin-9-yl)isoxazol-5-yl)methyl)-5-methoxy-3-(naphthalen-2-yl)isoxazole* (**33m**). Compound **33m** was prepared according to the general procedure GP-C from isoxazole **7e** (50 mg, 0.10 mmol) in mesytilene (10 mL) at 380 nm for 5h to give a pure product of 43 mg (70% yield), after column chromatography on silica (light petroleum/ethyl acetate, 20:1, (*v*/*v*)) as a beige solid: mp 162–163 °C (light petroleum/ethyl acetate); ^1^H NMR (400 MHz, C_6_D_6_): *δ* 8.03 (s, 1H), 7.84–7.81 (m, 1H), 7.58–7.56 (m, 2H), 7.54–7.51 (m, 1H), 7.27–7.20 (m, 2H), 7.03–7.00 (m, 2H), 6.92–6.88 (m, 1H), 6.77–6.74 (m, 1H), 6.61 (s, 1H), 6.57–6.53 (m, 1H), 6.10 (s, 2H), 6.01–5.97 (m, 2H), 5.58 (s, 1H), 4.91 (s, 1H), 3.84–3.75 (m, 2H), 3.49 (s, 2H), 3.33 (s, 3H), 1.99 (s, 6H), 1.94 (s, 3H); ^13^C NMR (100 MHz, C_6_D_6_) *δ* 170.7 (C), 170.5 (C), 170.4 (C), 164.5 (C), 139.2 (C), 137.4 (C), 136.8 (C), 136.0 (C), 134.2 (C), 133.6 (C), 129.8 (CH), 129.7 (CH), 129.6 (C), 129.4 (CH), 129.0 (CH), 128.9 (CH), 128.6 (CH), 128.4 (CH), 128.4 (CH), 128.2 (CH), 128.0 (CH), 127.9 (CH), 127.7 (CH), 127.1 (CH), 126.7 (CH), 125.5 (CH), 122.8 (C), 120.3 (CH), 113.3 (CH), 113.2 (CH), 103.7 (CH), 86.8 (C), 57.4 (CH_3_), 51.2 (CH_2_), 48.8 (C), 21.3 (CH_3_), 20.7 (CH_3_), 20.1 (CH_2_); HRMS (ESI) *m/z* [M + H]^+^ calcd for C_41_H_36_N_3_O_3_^+^ 618.2751, found 618.2738.
*5-(tert-Butoxy)-3-(9-(3,5-dimethylbenzyl)-9-phenyl-9,10-dihydroacridin-2-yl)isoxazole* (**33n**). Compound **33n** was prepared according to the general procedure GP-C from isoxazole **7l** (56 mg, 0.14 mmol) in mesytilene (15 mL) at 380 nm for 8h to give a pure product of 34 mg (47% yield), after column chromatography on silica (light petroleum/MTBE, 20:1, (*v*/*v*)) as a colorless solid: mp 138–139 °C (light petroleum/ethyl acetate); ^1^H NMR (400 MHz, C_6_D_6_) *δ* 7.68–7.66 (m, 1H), 7.53–7.47 (m, 3H), 7.12–7.10 (m, 2H), 7.04–7.01 (m, 1H), 6.92–6.88 (m, 1H), 6.82–6.80 (m, 1H), 6.68–6.64 (m, 2H), 6.02 (s, 2H), 5.99–5.94 (m, 2H), 5.37 (s, 1H), 4.92 (s, 1H), 3.40–3.30 (m, 2H), 2.00 (s, 6H), 1.11 (s, 9H); ^13^C NMR (100 MHz, C_6_D_6_) *δ* 172.3 (C), 163.9 (C), 151.3 (C), 140.4 (C), 138.6 (C), 137.6 (C), 136.1 (C), 130.8 (CH), 130.0 (CH), 129.1 (CH), 129.0 (CH), 128.4 (CH), 128.3 (CH), 127.8 (CH), 127.3 (C), 127.1 (C), 127.0 (CH), 126.4 (CH), 125.5 (CH), 122.4 (C), 120.8 (CH), 113.3 (CH), 113.0 (CH), 84.1 (C), 82.6 (CH), 52.5 (CH_2_), 51.7 (C), 28.2 (CH_3_), 21.3 (CH_3_); HRMS (ESI) *m/z* [M + H]^+^ calcd for C_35_H_35_N_2_O_2_^+^ 515.2693, found 515.2690.

#### 3.2.5. Cell Culture

The MCF7 breast cancer cell line, HCT 116 colorectal carcinoma cell line, A-704 kidney adenocarcinoma cell line, and WI-26 VA4 lung epithelial-like cells were purchased from the ATCC. The MCF7 cells were maintained in MEM (Gibco, Paisley, UK) supplemented with 10% fetal bovine serum (FBS, Gibco, UK), human recombinant insulin (0.01 mg/mL), penicillin (100 UI mL^−1^), streptomycin (100 µg mL^−1^), and GlutaMax (2 mM, Gibco, UK). The HCT 116 cells were maintained in McCoy’s 5A (Gibco, UK) supplemented with 10% fetal bovine serum (FBS, Gibco, UK), penicillin (100 UI mL^−1^), streptomycin (100 µg mL^−1^), and GlutaMax (1.5 mM, Gibco, UK). A-704 and WI-26 VA4 cells were maintained in MEM (Gibco, UK) supplemented with 10% fetal bovine serum (FBS, Gibco, UK), penicillin (100 UI mL^−1^), streptomycin (100 µg mL^−1^), and GlutaMax (1.87 mM, Gibco, UK). All cell lines’ cultivation was performed under a humidified atmosphere of 95% air/5% CO_2_ at 37 °C. Subconfluent monolayers, in the log growth phase, were harvested by a brief treatment with TrypLE Express solution (Gibco, UK) in phosphate-buffered saline (PBS, Capricorn Scientific, Ebsdorfergrund, Germany) and washed three times in serum-free PBS. The number of viable cells was determined by trypan blue exclusion.

#### 3.2.6. Antiproliferative Assay

The effects of the synthesized compounds on cell viability were determined using the MTT colorimetric test. All examined cells were diluted with the growth medium to 3.5 × 10^4^ cells per mL and the aliquots (7 × 10^3^ cells per 200 μL) were placed in individual wells in 96-multiplates (Eppendorf, Germany) and incubated for 24 h. The next day, the cells were then treated with synthesized compounds separately at a final concentration of 30 μM and incubated for 72 h at 37 °C in a 5% CO_2_ atmosphere. After incubation, the cells were then treated with 40 μL of MTT solution (3-(4,5-dimethylthiazol-2-yl)-2,5-diphenyltetrazolium bromide, 5 mg mL^−1^ in PBS) and incubated fir 4 h. After an additional 4h incubation, the medium with MTT was removed and DMSO (150 μL) was added to dissolve the crystals formazan. The plates were shaken for 10 min. The optical density of each well was determined at 560 nm using a microplate reader GloMax Multi+ (Promega, Madison, WI, USA). Each of the tested compounds was evaluated for cytotoxicity in three separate experiments.

## 4. Conclusions

Easy-to-handle *N*-hydroxyacridinecarbimidoyl chloride hydrochlorides were prepared from oximes in 69–94% by chlorination using Cl_2_. The cycloaddition of nitrile oxides, which were generated from them, to terminal alkynes gave 3-(acridin-9-yl)isoxazoles in a 80–98% yield. The reaction with 1,1-dichloroethene, accompanied by the dehydrochlorination of intermediate 5,5-dichloro-4,5-dihydroisoxazoles, gave acridinyl-substituted 5-chloroizoxazoles in a 62–64% yield, and the reaction with acrylonitrile gave 4,5-dihydroisoxazole-5-carbonitrile in an 83% yield. Acridinyl-containing azirines with a long-wave absorption of 320–420 nm were prepared from acridinyl isoxazoles or by traditional methods, in order to evaluate the possibility of their use in photoclick cycloaddition in the UV/visible radiation boundary region. However, their photolysis in the presence of DMAD or PMI in acetonitrile using 365, 380, 405, 425, or 450 nm LEDs, as well as white light LED (380–760 nm), did not produce the expected photolysis cycloaddition products of the nitrile ylide. A comparison of the results of DFT calculations of the cycloaddition of nitrile ylides, derived from acridinylazirines and 3-(pyren-1-yl)-2*H*-azirine, for which such cycloaddition was previously implemented, did not reveal any obstacles in terms of energy barriers to the cycloaddition of acridinyl-substituted nitrile ylides. This means that the acridine fragment, unlike the pyrene fragment, cannot be an antenna for transmitting light energy for the formation of nitrile ylides from azirines. One of the reasons for this may be the fast relaxation of the singlet excited state of acridinyl-substituted azirines through intersystem crossing into a triplet excited state, characteristic of acridine heterocyclic systems. This may prevent the energy required to break the azirine C2-C3 bond from being transferred from the excited acridine moiety to the azirine moiety and block, therefore, the formation of nitrile ylide from azirine. It was found that irradiation of the synthesized isoxazolylacridines with LED light already in the UV/visible radiation boundary region (385–405 nm) in the presence of toluene, *o*-xylene, mesitylene, 4-chlorotoluene, THF, 1,4-dioxane, and MTBE gave 9-alkylated acridane derivatives, usually in good yields. A series of synthesized acridine/acridane-substituted isoxazoles and acridine-substituted azirines were tested for their cytotoxicity. Azirines were toxic to all cell lines, including normal cells (WI-26 VA4). 5-Methoxy-4-((3-(2-methylacridin-9-yl)isoxazol-5-yl)methyl)-3-(naphthalen-2-yl)isoxazole, 3-(acridin-9-yl)-5-chloroisoxazole, and 5-(*tert*-butoxy)-3-(9-phenylacridin-2-yl)isoxazole are among the compounds that were active, but not particularly selective.

## Data Availability

Data are contained within the article.

## Supplementary Materials

The following supporting information can be downloaded at: https://www.mdpi.com/article/10.3390/molecules29071538/s1, X-ray diffraction experiment; UV-VIS absorption spectra of compounds **7**; **10**; **11a**; **28**; NMR spectra of compounds **4e**; **5**; **1**; **2**; **7**; **10**; **11a**; **26**; **27**; **28**; **32**; **33**; Computational details. References [60,61,62,63,64,65,66,67,68,69] are cited in the Supplementary Materials.
