# Supplementary material for: Acridine–Isoxazole and Acridine–Azirine Hybrids: Synthesis, Photochemical Transformations in the UV/Visible Radiation Boundary Region, and Anticancer Activity
Source: Molecules. 2024 Mar 29;29(7):1538. doi: 10.3390/molecules29071538 (PMC11013717; doi:10.3390/molecules29071538)
Supplement: Supplementary file 1 [file molecules-29-01538-s001.zip › SI.pdf]

Supporting Information for

Acridine-Isoxazole and Acridine-Azirine Hybrids. Synthesis, Photochemical Transformations in the UV/Visible Radiation Boundary Region and Anticancer Activity

Ekaterina E. Galenko[1], Mikhail S. Novikov[1], Alexander S. Bunev[2], and Alexander F. Khlebnikov[1,]*

[1] St. Petersburg State University, Institute of Chemistry, 7/9 Universitetskaya *Naberezhnaya*, St. Petersburg, 199034, Russia;

[2] Medicinal Chemistry Center, Togliatti State University, Togliatti, 445020 Russia.

*Correspondence: a.khlebnikov@spbu.ru; Tel.:+7 812-363-6000(9836) (A.F.K.)

Table of Contents

X-Ray Diffraction Experiments	S2
UV-VIS absorption spectra	S10
NMR Spectra of Compound **4e**	S11
NMR Spectra of Compounds **5**	S14
NMR Spectra of Compounds **1**	S23
NMR Spectra of Compounds **2**	S35
NMR Spectra of Compounds **7**	S50
NMR Spectra of Compound **10**	S86
NMR Spectra of Compound **11a**	S89
NMR Spectra of Compound **26**	S82
NMR Spectra of Compound **27**	S95
NMR Spectra of Compound **28**	S98
NMR Spectra of Compound **32**	S101
NMR Spectra of Compounds **33**	S104
Computational Details	S147

X-RAY DIFFRACTION EXPERIMENTS

Crystal structure of **33b** was determined by single crystal X-ray diffraction analysis. Suitable crystal was selected and fixed on micro-amounts and the diffraction data were collected on diffractometer. The crystal **33b** was measured at temperature 100 K, using monochromated CuKα radiation. The unit cell parameters and refinement characteristics of the crystal structure of **33b** is given below. Using Olex2[1], the structure was solved with the ShelXT [2] structure solution program using Intrinsic Phasing and refined with the ShelXL [3] refinement package using Least Squares minimization.

References

1. Dolomanov, O. V.; Bourhis, L. J.; Gildea, R. J.; Howard, J .A. K.; Puschmann, H. J. Appl. Cryst. 2009, 42, 339.

2. Sheldrick, G. M. Acta Cryst. 2015, A71, 3.

3. Sheldrick, G. M. Acta Cryst. 2015, C71, 3.

3-(9-(3,5-Dimethylbenzyl)-9,10-dihydroacridin-9-yl)-5-phenylisoxazole 33b

Single crystals of **33b** were obtained by slow recrystallization from acetonitrile at room temperature (CCDC 2328401)

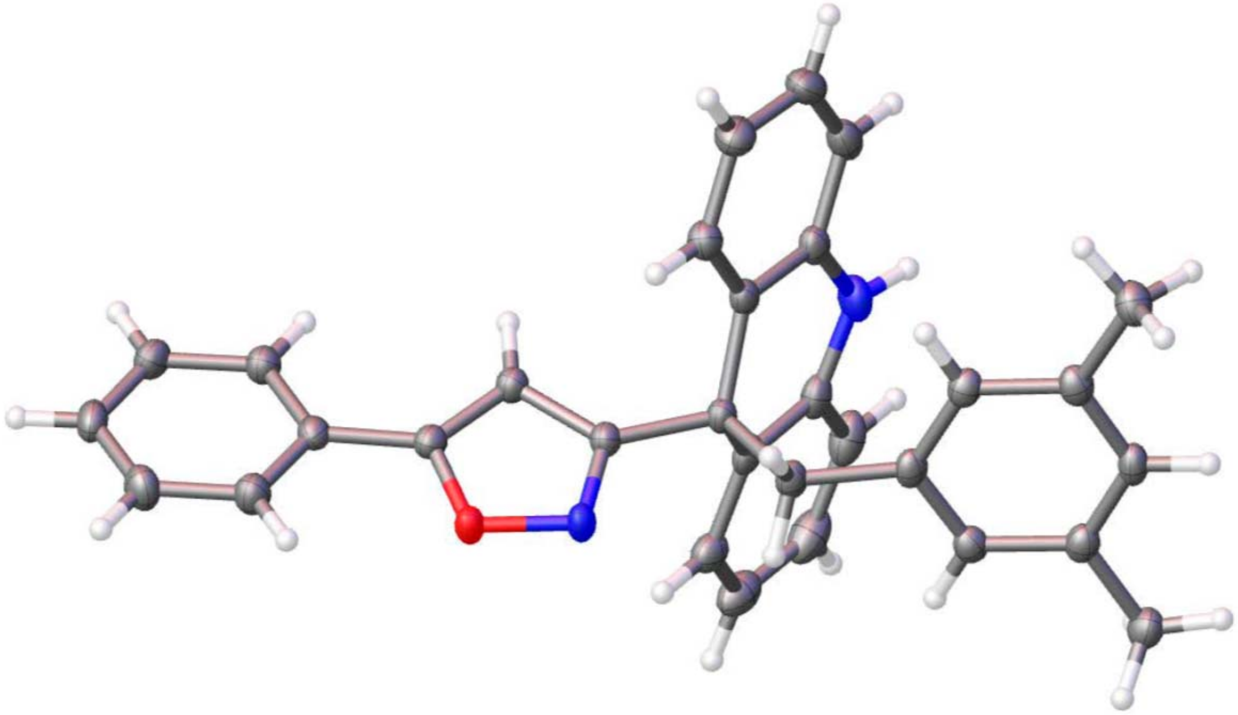

Figure S1. Molecular structure of compound **33b**, displacement parameters are drawn at 50% probability level.

Table S1. Crystal data and structure refinement for 33b.

Identification code	**33b** (23.168)
Empirical formula	$C_{31}H_{26}N_2O$
Formula weight	442.54
Temperature/K	100.00(10)
Crystal system	triclinic
Space group	P-1
a/Å	9.0617(2)
b/Å	10.7669(3)
c/Å	12.5728(3)
α/°	79.659(2)
β/°	75.465(2)
γ/°	84.439(2)
Volume/Å^3	1166.40(5)
Z	2
ρ_{calc}g/cm^3	1.260
μ/mm^{-1}	0.592
F(000)	468.0
Crystal size/mm^3	0.2 × 0.16 × 0.14
Radiation	Cu Kα (λ = 1.54184)
2Θ range for data collection/°	7.36 to 144.996
Index ranges	-11 ≤ h ≤ 10, -13 ≤ k ≤ 13, -15 ≤ l ≤ 15
Reflections collected	14436
Independent reflections	4614 [R_{int} = 0.0335, R_{sigma} = 0.0356]
Data/restraints/parameters	4614/0/309
Goodness-of-fit on F^2	1.057
Final R indexes [I>=2σ (I)]	R_1 = 0.0397, wR_2 = 0.1059
Final R indexes [all data]	R_1 = 0.0424, wR_2 = 0.1087
Largest diff. peak/hole / e Å^{-3}	0.26/-0.30

Table S2. Fractional Atomic Coordinates (×10^4) and Equivalent Isotropic Displacement Parameters (Å^2×10^3) for 33b. U_{eq} is defined as 1/3 of the trace of the orthogonalised U_{IJ} tensor.

Atom	*x*	*y*	*z*	U(eq)
O1	6956.6(9)	3982.6(8)	8820.7(7)	27.60(19)
N1	5892.5(11)	4719.9(10)	8300.8(8)	27.7(2)
N2	5997.4(12)	9241.5(9)	5809.4(9)	29.7(2)
C1	8319.6(12)	4525.1(11)	8453.4(9)	22.7(2)
C24	3215.2(12)	6968.7(10)	6333.9(9)	22.7(2)
C2	8185.7(12)	5591.2(10)	7715.3(9)	22.4(2)
C3	6633.9(12)	5666.0(10)	7650.5(9)	21.6(2)
C27	1050.2(12)	8260.7(10)	5250.8(10)	25.1(2)
C29	3370.4(12)	6969.3(10)	5198.7(9)	23.8(2)
C14	6737.2(12)	7021.9(10)	5796.0(9)	22.3(2)
C26	888.9(12)	8314.4(10)	6370.6(10)	24.5(2)
C13	6735.3(12)	8276.9(11)	5243.4(10)	24.9(2)
C10	5784.5(12)	6680.0(10)	6981.6(9)	21.2(2)
C15	7496.9(13)	6084.3(11)	5199.7(10)	26.7(2)
C4	9560.0(12)	3854.6(11)	8919.3(9)	23.1(2)
C12	5462.5(12)	9056.8(11)	6955.4(10)	26.9(2)
C25	1986.5(12)	7660.5(10)	6905.7(9)	23.6(2)
C5	10913.6(13)	4436.4(11)	8788.3(10)	27.3(2)
C28	2287.0(13)	7601.5(10)	4648.2(9)	24.9(2)
C11	5400.3(12)	7835.7(11)	7572.1(10)	23.8(2)
C23	4262.7(12)	6124.7(10)	6955.9(9)	23.7(2)
C19	4864.3(13)	7691.2(12)	8726.6(10)	29.0(3)
C7	11879.3(13)	2596.7(12)	9855.5(10)	29.9(3)
C18	7462.2(14)	8553.8(12)	4111.5(10)	31.2(3)
C30	-464.4(13)	9021.5(11)	7009.4(10)	29.9(3)
C31	2395.6(14)	7512.7(12)	3445.2(10)	31.0(3)
C6	12071.0(13)	3803.5(12)	9258.4(10)	30.7(3)
C8	10541.9(15)	2003.5(12)	9973.6(11)	33.2(3)
C9	9383.2(14)	2627.0(12)	9510.4(10)	29.8(3)
C16	8208.6(14)	6356.4(13)	4077.6(10)	33.6(3)
C22	4963.9(14)	10100.4(12)	7502.9(12)	35.1(3)
C17	8170.9(14)	7602.3(14)	3535.9(10)	35.0(3)
C20	4388.7(14)	8727.8(14)	9268.0(11)	36.1(3)
C21	4430.8(15)	9930.9(13)	8644.6(13)	39.2(3)

Table S3. Anisotropic Displacement Parameters (Å^2×10^3) for 33b. The Anisotropic displacement factor exponent takes the form: -2π^2[h^{2}a*^{2}U$_{11}$+2hka*b*U$_{12}$+…].

Atom	U_{11}	U_{22}	U_{33}	U_{23}	U_{13}	U_{12}
O1	19.1(4)	32.7(4)	28.7(4)	7.5(3)	-10.0(3)	-2.3(3)
N1	19.2(4)	33.2(5)	28.6(5)	6.4(4)	-10.2(4)	-0.5(4)
N2	32.8(5)	22.3(5)	35.0(5)	1.7(4)	-15.2(4)	0.3(4)
C1	18.8(5)	28.9(5)	20.7(5)	-3.6(4)	-5.5(4)	-1.5(4)
C24	19.4(5)	22.1(5)	28.6(5)	-0.5(4)	-10.3(4)	-5.0(4)
C2	19.3(5)	24.9(5)	23.5(5)	-1.7(4)	-7.3(4)	-1.7(4)
C3	18.9(5)	25.6(5)	20.6(5)	-2.0(4)	-6.9(4)	-0.6(4)
C27	22.4(5)	23.6(5)	31.2(6)	1.0(4)	-13.5(4)	-1.4(4)
C29	20.7(5)	23.1(5)	28.9(6)	-4.1(4)	-8.2(4)	-1.9(4)
C14	18.0(5)	26.6(5)	23.1(5)	0.2(4)	-9.1(4)	-2.2(4)
C26	20.9(5)	22.2(5)	30.1(6)	-0.3(4)	-7.8(4)	-3.5(4)
C13	20.9(5)	27.7(5)	27.8(6)	1.2(4)	-11.7(4)	-3.7(4)
C10	17.4(5)	24.1(5)	22.7(5)	-0.7(4)	-8.1(4)	-1.5(4)
C15	25.4(5)	29.8(6)	25.8(5)	-2.6(4)	-9.2(4)	-0.5(4)
C4	21.2(5)	29.3(5)	18.5(5)	-3.0(4)	-6.0(4)	1.5(4)
C12	20.9(5)	28.1(6)	35.8(6)	-5.7(5)	-14.7(4)	0.9(4)
C25	22.1(5)	25.0(5)	24.4(5)	-0.4(4)	-8.4(4)	-5.0(4)
C5	23.0(5)	31.8(6)	26.7(5)	-0.7(4)	-8.2(4)	-1.7(4)
C28	26.0(5)	23.4(5)	27.8(6)	-1.1(4)	-11.5(4)	-5.2(4)
C11	16.0(5)	28.5(5)	29.8(6)	-5.4(4)	-10.8(4)	0.1(4)
C23	19.3(5)	24.9(5)	27.6(5)	0.3(4)	-9.7(4)	-3.2(4)
C19	20.1(5)	39.0(6)	30.3(6)	-8.3(5)	-8.6(4)	-1.8(4)
C7	24.2(5)	38.2(6)	28.0(6)	-5.0(5)	-11.2(4)	7.7(5)
C18	27.6(6)	35.4(6)	30.0(6)	7.2(5)	-12.4(5)	-7.9(5)
C30	24.9(6)	30.3(6)	33.1(6)	-1.2(5)	-8.2(5)	1.9(4)
C31	33.5(6)	32.6(6)	30.1(6)	-4.5(5)	-14.6(5)	0.5(5)
C6	20.4(5)	41.1(7)	30.7(6)	-3.0(5)	-8.4(4)	-2.0(5)
C8	33.2(6)	31.1(6)	34.8(6)	2.9(5)	-13.9(5)	0.6(5)
C9	25.2(6)	32.7(6)	31.8(6)	0.6(5)	-10.5(5)	-3.6(5)
C16	30.7(6)	43.7(7)	26.1(6)	-7.3(5)	-6.5(5)	2.2(5)
C22	30.6(6)	29.0(6)	53.0(8)	-12.1(5)	-21.4(6)	3.3(5)
C17	28.5(6)	50.9(8)	22.9(6)	2.8(5)	-5.9(5)	-6.2(5)
C20	25.1(6)	52.3(8)	36.6(7)	-19.5(6)	-10.0(5)	0.4(5)
C21	29.5(6)	44.1(7)	53.5(8)	-26.6(6)	-17.2(6)	6.6(5)

Table S4. Bond Lengths for 33b.

Atom	Atom	Length/Å	Atom	Atom	Length/Å
O1	N1	1.4032(11)	C13	C18	1.4012(17)
O1	C1	1.3552(13)	C10	C11	1.5293(15)
N1	C3	1.3098(15)	C10	C23	1.5650(14)
N2	C13	1.3870(16)	C15	C16	1.3870(17)
N2	C12	1.3841(16)	C4	C5	1.3909(16)
C1	C2	1.3567(15)	C4	C9	1.3977(16)
C1	C4	1.4659(14)	C12	C11	1.3997(16)
C24	C29	1.3985(16)	C12	C22	1.4013(17)
C24	C25	1.3933(16)	C5	C6	1.3935(16)
C24	C23	1.5128(14)	C28	C31	1.5107(16)
C2	C3	1.4225(14)	C11	C19	1.3954(17)
C3	C10	1.5246(14)	C19	C20	1.3881(18)
C27	C26	1.3897(17)	C7	C6	1.3821(18)
C27	C28	1.3952(17)	C7	C8	1.3873(18)
C29	C28	1.3959(15)	C18	C17	1.375(2)
C14	C13	1.4041(16)	C8	C9	1.3864(16)
C14	C10	1.5237(15)	C16	C17	1.3926(19)
C14	C15	1.3904(16)	C22	C21	1.379(2)
C26	C25	1.4027(15)	C20	C21	1.387(2)
C26	C30	1.5076(16)			

Table S5. Bond Angles for 33b.

Atom	Atom	Atom	Angle/°	Atom	Atom	Atom	Angle/°
C1	O1	N1	108.72(8)	C11	C10	C23	108.90(8)
C3	N1	O1	105.89(8)	C16	C15	C14	121.70(11)
C12	N2	C13	122.16(10)	C5	C4	C1	120.46(10)
O1	C1	C2	109.49(9)	C5	C4	C9	119.44(10)
O1	C1	C4	115.20(9)	C9	C4	C1	120.10(10)
C2	C1	C4	135.30(10)	N2	C12	C11	120.60(10)
C29	C24	C23	120.54(10)	N2	C12	C22	119.74(11)
C25	C24	C29	118.80(10)	C11	C12	C22	119.65(12)
C25	C24	C23	120.35(10)	C24	C25	C26	121.11(10)
C1	C2	C3	104.43(9)	C4	C5	C6	120.04(11)
N1	C3	C2	111.46(9)	C27	C28	C29	118.53(10)
N1	C3	C10	119.38(9)	C27	C28	C31	120.46(10)
C2	C3	C10	129.12(10)	C29	C28	C31	120.91(10)
C26	C27	C28	121.69(10)	C12	C11	C10	120.41(10)
C28	C29	C24	121.23(10)	C19	C11	C10	120.59(10)
C13	C14	C10	120.54(10)	C19	C11	C12	118.80(11)
C15	C14	C13	118.45(10)	C24	C23	C10	117.18(9)
C15	C14	C10	120.68(10)	C20	C19	C11	121.41(12)
C27	C26	C25	118.58(10)	C6	C7	C8	120.04(10)
C27	C26	C30	120.85(10)	C17	C18	C13	120.39(11)
C25	C26	C30	120.53(10)	C7	C6	C5	120.19(11)
N2	C13	C14	120.27(10)	C9	C8	C7	120.15(11)
N2	C13	C18	119.85(10)	C8	C9	C4	120.13(11)
C18	C13	C14	119.87(11)	C15	C16	C17	119.16(12)
C3	C10	C11	109.03(9)	C21	C22	C12	120.38(12)
C3	C10	C23	107.46(8)	C18	C17	C16	120.40(11)
C14	C10	C3	110.49(8)	C21	C20	C19	119.17(12)
C14	C10	C11	111.40(9)	C22	C21	C20	120.57(12)
C14	C10	C23	109.47(8)				

Table S6. Torsion Angles for 33b.

A	B	C	D	Angle/°	A	B	C	D	Angle/°
O1	N1	C3	C2	-0.02(12)	C13	C14	C10	C3	-142.68(10)
O1	N1	C3	C10	-178.18(9)	C13	C14	C10	C11	-21.34(13)
O1	C1	C2	C3	-0.17(12)	C13	C14	C10	C23	99.16(11)
O1	C1	C4	C5	-166.94(10)	C13	C14	C15	C16	-1.72(16)
O1	C1	C4	C9	12.38(15)	C13	C18	C17	C16	-1.66(18)
N1	O1	C1	C2	0.16(12)	C10	C14	C13	N2	6.93(15)
N1	O1	C1	C4	-179.61(9)	C10	C14	C13	C18	-172.11(9)
N1	C3	C10	C14	-136.69(11)	C10	C14	C15	C16	171.67(10)
N1	C3	C10	C11	100.57(12)	C10	C11	C19	C20	-174.52(10)
N1	C3	C10	C23	-17.31(14)	C15	C14	C13	N2	-179.67(10)
N2	C13	C18	C17	-178.67(10)	C15	C14	C13	C18	1.29(15)
N2	C12	C11	C10	-5.60(15)	C15	C14	C10	C3	44.07(13)
N2	C12	C11	C19	179.53(10)	C15	C14	C10	C11	165.42(9)
N2	C12	C22	C21	-179.88(11)	C15	C14	C10	C23	-74.09(12)
C1	O1	N1	C3	-0.08(12)	C15	C16	C17	C18	1.24(18)
C1	C2	C3	N1	0.12(13)	C4	C1	C2	C3	179.54(12)
C1	C2	C3	C10	178.05(10)	C4	C5	C6	C7	0.04(18)
C1	C4	C5	C6	178.36(10)	C12	N2	C13	C14	10.87(16)
C1	C4	C9	C8	-178.48(11)	C12	N2	C13	C18	-170.09(10)
C24	C29	C28	C27	1.28(16)	C12	C11	C19	C20	0.34(16)
C24	C29	C28	C31	-175.13(10)	C12	C22	C21	C20	0.40(18)
C2	C1	C4	C5	13.37(19)	C25	C24	C29	C28	-2.79(16)
C2	C1	C4	C9	-167.31(13)	C25	C24	C23	C10	-97.11(12)
C2	C3	C10	C14	45.52(15)	C5	C4	C9	C8	0.84(18)
C2	C3	C10	C11	-77.22(13)	C28	C27	C26	C25	-1.52(17)
C2	C3	C10	C23	164.90(11)	C28	C27	C26	C30	-179.40(10)
C3	C10	C11	C12	142.88(10)	C11	C10	C23	C24	61.95(12)
C3	C10	C11	C19	-42.34(13)	C11	C12	C22	C21	0.76(17)
C3	C10	C23	C24	179.92(9)	C11	C19	C20	C21	0.80(17)
C27	C26	C25	C24	-0.05(16)	C23	C24	C29	C28	170.83(10)
C29	C24	C25	C26	2.15(16)	C23	C24	C25	C26	-171.47(10)
C29	C24	C23	C10	89.37(12)	C23	C10	C11	C12	-100.15(11)
C14	C13	C18	C17	0.37(17)	C23	C10	C11	C19	74.63(12)
C14	C10	C11	C12	20.69(13)	C19	C20	C21	C22	-1.17(18)
C14	C10	C11	C19	-164.54(9)	C7	C8	C9	C4	0.21(19)
C14	C10	C23	C24	-60.06(12)	C30	C26	C25	C24	177.84(10)
C14	C15	C16	C17	0.47(18)	C6	C7	C8	C9	-1.15(19)
C26	C27	C28	C29	0.91(17)	C8	C7	C6	C5	1.02(18)
C26	C27	C28	C31	177.34(10)	C9	C4	C5	C6	-0.97(17)
C13	N2	C12	C11	-11.54(16)	C22	C12	C11	C10	173.75(10)
C13	N2	C12	C22	169.10(10)	C22	C12	C11	C19	-1.12(15)

Table S7. Hydrogen Atom Coordinates (Å×10^4) and Isotropic Displacement Parameters (Å^2×10^3) for 33b.

Atom	*x*	*y*	*z*	U(eq)
H2	5865	9996	5427	36
H2A	8955	6160	7329	27
H27	299	8684	4886	30
H29	4229	6531	4795	29
H15	7529	5235	5571	32
H25	1890	7690	7671	28
H5	11049	5266	8378	33
H23A	4527	5343	6622	28
H23B	3683	5879	7734	28
H19	4824	6866	9151	35
H7	12664	2173	10185	36
H18	7465	9404	3739	37
H30A	-112	9726	7260	45
H30B	-1003	8448	7657	45
H30C	-1158	9350	6527	45
H31A	1604	6977	3404	47
H31B	3403	7143	3119	47
H31C	2250	8360	3032	47
H6	12994	4203	9168	37
H8	10420	1168	10373	40
H9	8467	2219	9595	36
H16	8715	5701	3684	40
H22	4993	10929	7085	42
H17	8637	7796	2764	42
H20	4039	8615	10056	43
H21	4089	10644	9007	47

UV-VIS ABSORPTION SPECTRA

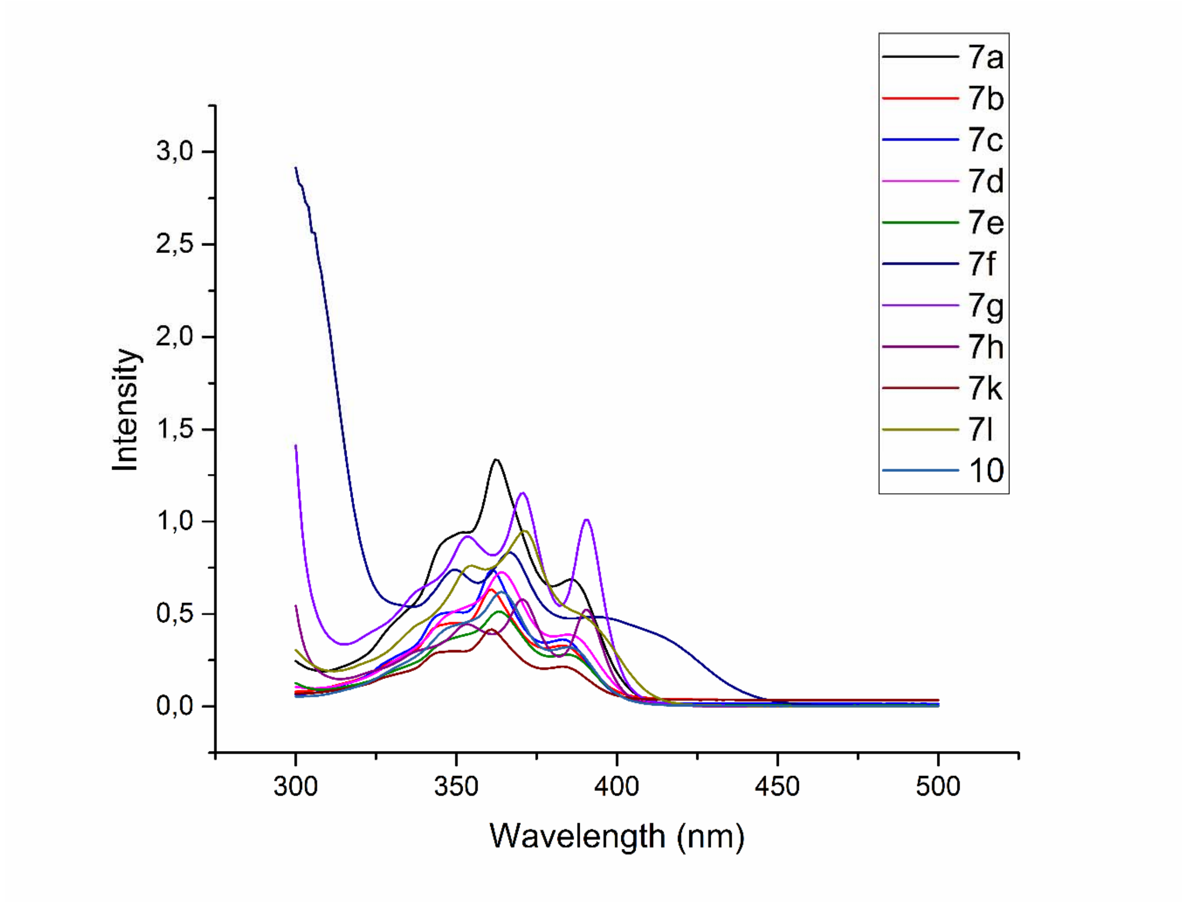

Figure S2. UV-VIS absorption spectra of isoxazoles **7** and **10**.

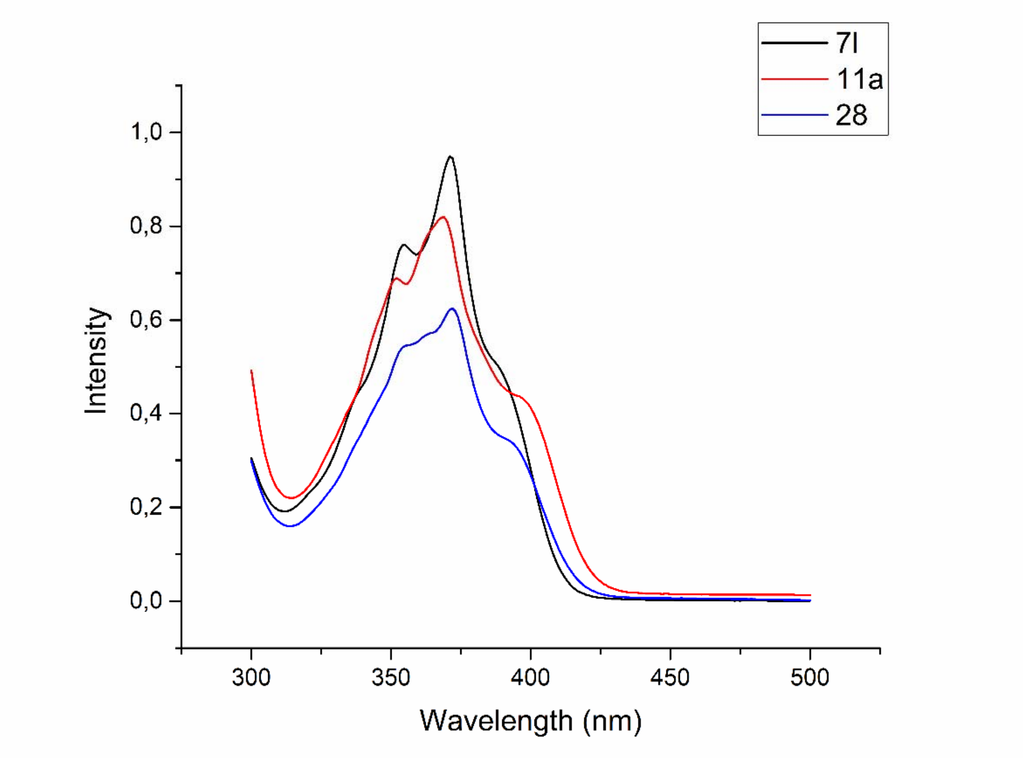

Figure S3. UV-VIS absorption spectra of azirines **11a**, **28** and isoxazole **7l**.

2-Methyl-9-phenylacridine (4e), ¹H NMR, CDCl$_3$, 400 MHz

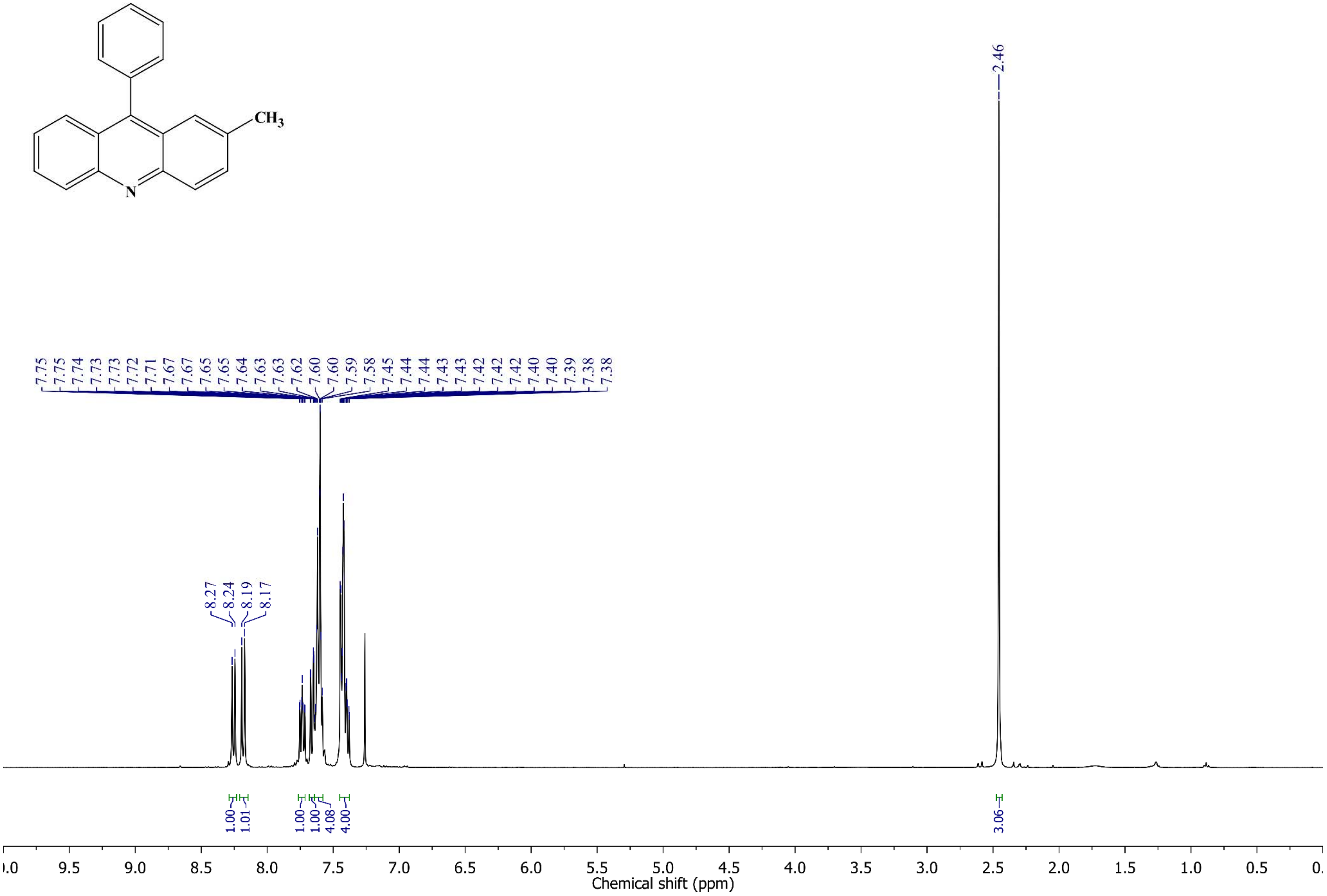

2-Methyl-9-phenylacridine (4e), $^{13}C\{^{1}H\}$ NMR, $CDCl_3$, 100 MHz

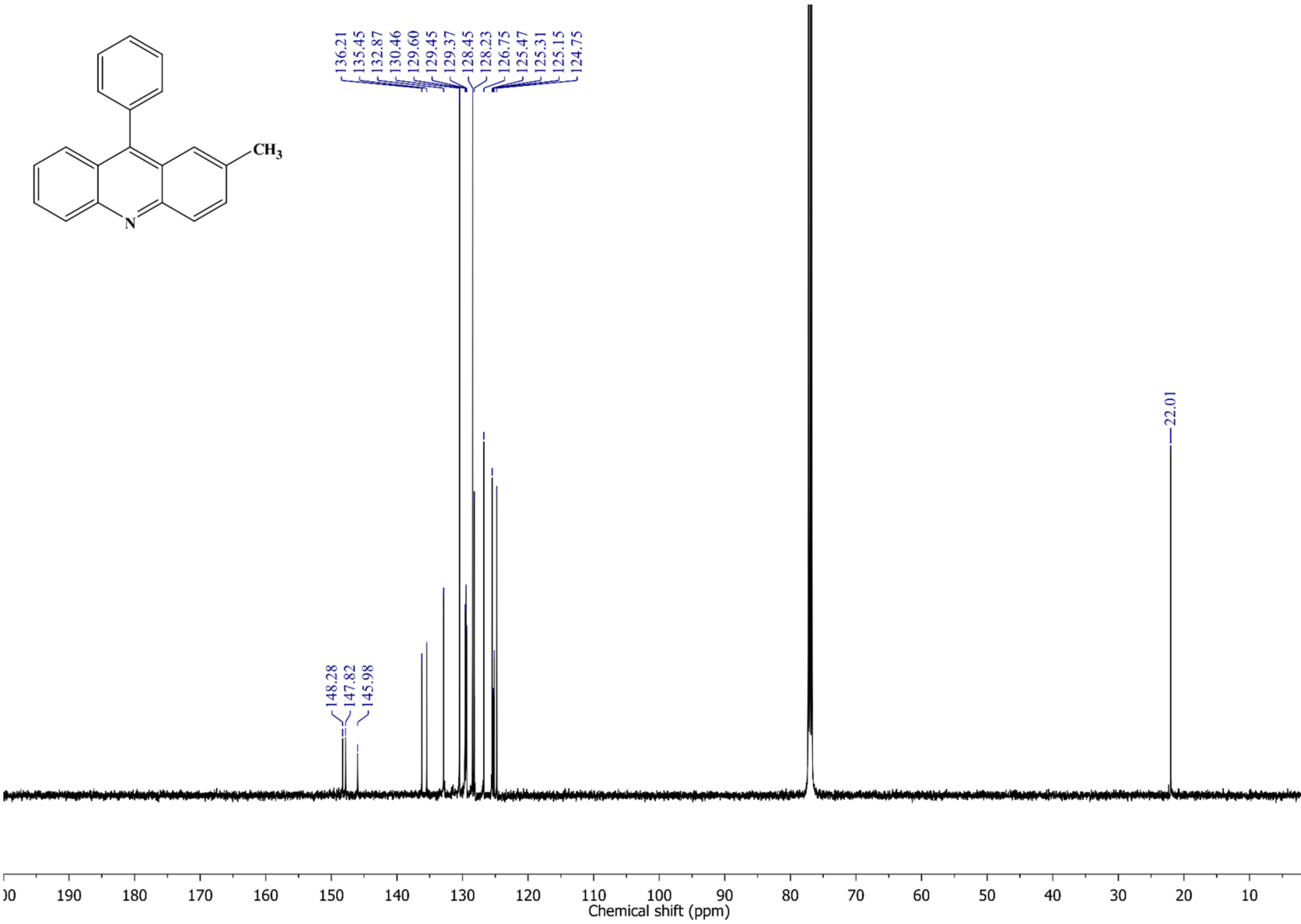

2-Methyl-9-phenylacridine (4e), DEPT, $CDCl_3$, 100 MHz

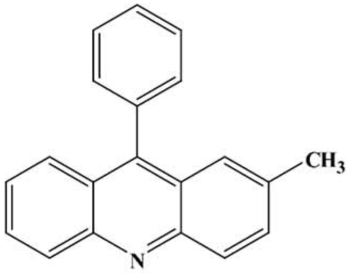

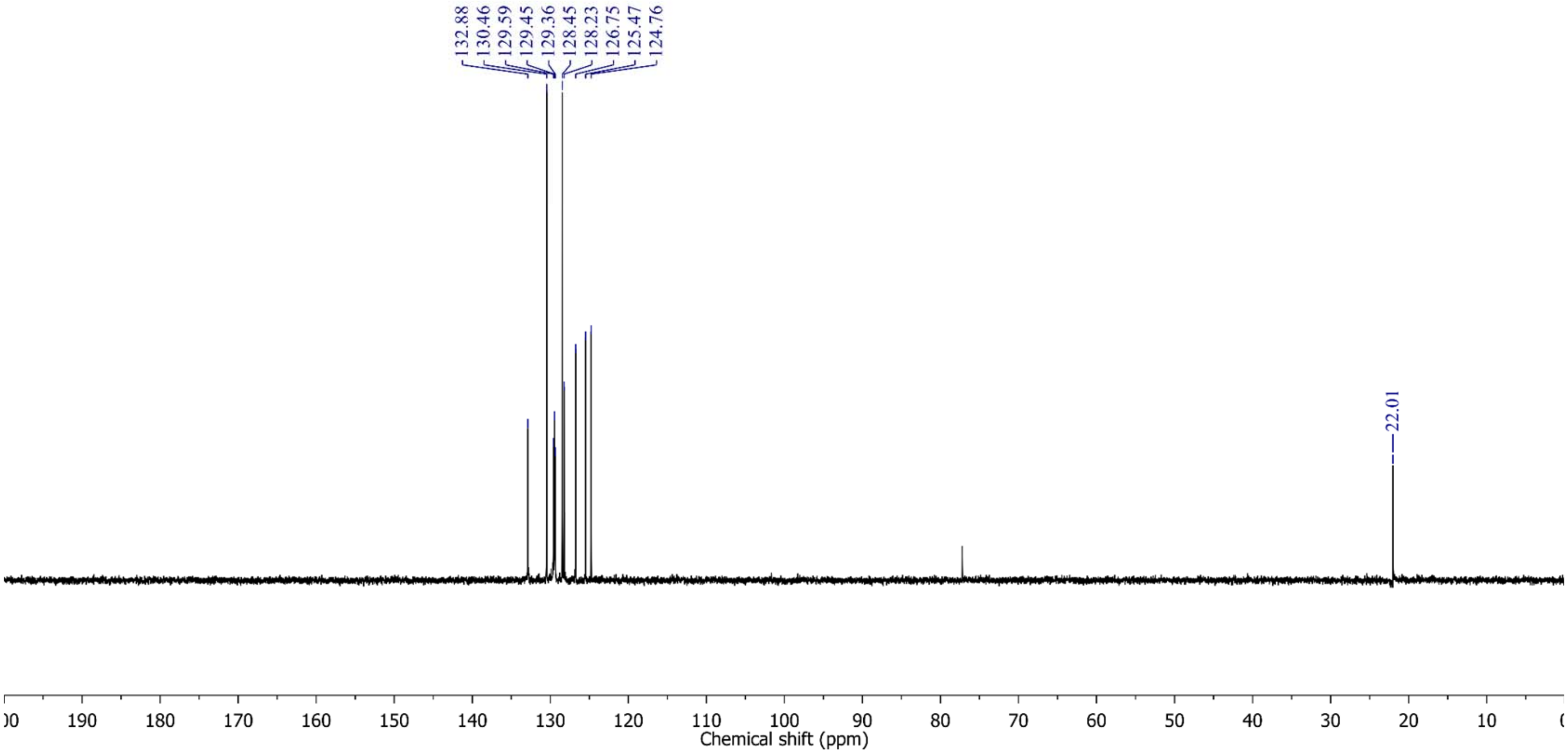

2-Nitroacridine-9-carbaldehyde (5c), ^{1}H NMR, DMSO-d_6, 400 MHz

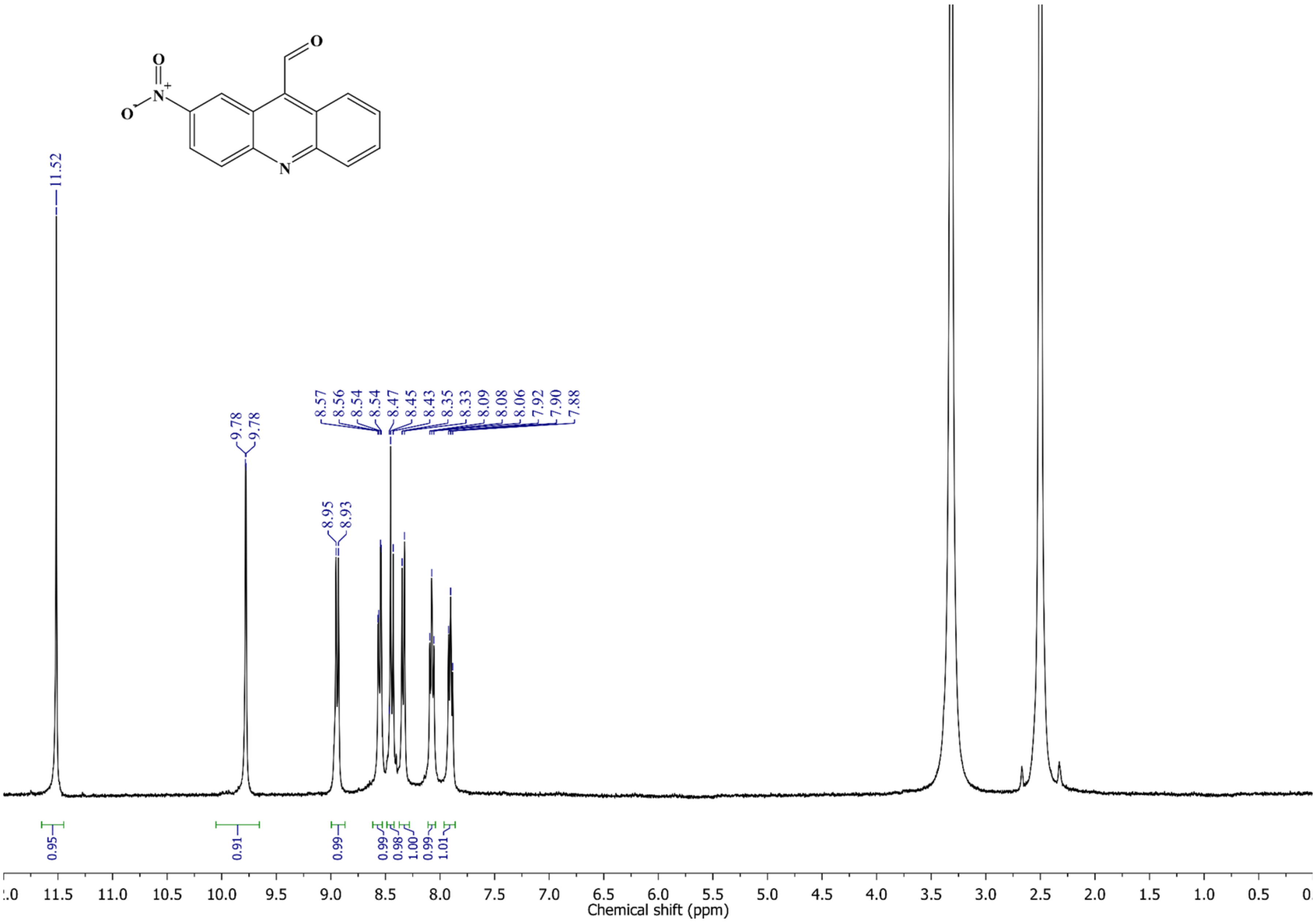

2-Nitroacridine-9-carbaldehyde (5c), $^{13}C\{^1H\}$ NMR, DMSO-d_6, 100 MHz

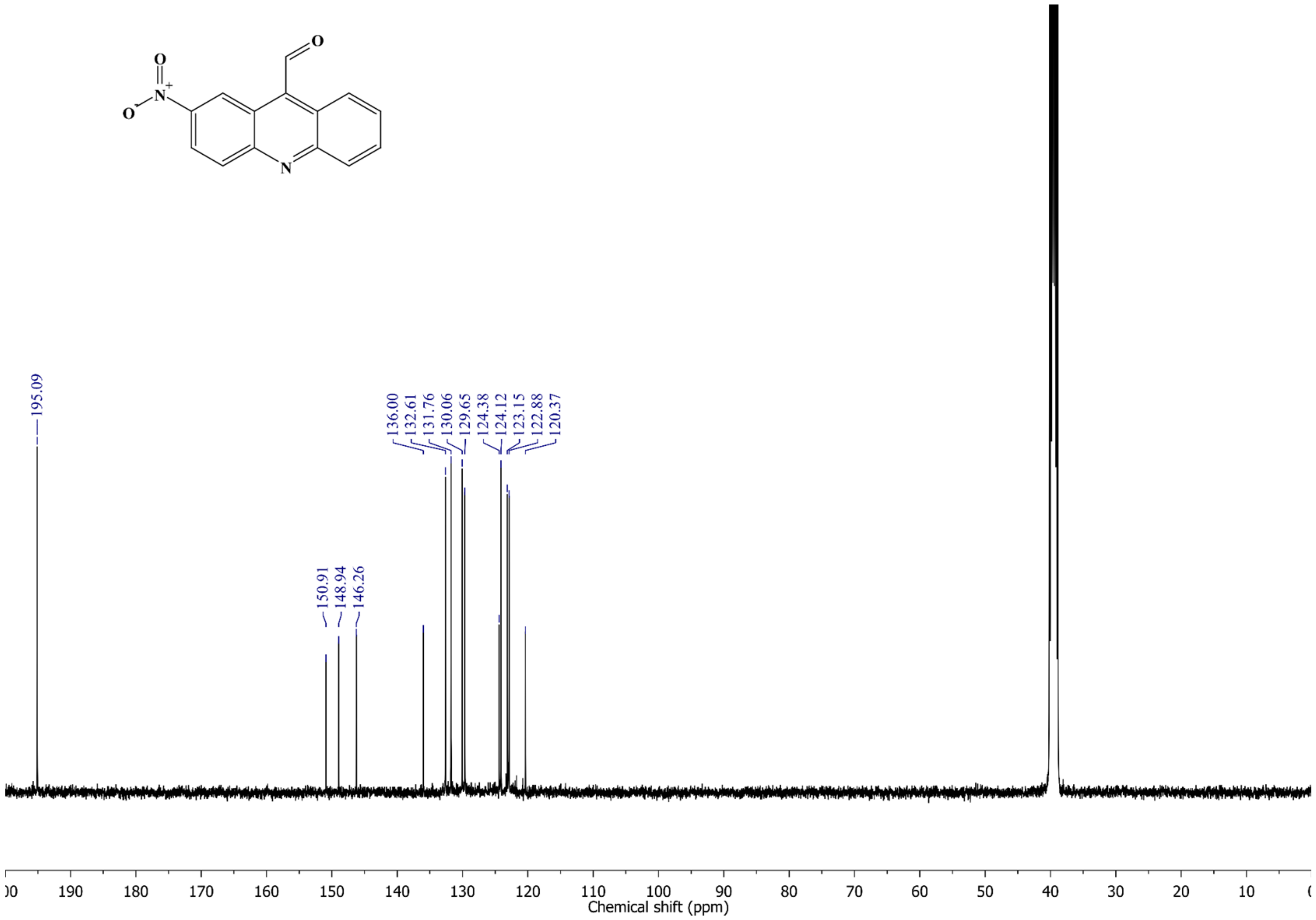

2-Nitroacridine-9-carbaldehyde (5c), DEPT, DMSO-d_6, 100 MHz

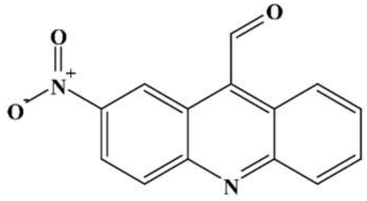

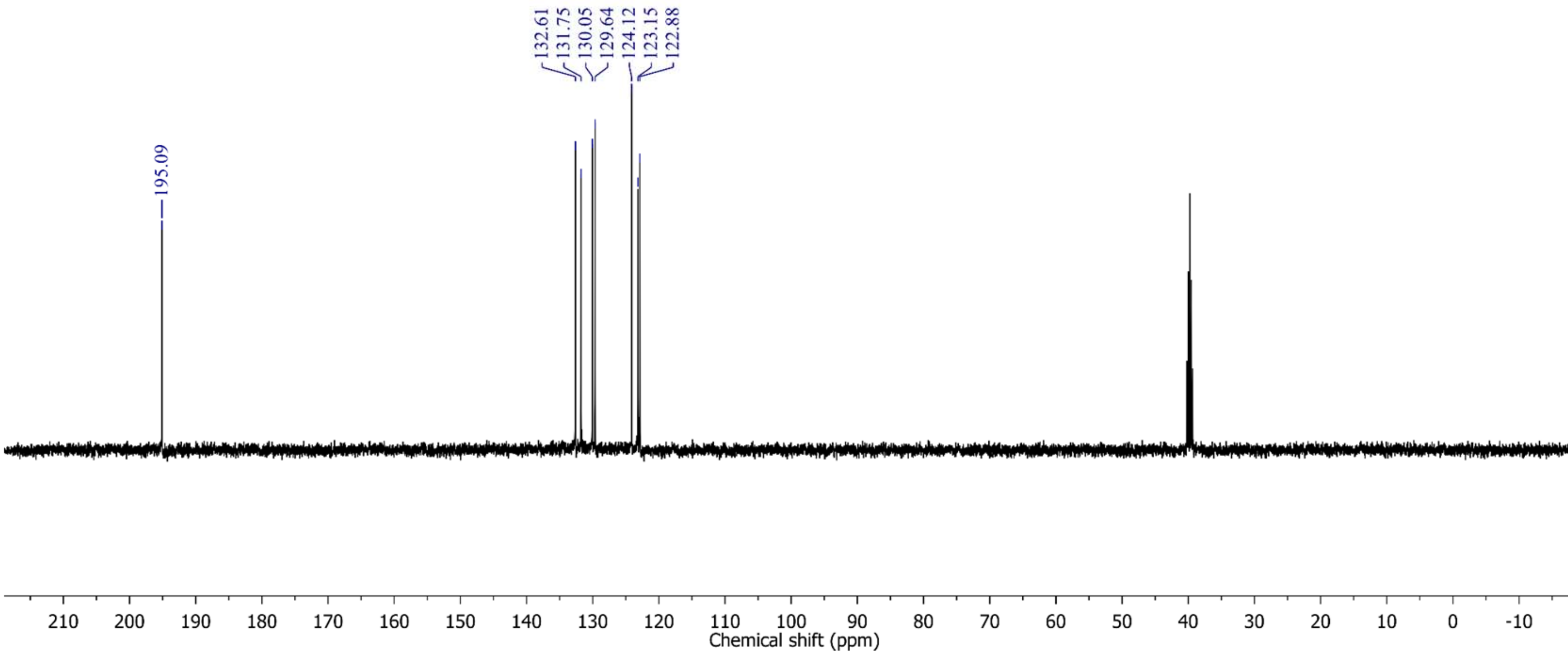

9-Methylbenzo[*c*]acridine-7-carbaldehyde (5d), ^{1}H NMR, $CDCl_3$, 400 MHz

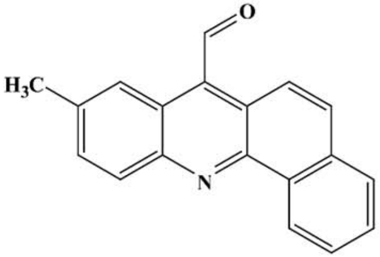

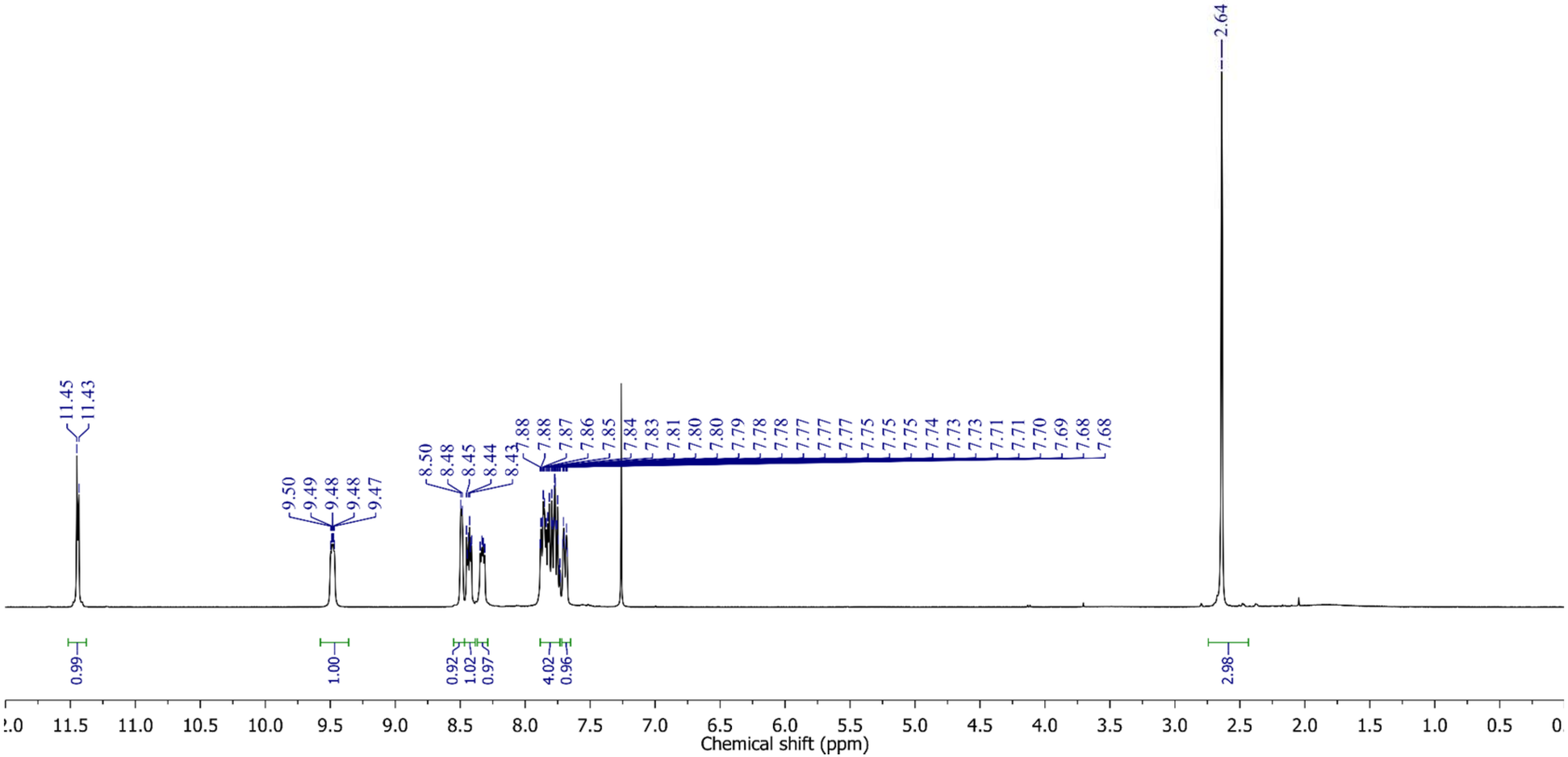

9-Methylbenzo[*c*]acridine-7-carbaldehyde (5d), $^{13}C\{^{1}H\}$ NMR, $CDCl_3$, 100 MHz

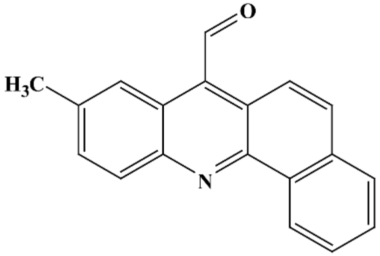

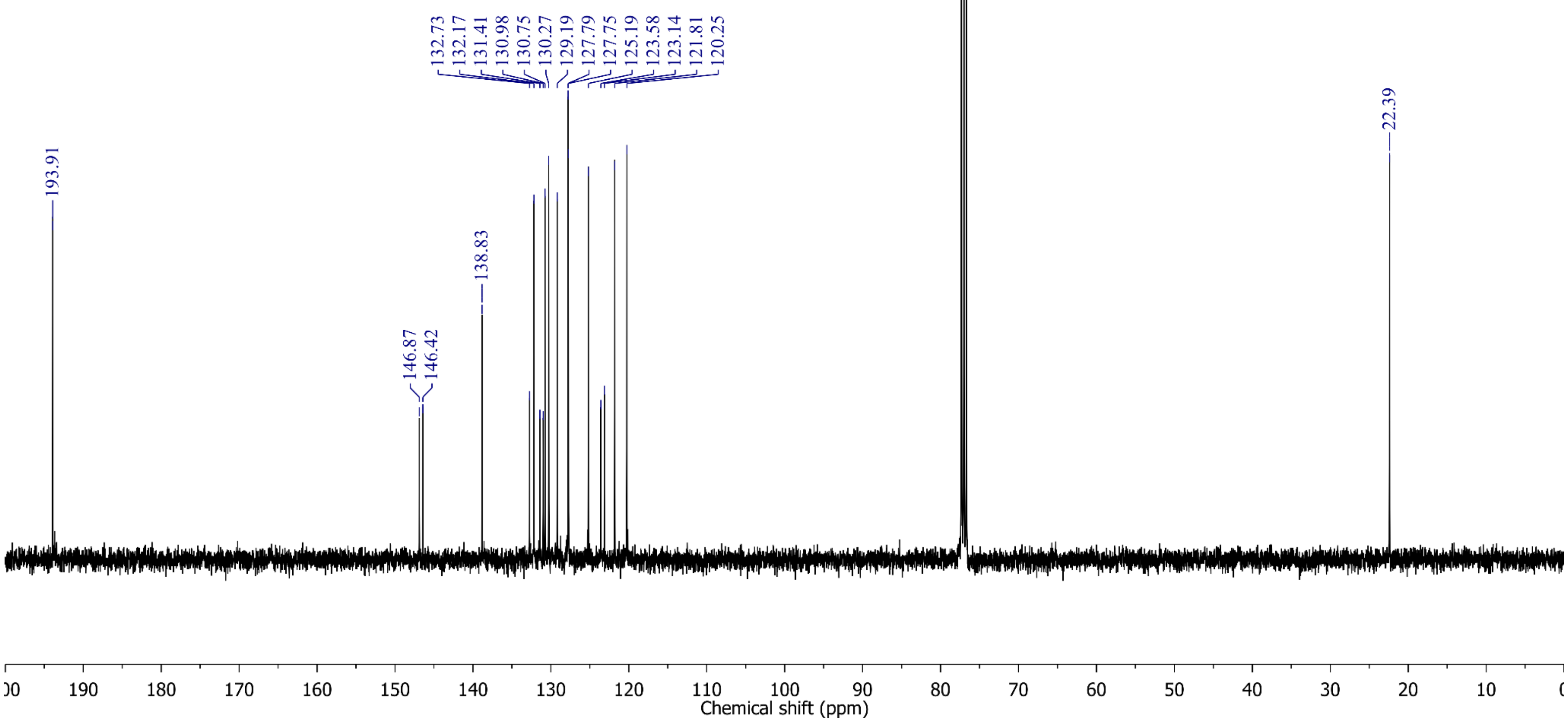

9-Methylbenzo[*c*]acridine-7-carbaldehyde (5d), DEPT, $CDCl_3$, 100 MHz

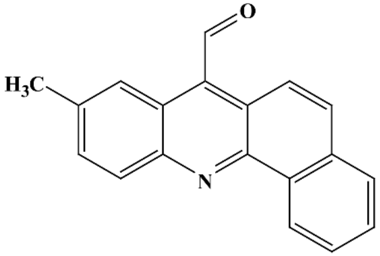

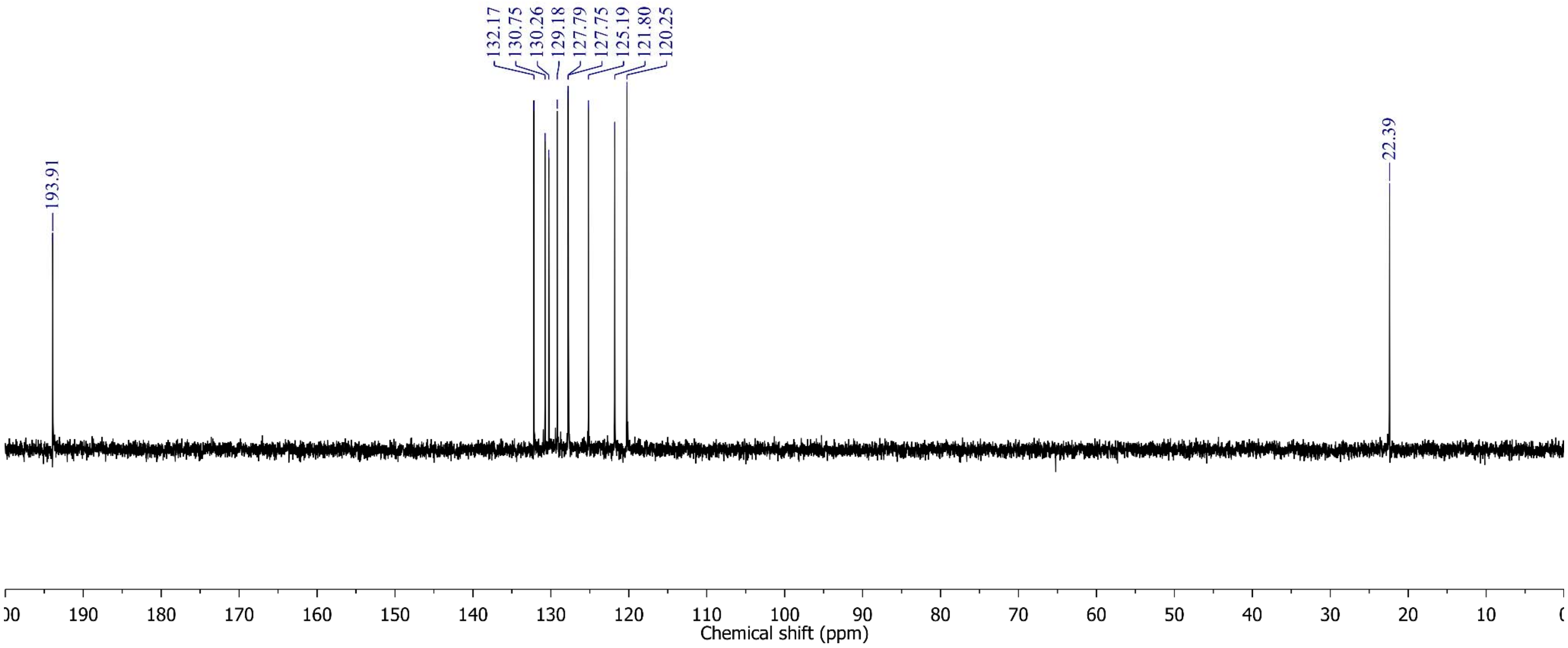

9-Phenylacridine-2-carbaldehyde (5e), ^{1}H NMR, $CDCl_3$, 400 MHz

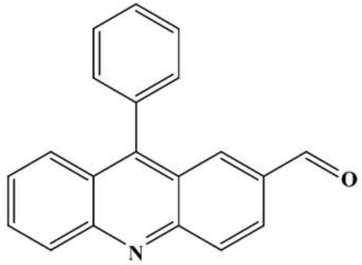

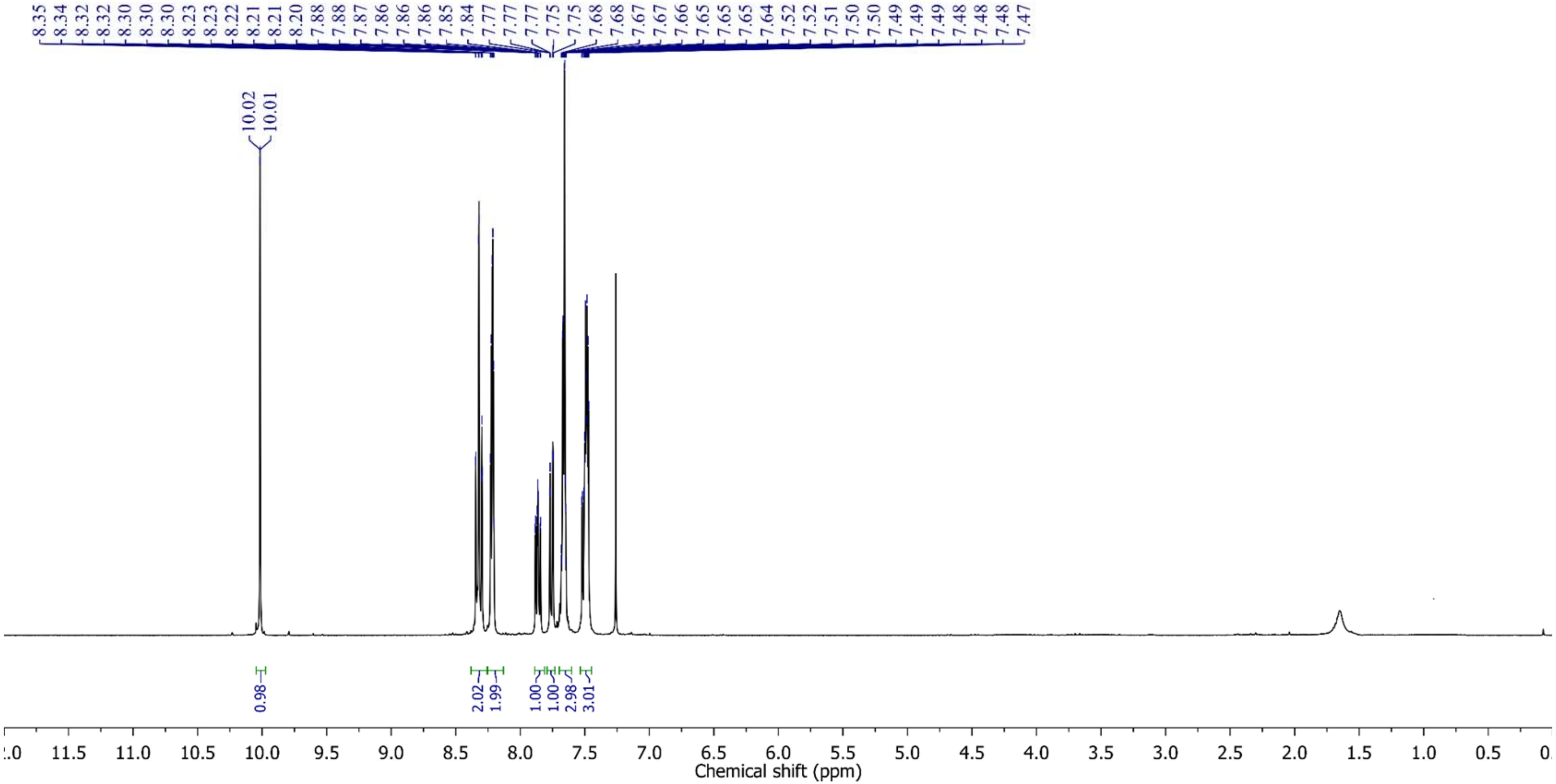

9-Phenylacridine-2-carbaldehyde (5e), $^{13}C\{^1H\}$ NMR, $CDCl_3$, 100 MHz

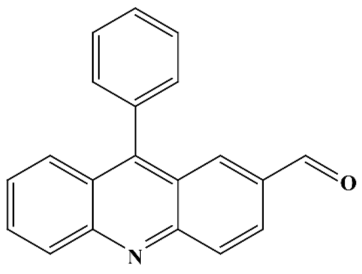

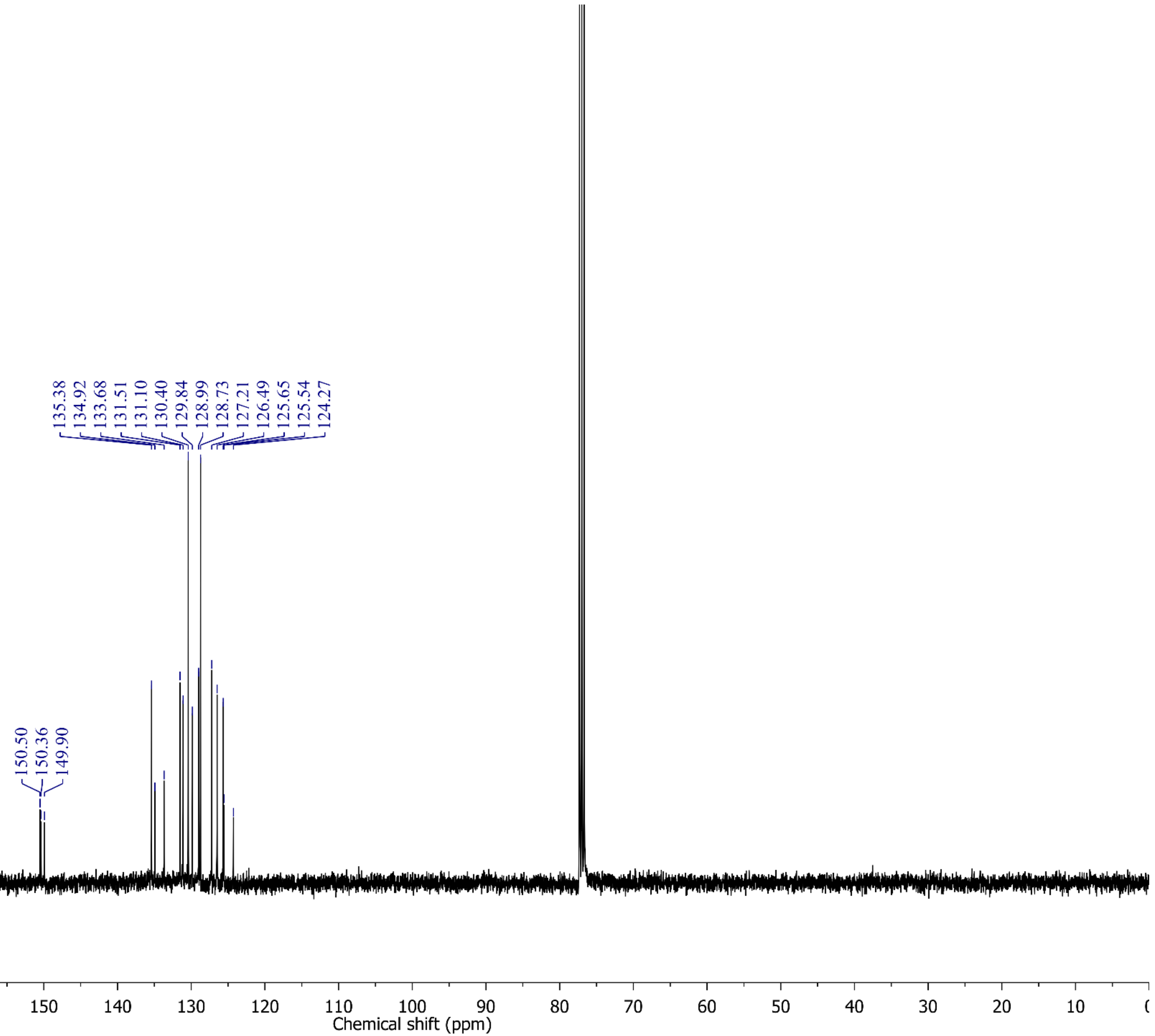

9-Phenylacridine-2-carbaldehyde (5e), DEPT, $CDCl_3$, 100 MHz

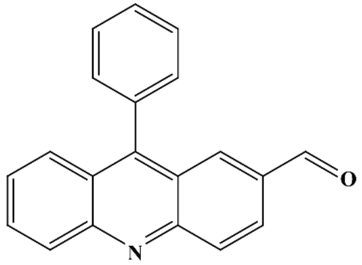

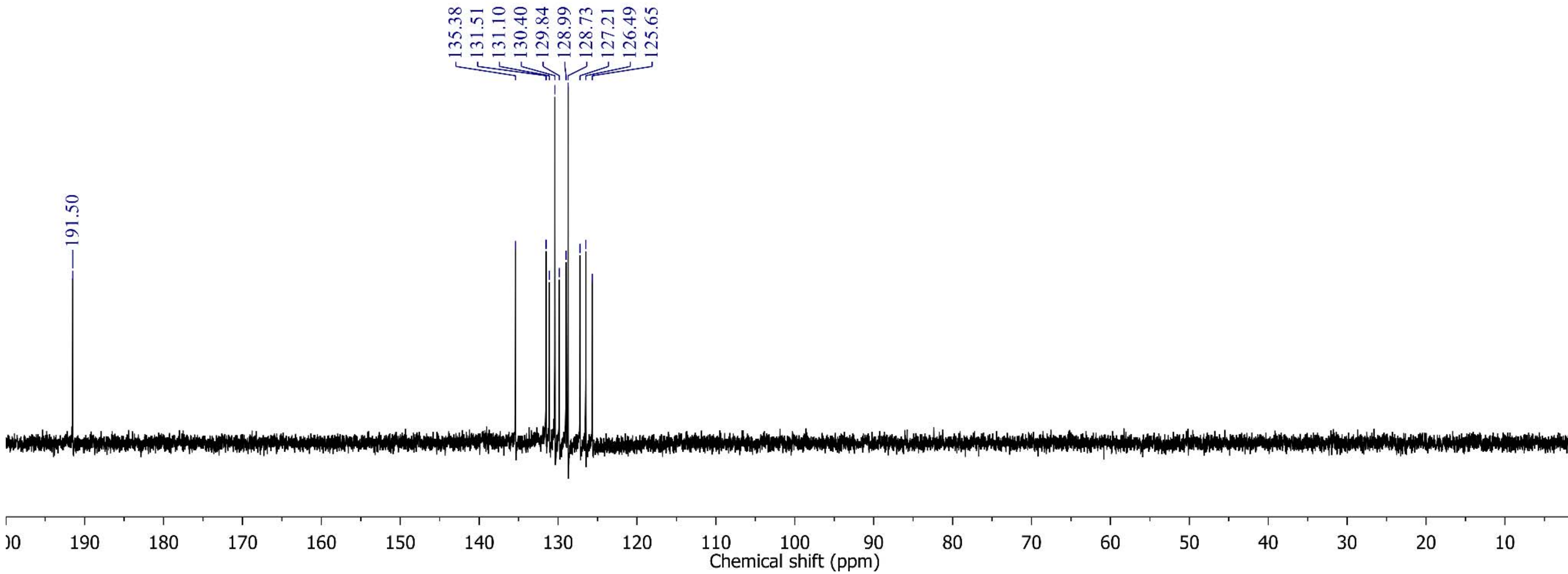

2-Methylacridine-9-carbaldehyde oxime (1b), ^{1}H NMR, DMSO-d_6, 400 MHz

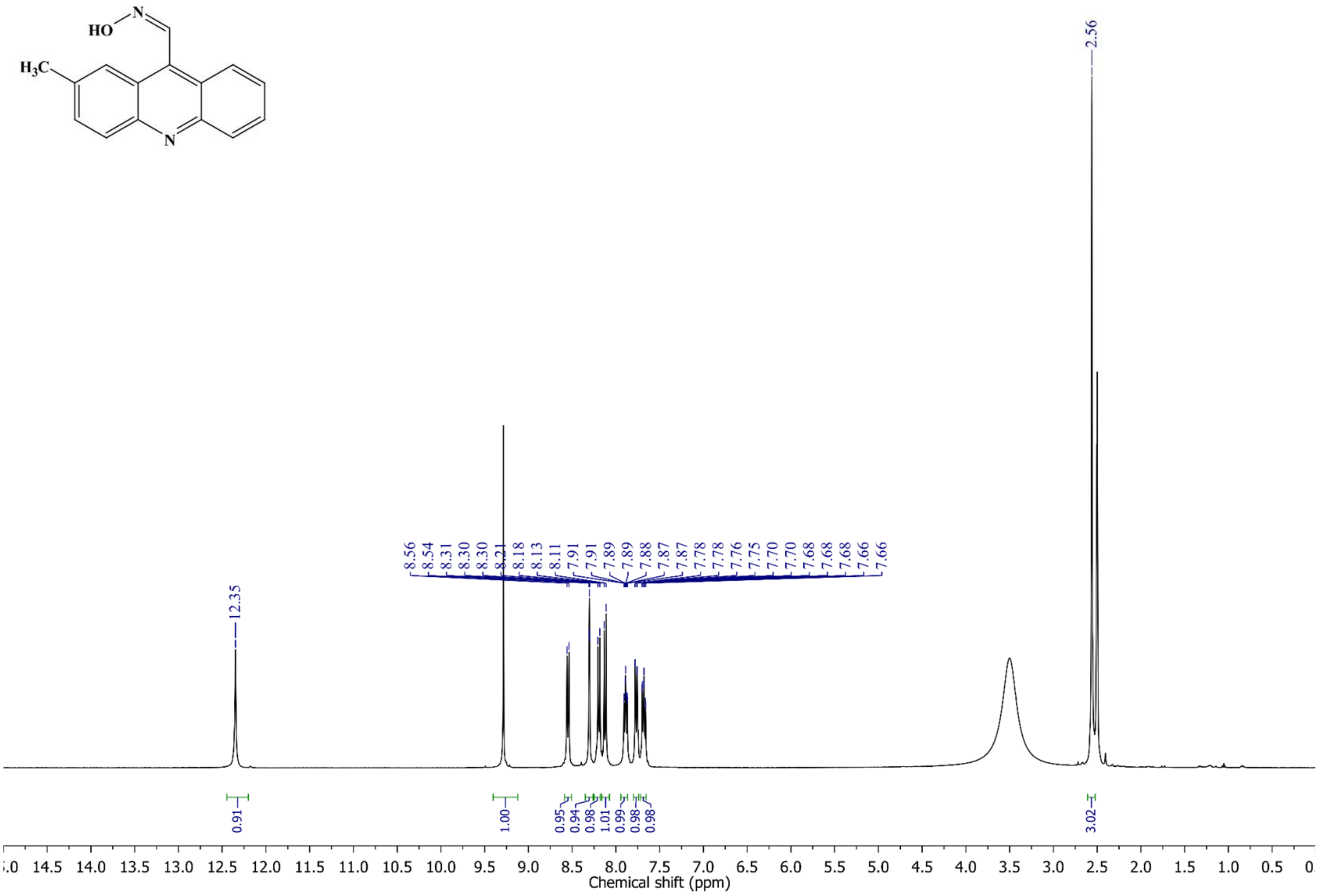

2-Methylacridine-9-carbaldehyde oxime (1b), $^{13}C\{^{1}H\}$ NMR, DMSO-d_6, 100 MHz

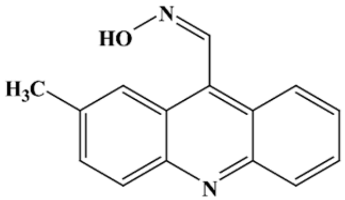

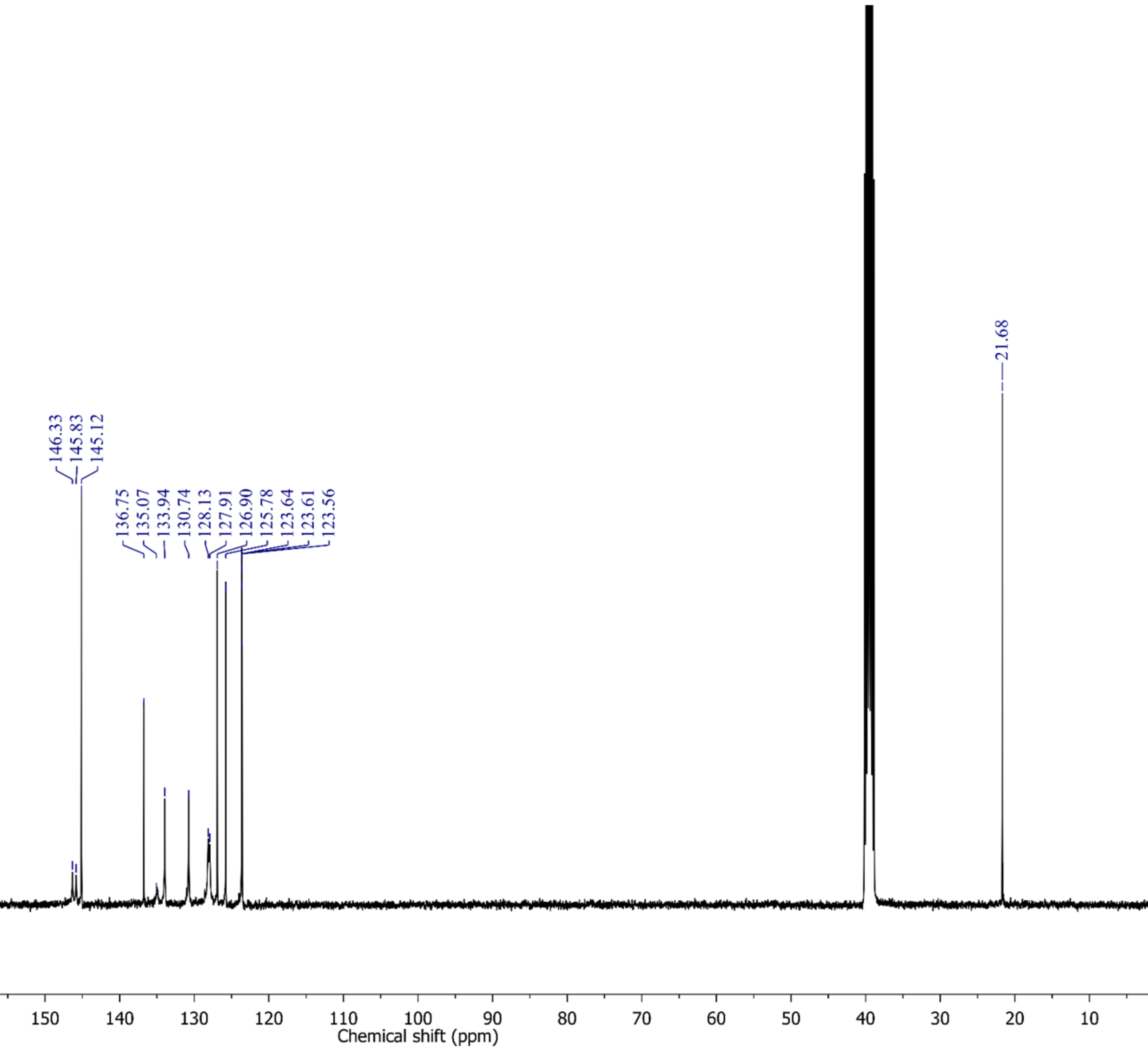

2-Methylacridine-9-carbaldehyde oxime (1b), DEPT, DMSO-d_6, 100 MHz

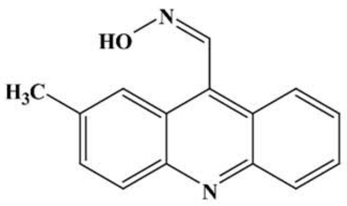

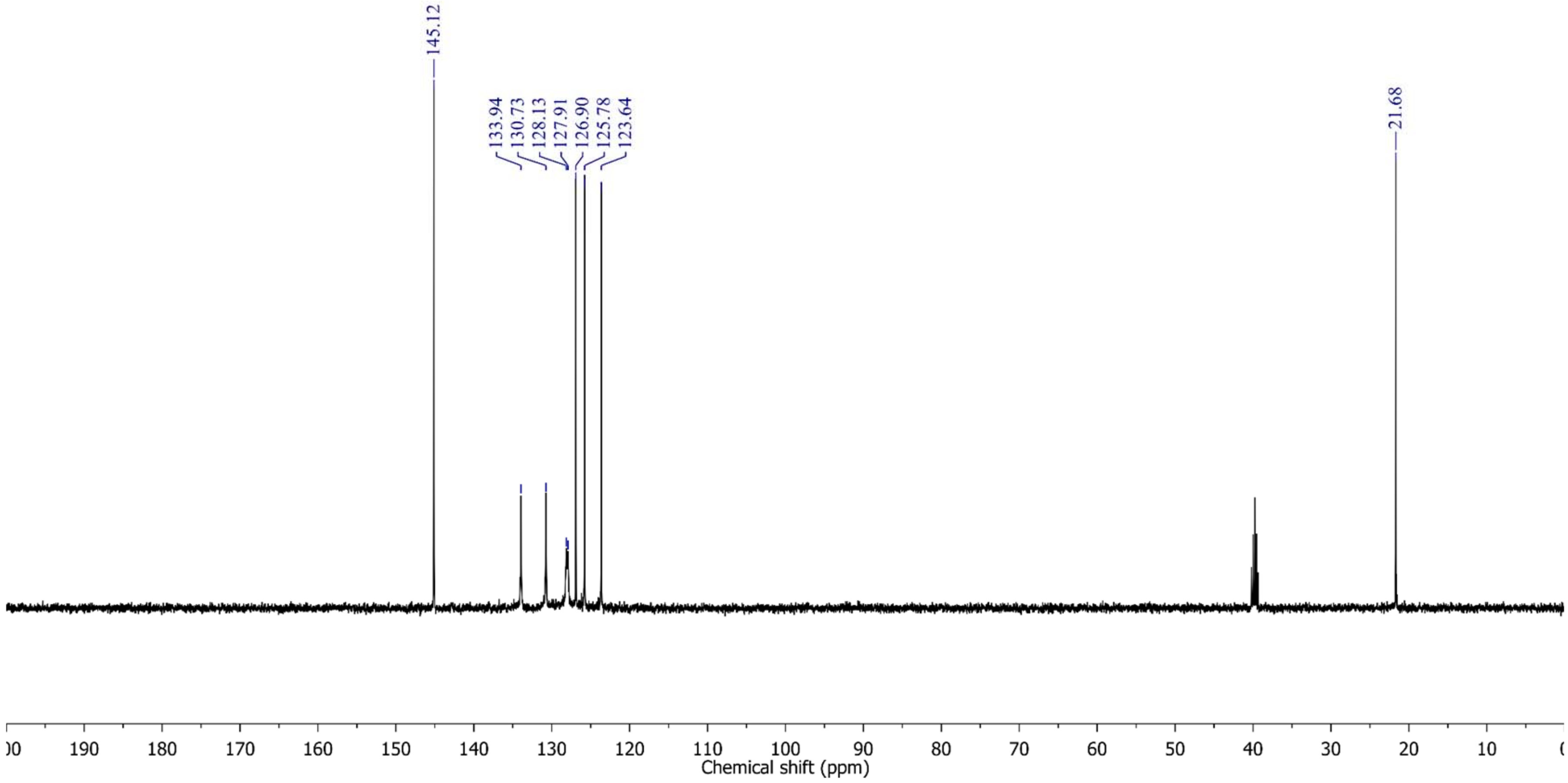

2-Nitroacridine-9-carbaldehyde oxime (1c), ^{1}H NMR, DMSO-d_6, 400 MHz

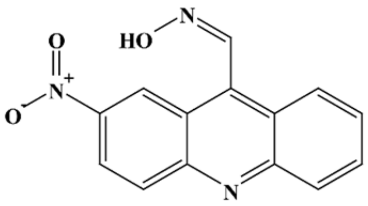

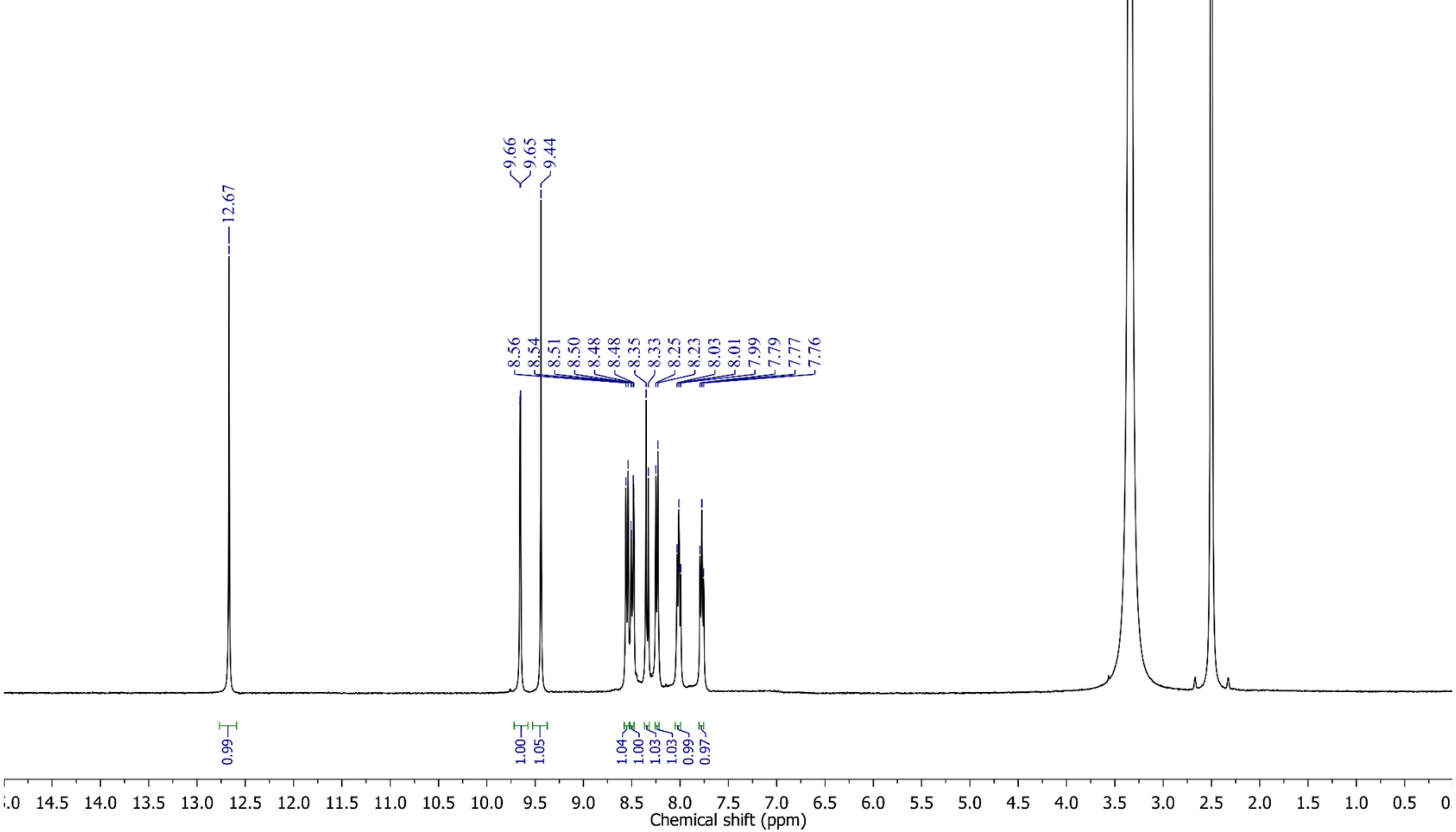

2-Nitroacridine-9-carbaldehyde oxime (1c), $^{13}C\{^{1}H\}$ NMR, DMSO-d_6, 100 MHz

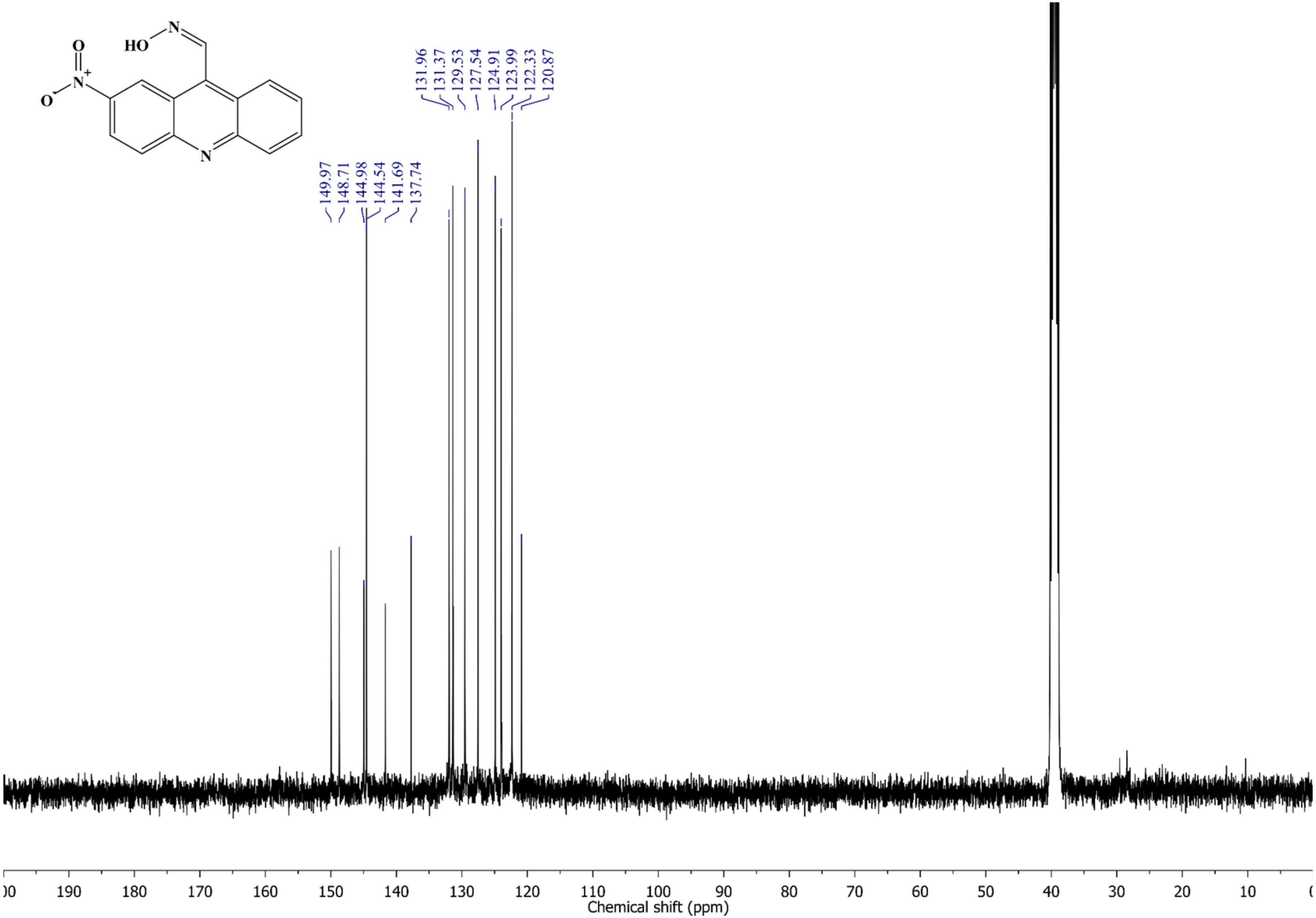

2-Nitroacridine-9-carbaldehyde oxime (1c), DEPT, DMSO-d_6, 100 MHz

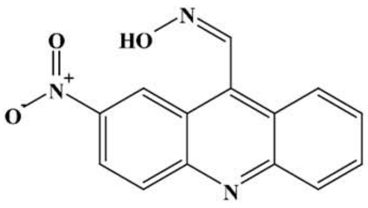

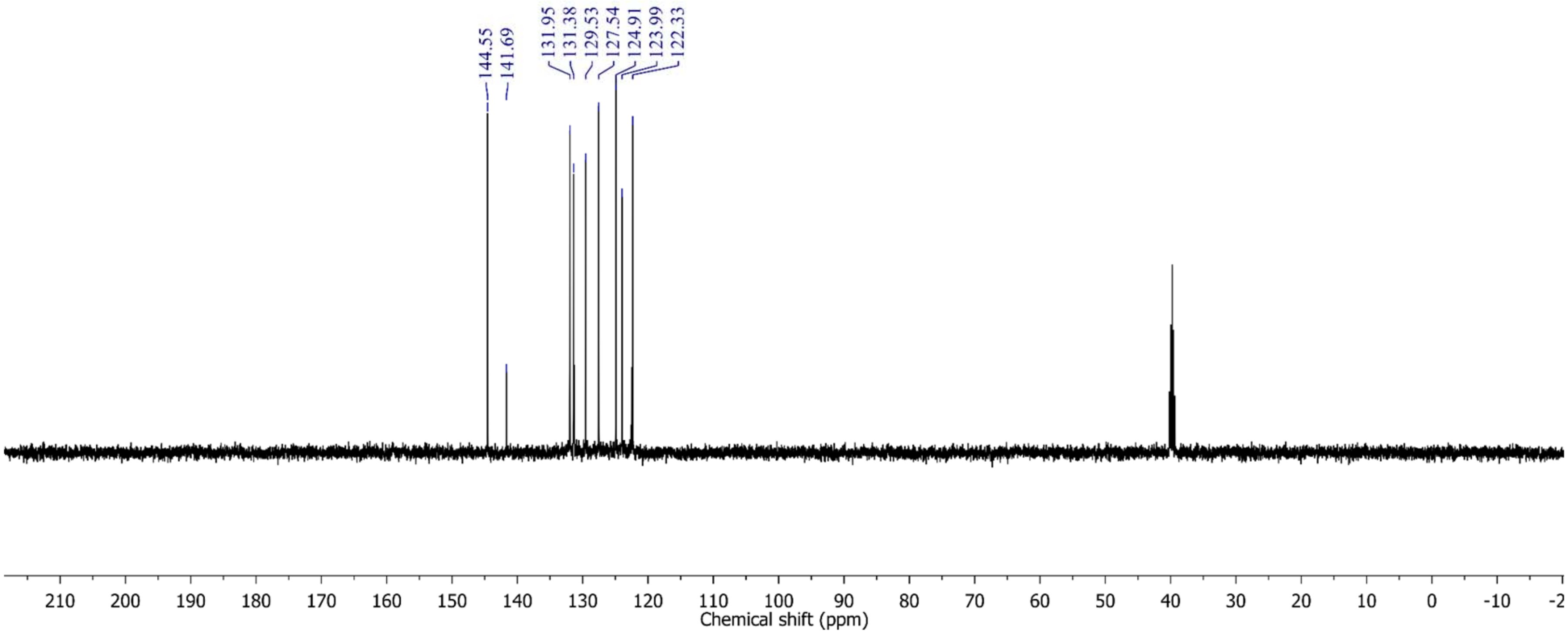

9-Methylbenzo[*c*]acridine-7-carbaldehyde oxime (1d), ^{1}H NMR, DMSO-d$_6$, 400 MHz

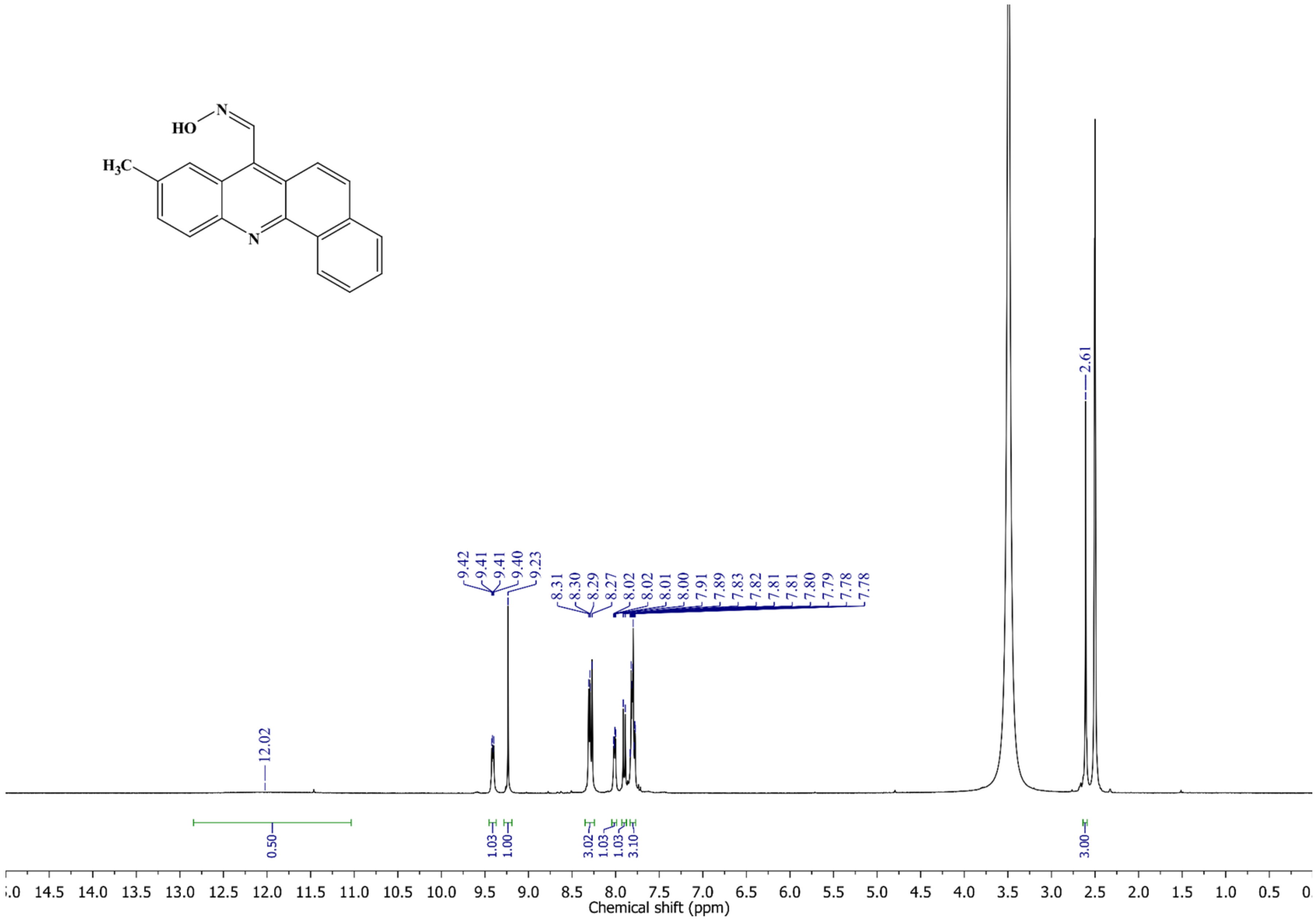

9-Methylbenzo[*c*]acridine-7-carbaldehyde oxime (1d), $^{13}C\{^{1}H\}$ NMR, DMSO-d_6, 100 MHz

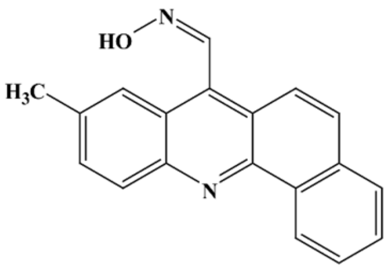

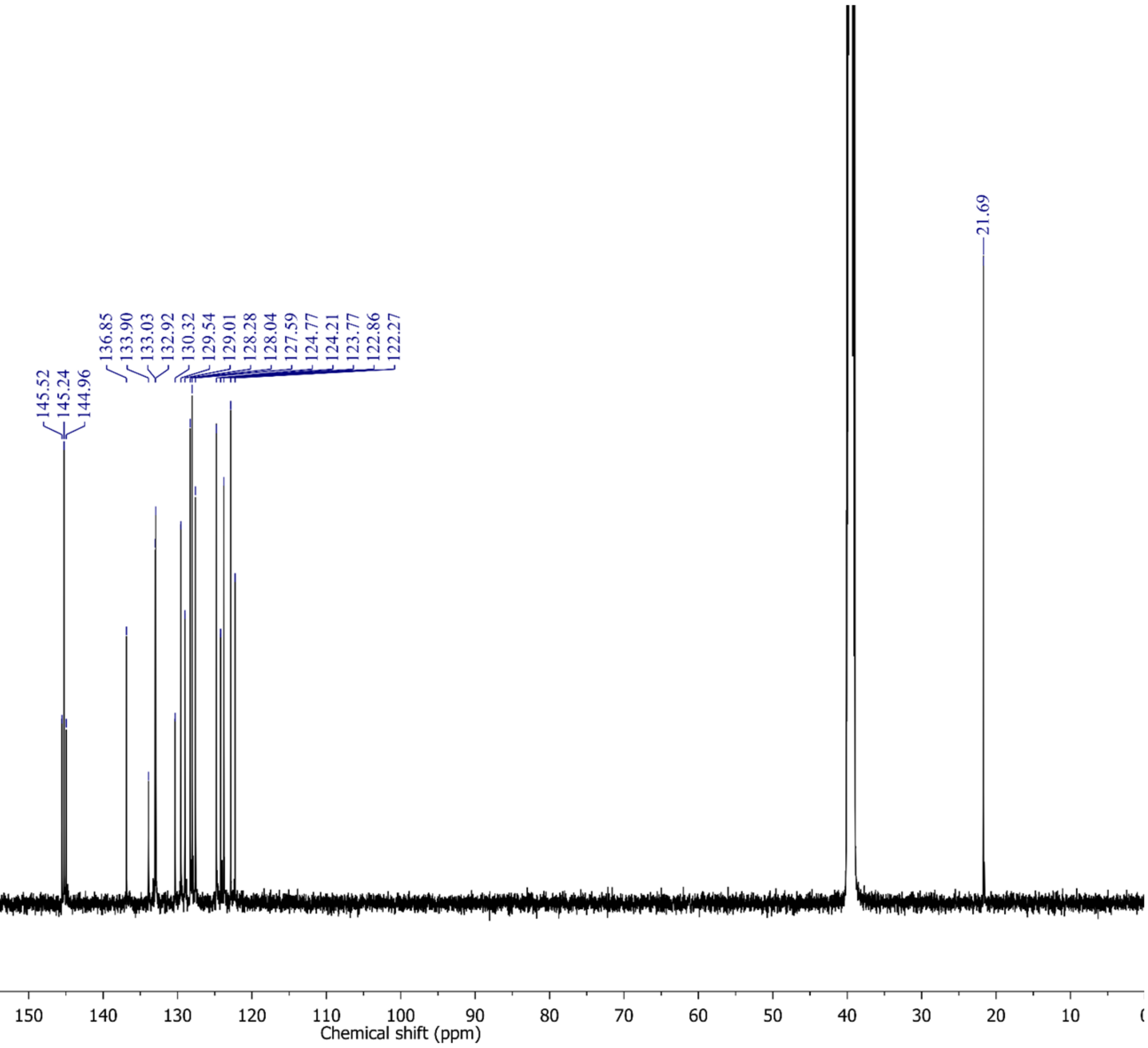

9-Methylbenzo[*c*]acridine-7-carbaldehyde oxime (1d), DEPT, DMSO-d$_6$, 100 MHz

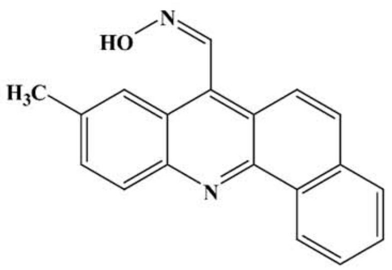

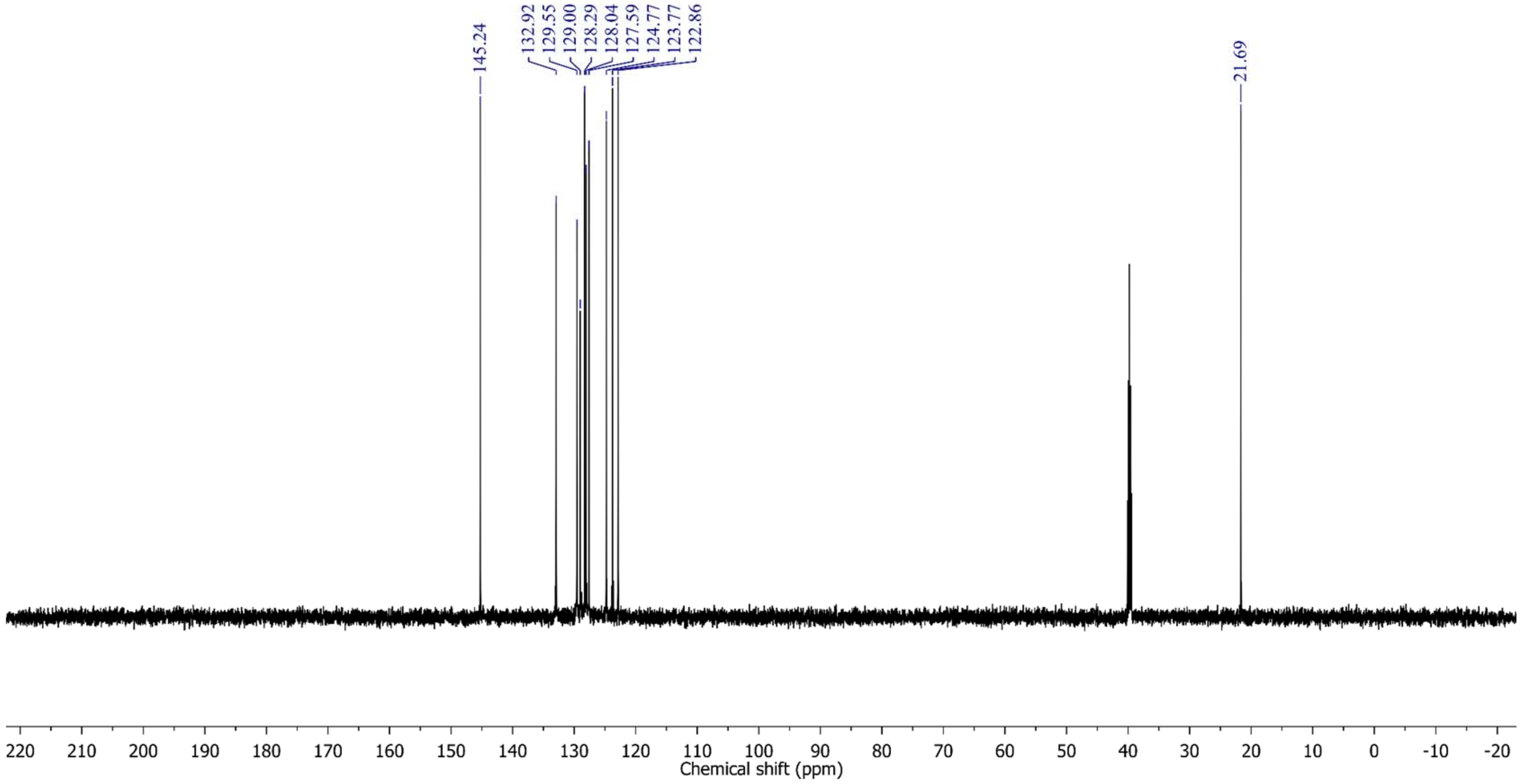

9-Phenylacridine-2-carbaldehyde oxime (1e), ^{1}H NMR, DMSO-d$_6$, 400 MHz

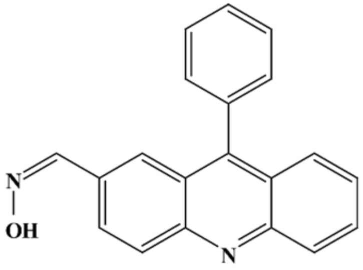

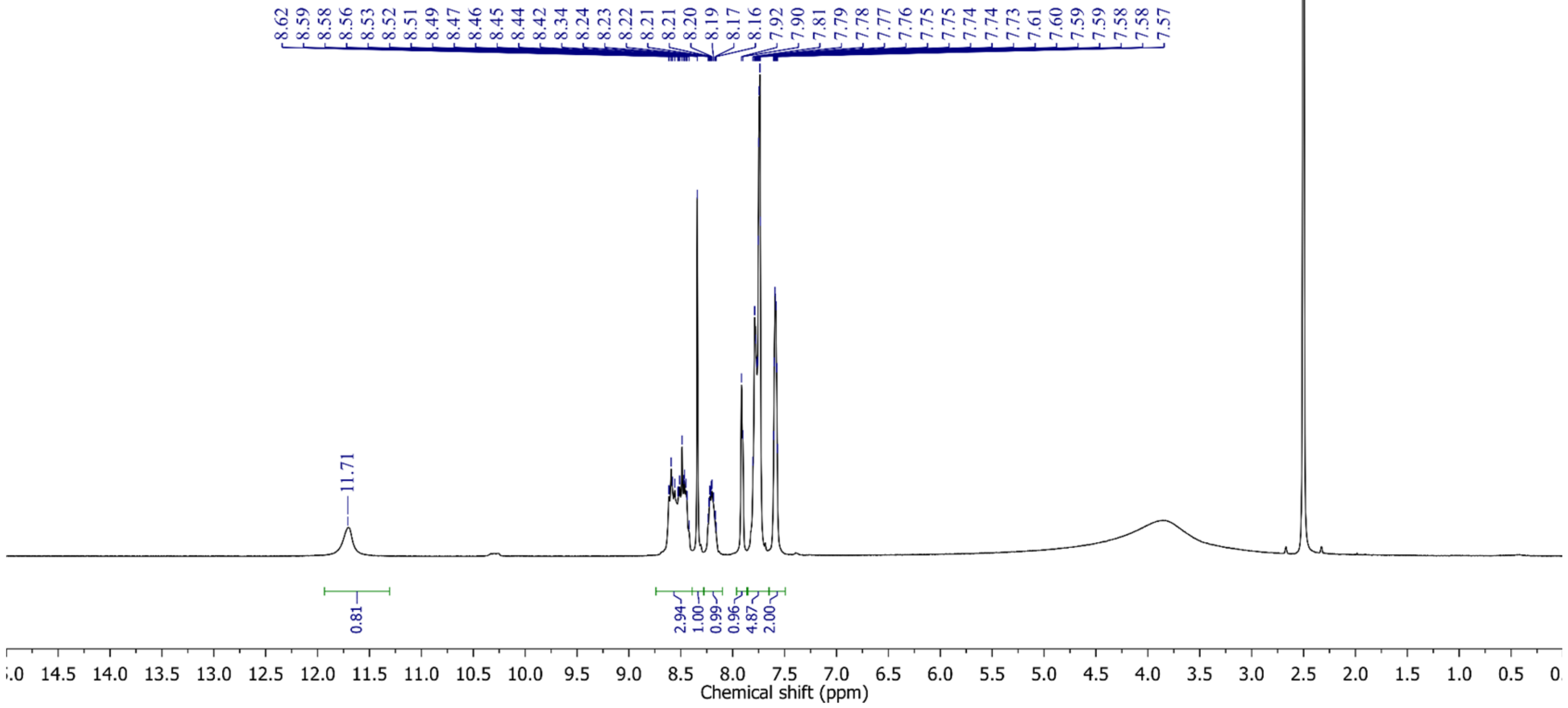

9-Phenylacridine-2-carbaldehyde oxime (1e), $^{13}C\{^1H\}$ NMR, DMSO-d_6, 100 MHz

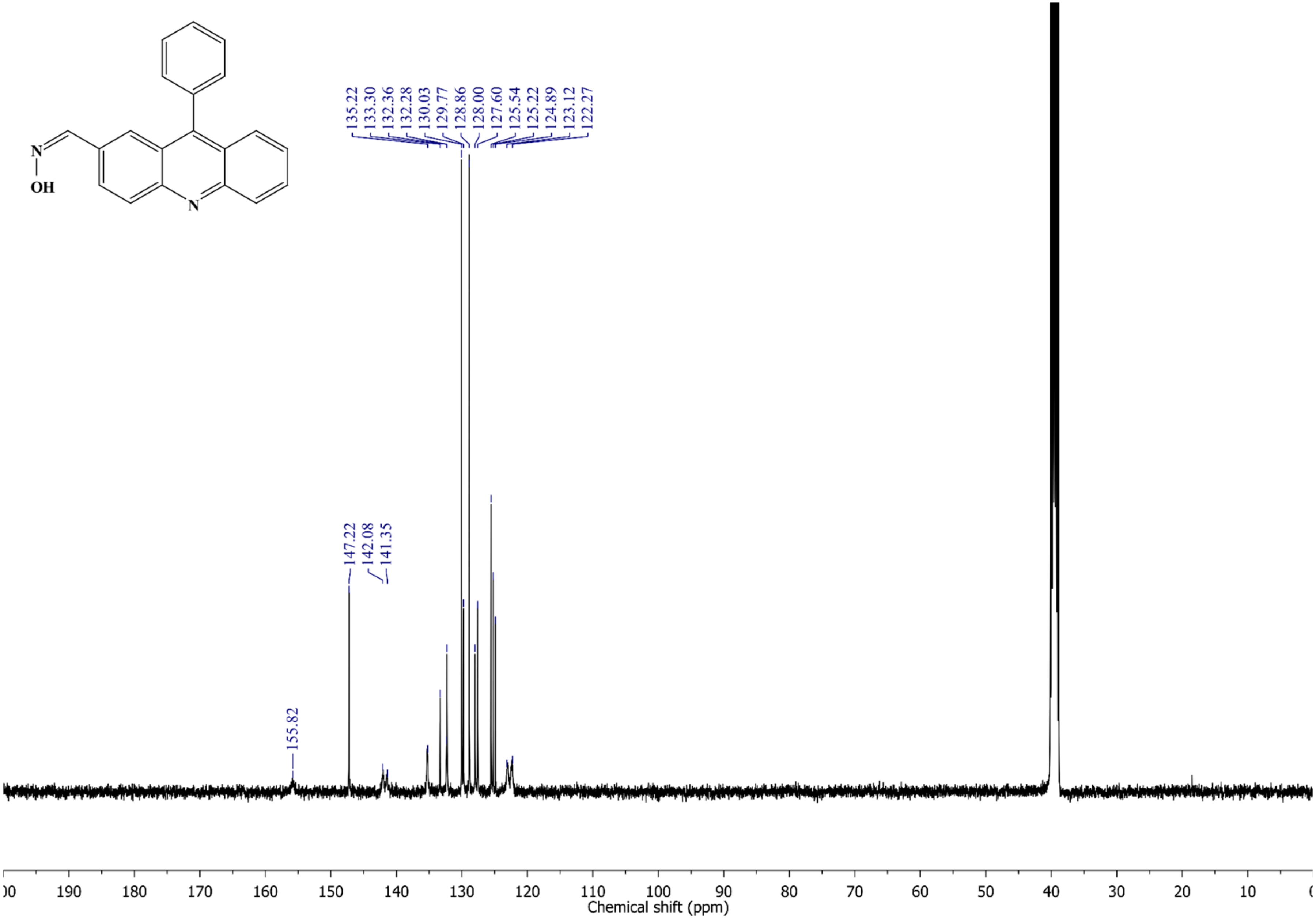

9-Phenylacridine-2-carbaldehyde oxime (1e), DEPT, DMSO-d_6, 100 MHz

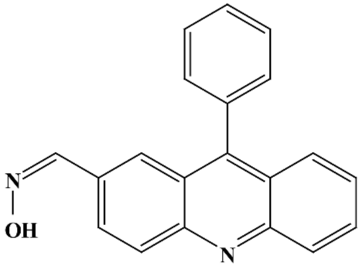

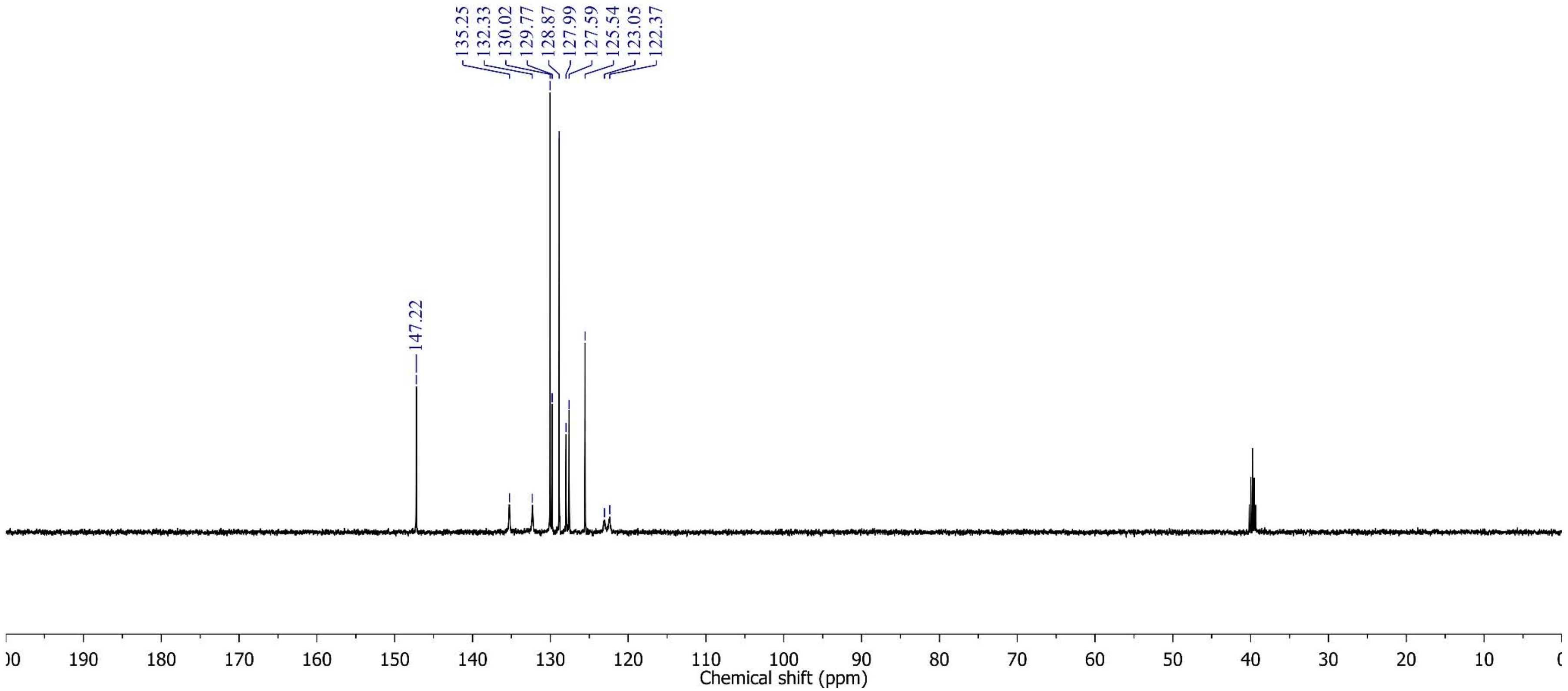

N-Hydroxyacridine-9-carbimidoyl chloride hydrochloride (2a), ^{1}H NMR, DMSO-d$_6$, 400 MHz

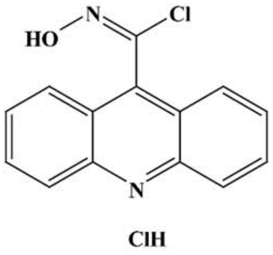

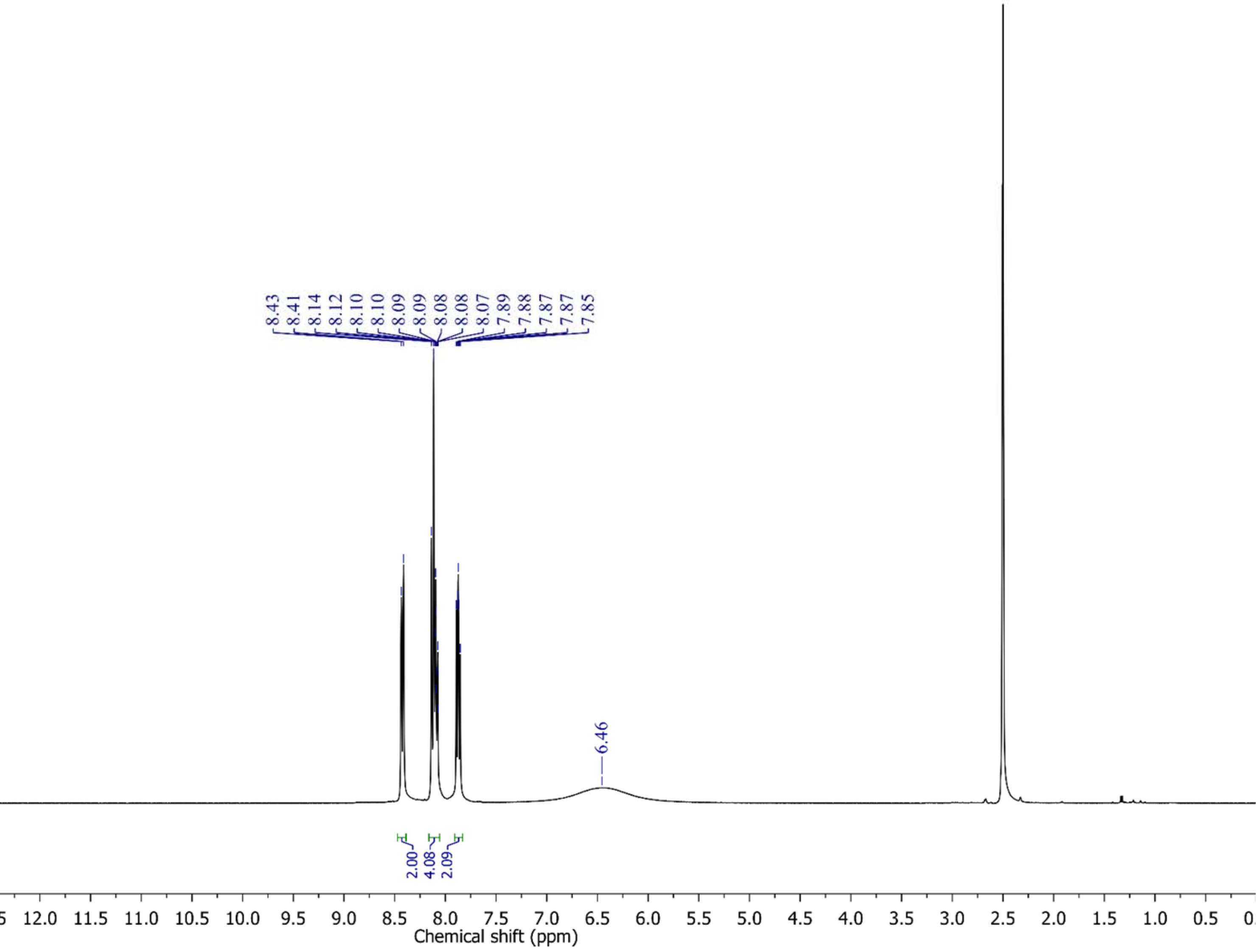

N-Hydroxyacridine-9-carbimidoyl chloride hydrochloride (2a), $^{13}C\{^1H\}$ NMR, DMSO-d_6, 100 MHz

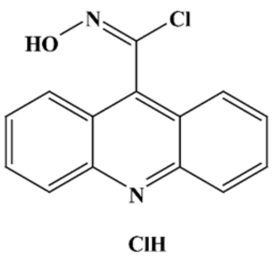

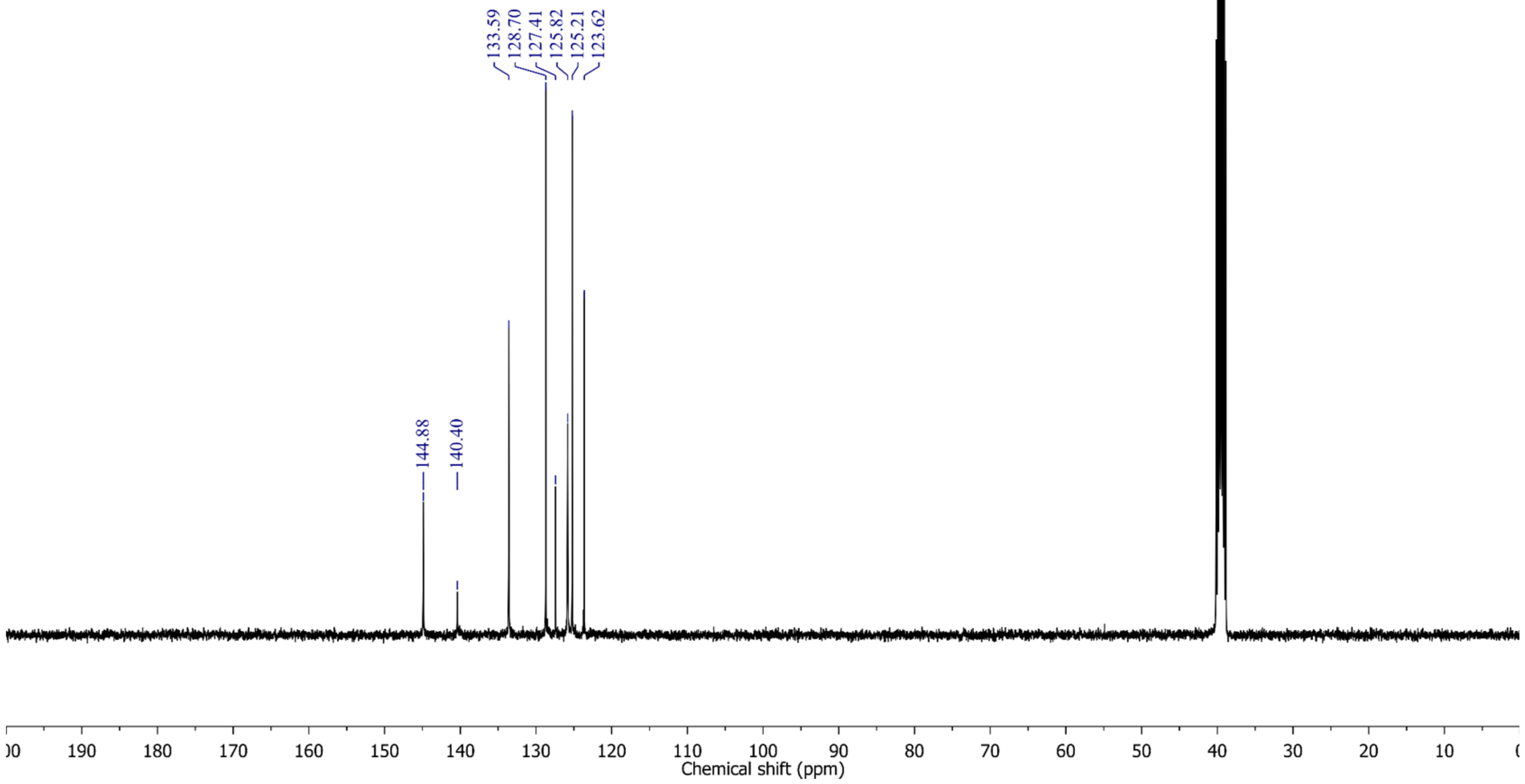

N-Hydroxyacridine-9-carbimidoyl chloride hydrochloride (2a), DEPT, DMSO-d_6, 100 MHz

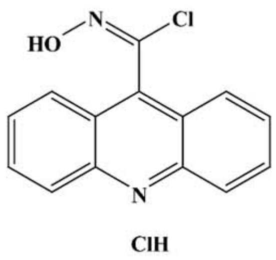

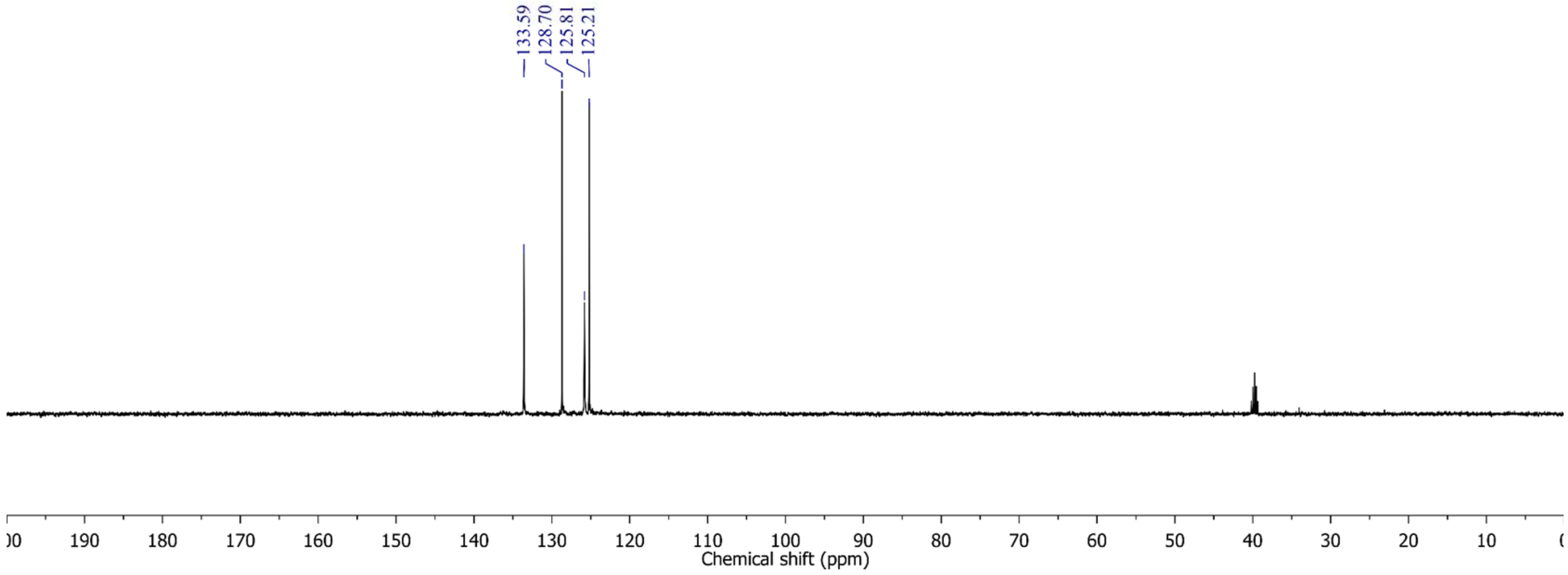

N-Hydroxy-2-methylacridine-9-carbimidoyl chloride hydrochloride (2b), ^{1}H NMR, DMSO-d$_6$, 400 MHz

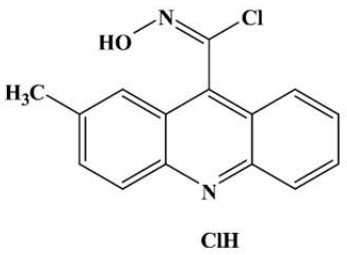

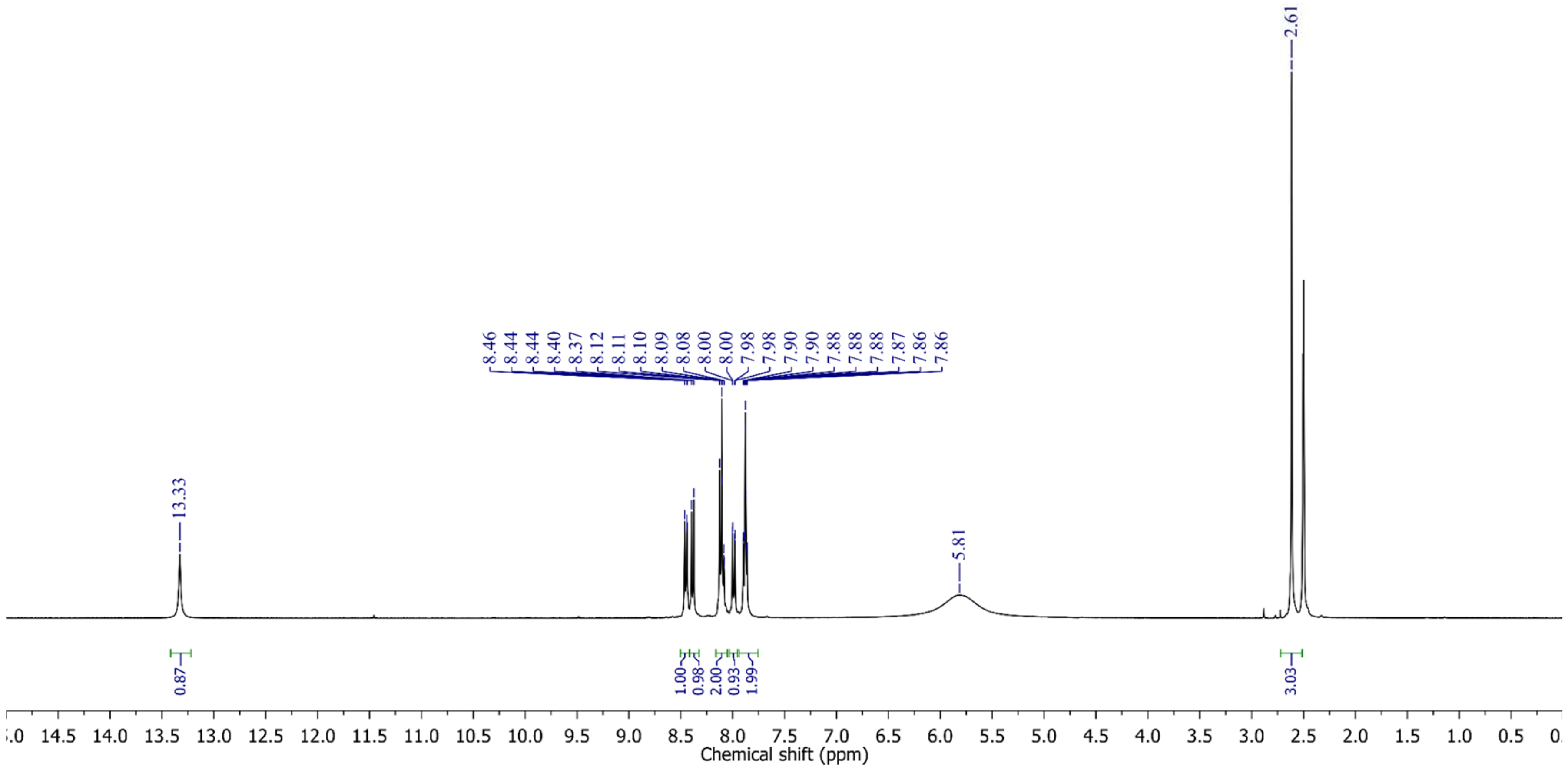

***N*-Hydroxy-2-methylacridine-9-carbimidoyl chloride hydrochloride (2b), $^{13}C\{^{1}H\}$ NMR, DMSO-d_6, 100 MHz**

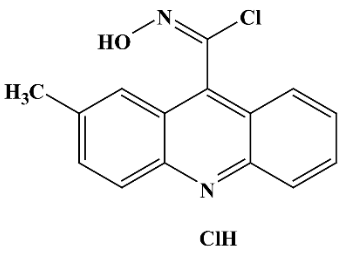

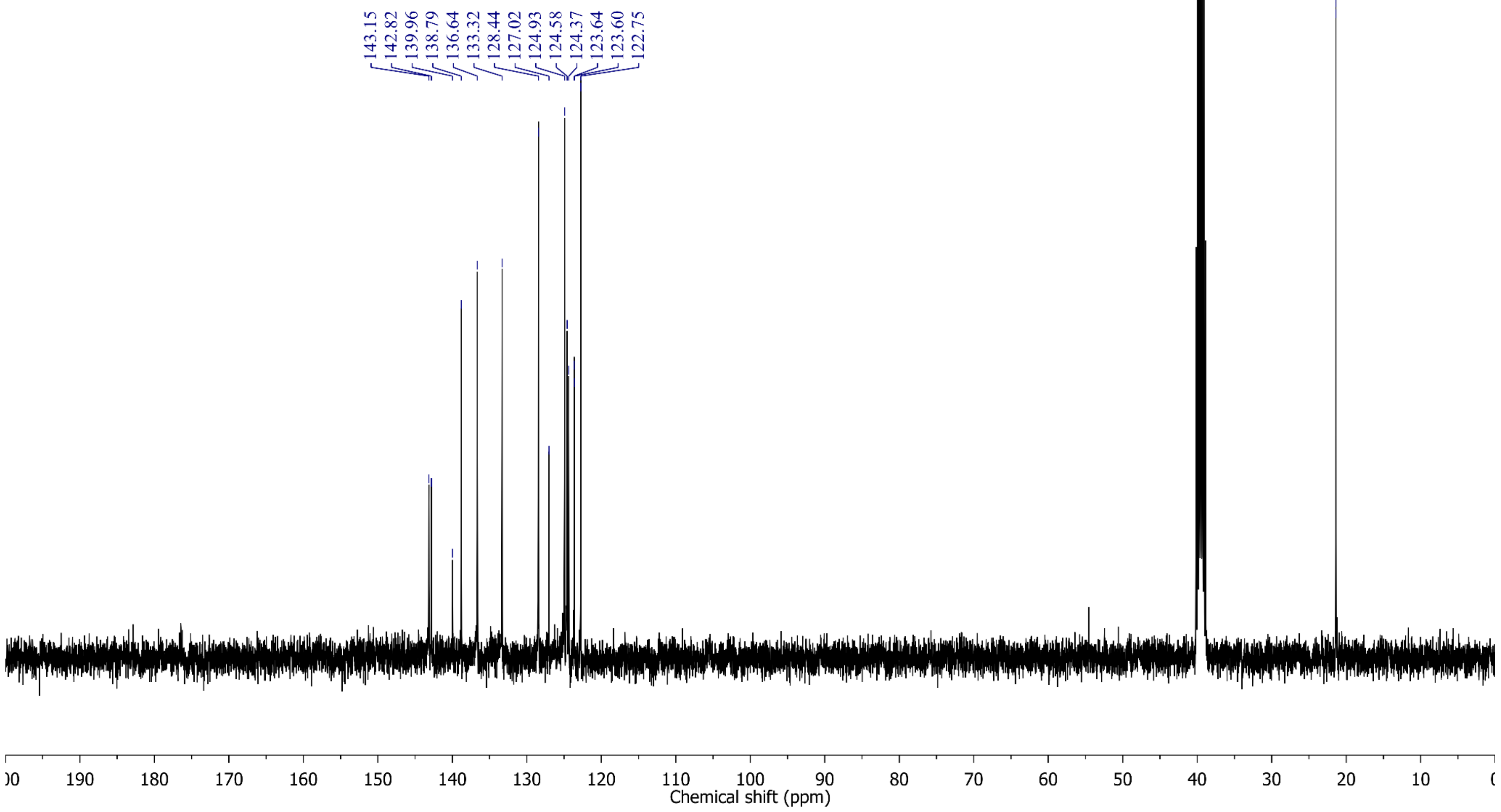

_N_-Hydroxy-2-methylacridine-9-carbimidoyl chloride hydrochloride (2b), DEPT, DMSO-d_6, 100 MHz

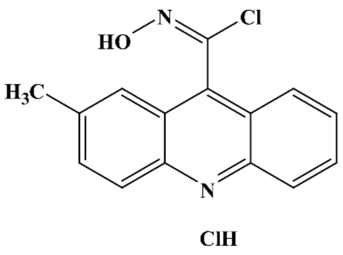

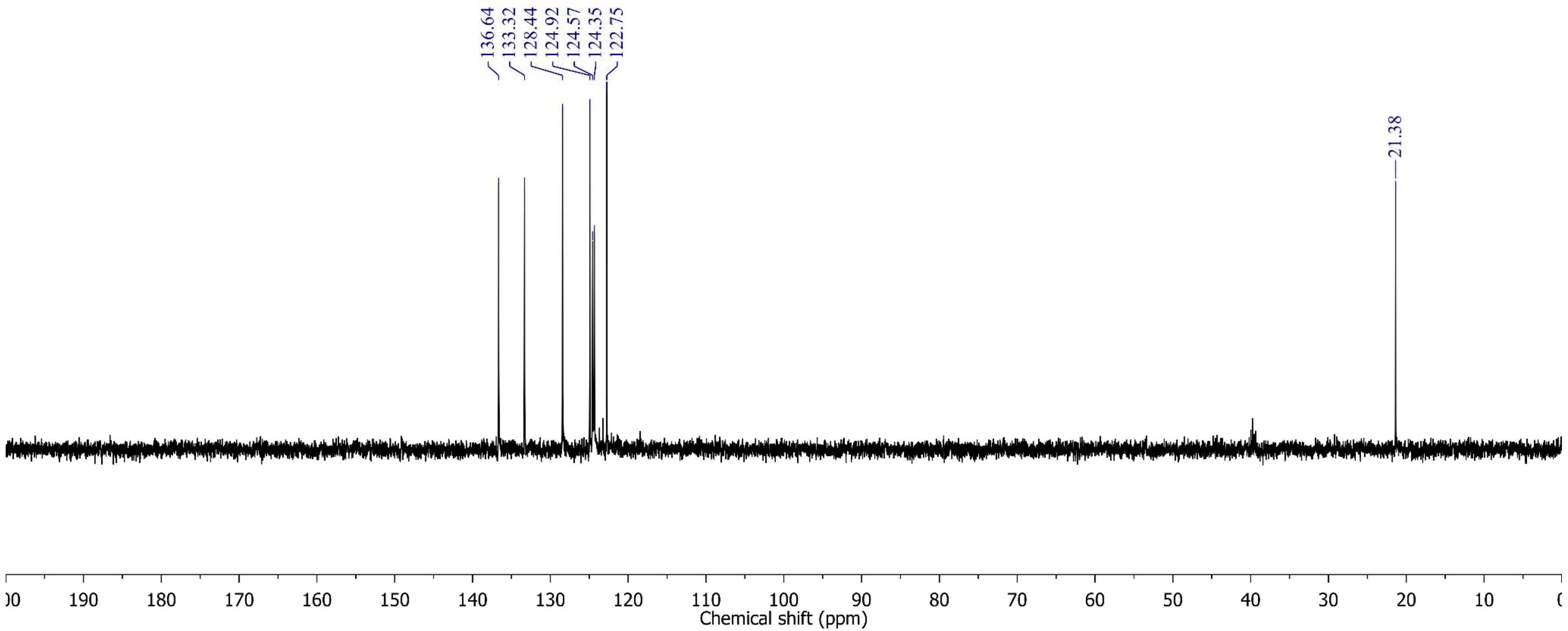

***N*-Nydroxy-2-nitroacridine-9-carbimidoyl chloride hydrochloride (2c), ^{1}H NMR, DMSO-d$_6$, 400 MHz**

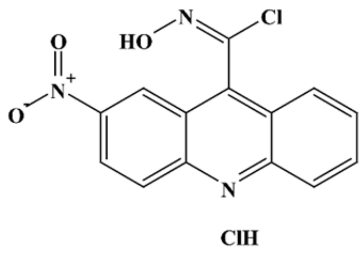

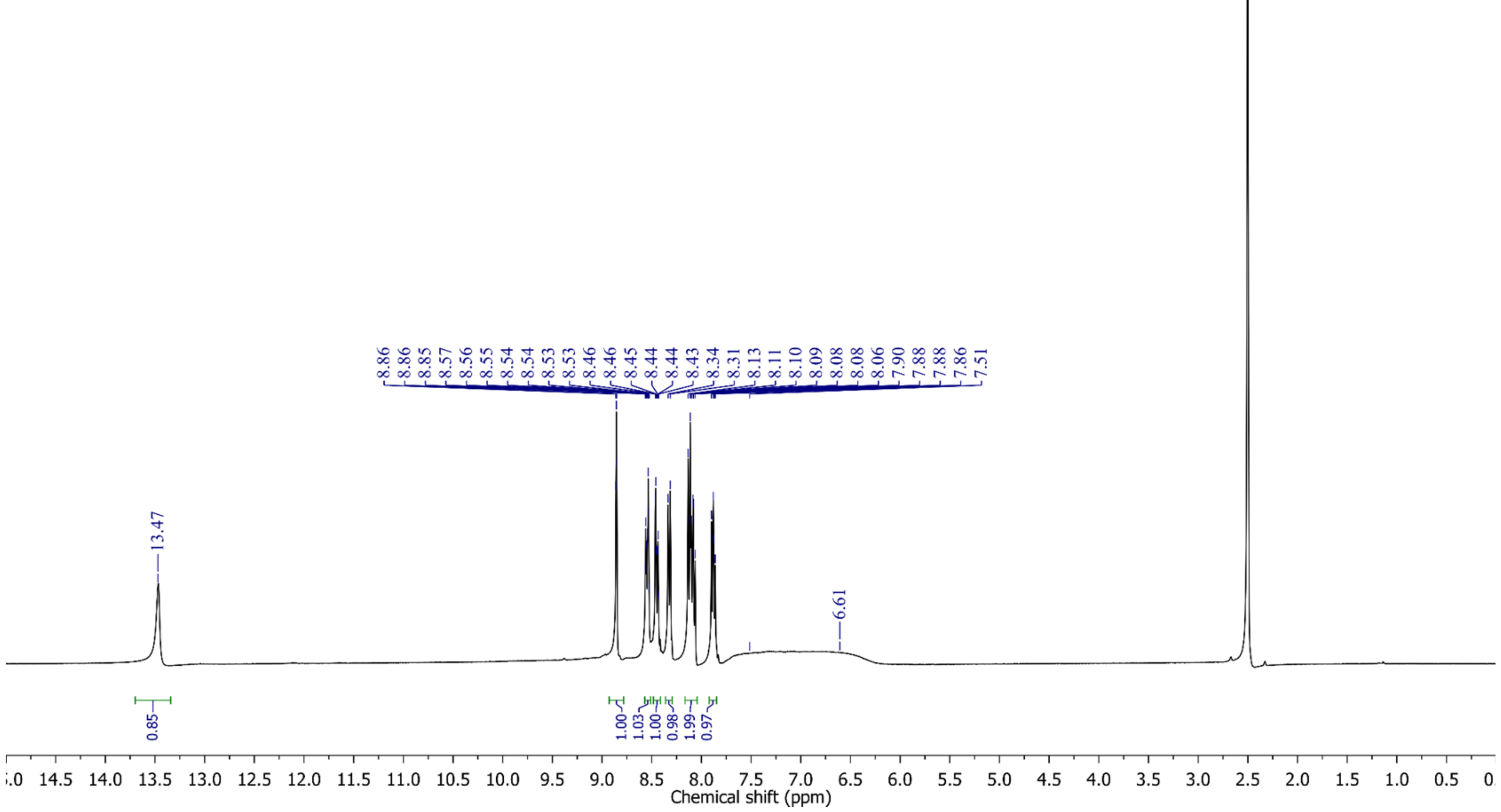

***N*-Nydroxy-2-nitroacridine-9-carbimidoyl chloride hydrochloride (2c), $^{13}C\{^1H\}$ NMR, DMSO-d_6, 100 MHz**

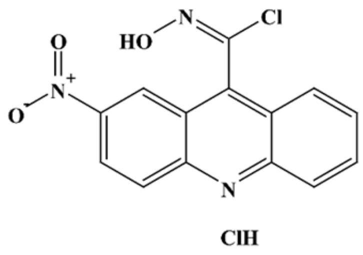

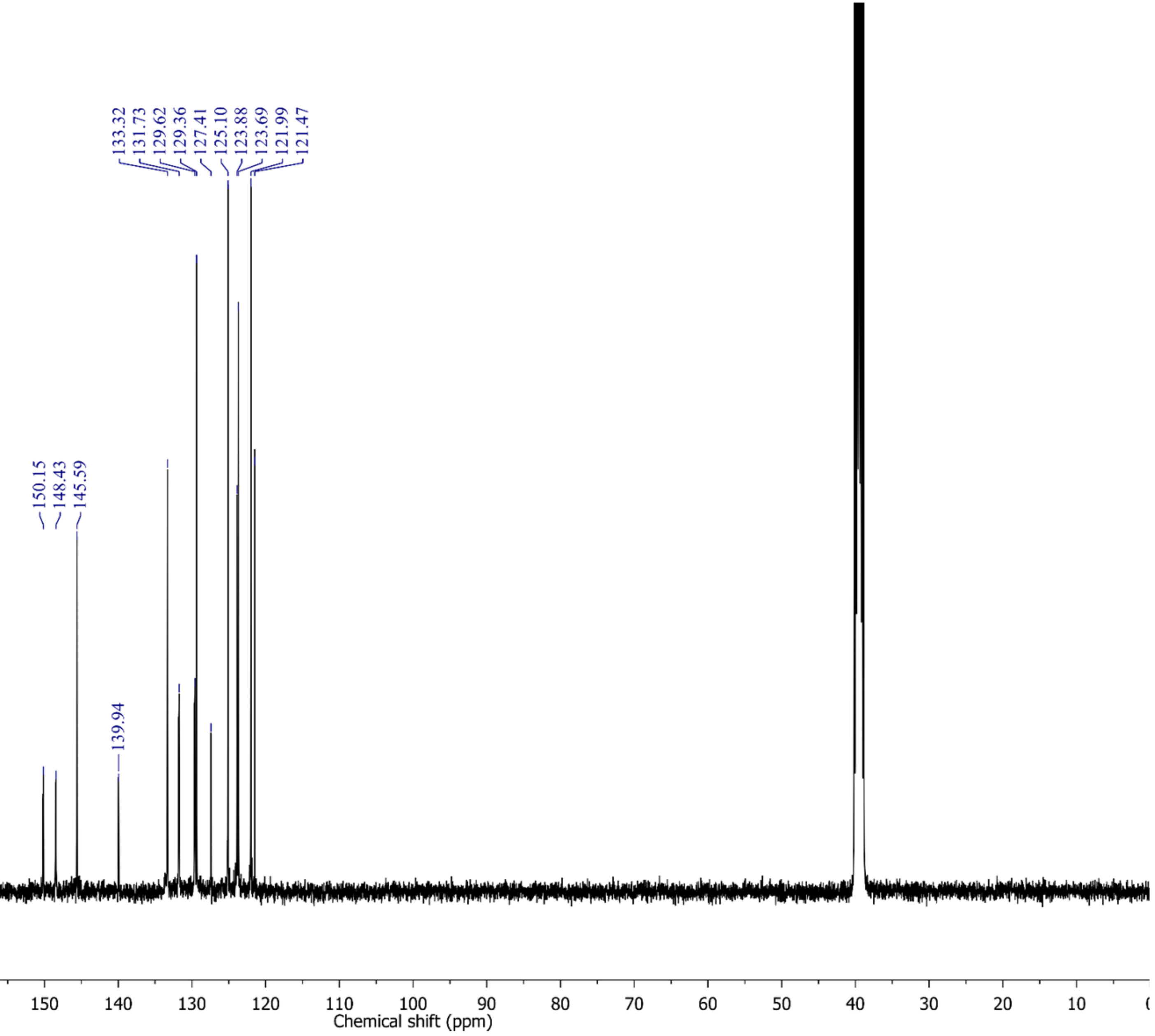

***N*-Nydroxy-2-nitroacridine-9-carbimidoyl chloride hydrochloride (2c), DEPT, DMSO-d_6, 100 MHz**

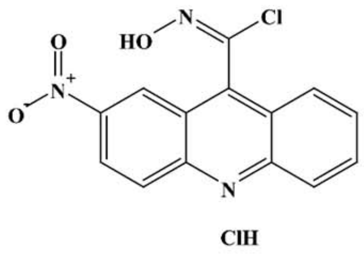

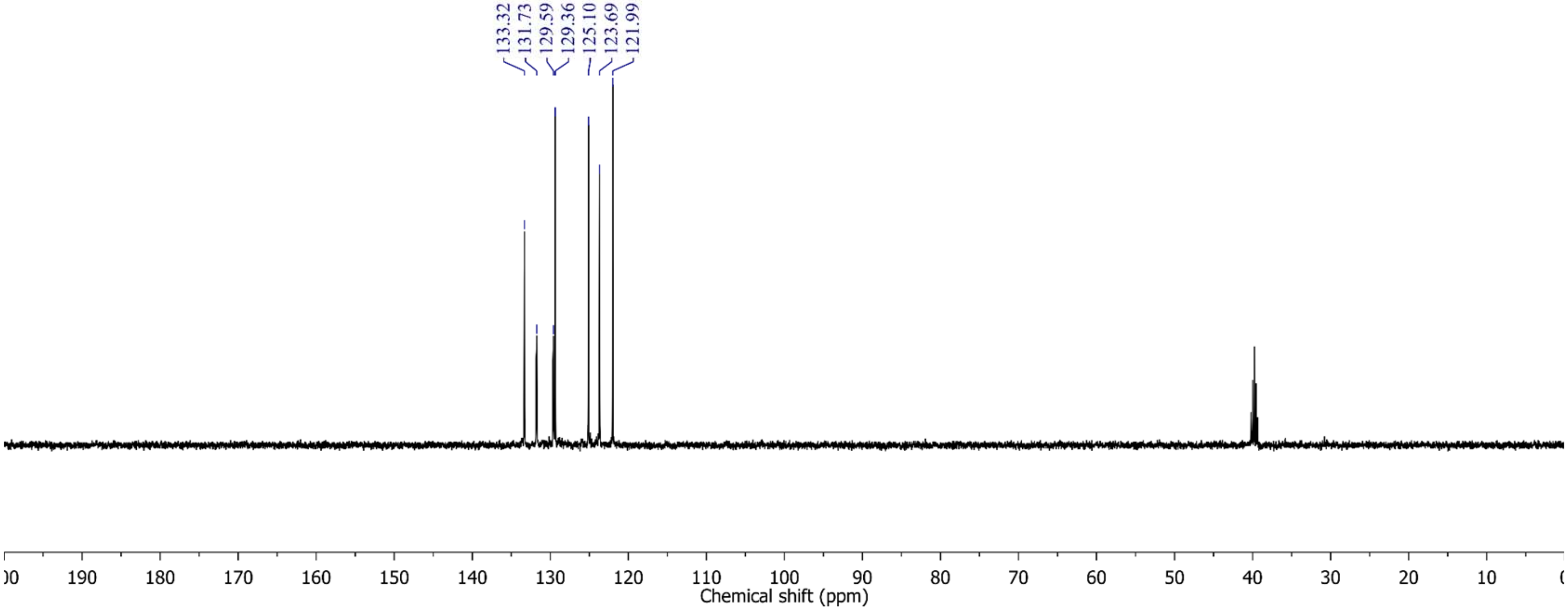

***N*-Hydroxy-9-methylbenzo[*c*]acridine-7-carbimidoyl chloride hydrochloride (2d), ^{1}H NMR, DMSO-d$_6$, 400 MHz**

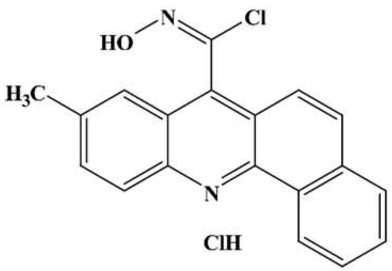

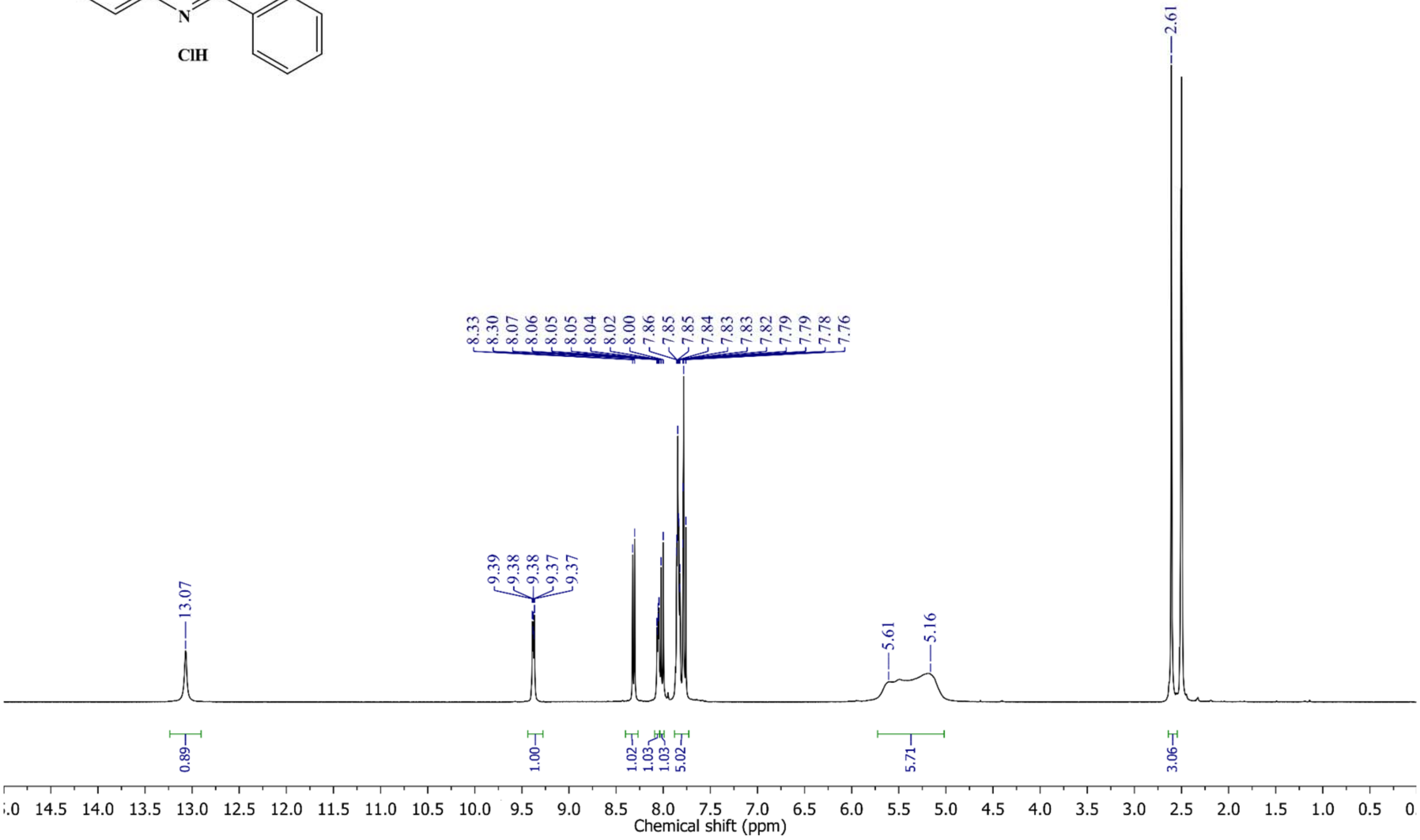

***N*-Hydroxy-9-methylbenzo[*c*]acridine-7-carbimidoyl chloride hydrochloride (2d), $^{13}C\{^1H\}$ NMR, DMSO-d_6, 100 MHz**

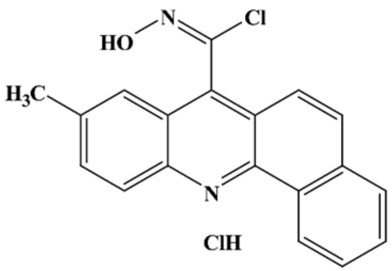

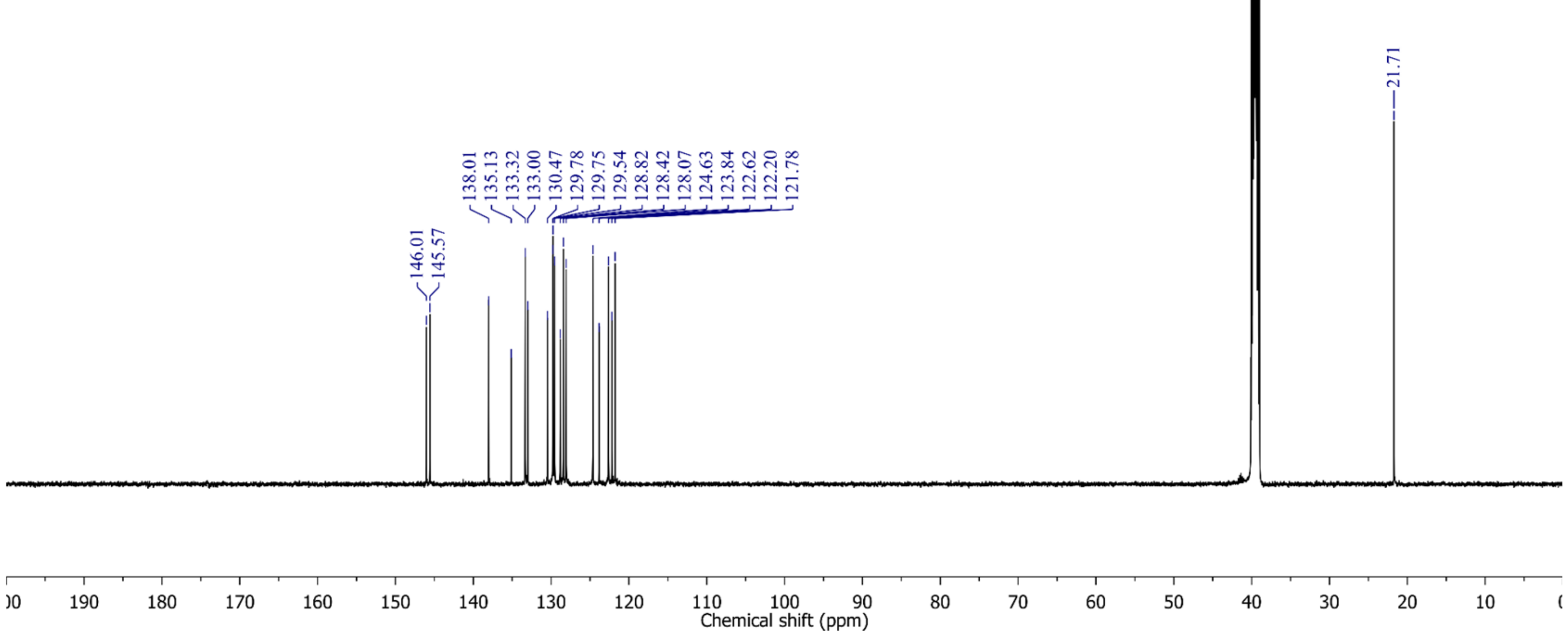

_N_-Hydroxy-9-methylbenzo[_c_]acridine-7-carbimidoyl chloride hydrochloride (2d), DEPT, DMSO-d_6, 100 MHz

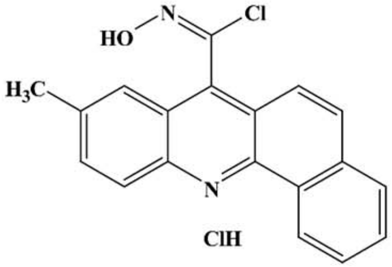

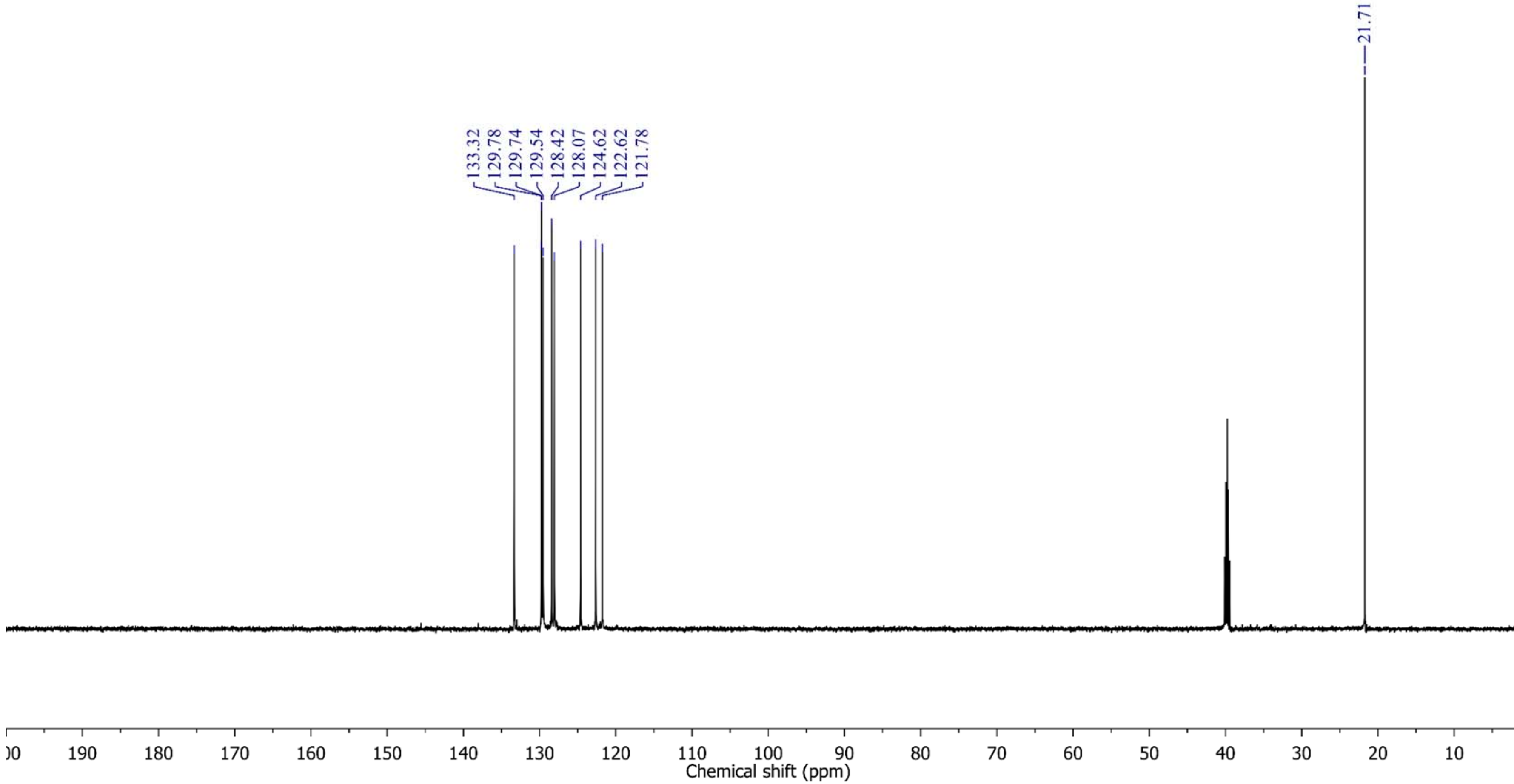

***N*-Hydroxy-9-phenylacridine-2-carbimidoyl chloride hydrochloride (2e), ^{1}H NMR, DMSO-d$_6$, 400 MHz**

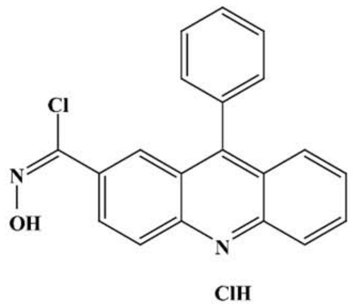

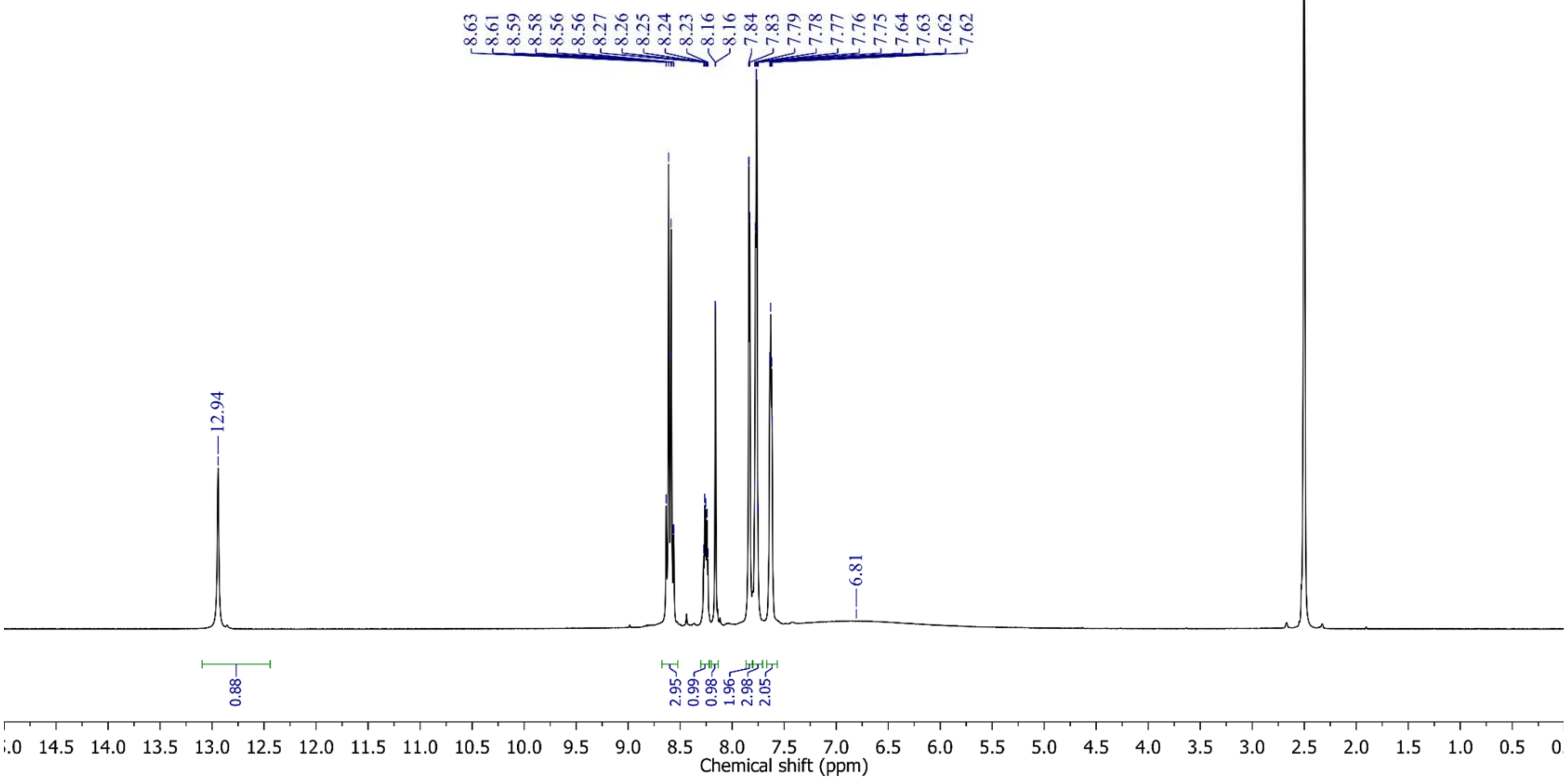

***N*-Hydroxy-9-phenylacridine-2-carbimidoyl chloride hydrochloride (2e), $^{13}C\{^1H\}$ NMR, DMSO-d_6, 100 MHz**

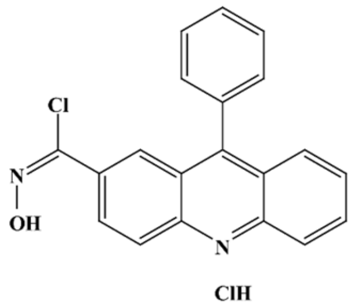

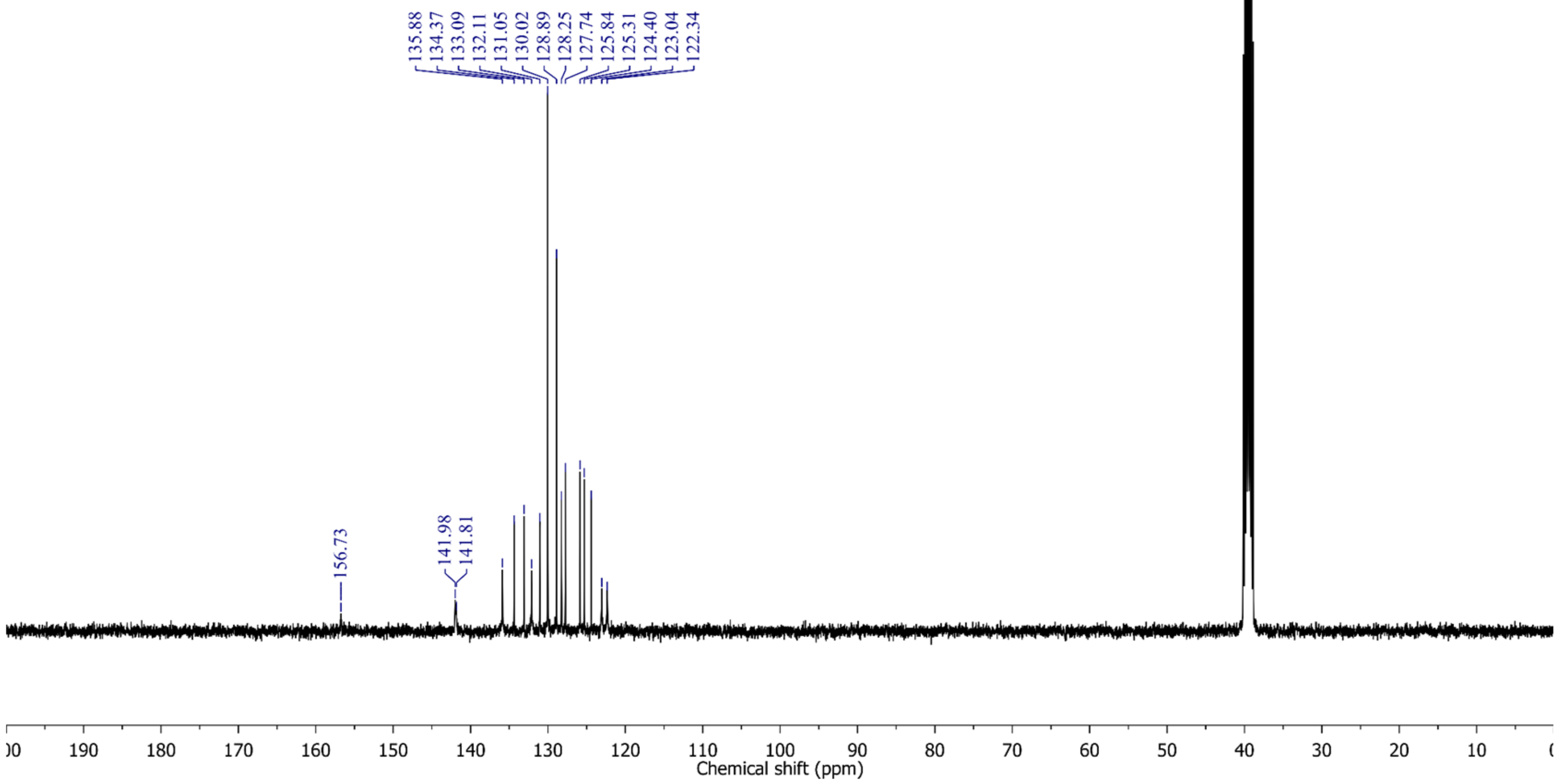

N-Hydroxy-9-phenylacridine-2-carbimidoyl chloride hydrochloride (2e), DEPT, DMSO-d$_6$, 100 MHz

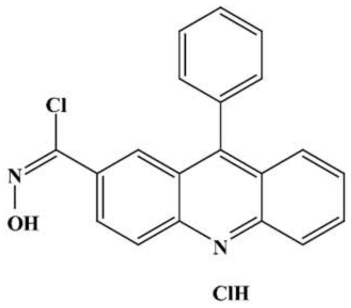

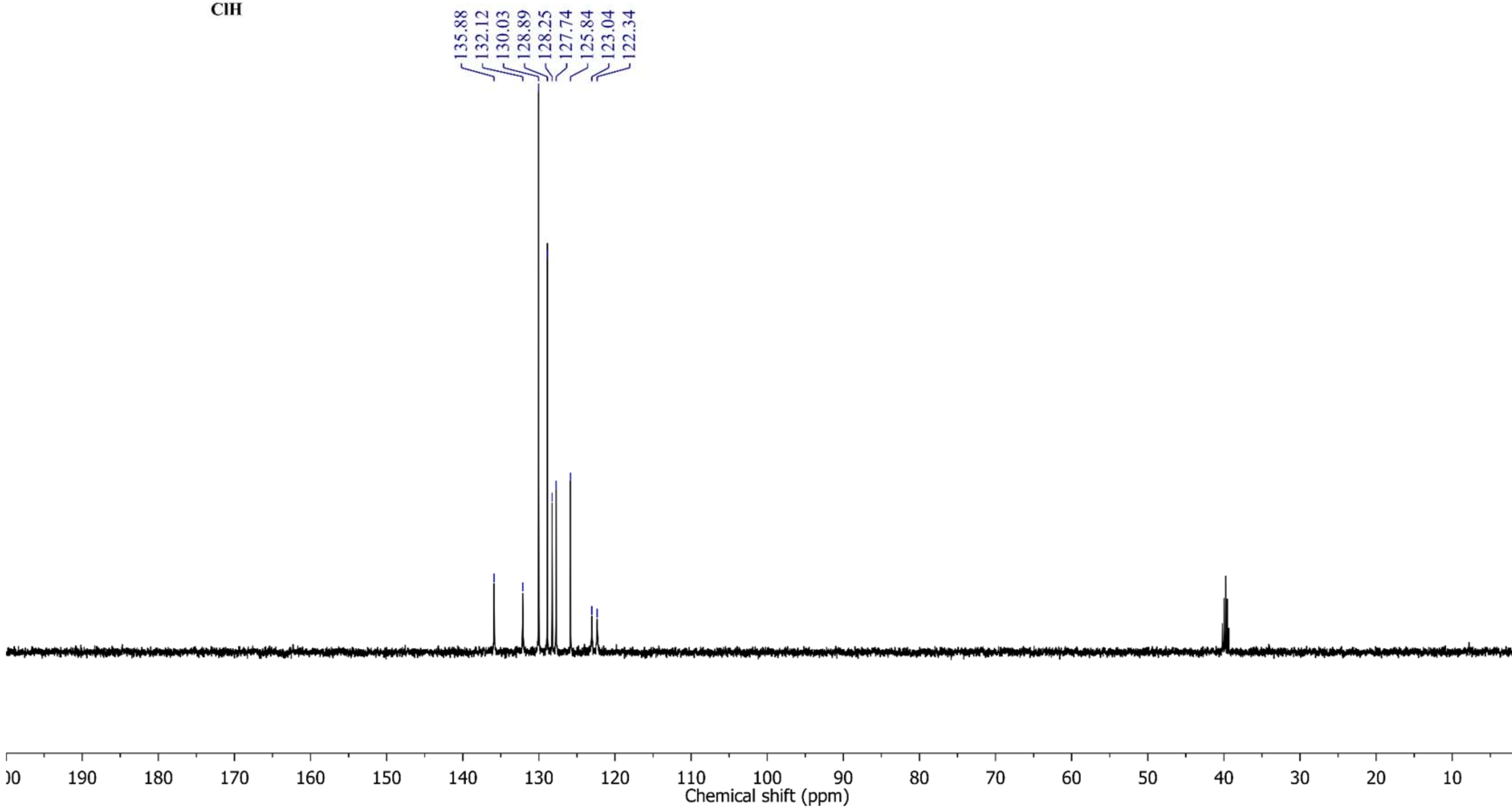

3-(Acridin-9-yl)-5-phenylisoxazole (7a), ^{1}H NMR, $CDCl_3$, 400 MHz

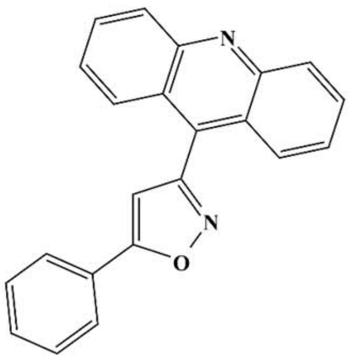

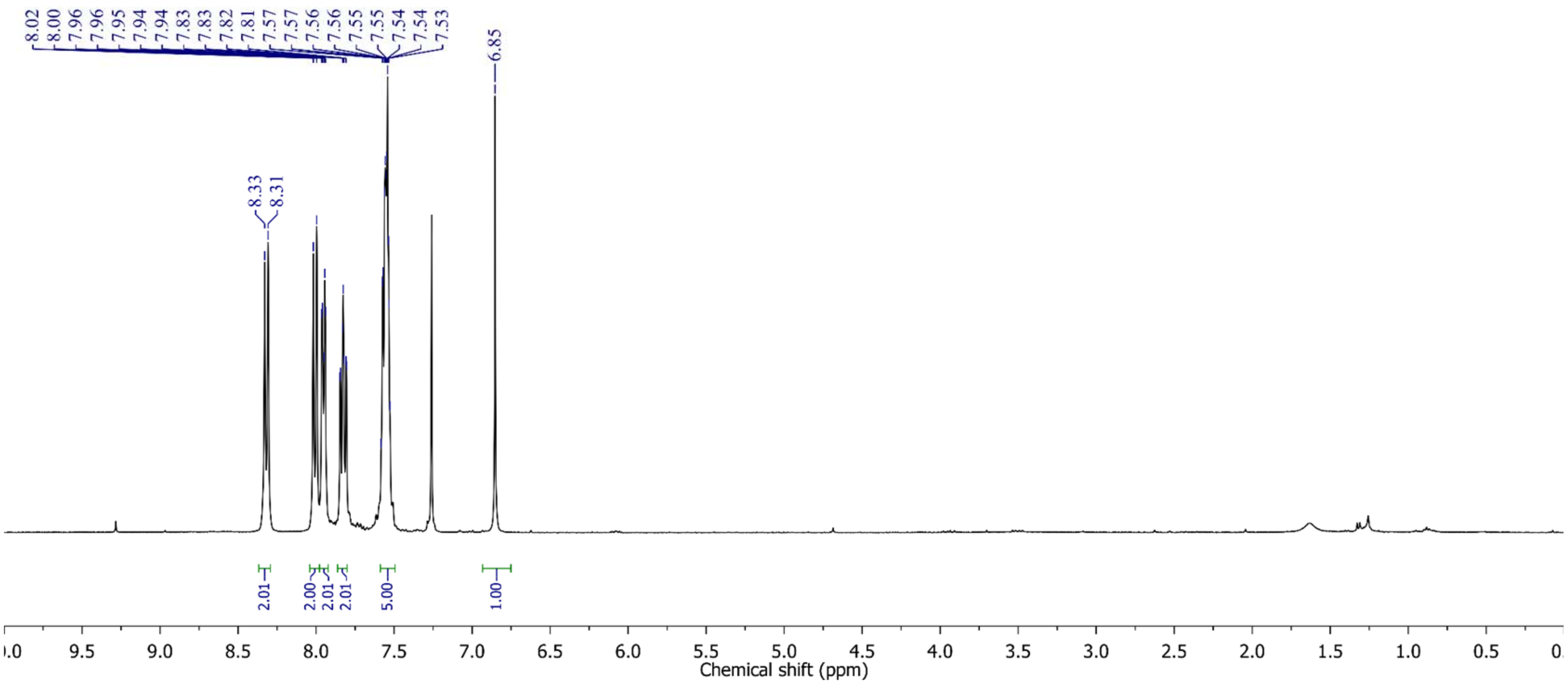

3-(Acridin-9-yl)-5-phenylisoxazole (7a), $^{13}C\{^1H\}$ NMR, $CDCl_3$, 100 MHz

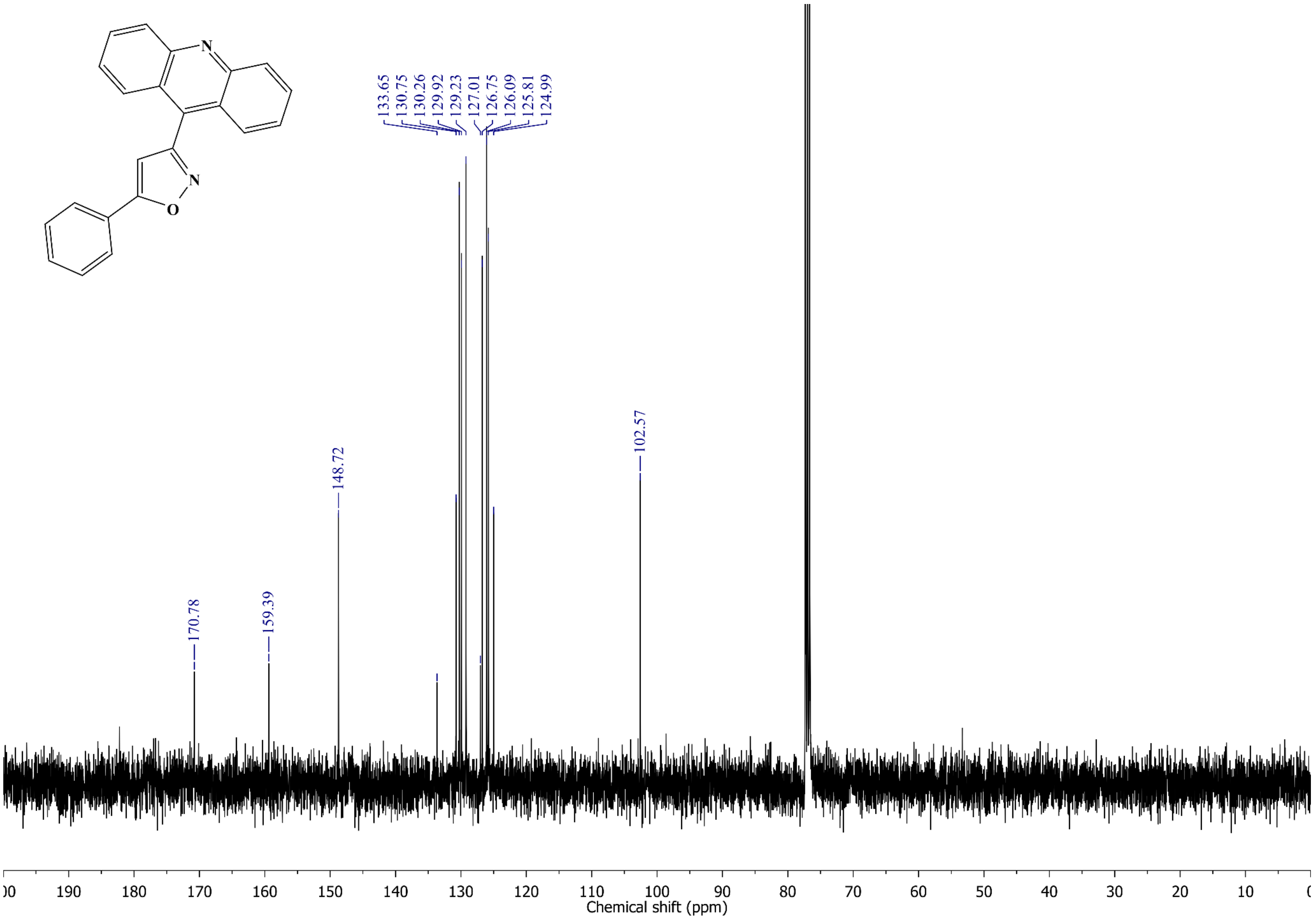

3-(Acridin-9-yl)-5-phenylisoxazole (7a), DEPT, $CDCl_3$, 100 MHz

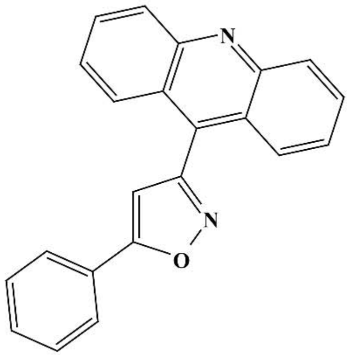

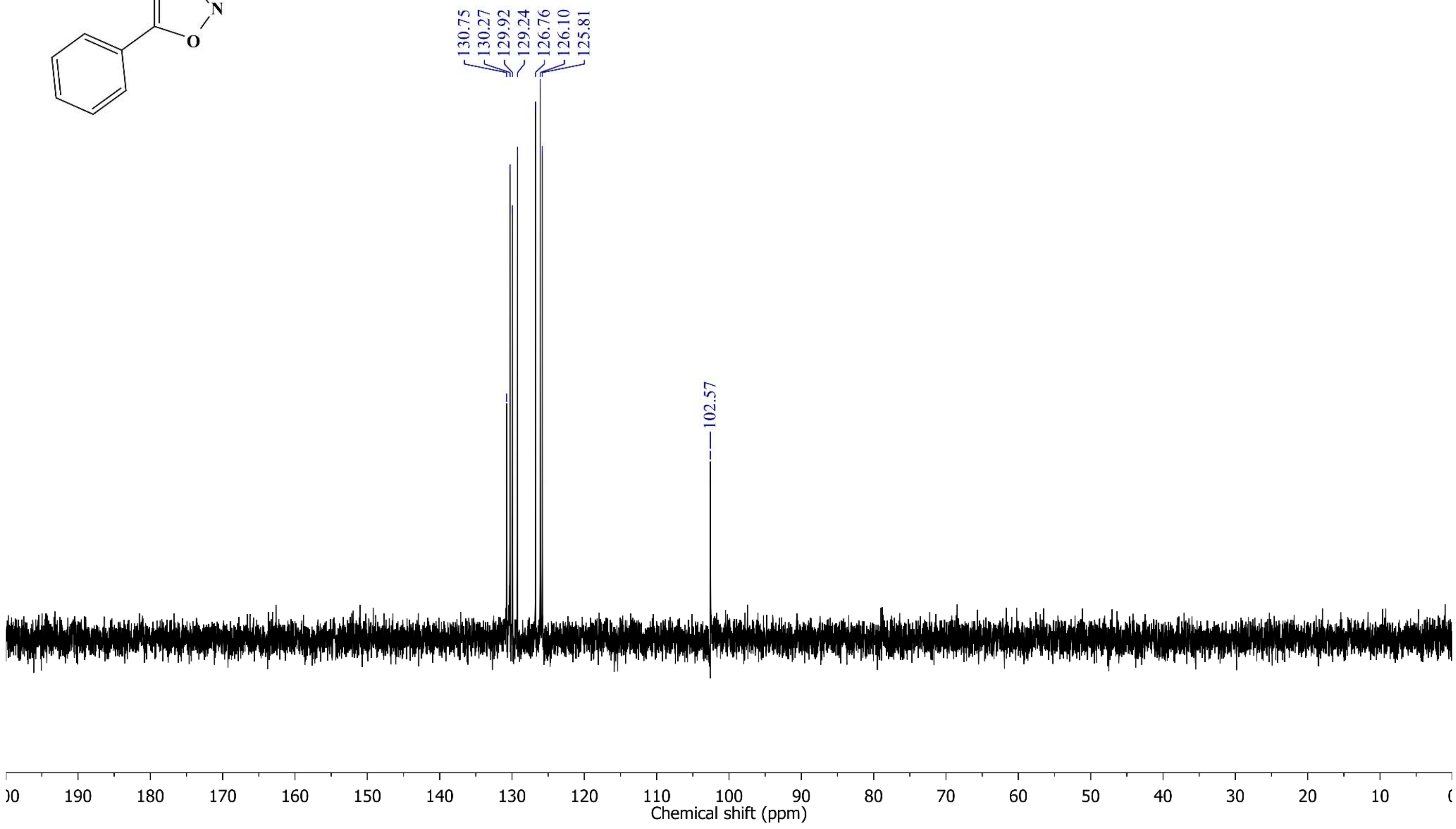

3-(Acridin-9-yl)-5-(trimethylsilyl)isoxazole (7b), ^{1}H NMR, $CDCl_3$, 400 MHz

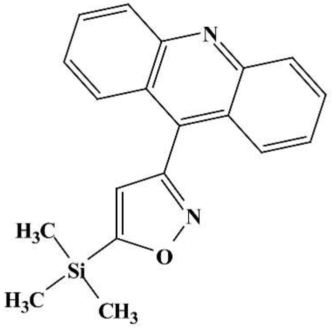

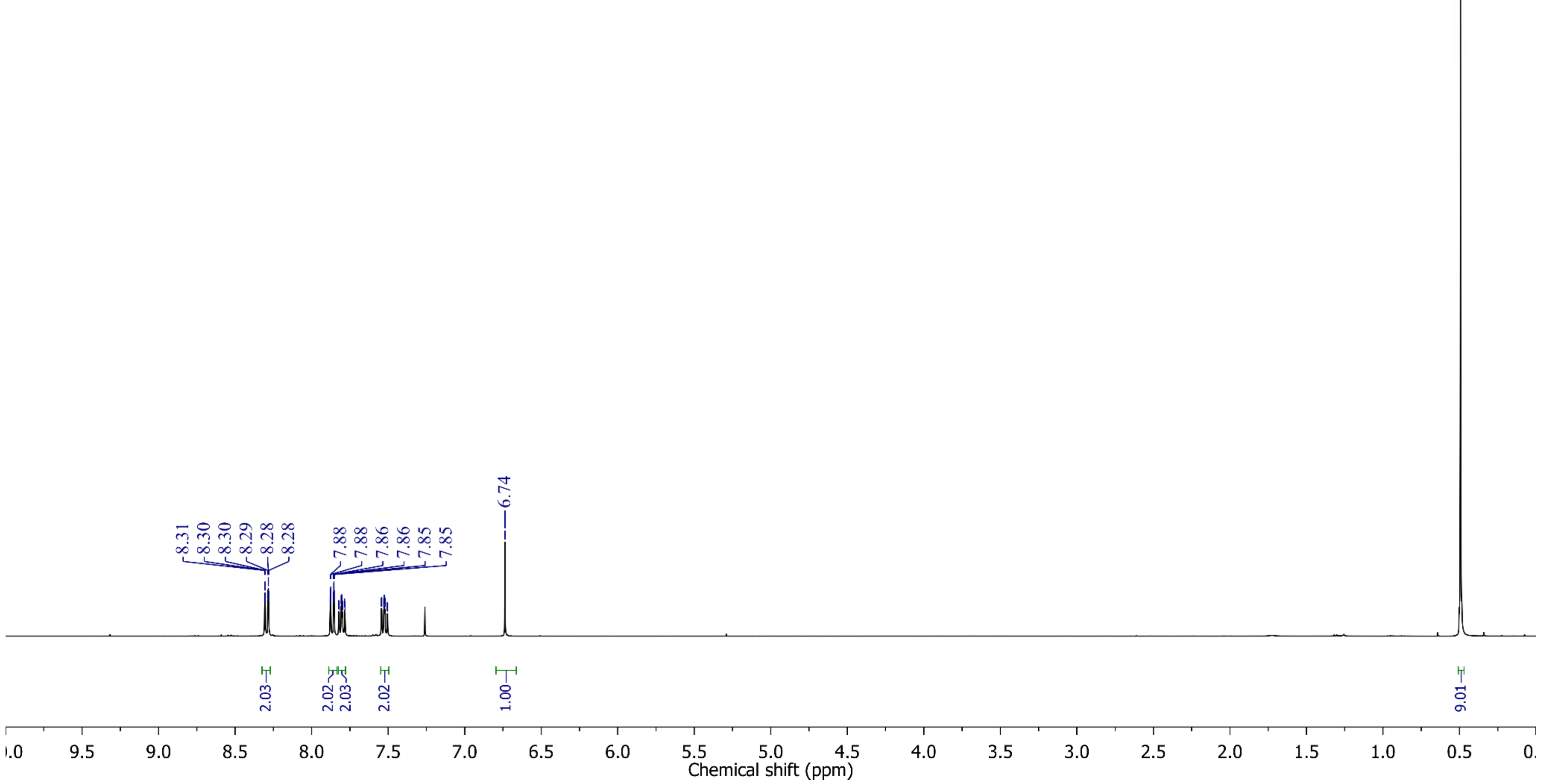

3-(Acridin-9-yl)-5-(trimethylsilyl)isoxazole (7b), $^{13}C\{^{1}H\}$ NMR, $CDCl_3$, 100 MHz

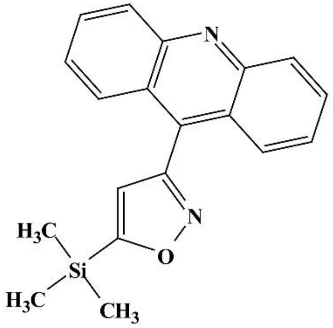

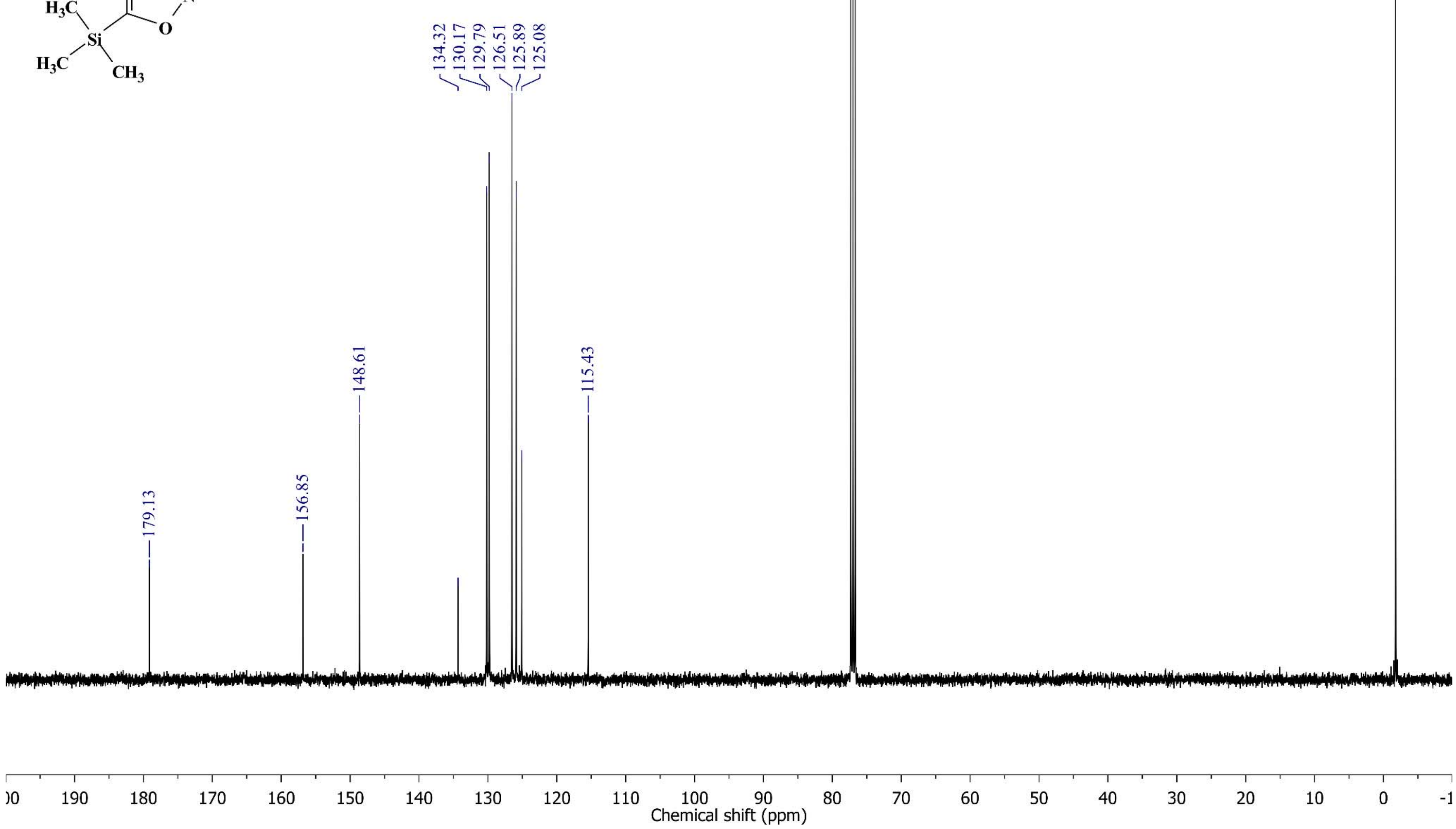

3-(Acridin-9-yl)-5-(trimethylsilyl)isoxazole (7b), DEPT, $CDCl_3$, 100 MHz

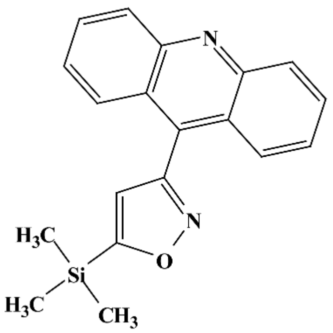

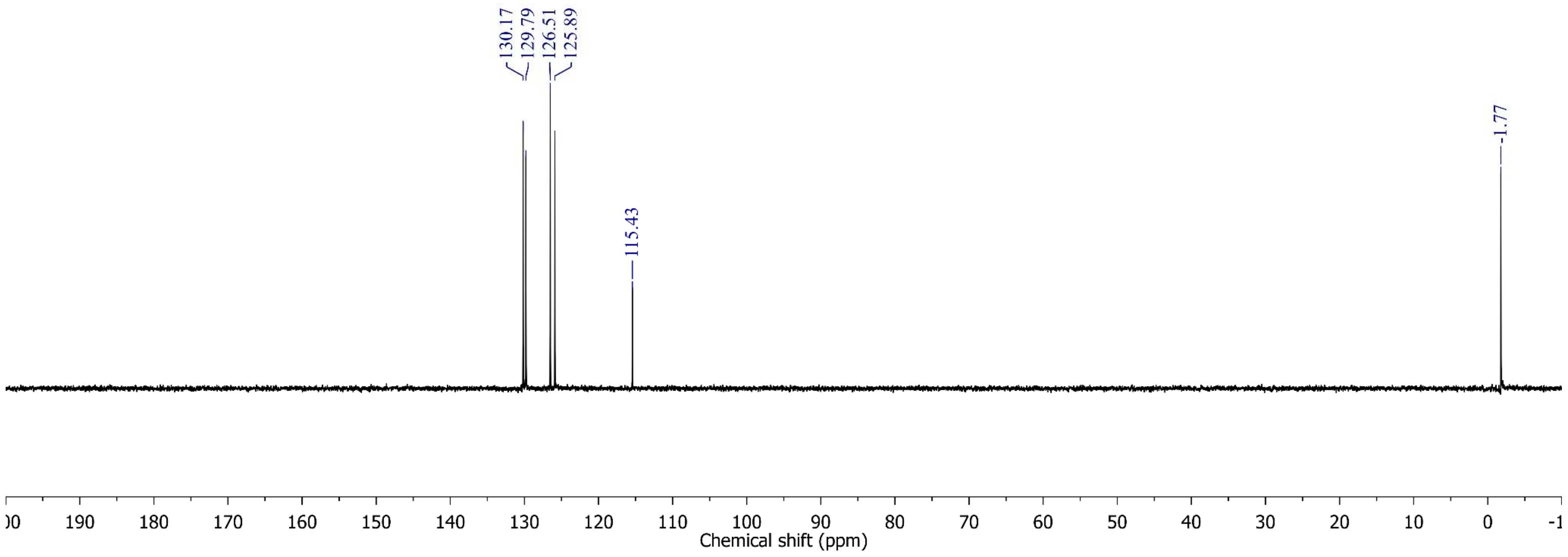

3-(Acridin-9-yl)-5-((4-isopropylphenoxy)methyl)isoxazole (7c), ^{1}H NMR, CDCl$_3$, 400 MHz

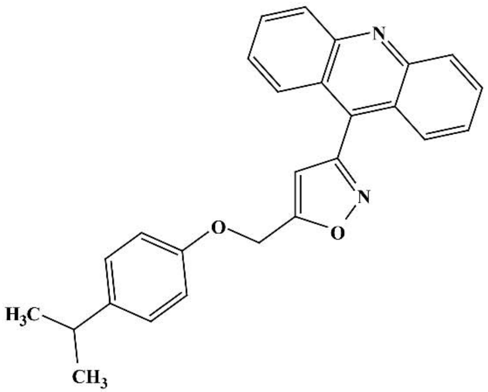

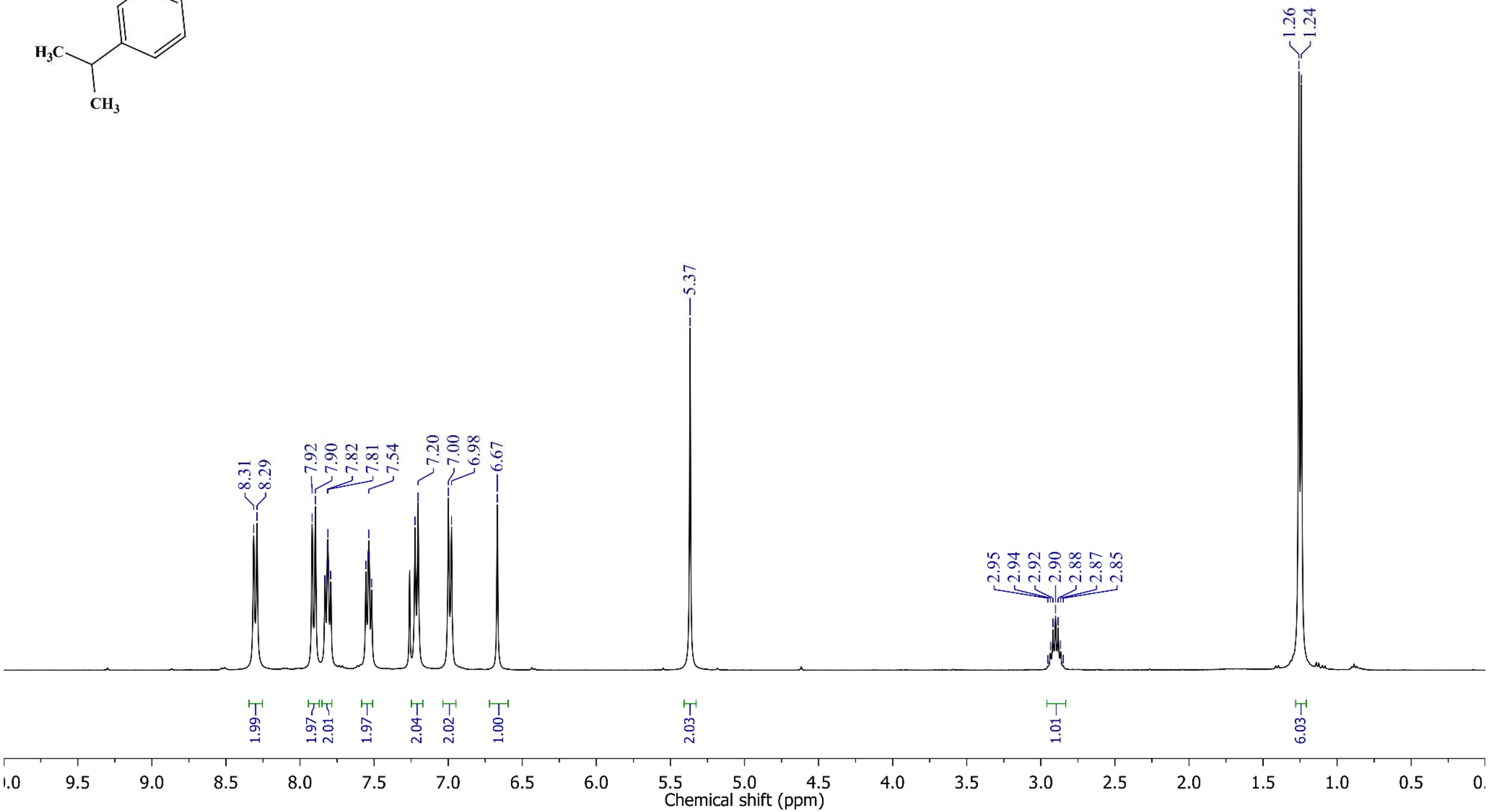

3-(Acridin-9-yl)-5-((4-isopropylphenoxy)methyl)isoxazole (7c), $^{13}C\{^{1}H\}$ NMR, $CDCl_3$, 100 MHz

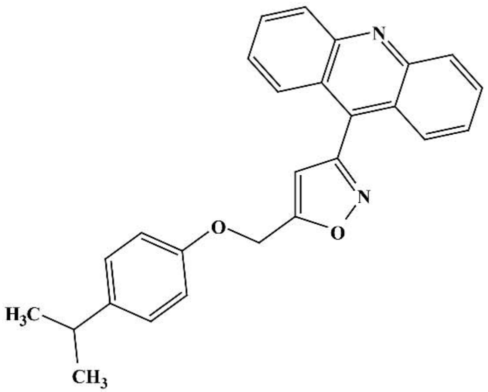

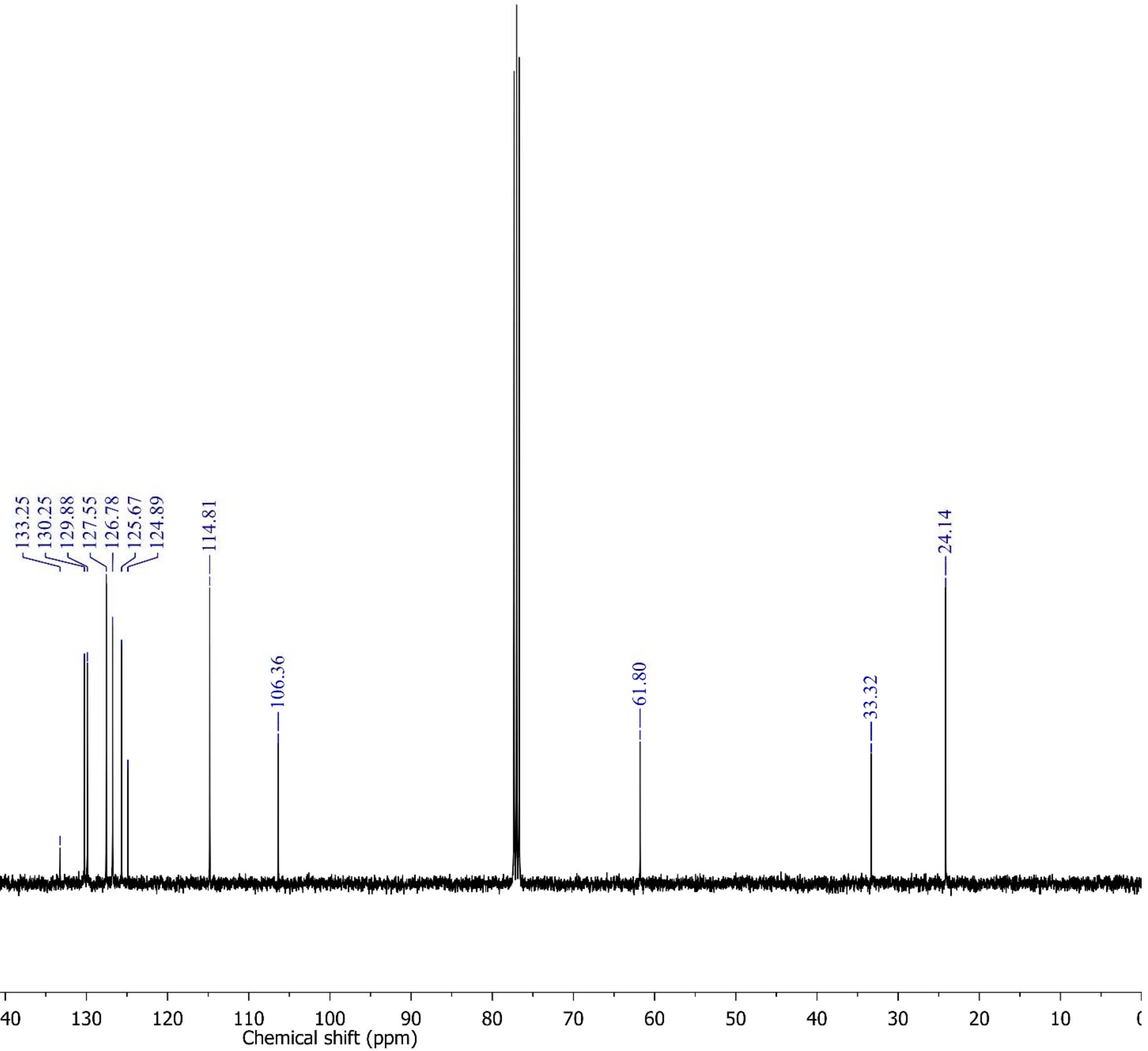

3-(Acridin-9-yl)-5-((4-isopropylphenoxy)methyl)isoxazole (7c), DEPT, $CDCl_3$, 100 MHz

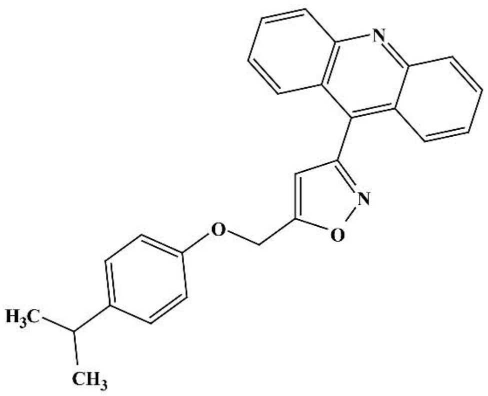

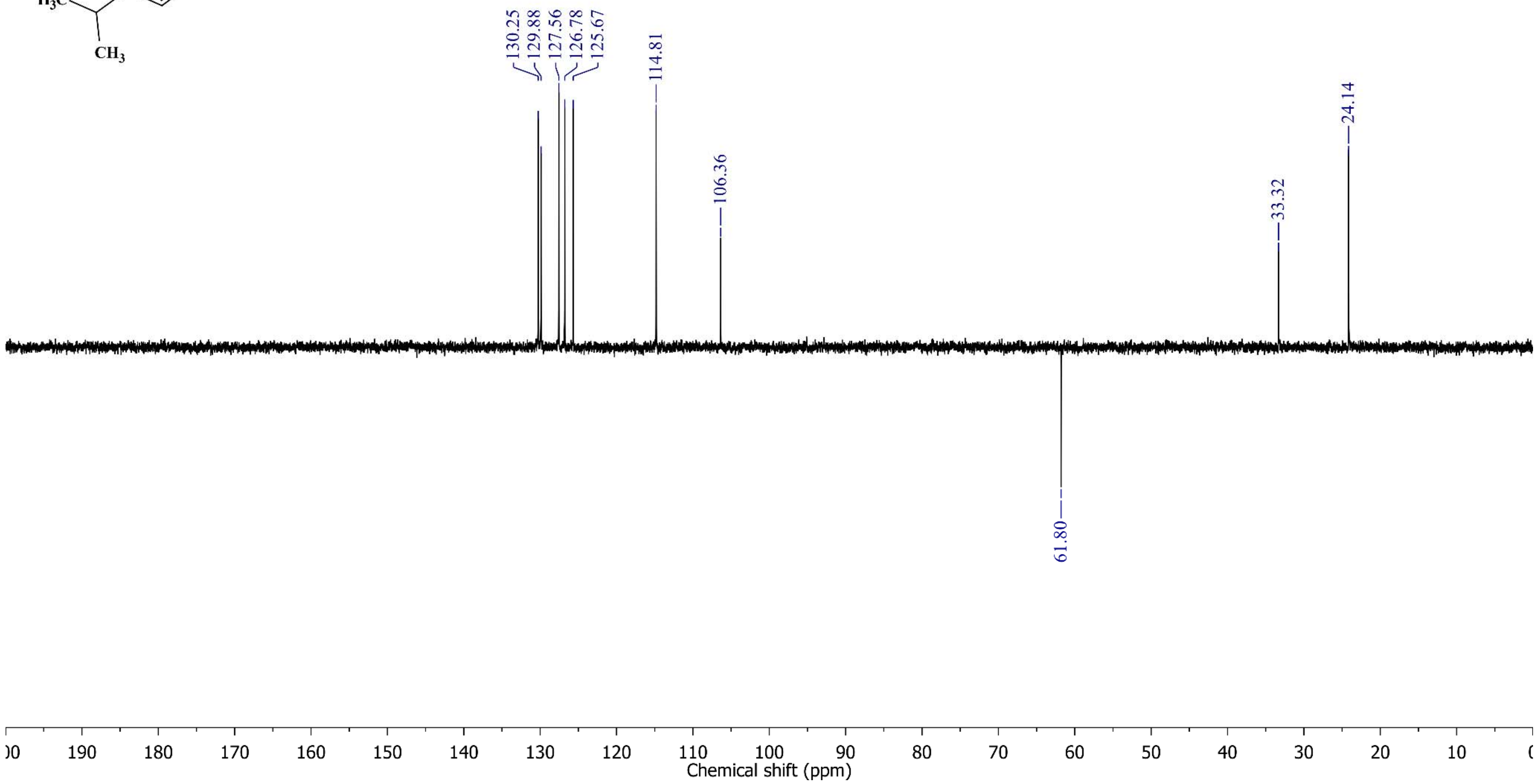

Methyl 3-(2-methylacridin-9-yl)isoxazole-5-carboxylate (7d), ^{1}H NMR, $CDCl_3$, 400 MHz

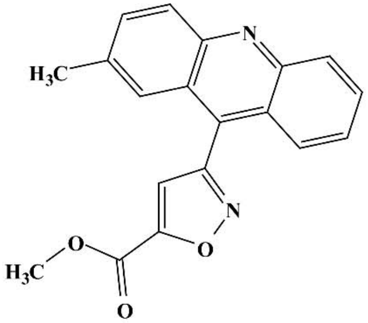

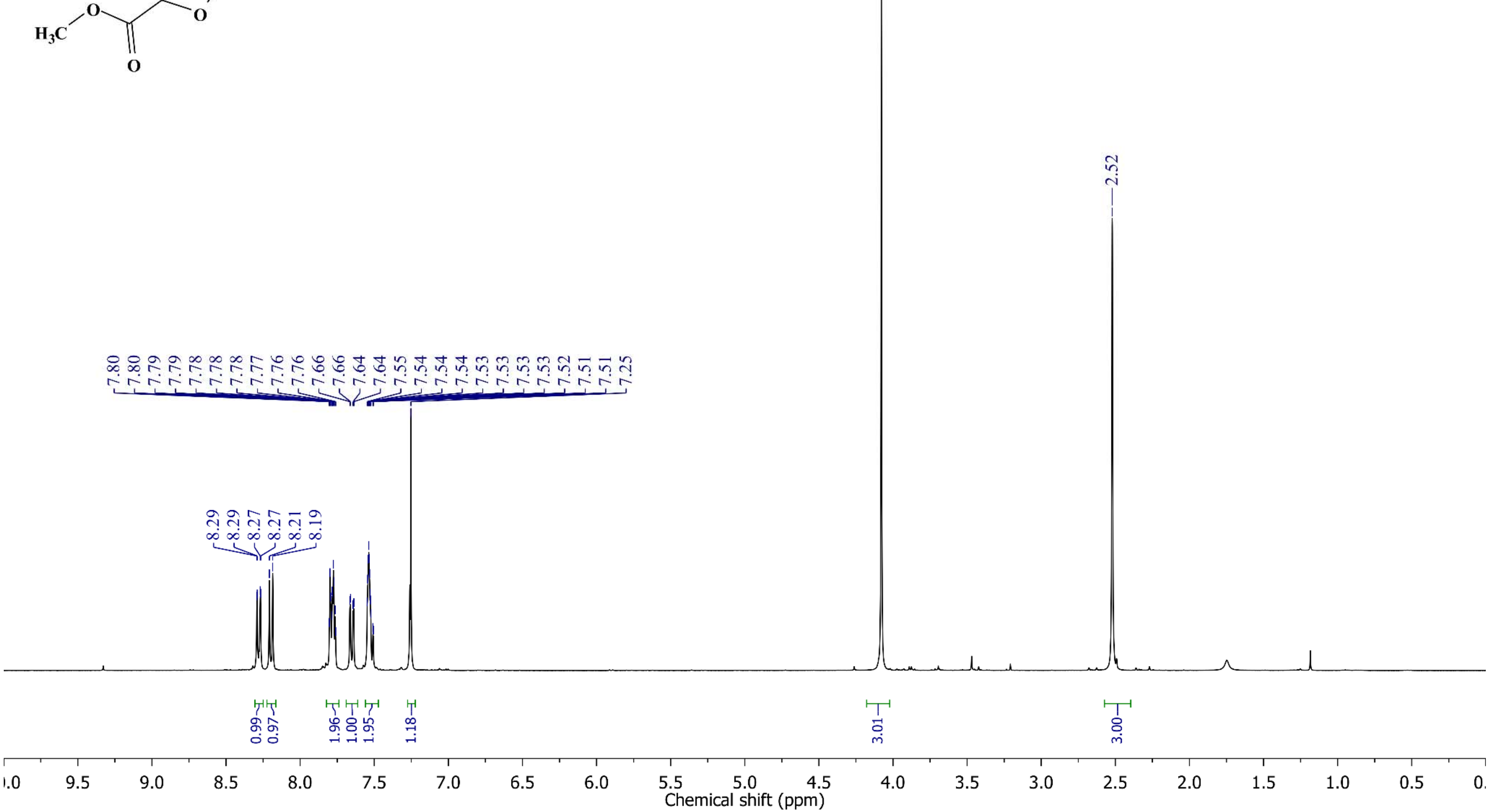

Methyl 3-(2-methylacridin-9-yl)isoxazole-5-carboxylate (7d), $^{13}C\{^{1}H\}$ NMR, $CDCl_3$, 100 MHz

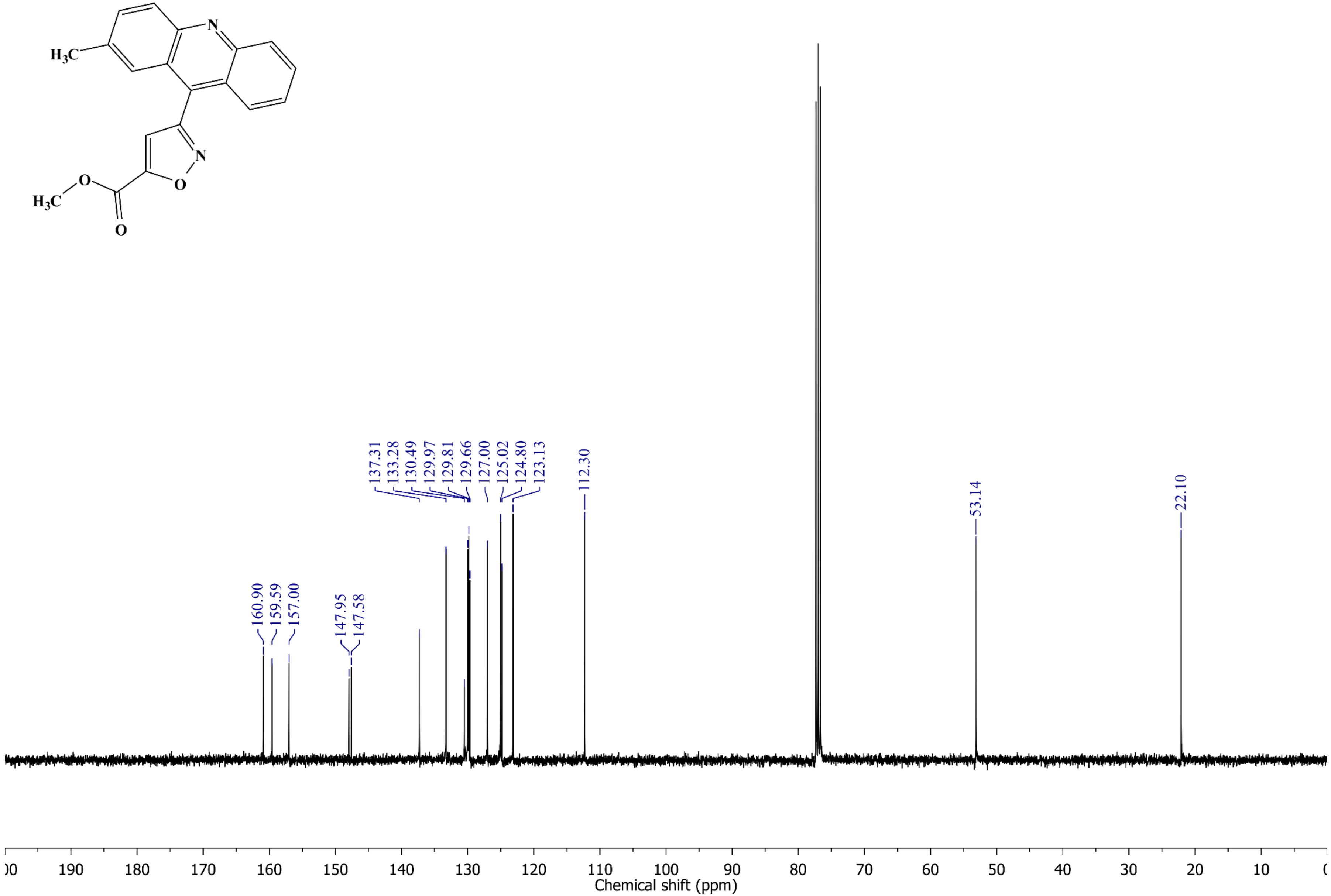

Methyl 3-(2-methylacridin-9-yl)isoxazole-5-carboxylate (7d), DEPT, $CDCl_3$, 100 MHz

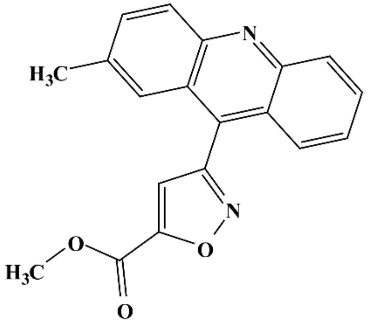

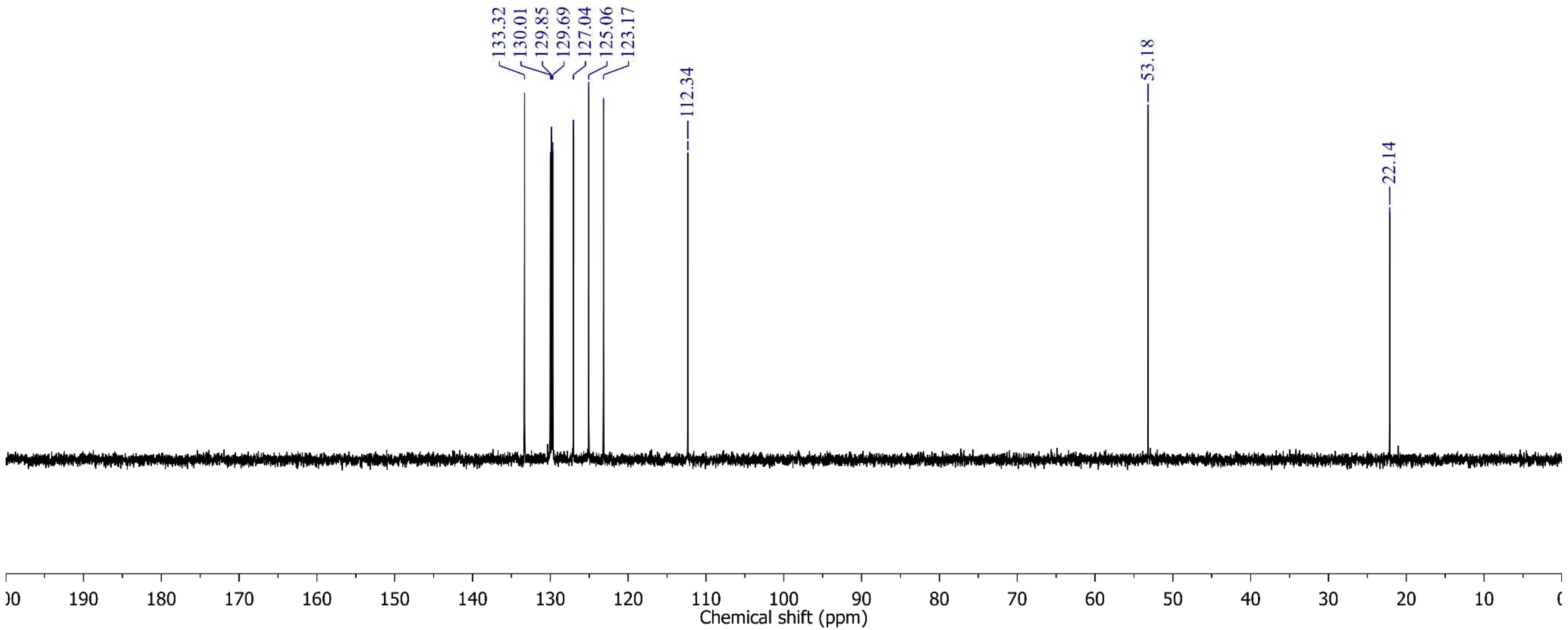

5-Methoxy-4-((3-(2-methylacridin-9-yl)isoxazol-5-yl)methyl)-3-(naphthalen-2-yl)isoxazole (7e), ^{1}H NMR, $CDCl_3$, 400 MHz

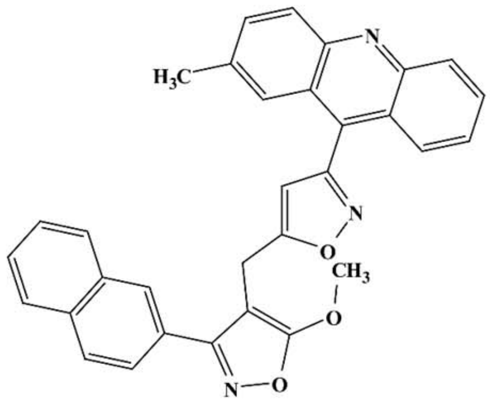

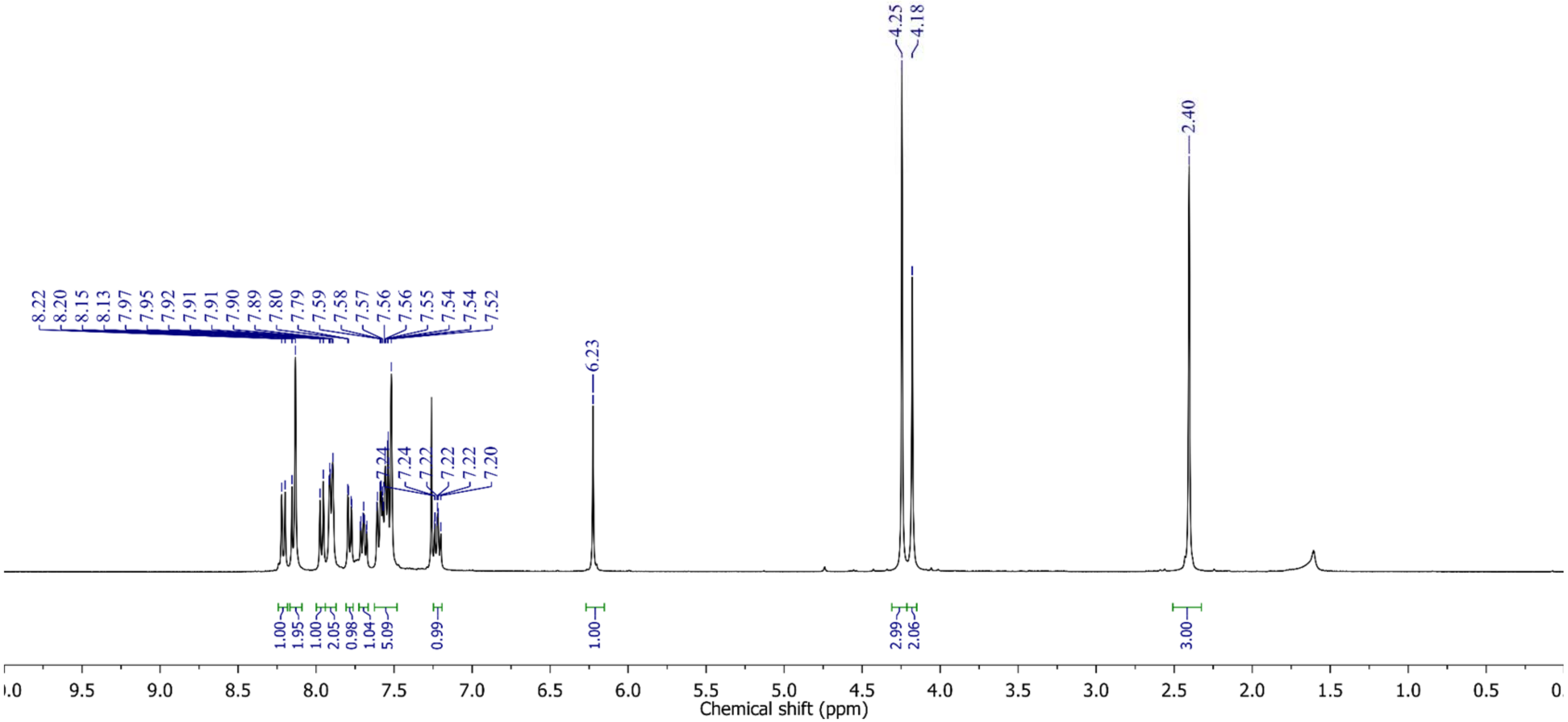

5-Methoxy-4-((3-(2-methylacridin-9-yl)isoxazol-5-yl)methyl)-3-(naphthalen-2-yl)isoxazole (7e), $^{13}C\{^1H\}$ NMR, $CDCl_3$, 100 MHz

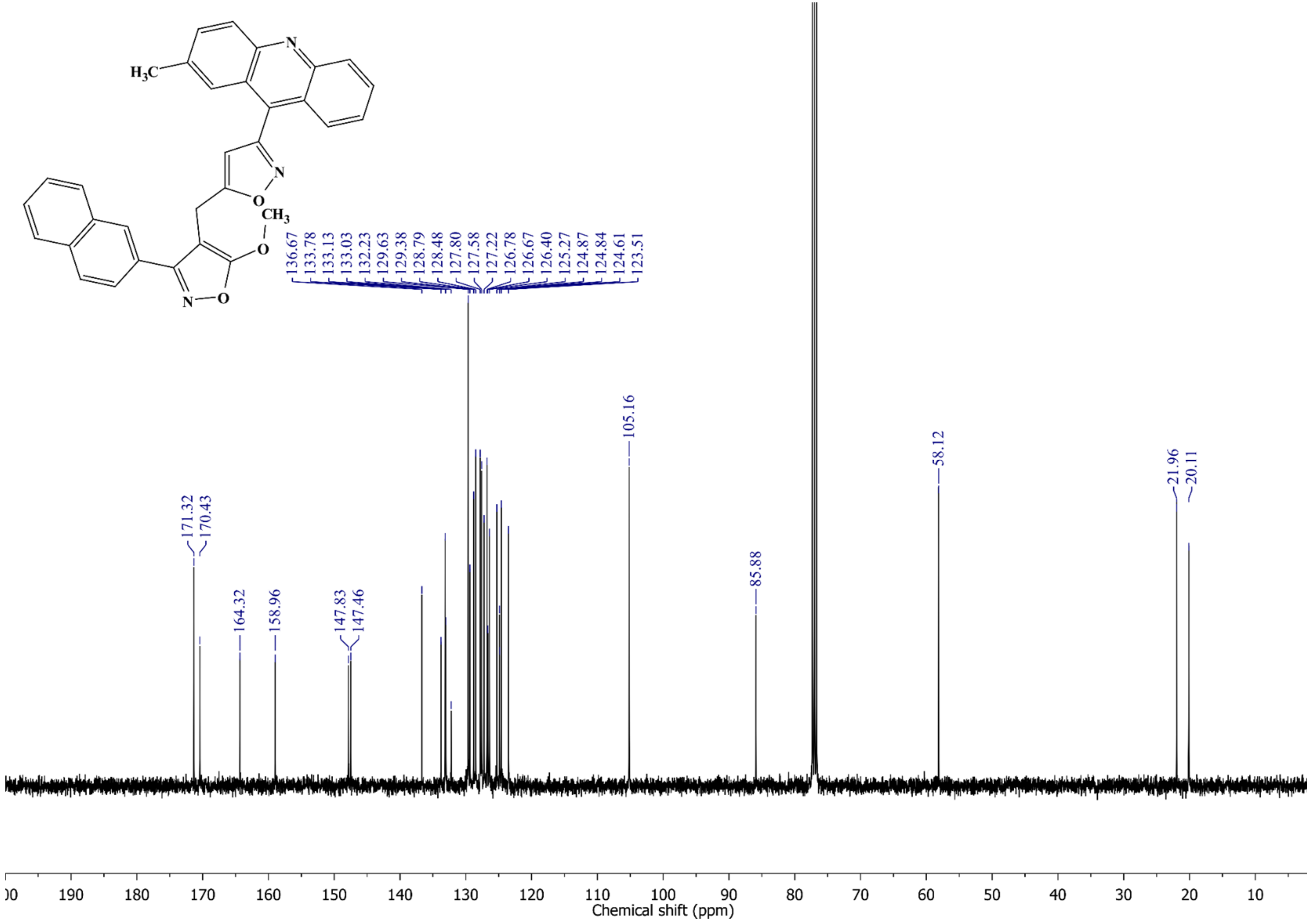

5-Methoxy-4-((3-(2-methylacridin-9-yl)isoxazol-5-yl)methyl)-3-(naphthalen-2-yl)isoxazole (7e), DEPT, $CDCl_3$, 100 MHz

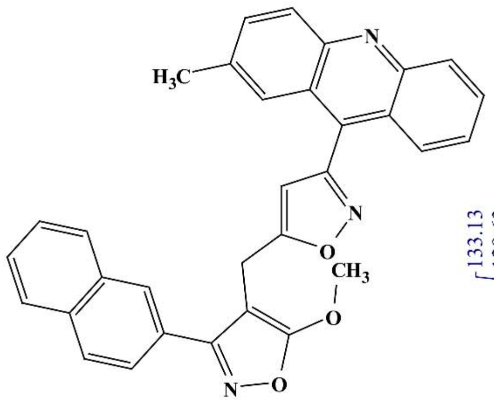

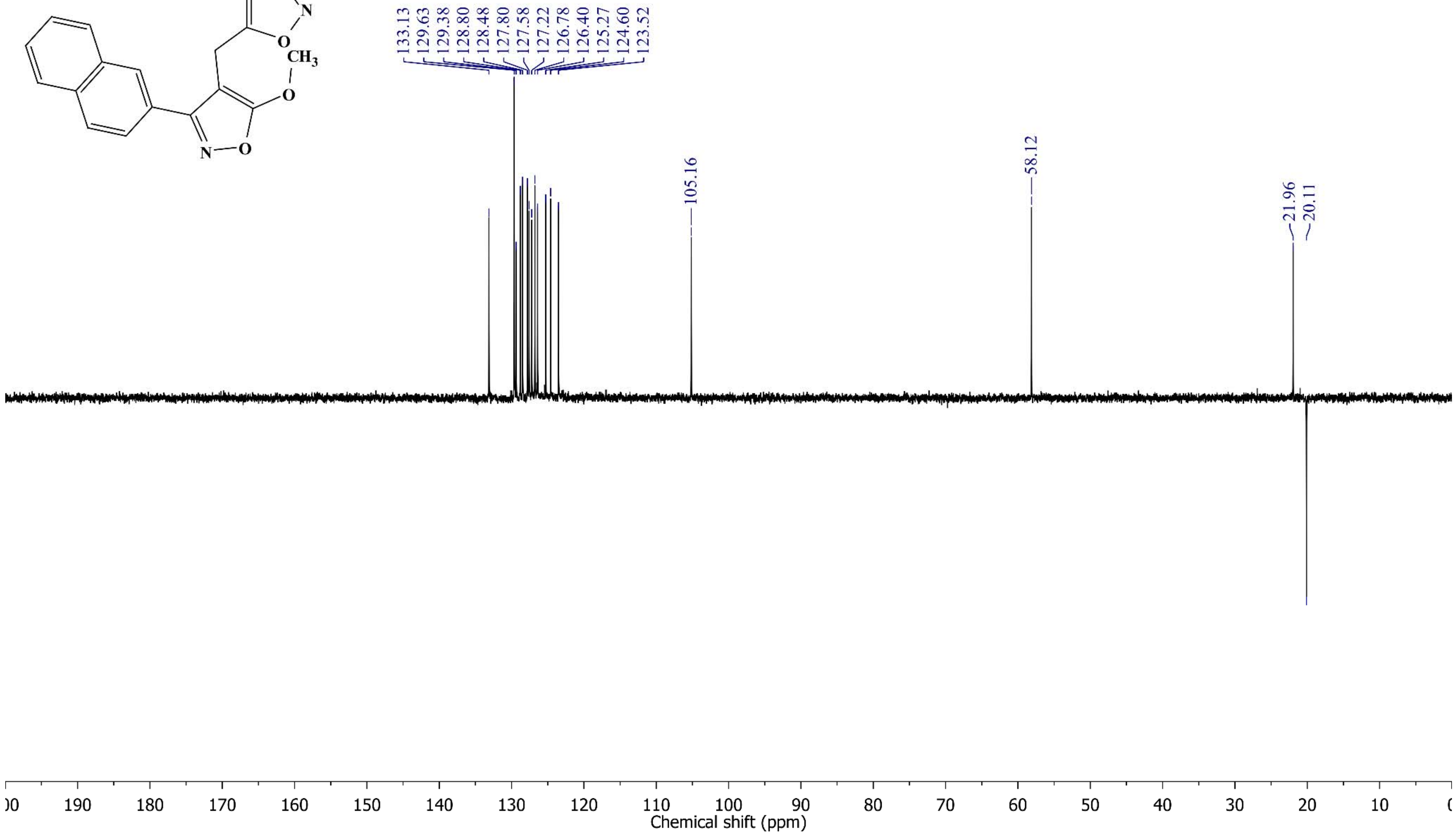

5-(4-Chlorophenyl)-3-(2-nitroacridin-9-yl)isoxazole (7f), ^{1}H NMR, DMSO-d$_6$, 400 MHz

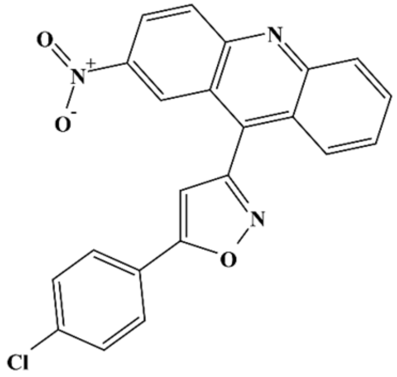

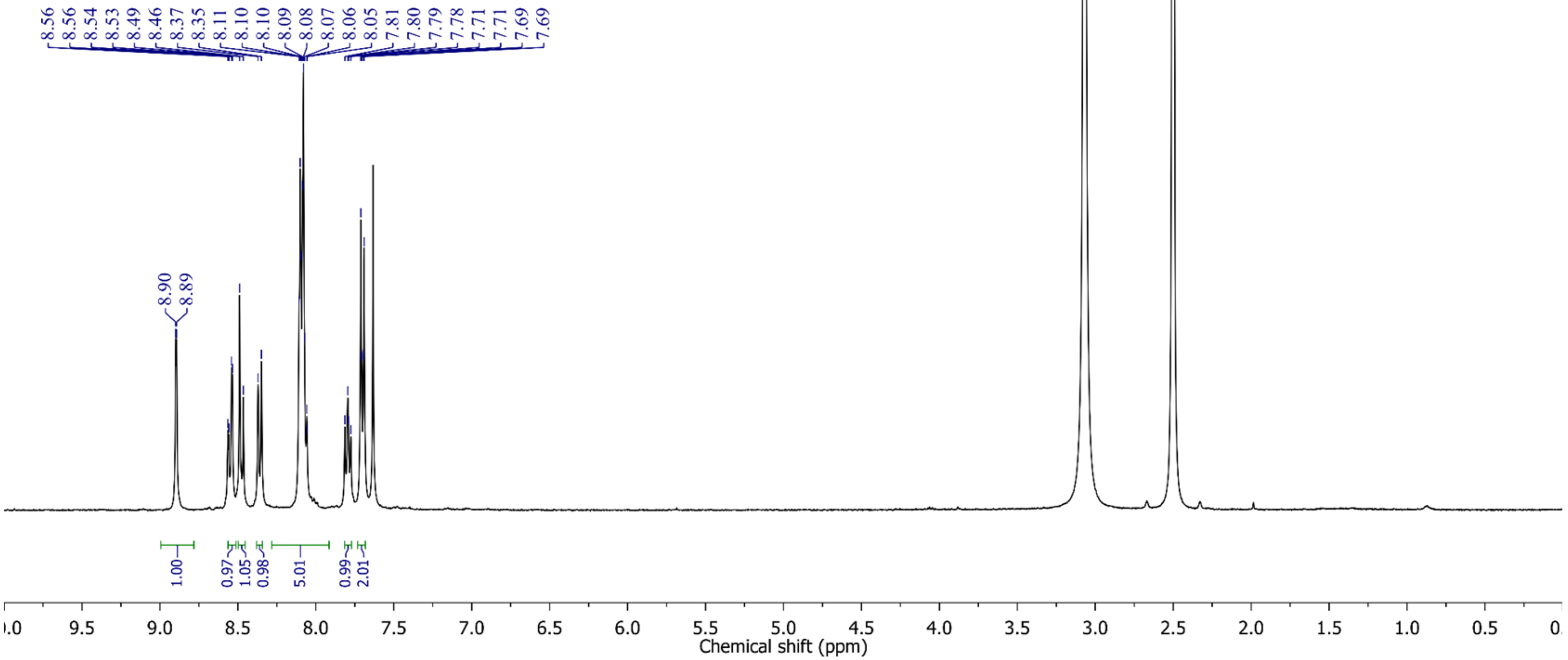

5-(4-Chlorophenyl)-3-(2-nitroacridin-9-yl)isoxazole (7f), $^{13}C\{^1H\}$ NMR, DMSO-d_6, 100 MHz

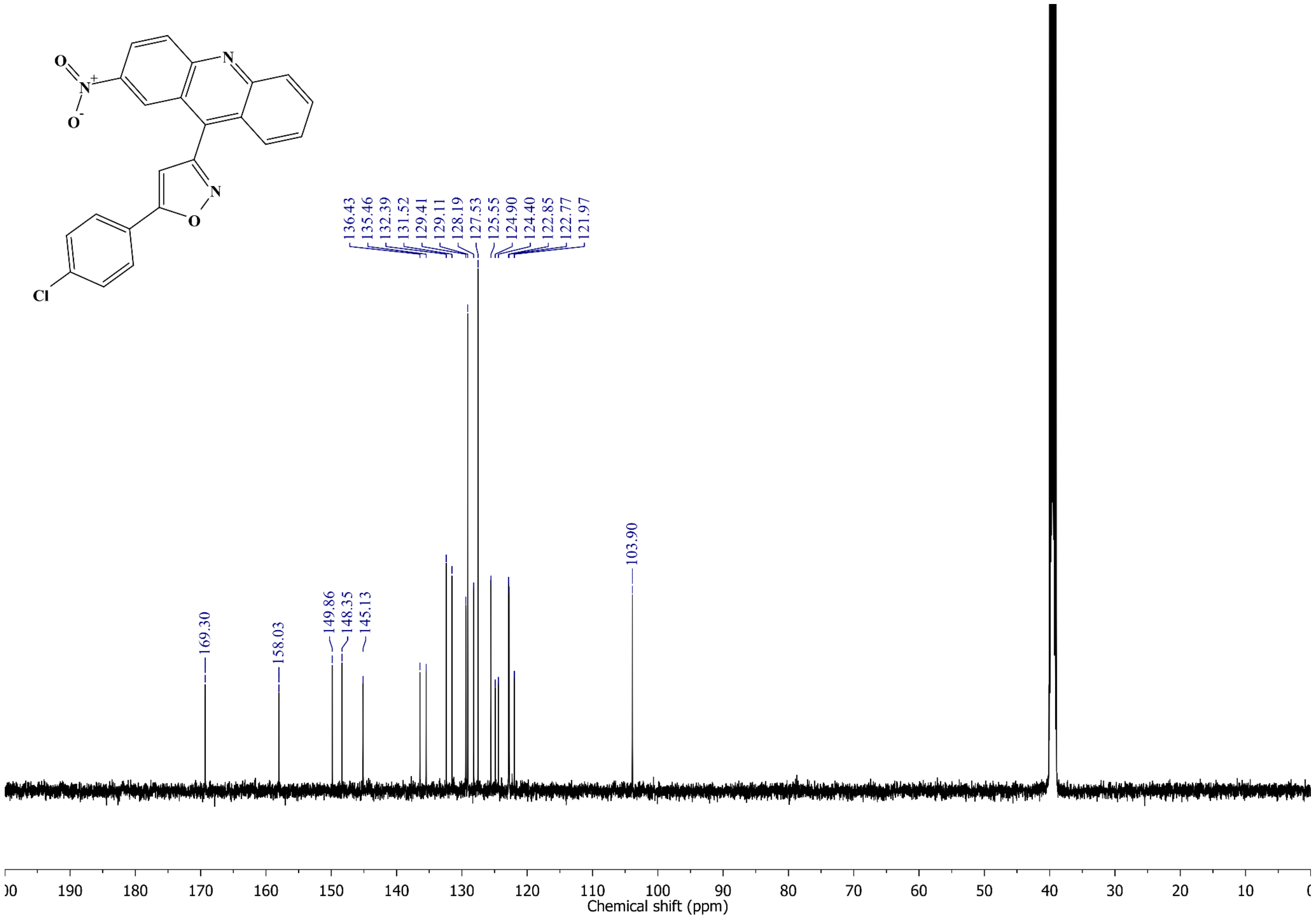

5-(4-Chlorophenyl)-3-(2-nitroacridin-9-yl)isoxazole (7f), DEPT, DMSO-d$_6$, 100 MHz

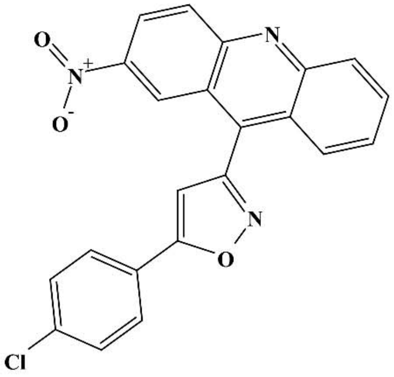

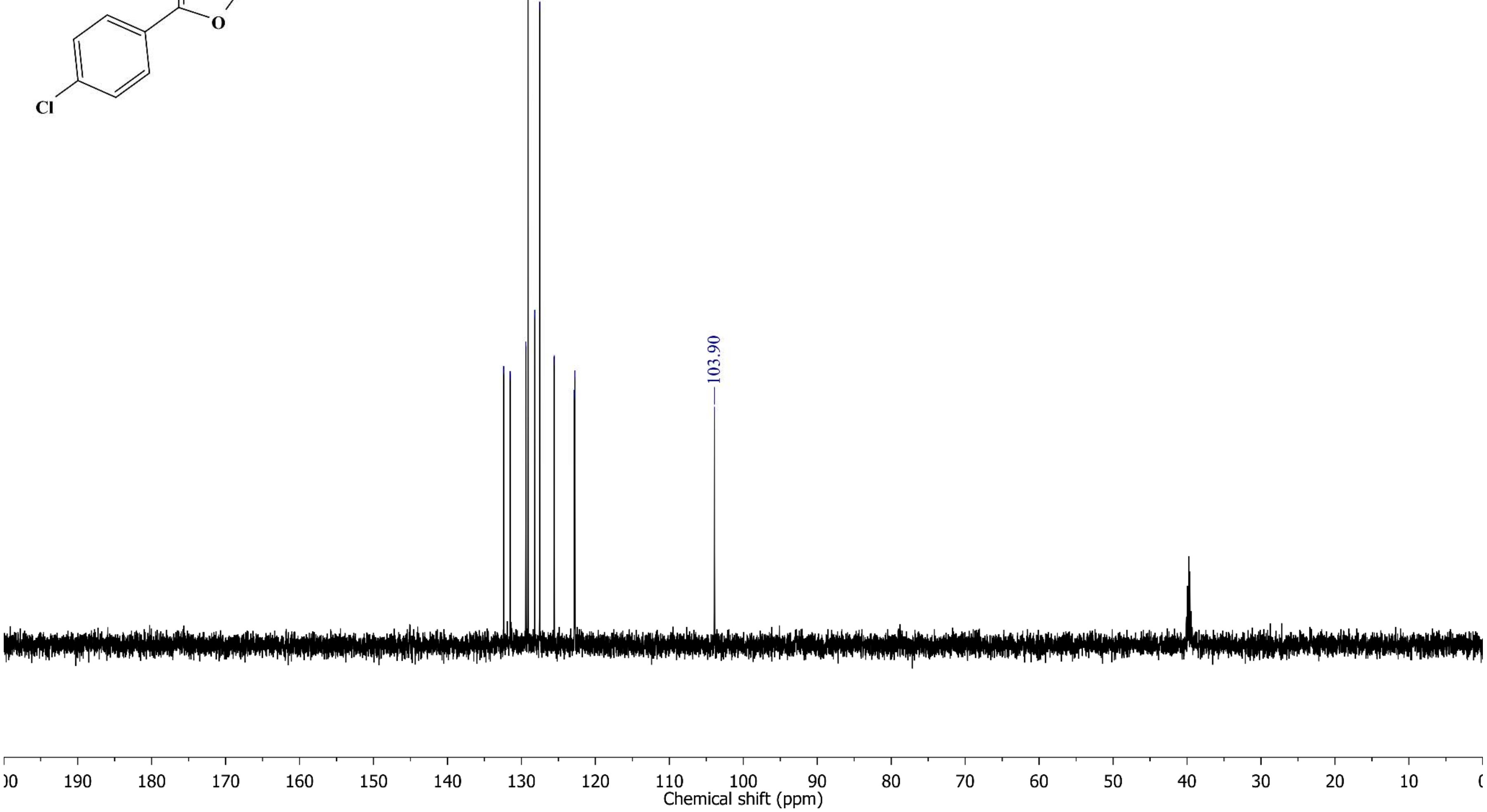

5-(2-Fluorophenyl)-3-(9-methylbenzo[*c*]acridin-7-yl)isoxazole (7g), ^{1}H NMR, $CDCl_3$, 400 MHz

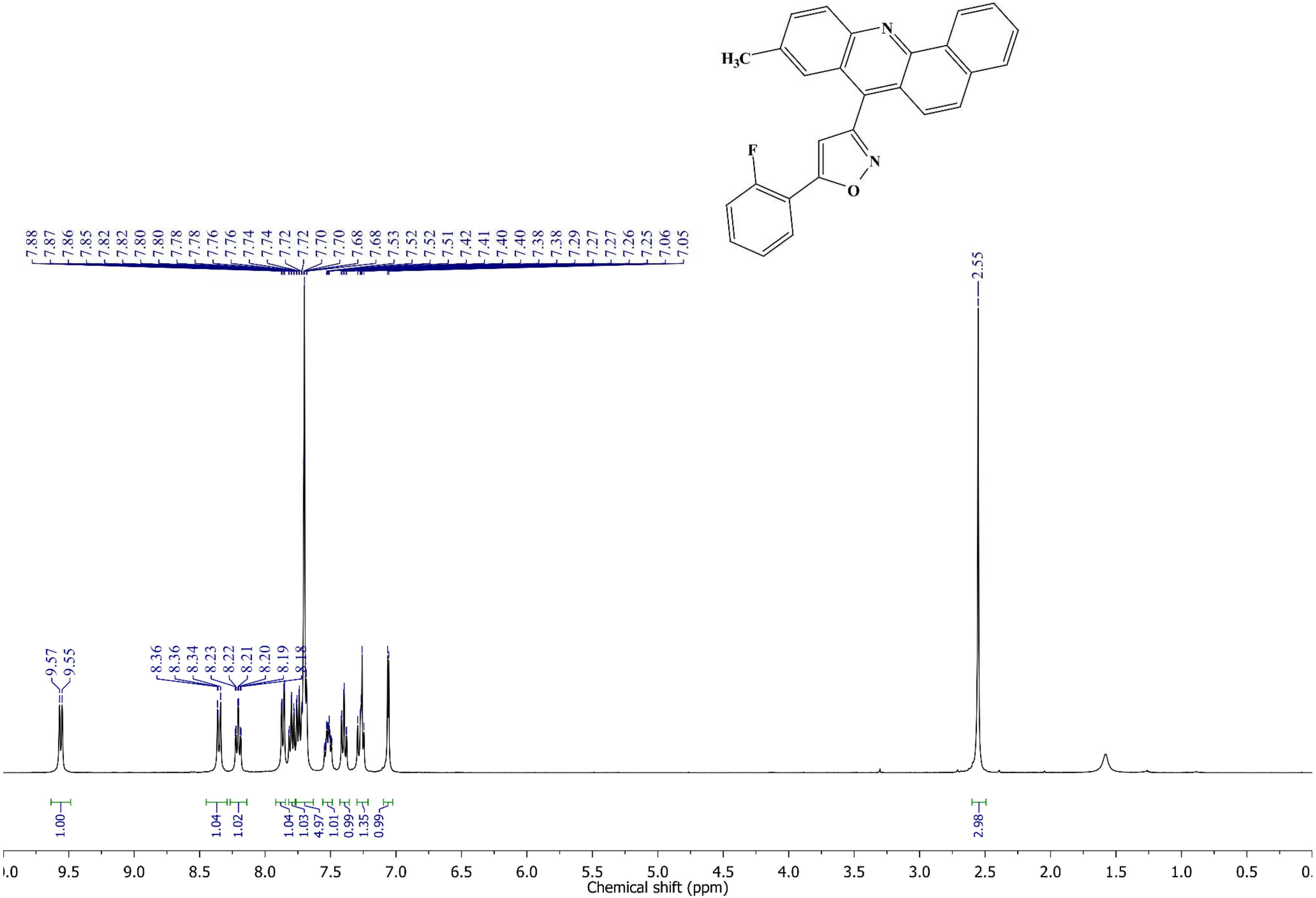

5-(2-Fluorophenyl)-3-(9-methylbenzo[*c*]acridin-7-yl)isoxazole (7g), $^{13}C\{^1H\}$ NMR, $CDCl_3$, 100 MHz

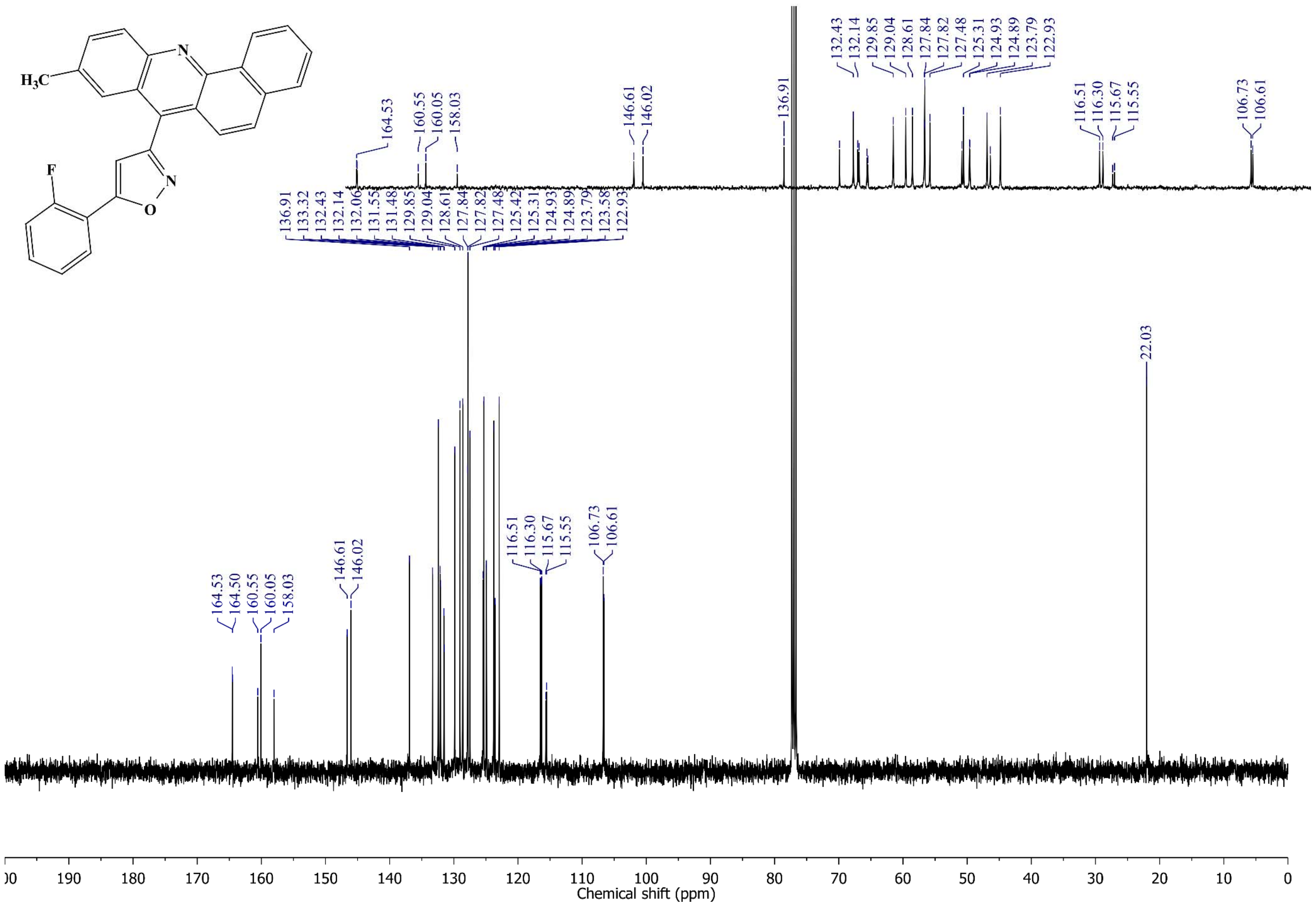

5-(2-Fluorophenyl)-3-(9-methylbenzo[*c*]acridin-7-yl)isoxazole (7g), DEPT, $CDCl_3$, 100 MHz

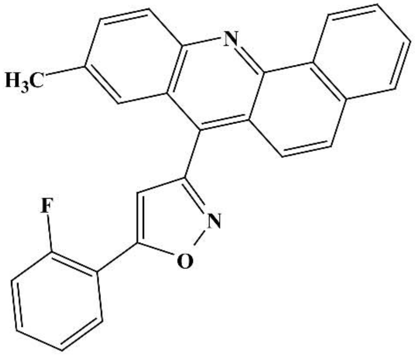

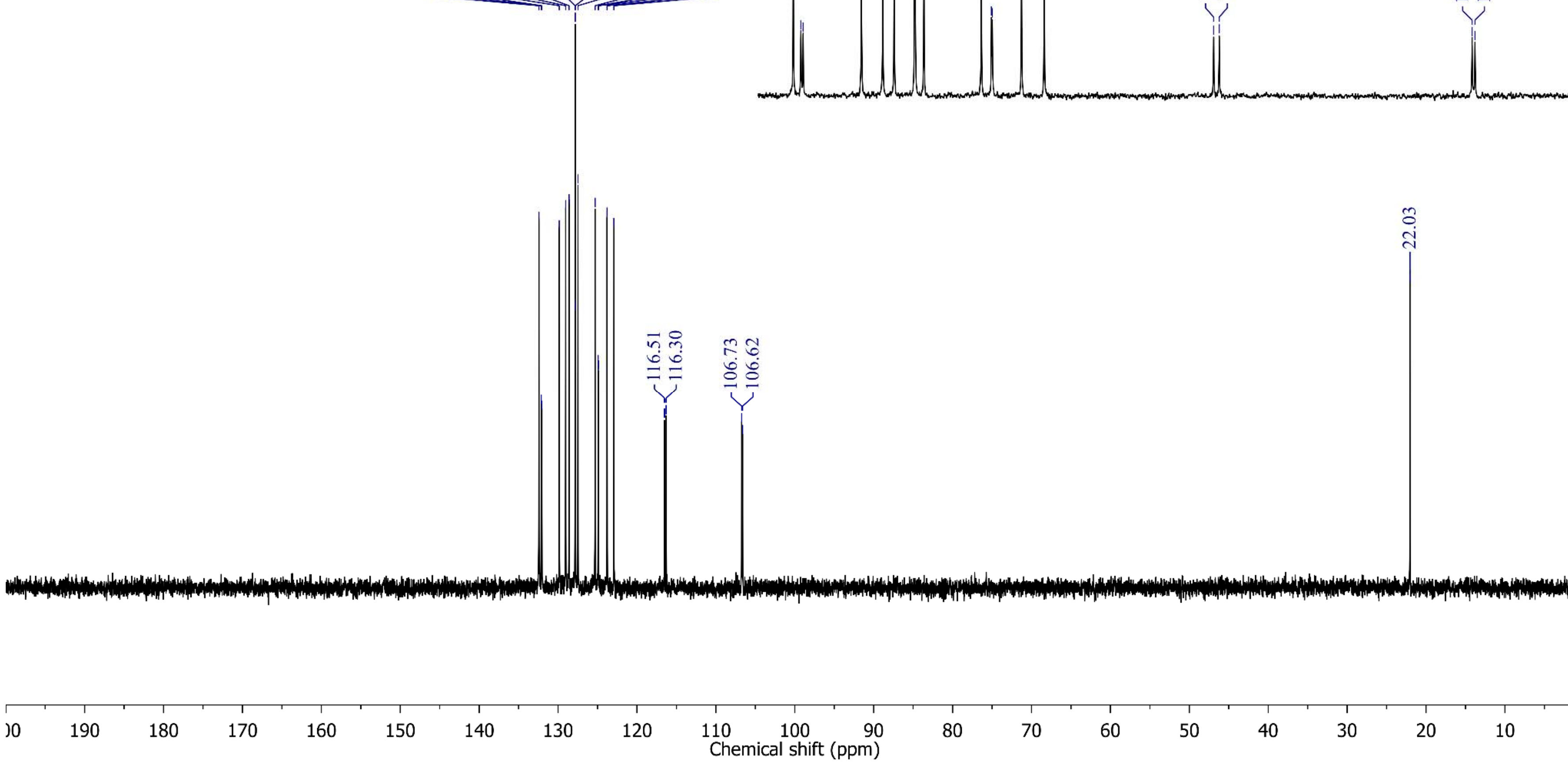

5-((4-Isopropylphenoxy)methyl)-3-(9-methylbenzo[*c*]acridin-7-yl)isoxazole (7h), ^{1}H NMR, $CDCl_3$, 400 MHz

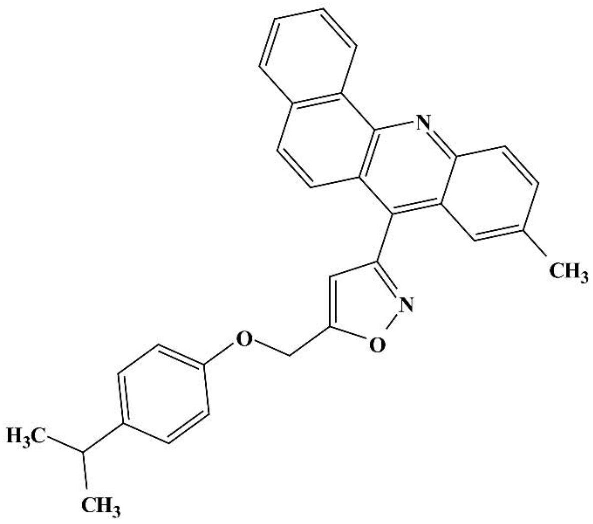

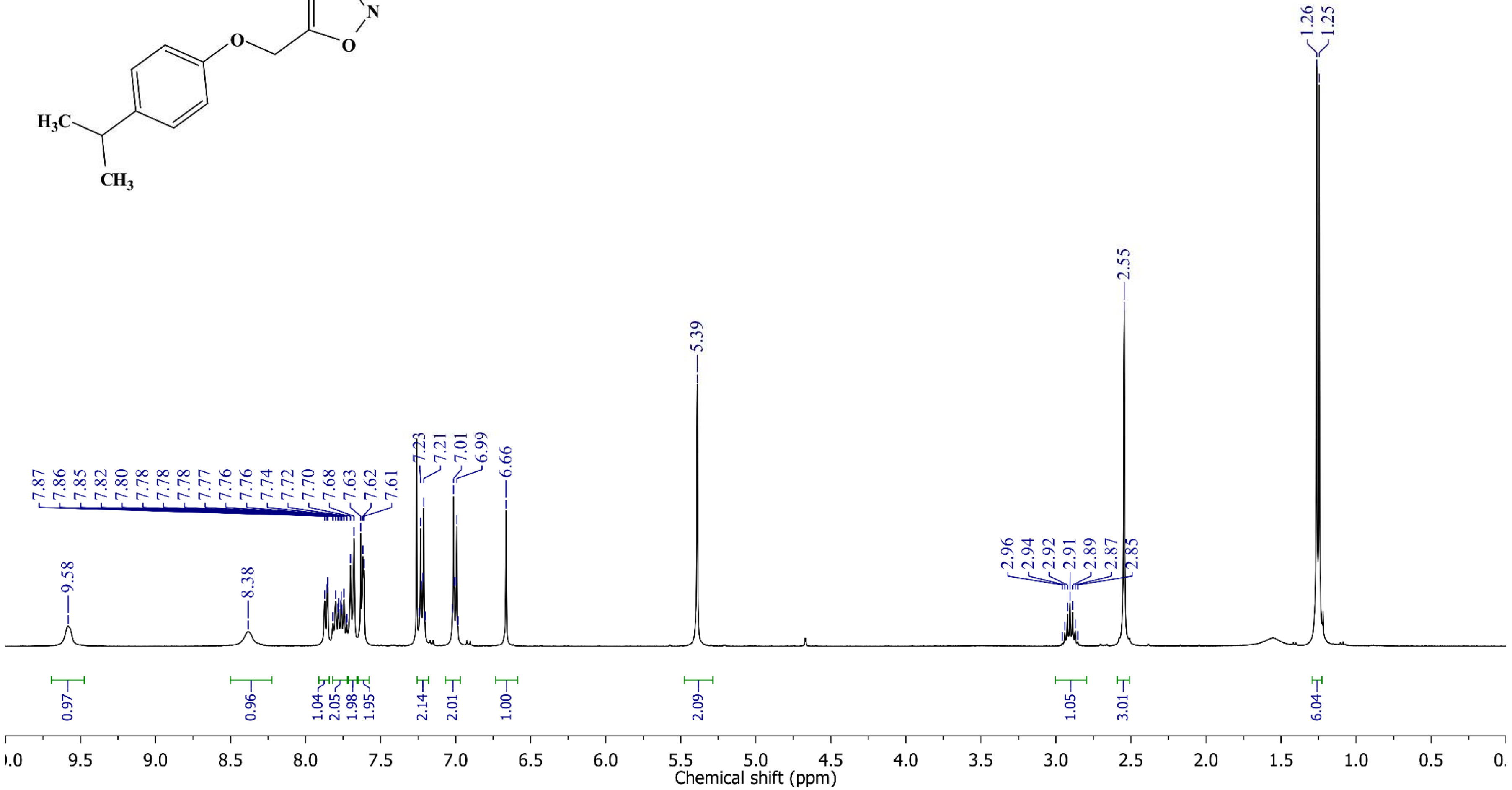

5-((4-Isopropylphenoxy)methyl)-3-(9-methylbenzo[*c*]acridin-7-yl)isoxazole (7h), $^{13}C\{^1H\}$ NMR, $CDCl_3$, 100 MHz

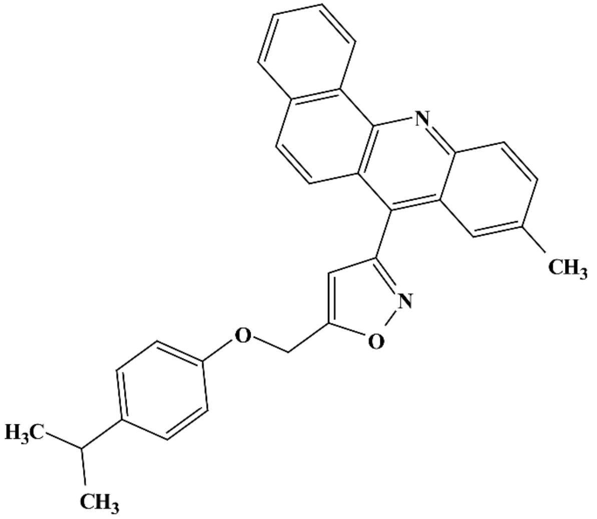

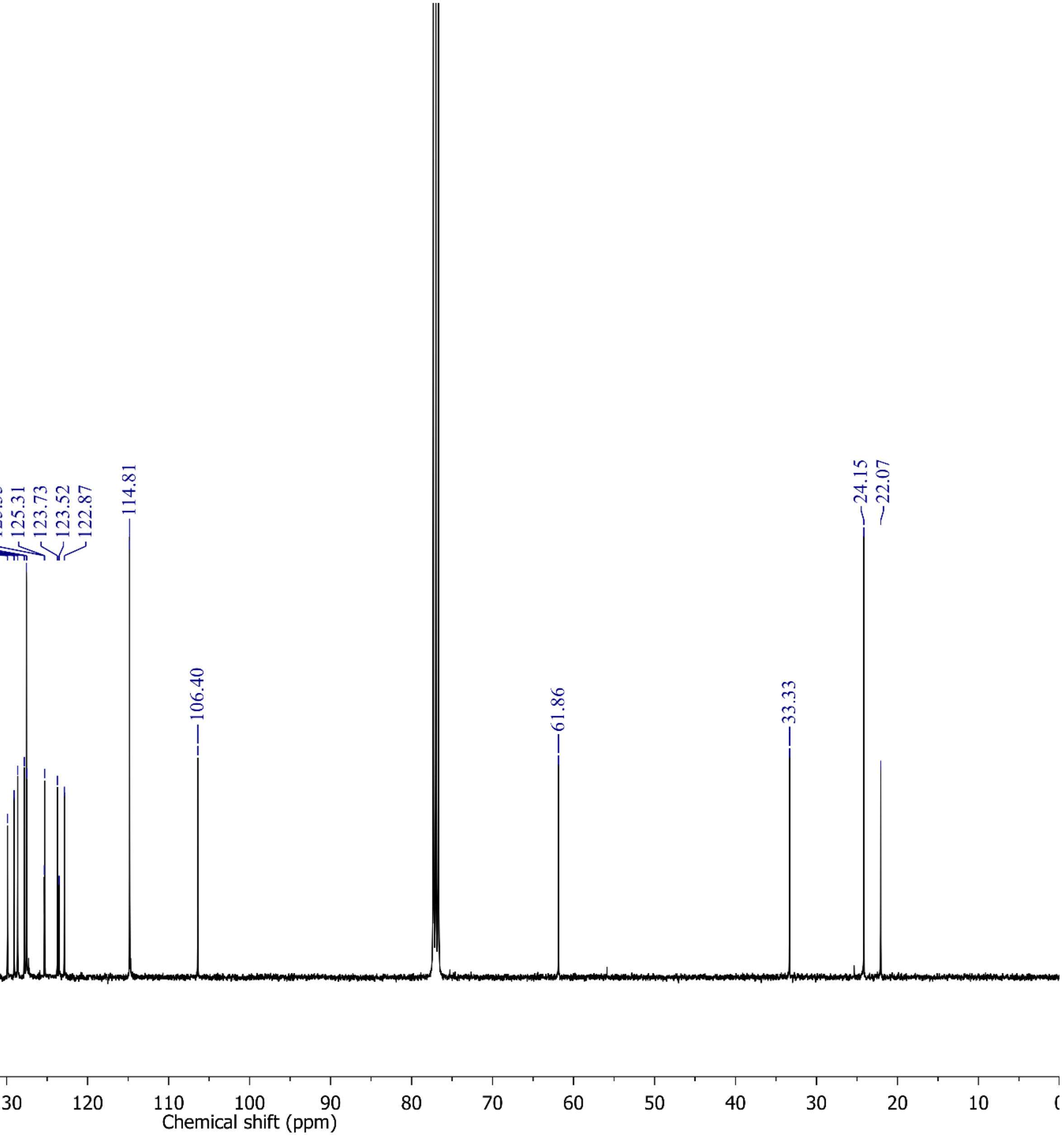

5-((4-Isopropylphenoxy)methyl)-3-(9-methylbenzo[*c*]acridin-7-yl)isoxazole (7h), DEPT, $CDCl_3$, 100 MHz

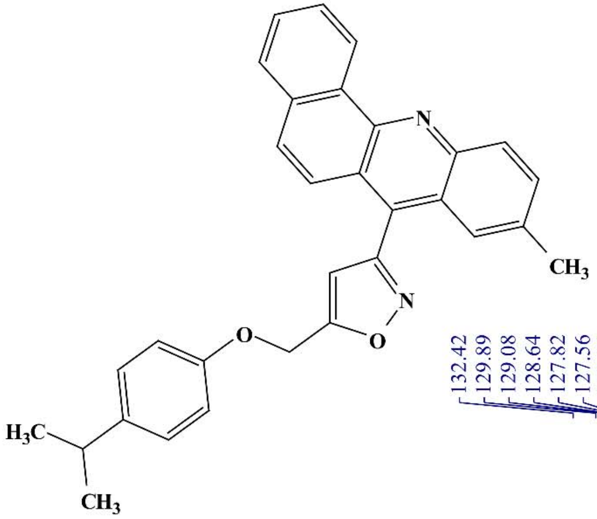

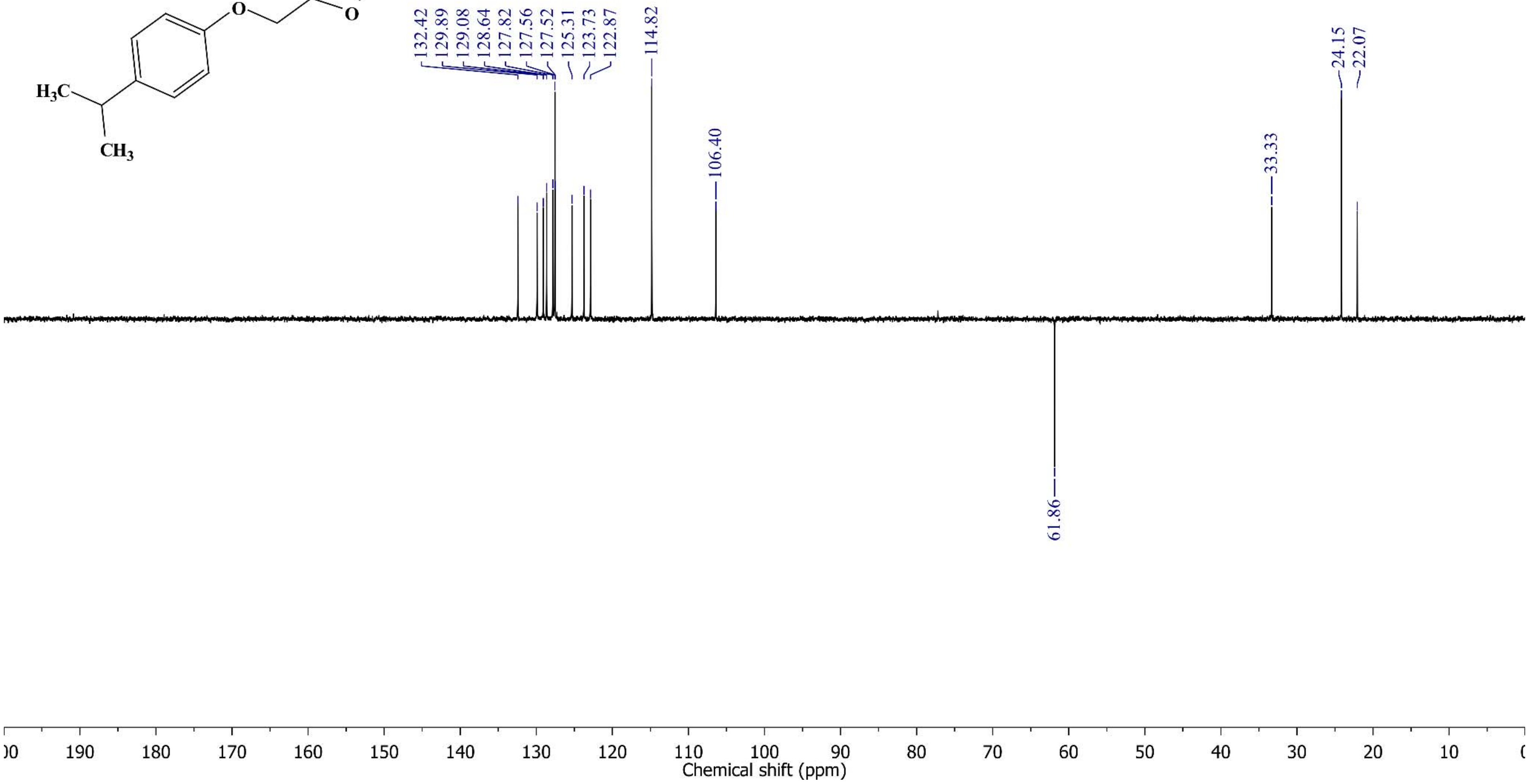

3-(Acridin-9-yl)-5-chloroisoxazole (7i), ^{1}H NMR, $CDCl_3$, 400 MHz

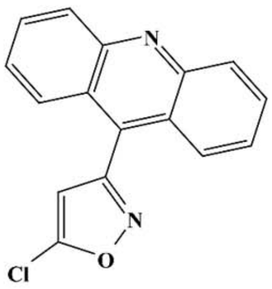

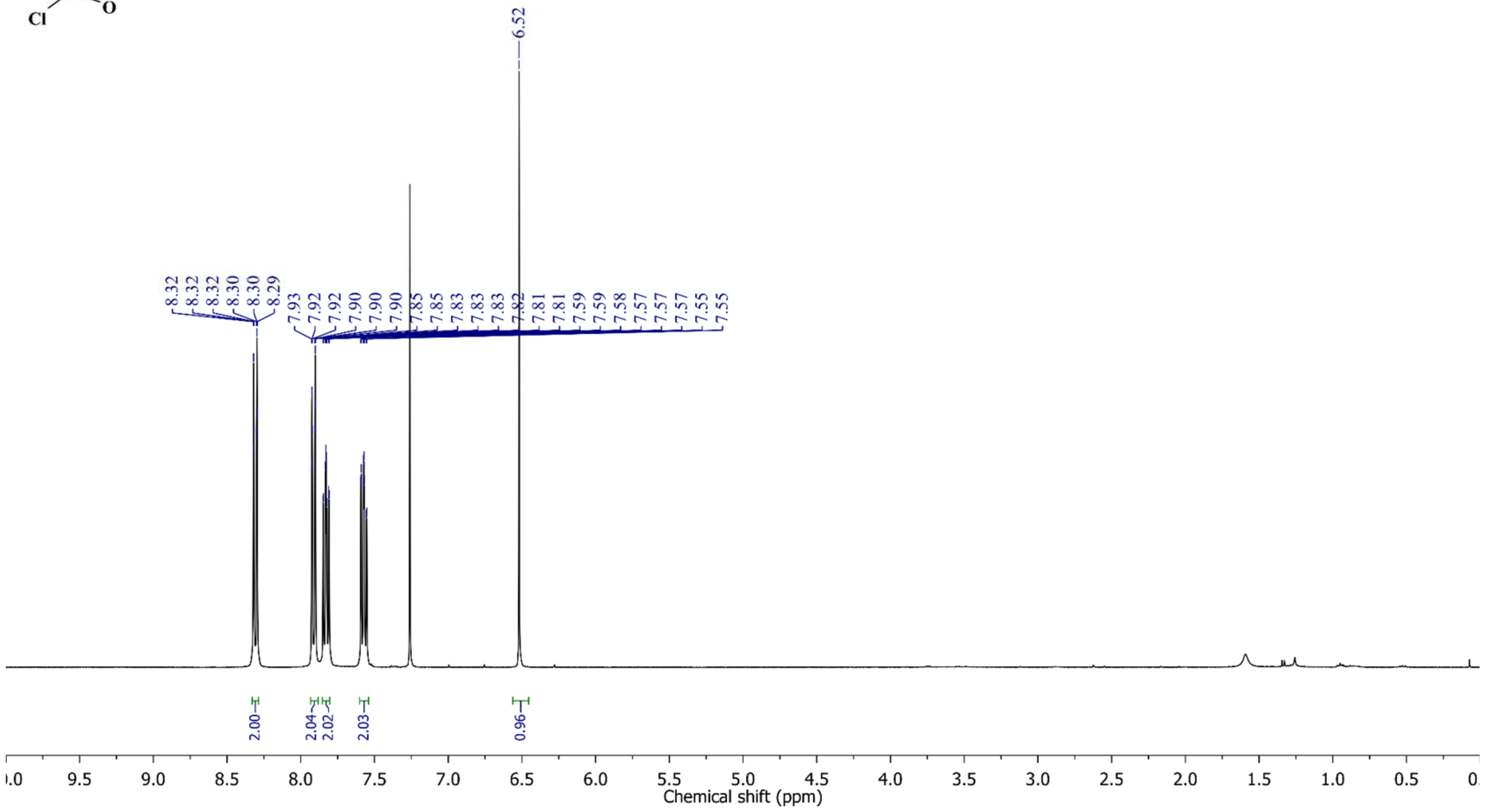

3-(Acridin-9-yl)-5-chloroisoxazole (7i), $^{13}C\{^{1}H\}$ NMR, $CDCl_3$, 100 MHz

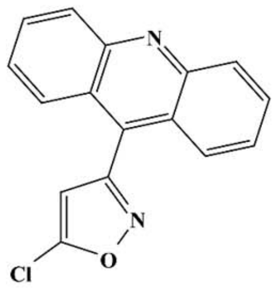

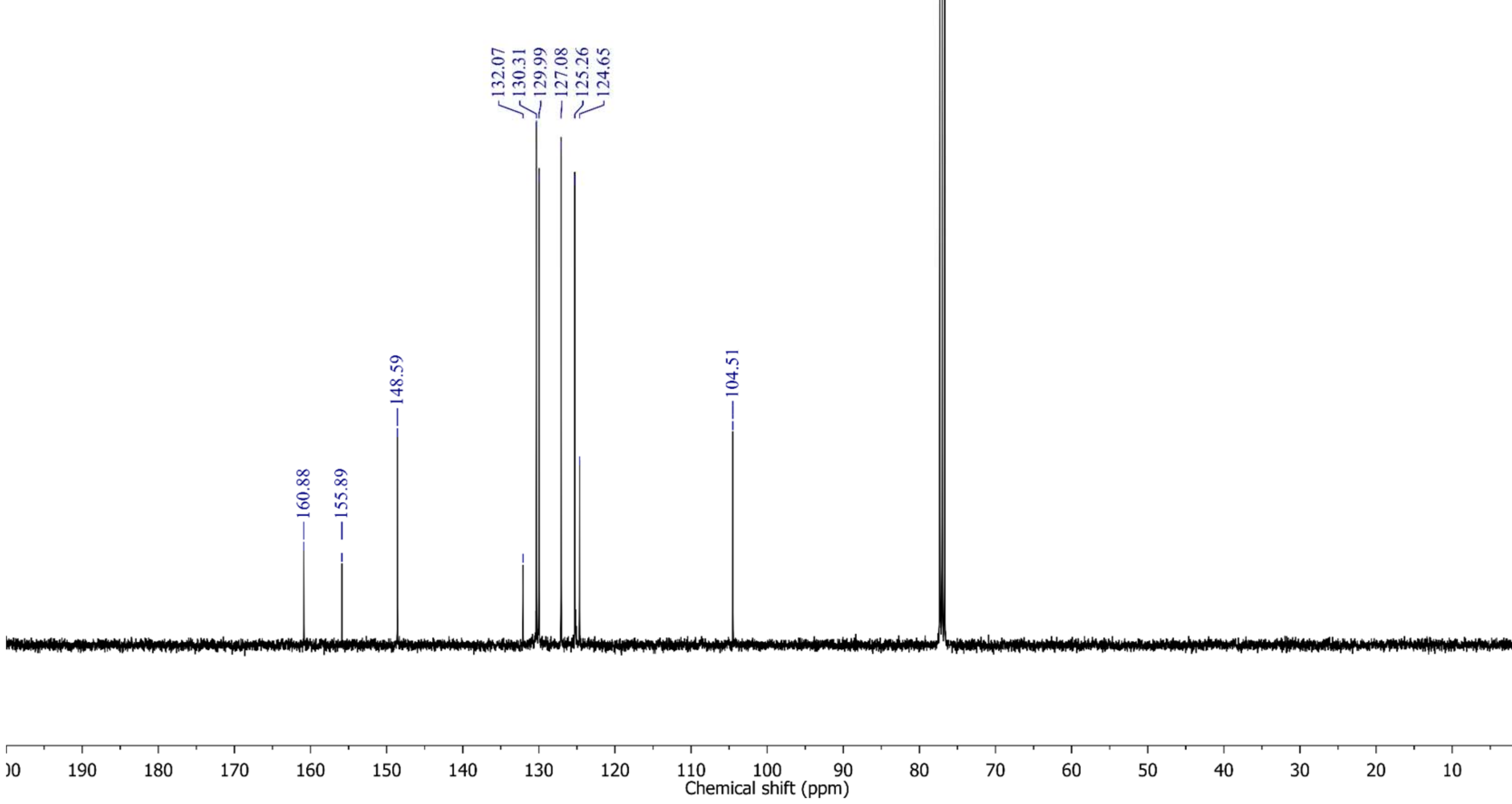

3-(Acridin-9-yl)-5-chloroisoxazole (7i), DEPT, $CDCl_3$, 100 MHz

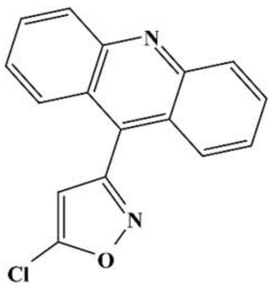

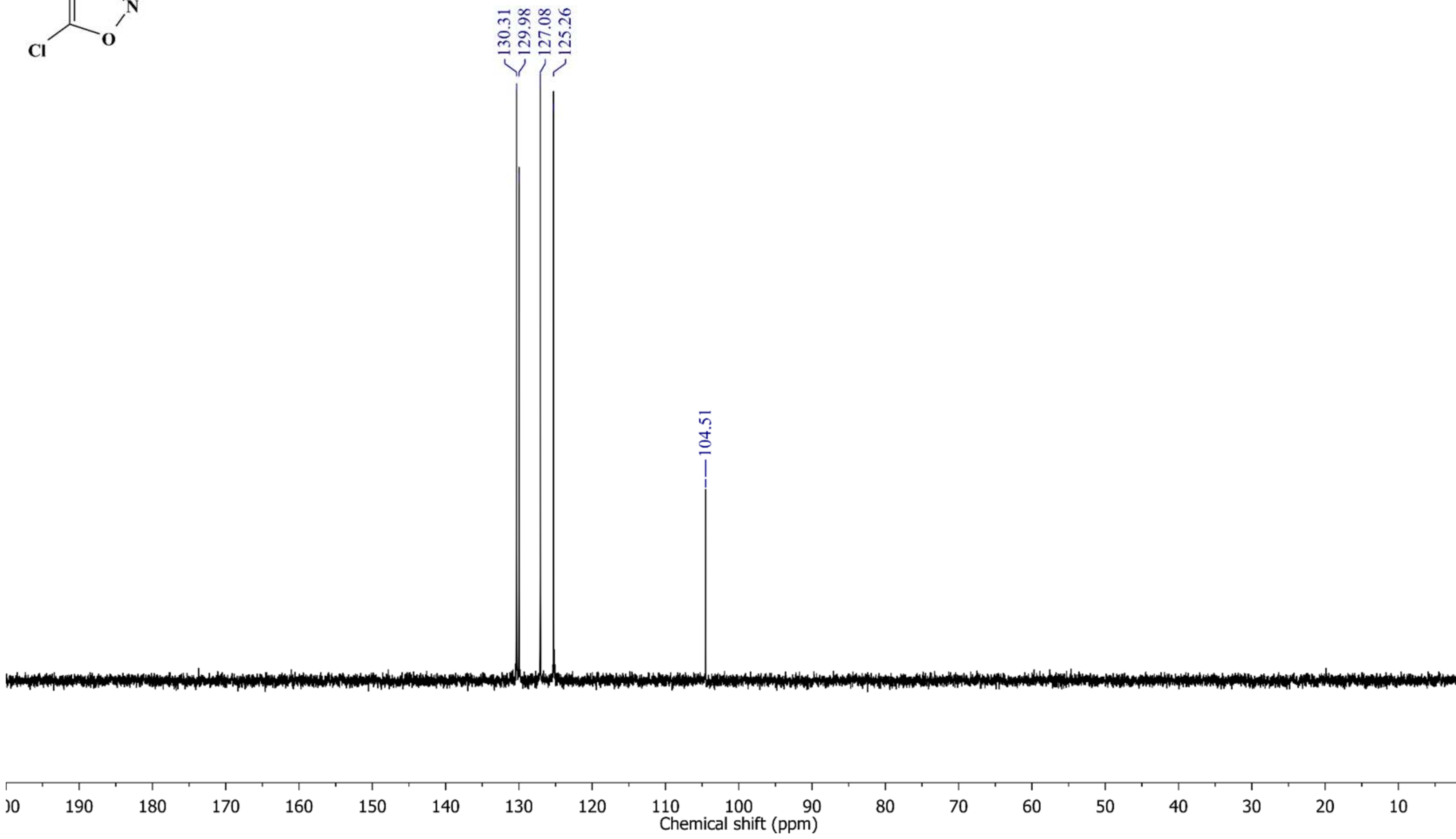

5-Chloro-3-(9-phenylacridin-2-yl)isoxazole (7j), ¹H NMR, CDCl₃, 400 MHz

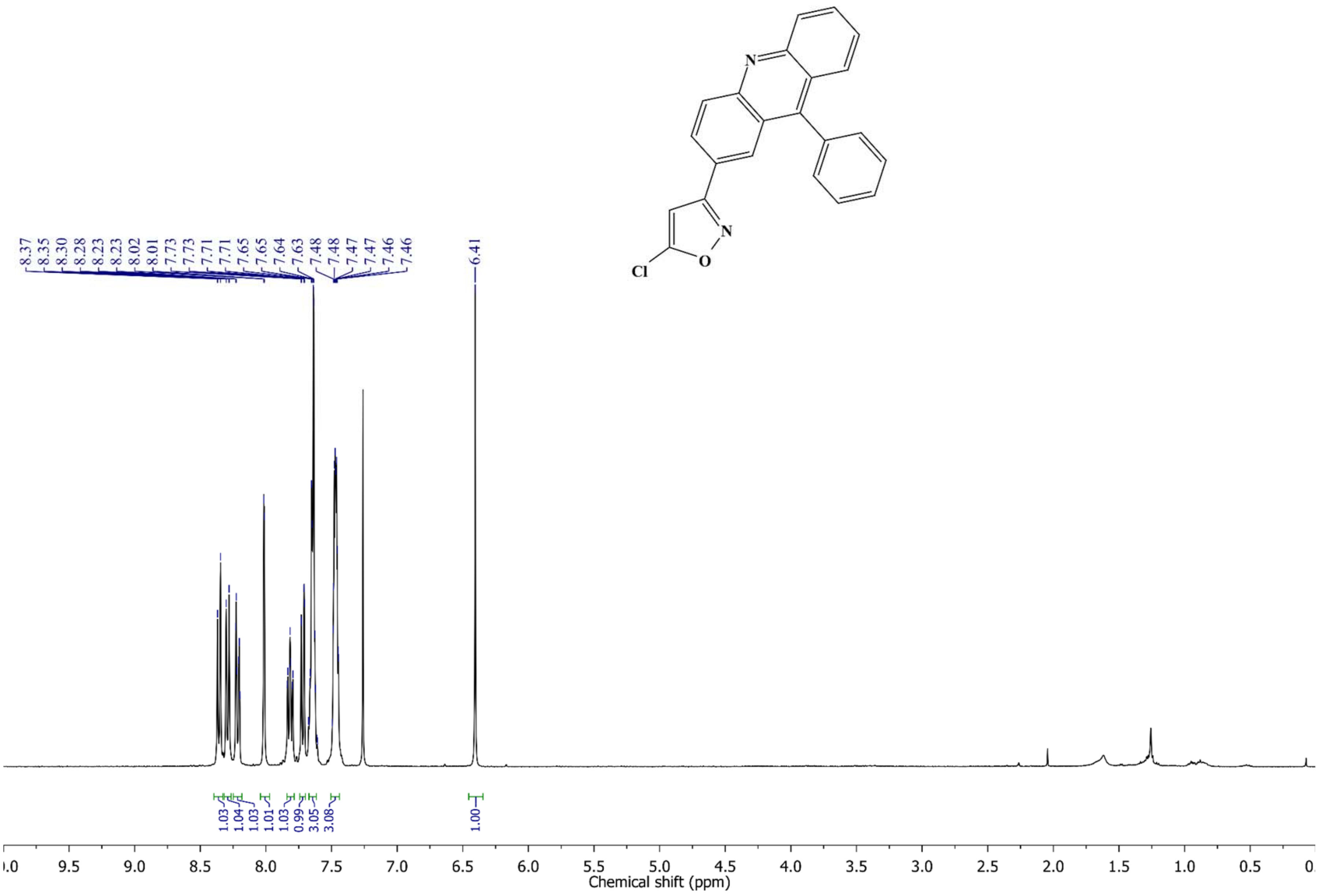

5-Chloro-3-(9-phenylacridin-2-yl)isoxazole (7j), $^{13}C\{^{1}H\}$ NMR, $CDCl_3$, 100 MHz

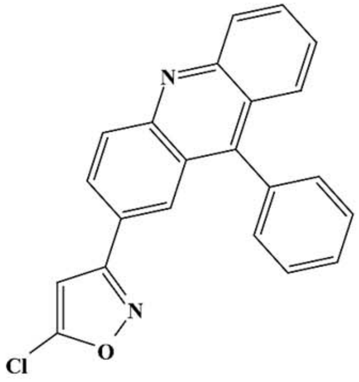

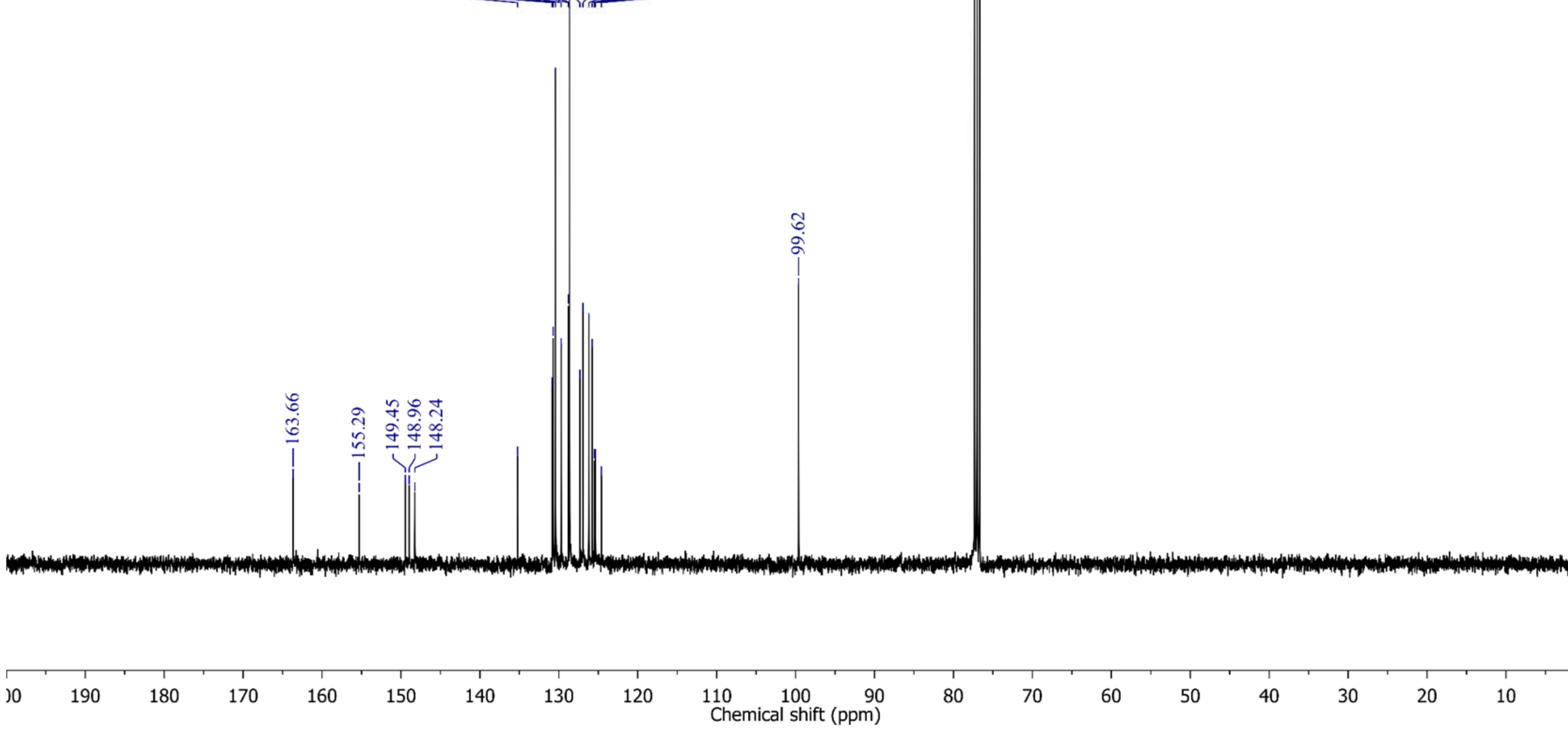

5-Chloro-3-(9-phenylacridin-2-yl)isoxazole (7j), DEPT, $CDCl_3$, 100 MHz

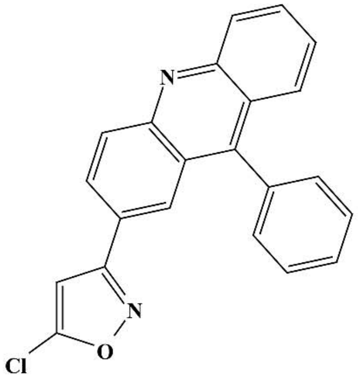

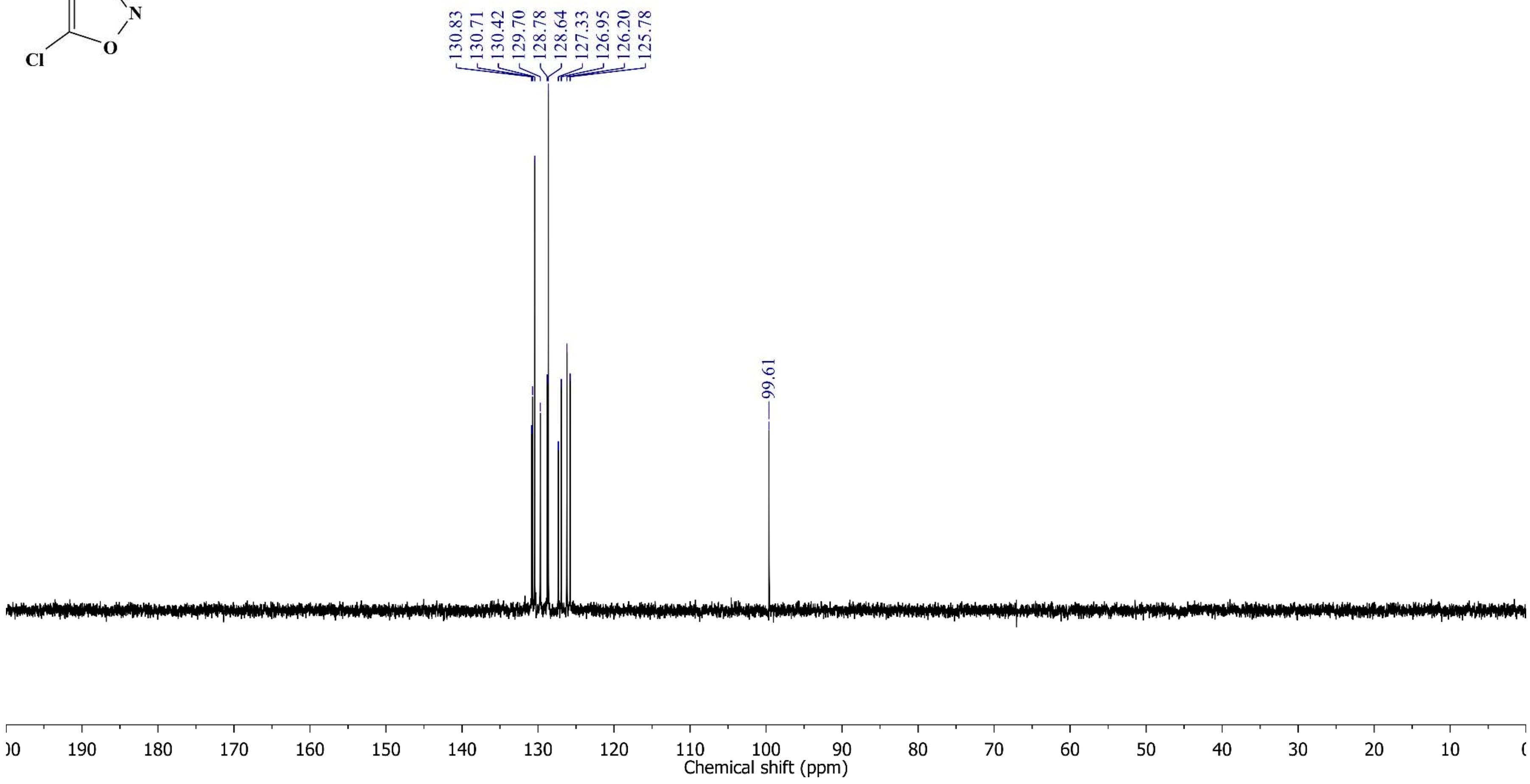

3-(Acridin-9-yl)-5-(*tert*-butoxy)isoxazole (7k), ^{1}H NMR, $CDCl_3$, 400 MHz

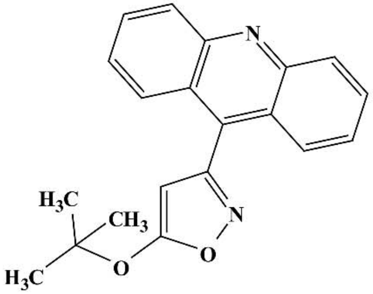

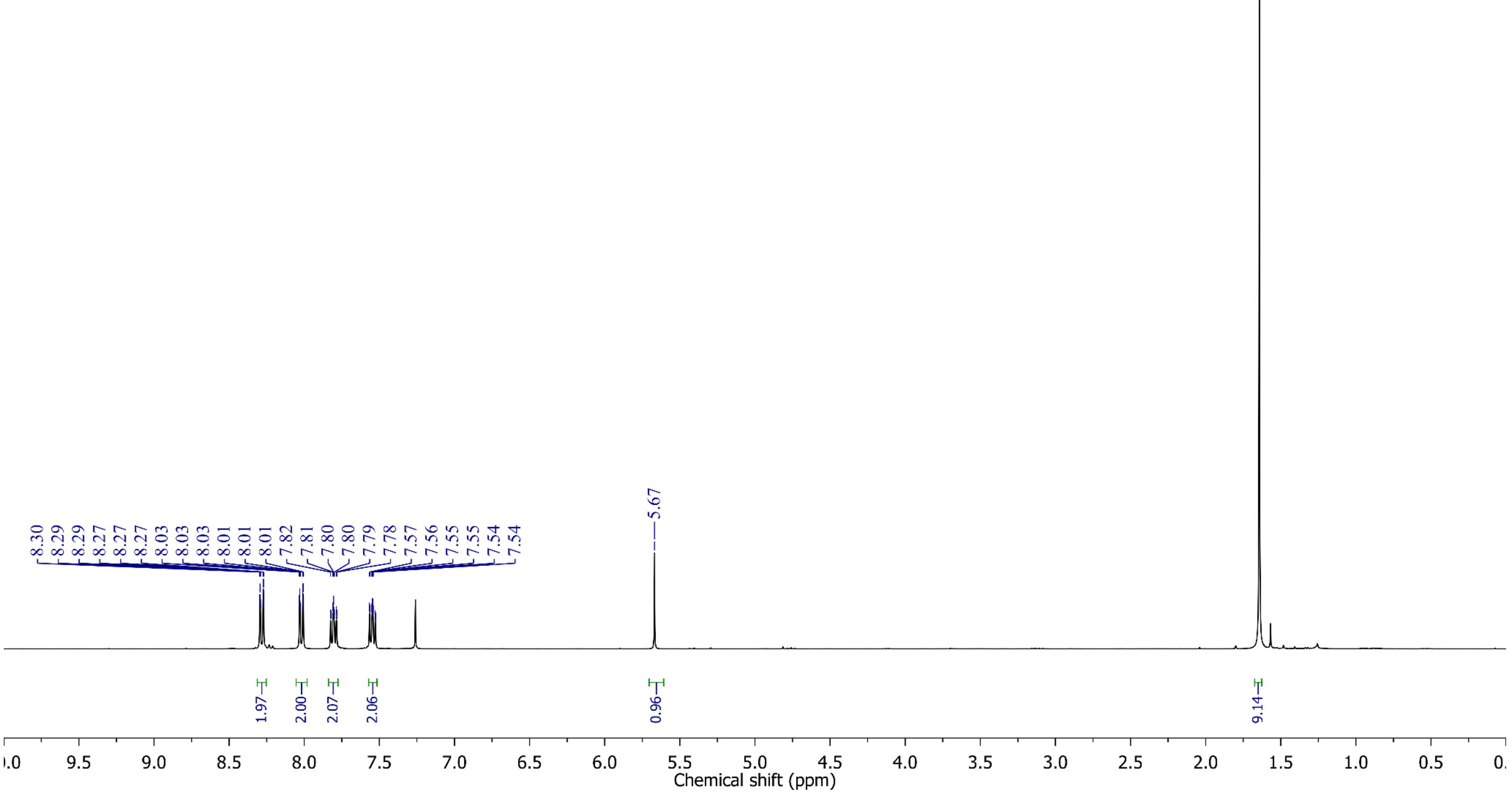

3-(Acridin-9-yl)-5-(*tert*-butoxy)isoxazole (7k), $^{13}C\{^1H\}$ NMR, $CDCl_3$, 100 MHz

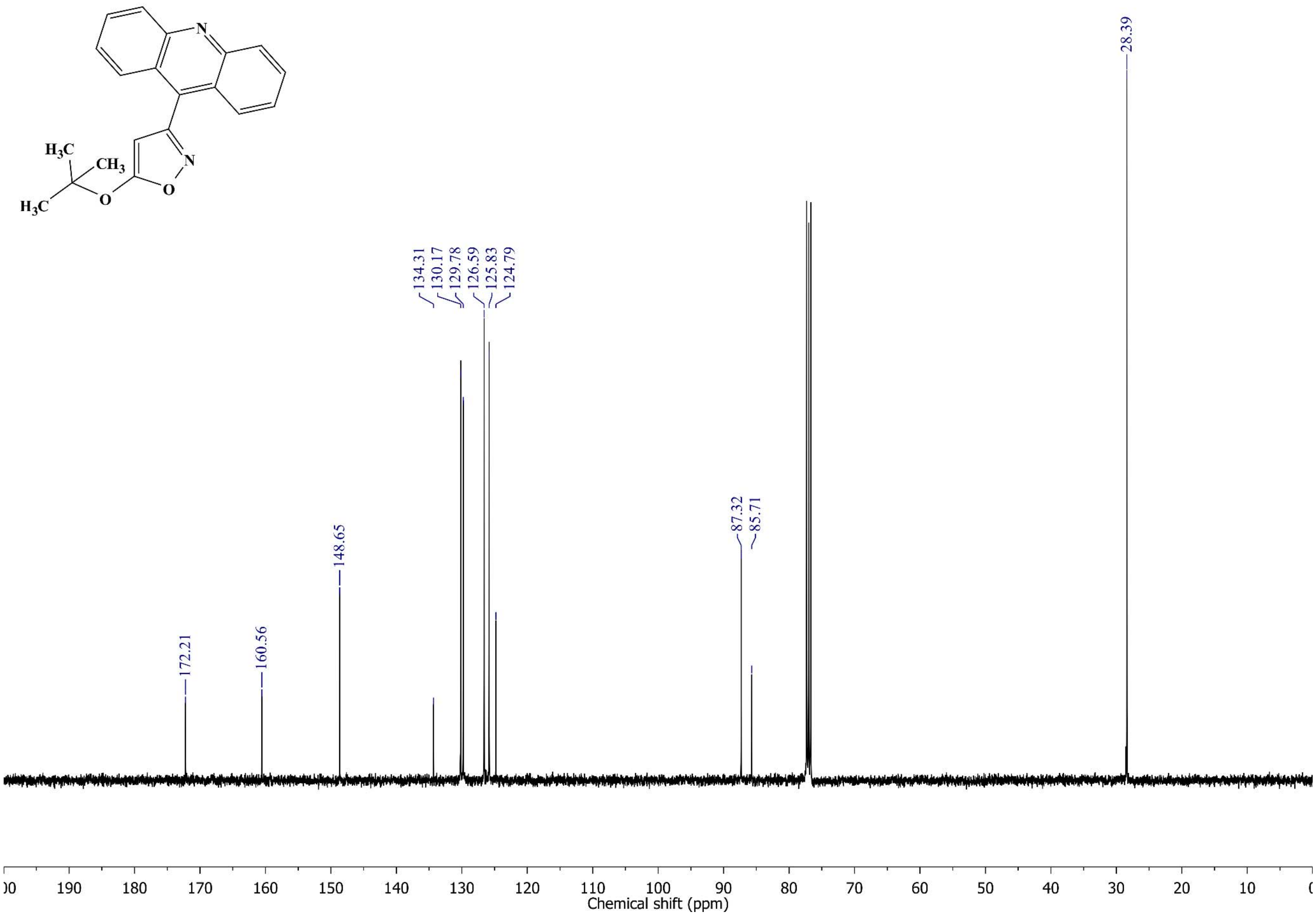

3-(Acridin-9-yl)-5-(*tert*-butoxy)isoxazole (7k), DEPT, $CDCl_3$, 100 MHz

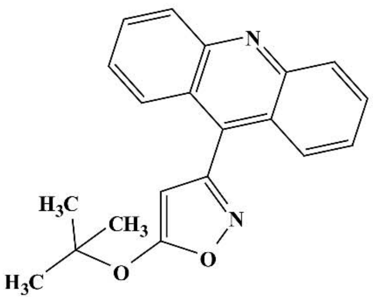

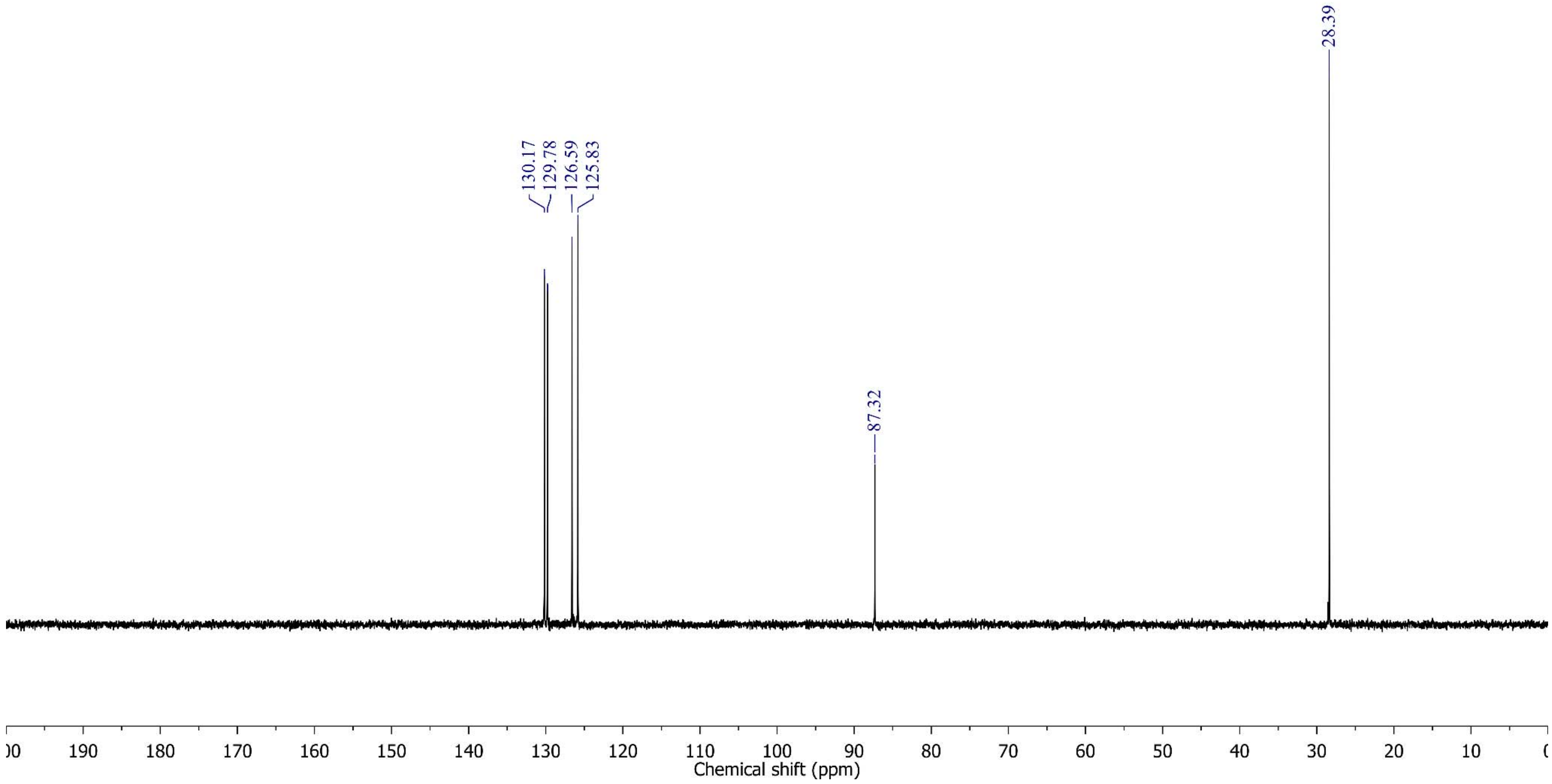

5-(*tert*-Butoxy)-3-(9-phenylacridin-2-yl)isoxazole (7l), ^{1}H NMR, $CDCl_3$, 400 MHz

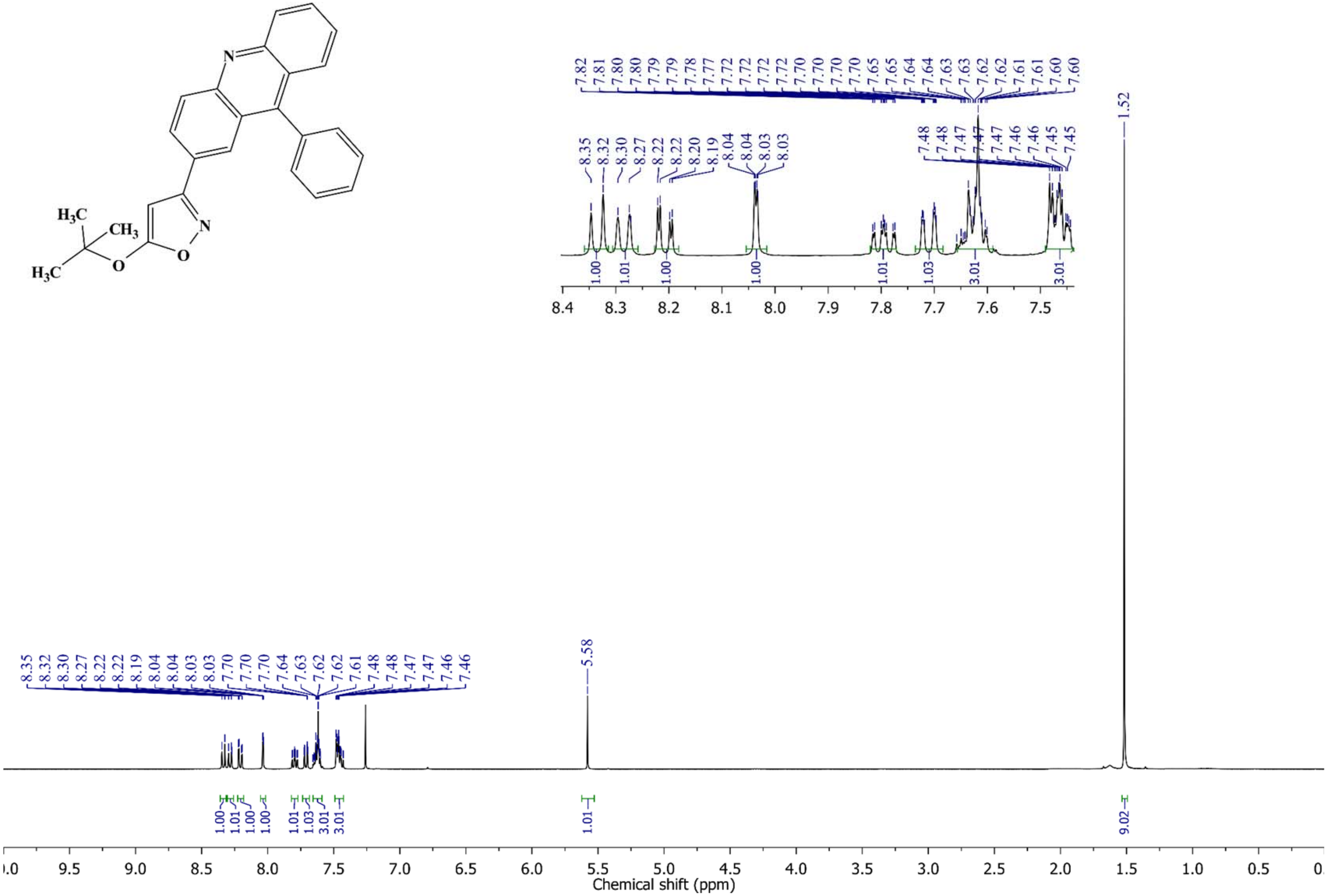

5-(*tert*-Butoxy)-3-(9-phenylacridin-2-yl)isoxazole (7l), $^{13}C\{^1H\}$ NMR, $CDCl_3$, 100 MHz

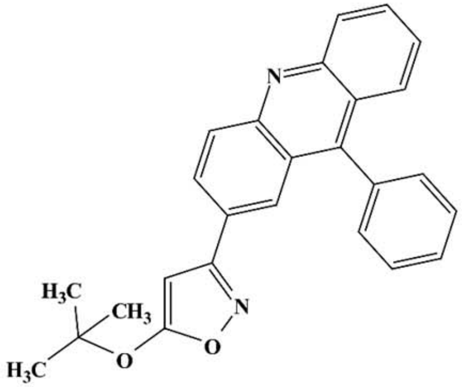

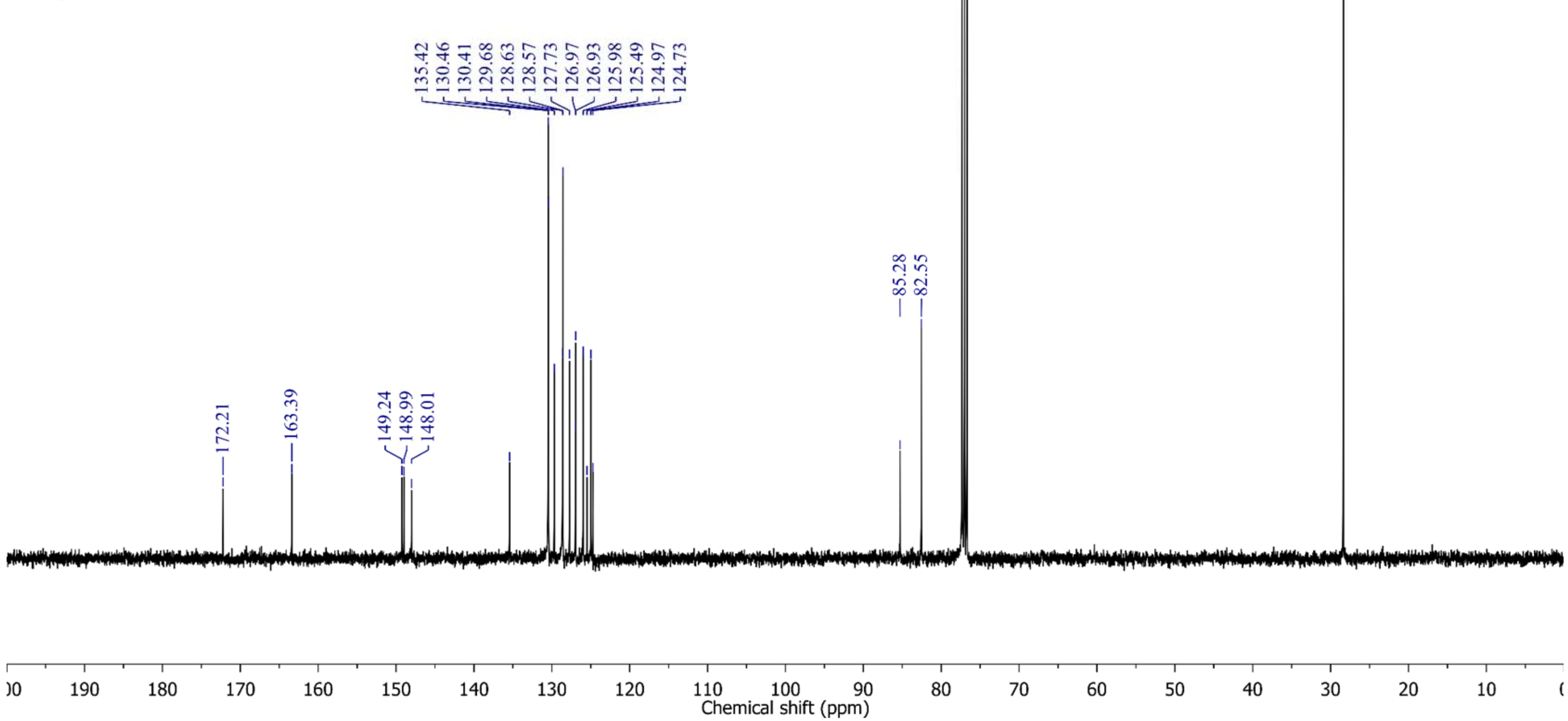

5-(*tert*-Butoxy)-3-(9-phenylacridin-2-yl)isoxazole (7l), DEPT, $CDCl_3$, 100 MHz

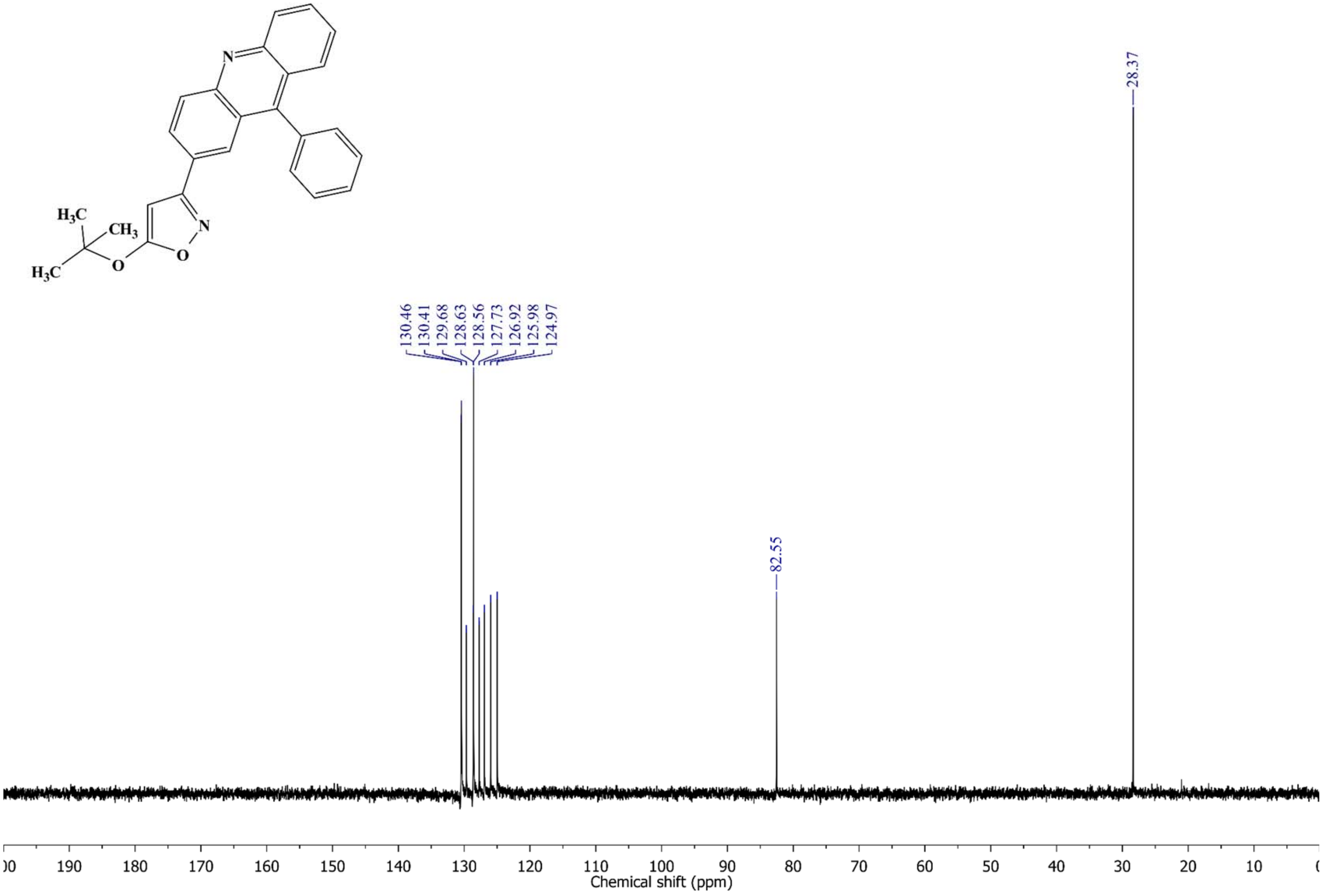

3-(2-Methylacridin-9-yl)-4,5-dihydroisoxazole-5-carbonitrile (10), ^{1}H NMR, $CDCl_3$, 400 MHz

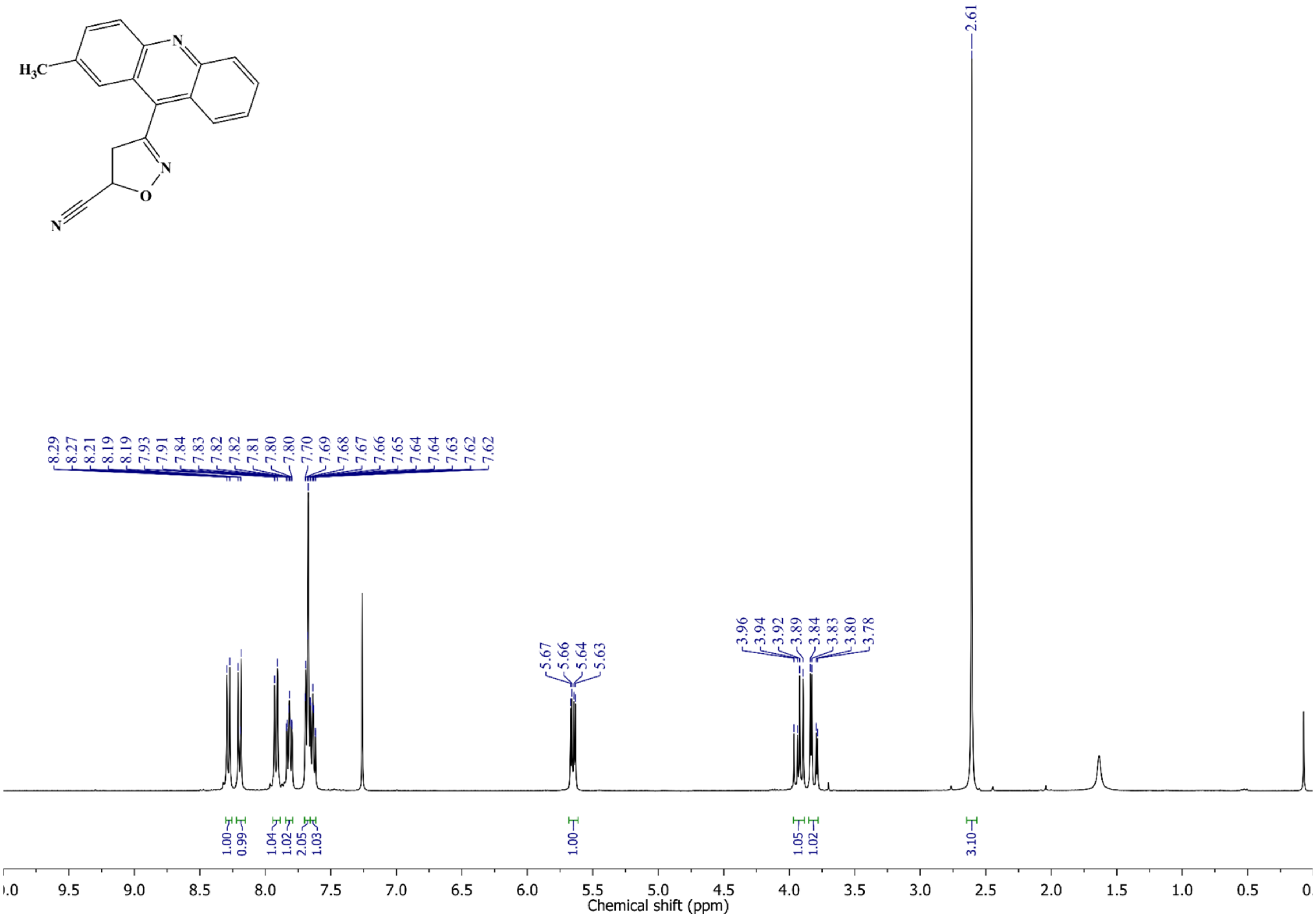

3-(2-Methylacridin-9-yl)-4,5-dihydroisoxazole-5-carbonitrile (10), $^{13}C\{^{1}H\}$ NMR, $CDCl_3$, 100 MHz

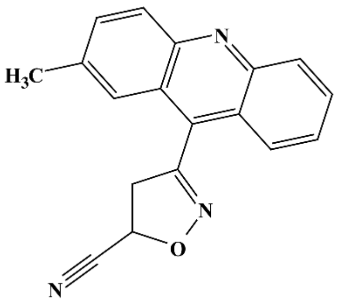

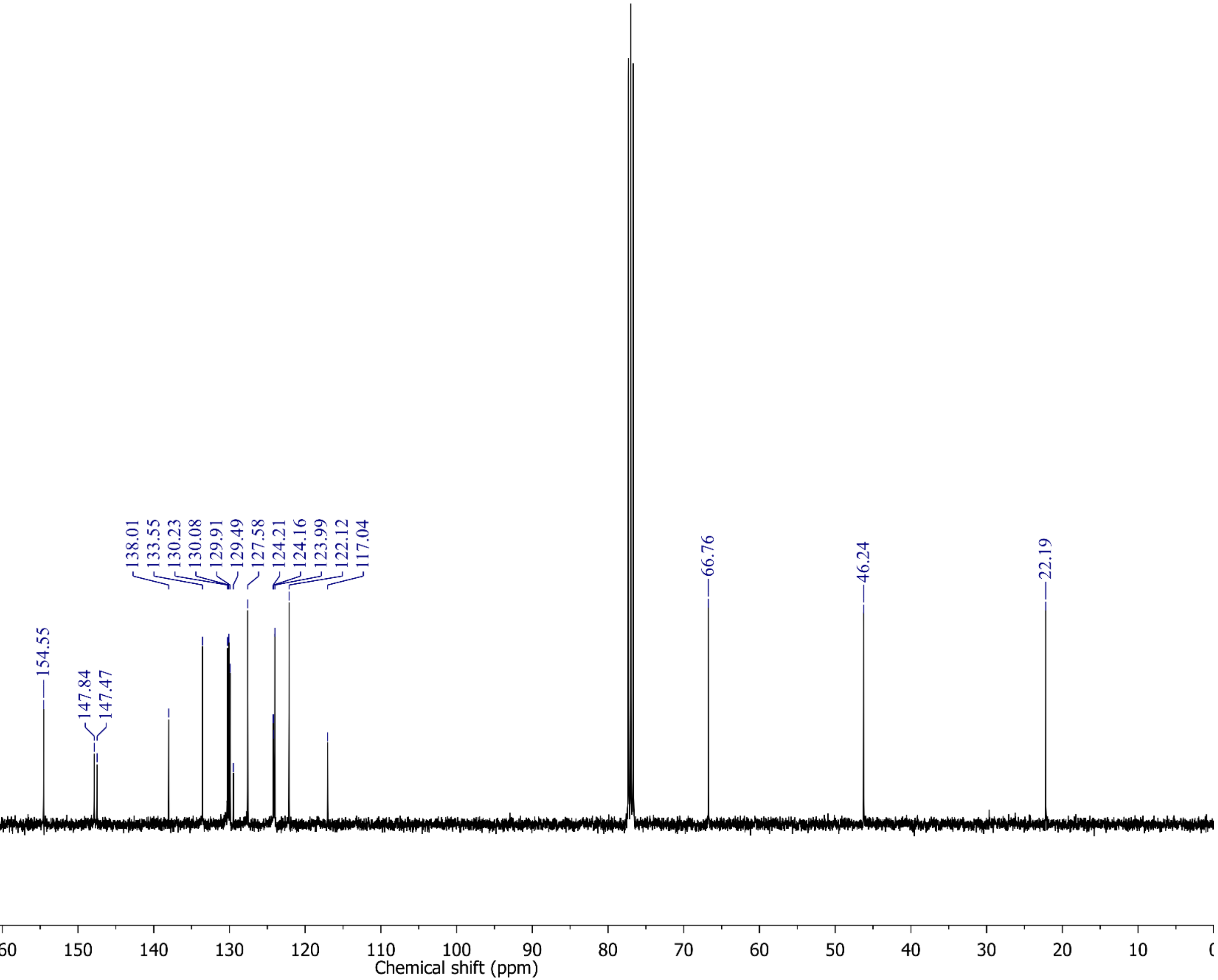

3-(2-Methylacridin-9-yl)-4,5-dihydroisoxazole-5-carbonitrile (10), DEPT, $CDCl_3$, 100 MHz

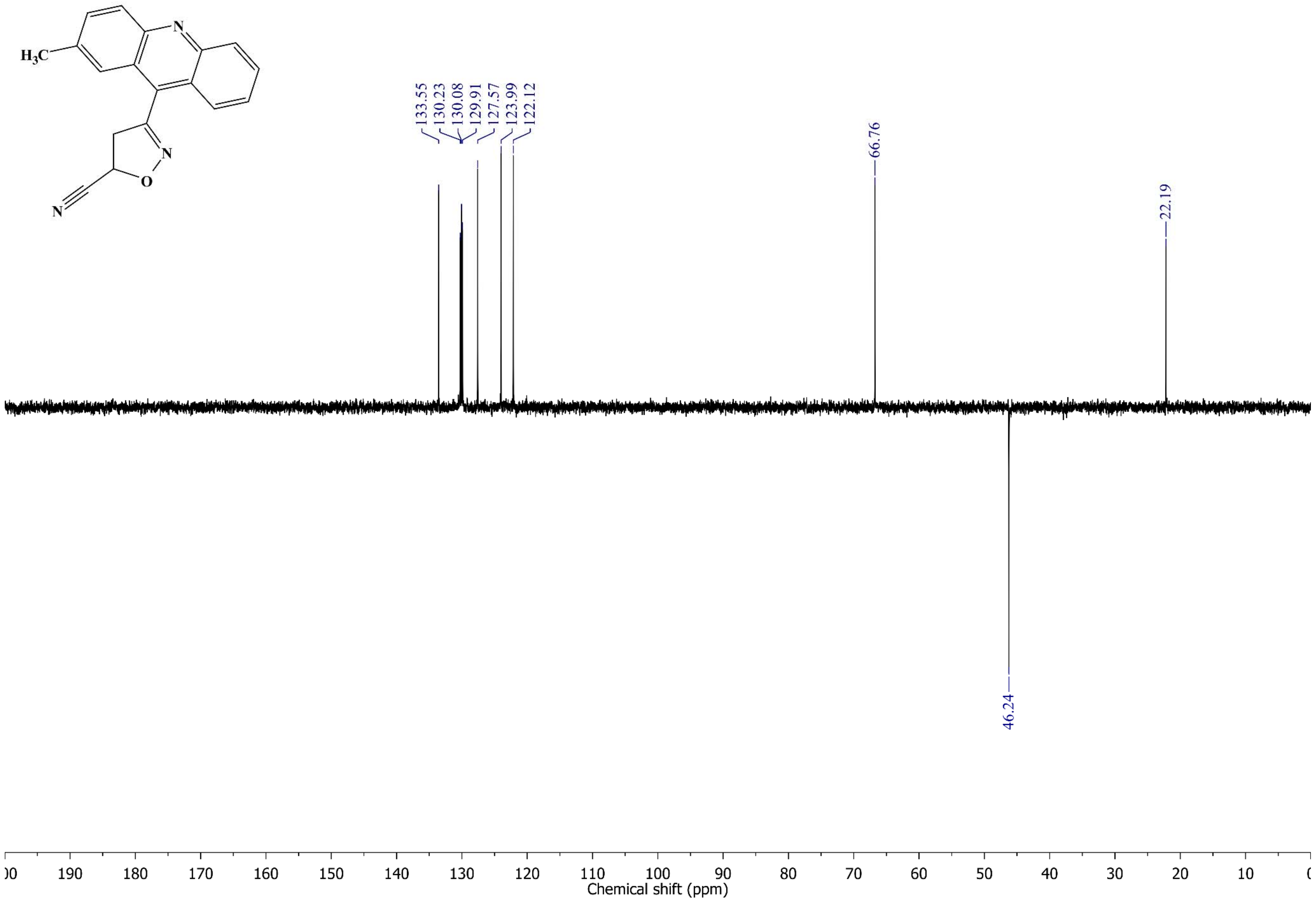

tert-Butyl 3-(9-phenylacridin-2-yl)-2_H_-azirine-2-carboxylate (11a), ^{1}H NMR, $CDCl_3$, 400 MHz

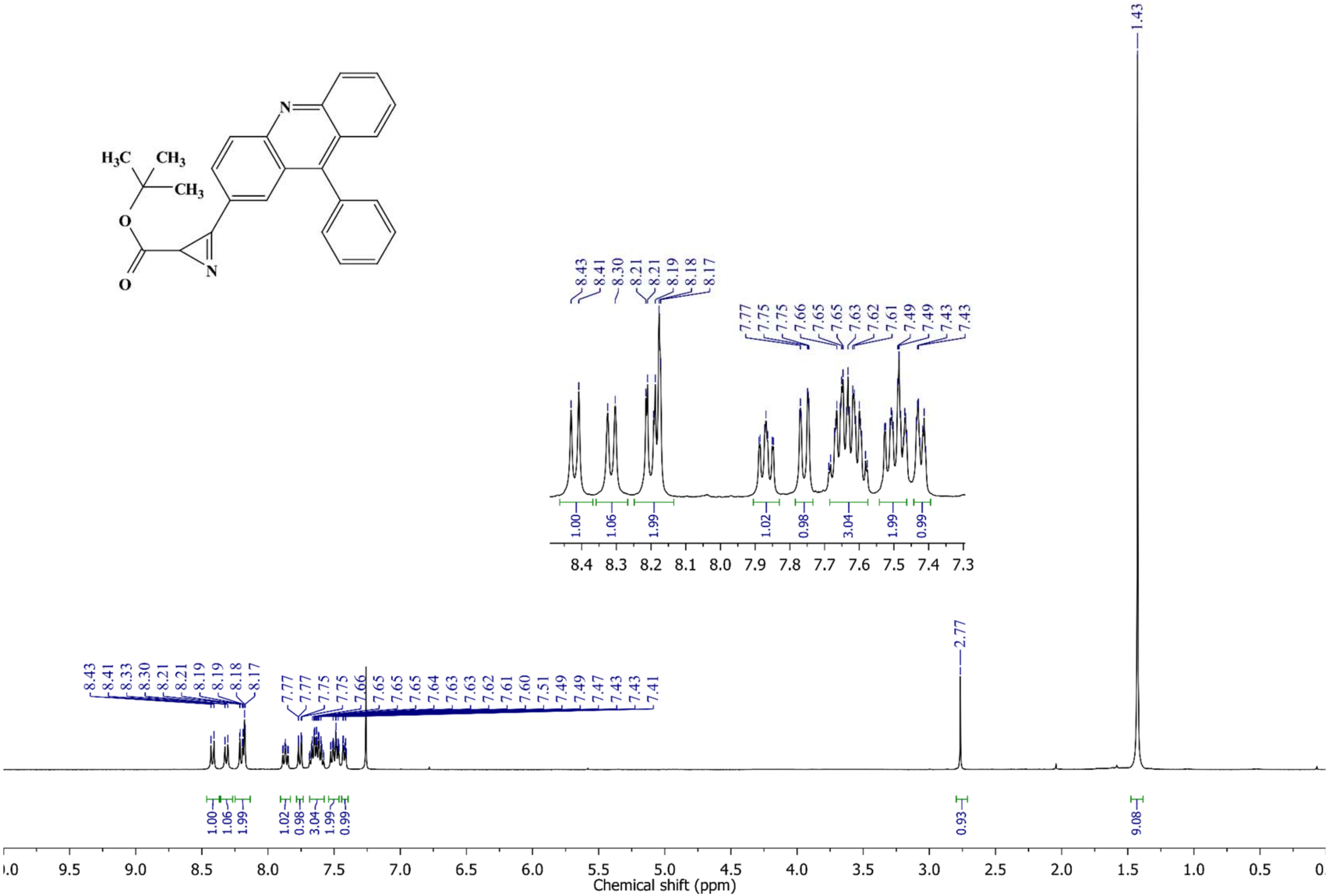

***tert*-Butyl 3-(9-phenylacridin-2-yl)-2*H*-azirine-2-carboxylate (11a), $^{13}C\{^1H\}$ NMR, $CDCl_3$, 100 MHz**

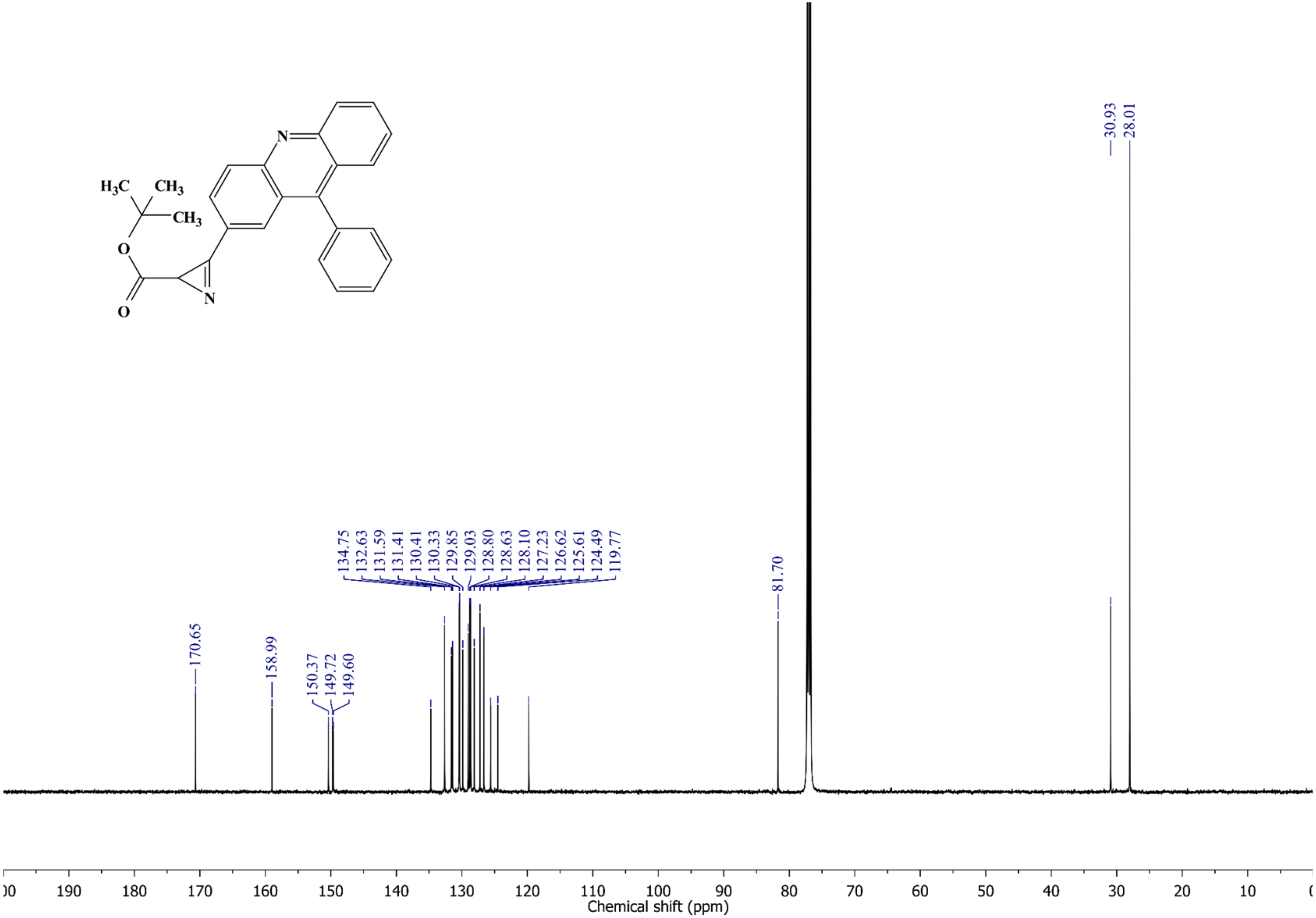

***tert*-Butyl 3-(9-phenylacridin-2-yl)-2*H*-azirine-2-carboxylate (11a), DEPT, $CDCl_3$, 100 MHz**

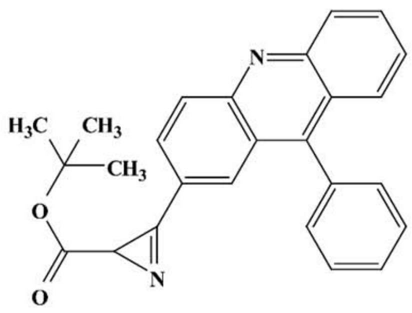

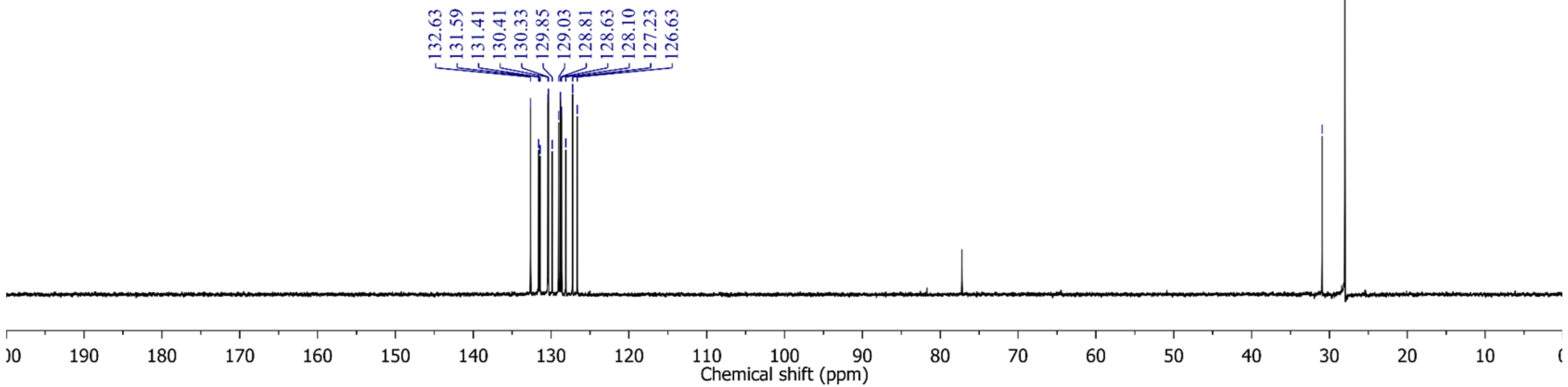

9-Phenyl-2-vinylacridine (26), ^{1}H NMR, $CDCl_3$, 400 MHz

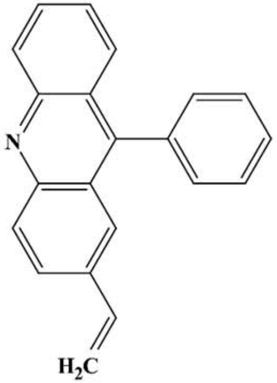

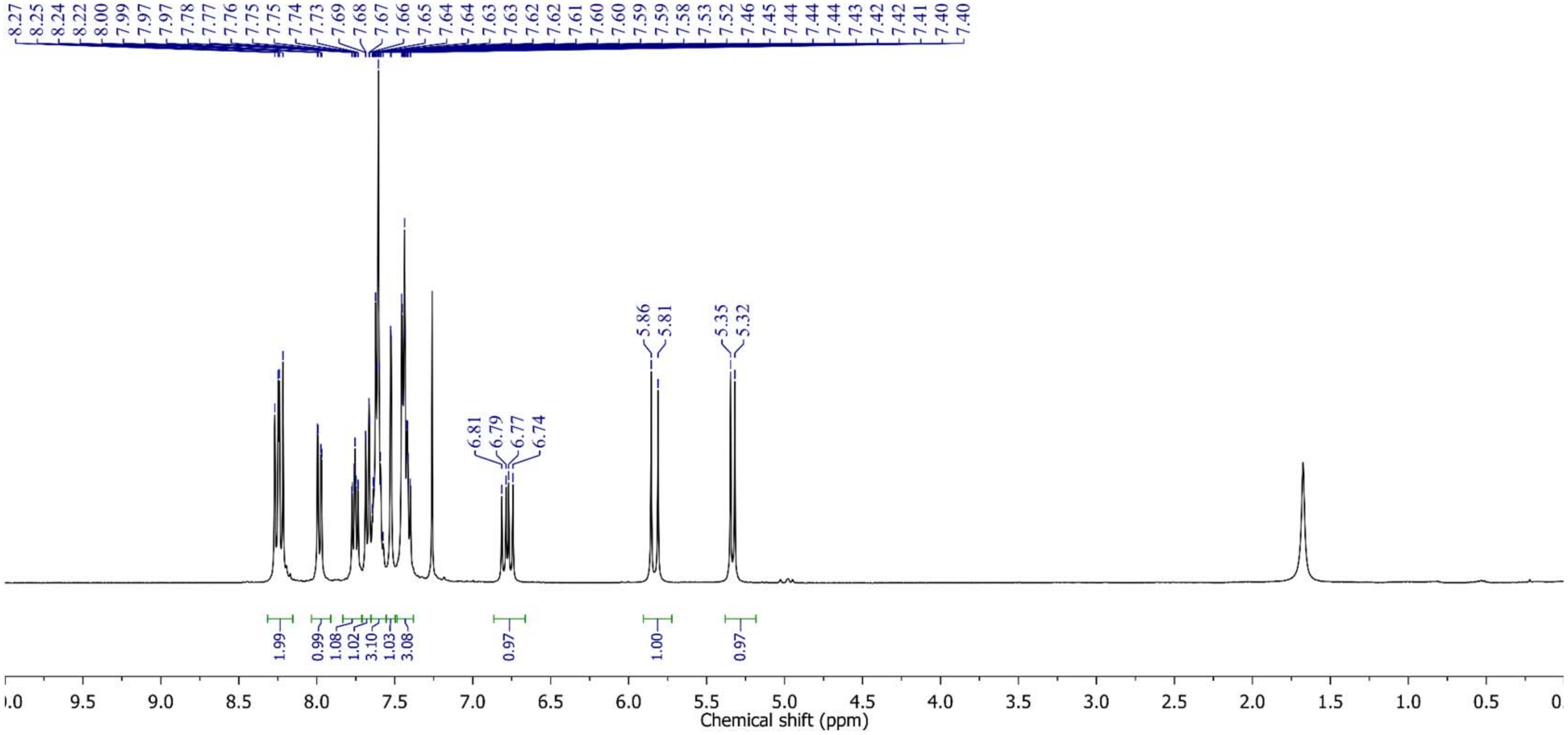

9-Phenyl-2-vinylacridine (26), $^{13}C\{^1H\}$ NMR, $CDCl_3$, 100 MHz

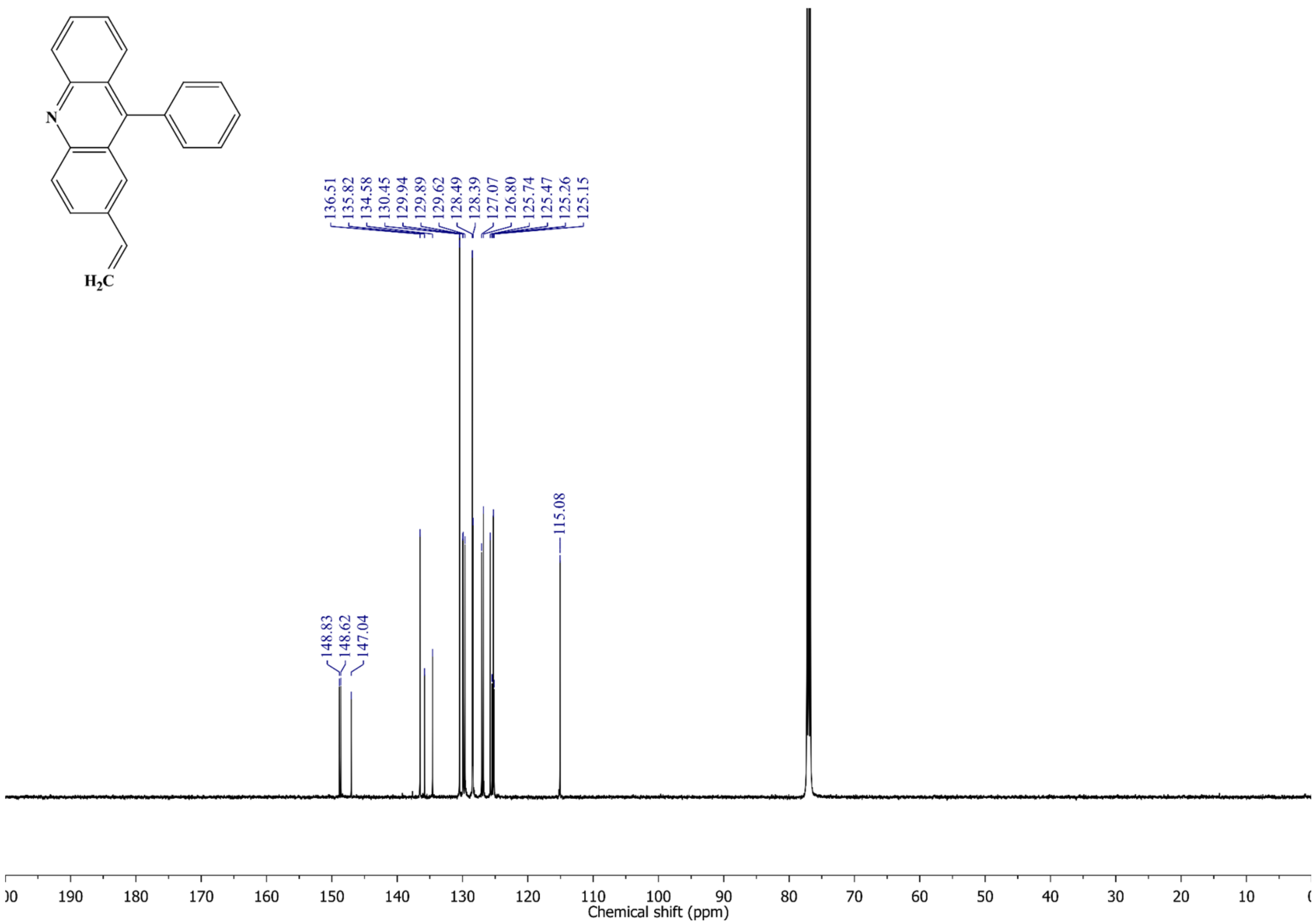

9-Phenyl-2-vinylacridine (26), DEPT, CDCl$_3$, 100 MHz

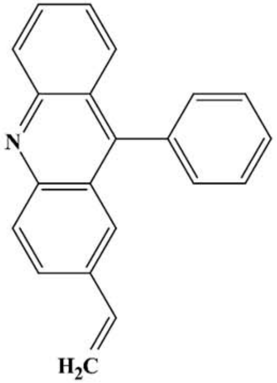

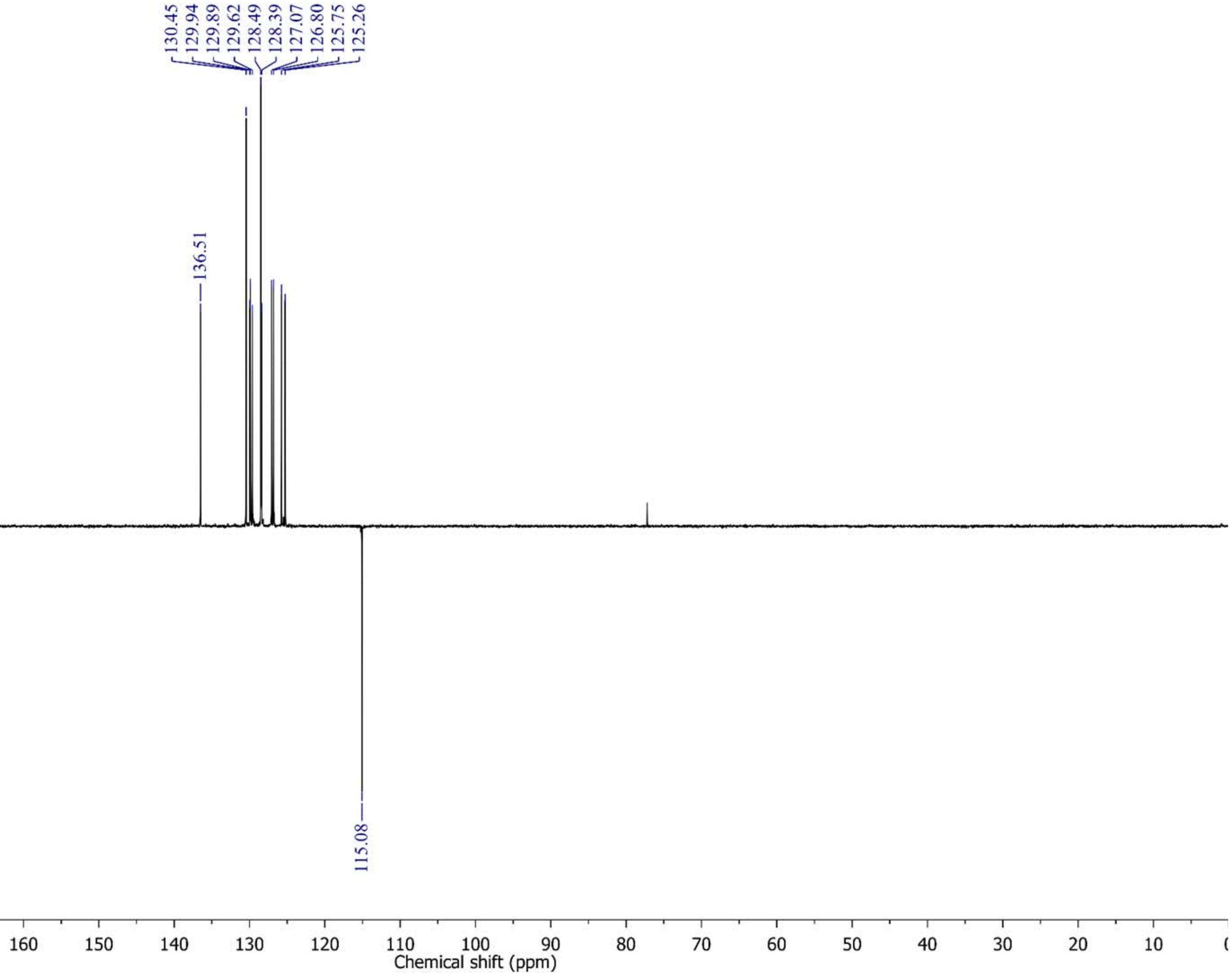

2-(1,2-Dibromoethyl)-9-phenylacridine (27), ^{1}H NMR, $CDCl_3$, 400 MHz

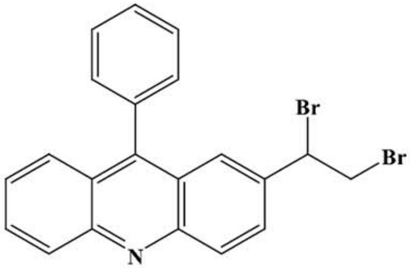

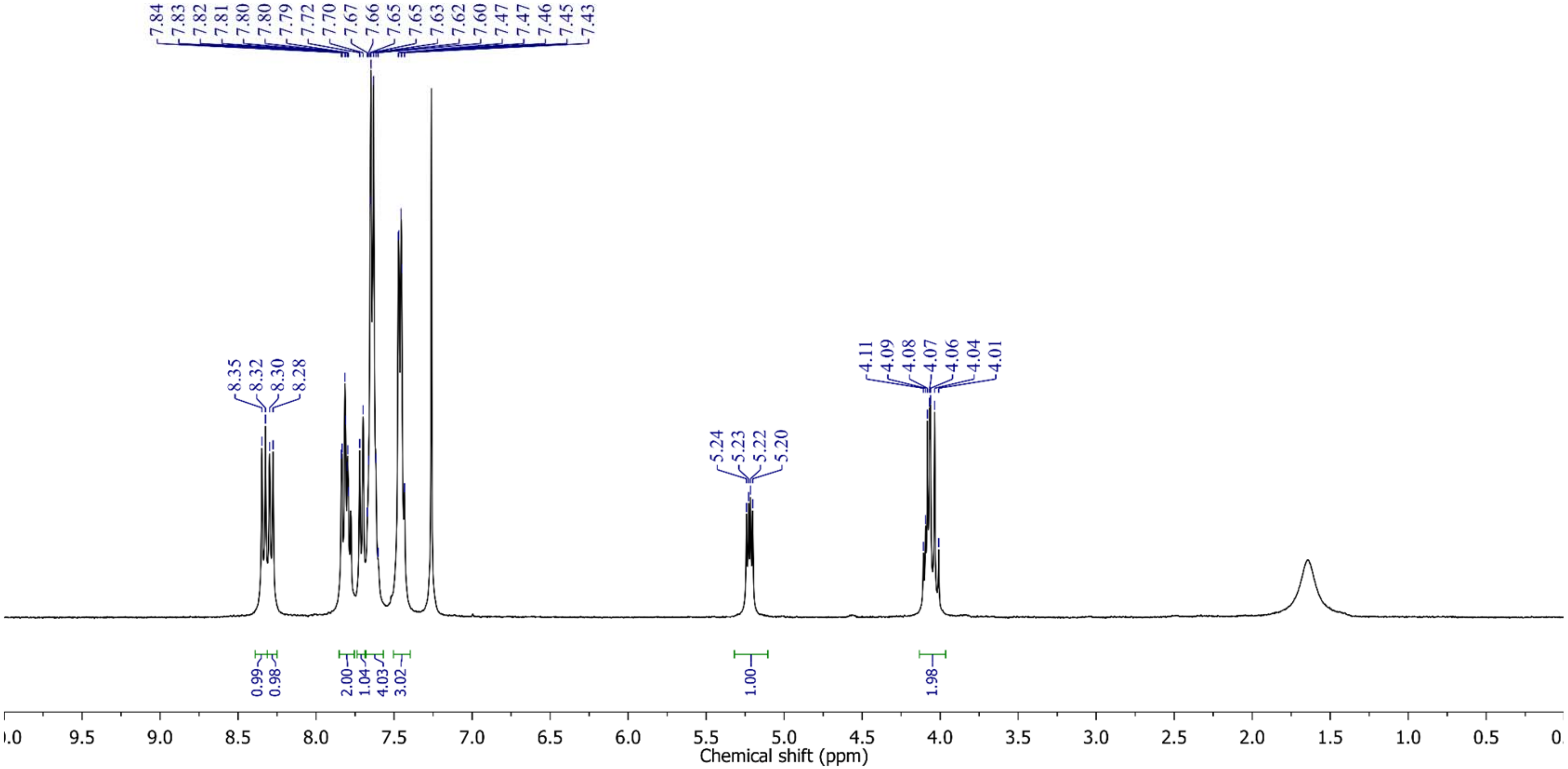

2-(1,2-Dibromoethyl)-9-phenylacridine (27), $^{13}C\{^{1}H\}$ NMR, $CDCl_3$, 100 MHz

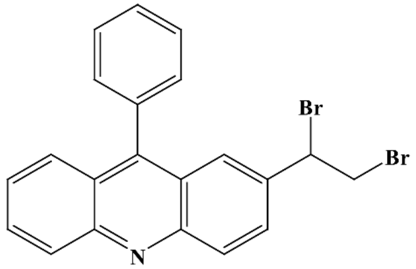

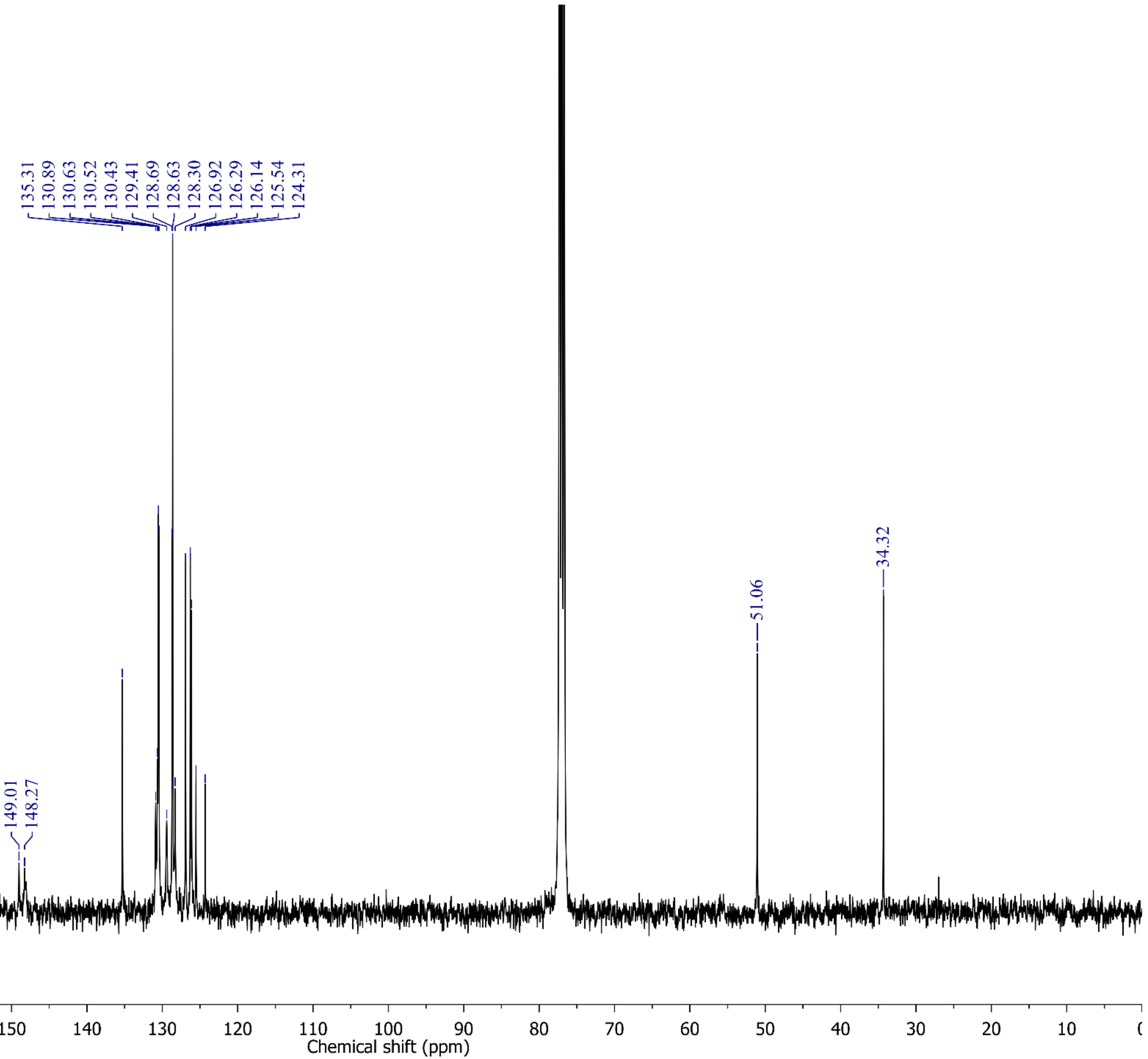

2-(1,2-Dibromoethyl)-9-phenylacridine (27), DEPT, $CDCl_3$, 100 MHz

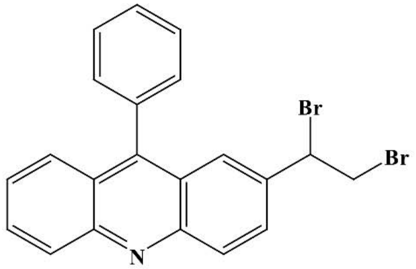

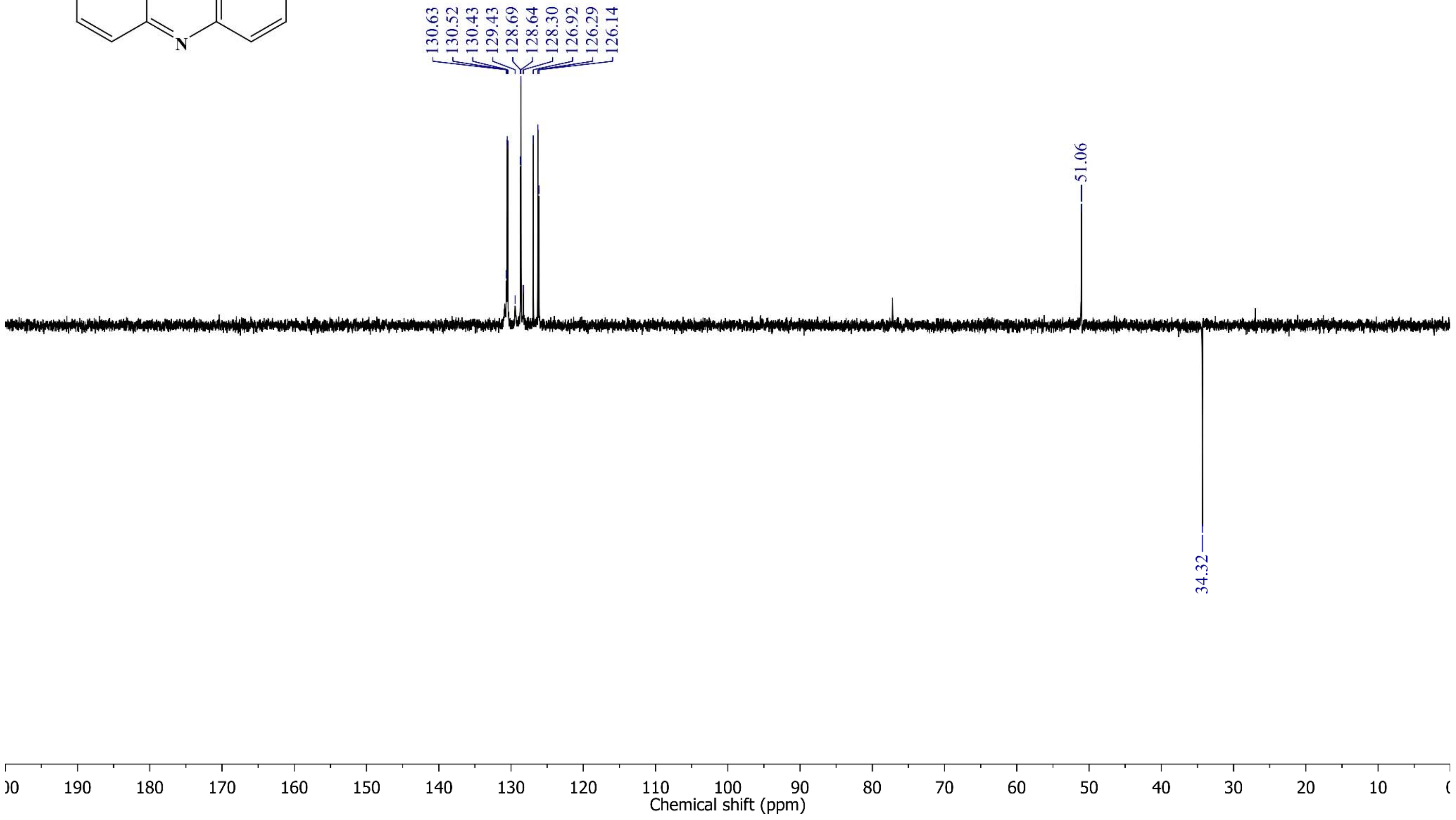

2-(2*H*-Azirin-3-yl)-9-phenylacridine (28), ^{1}H NMR, $CDCl_3$, 400 MHz

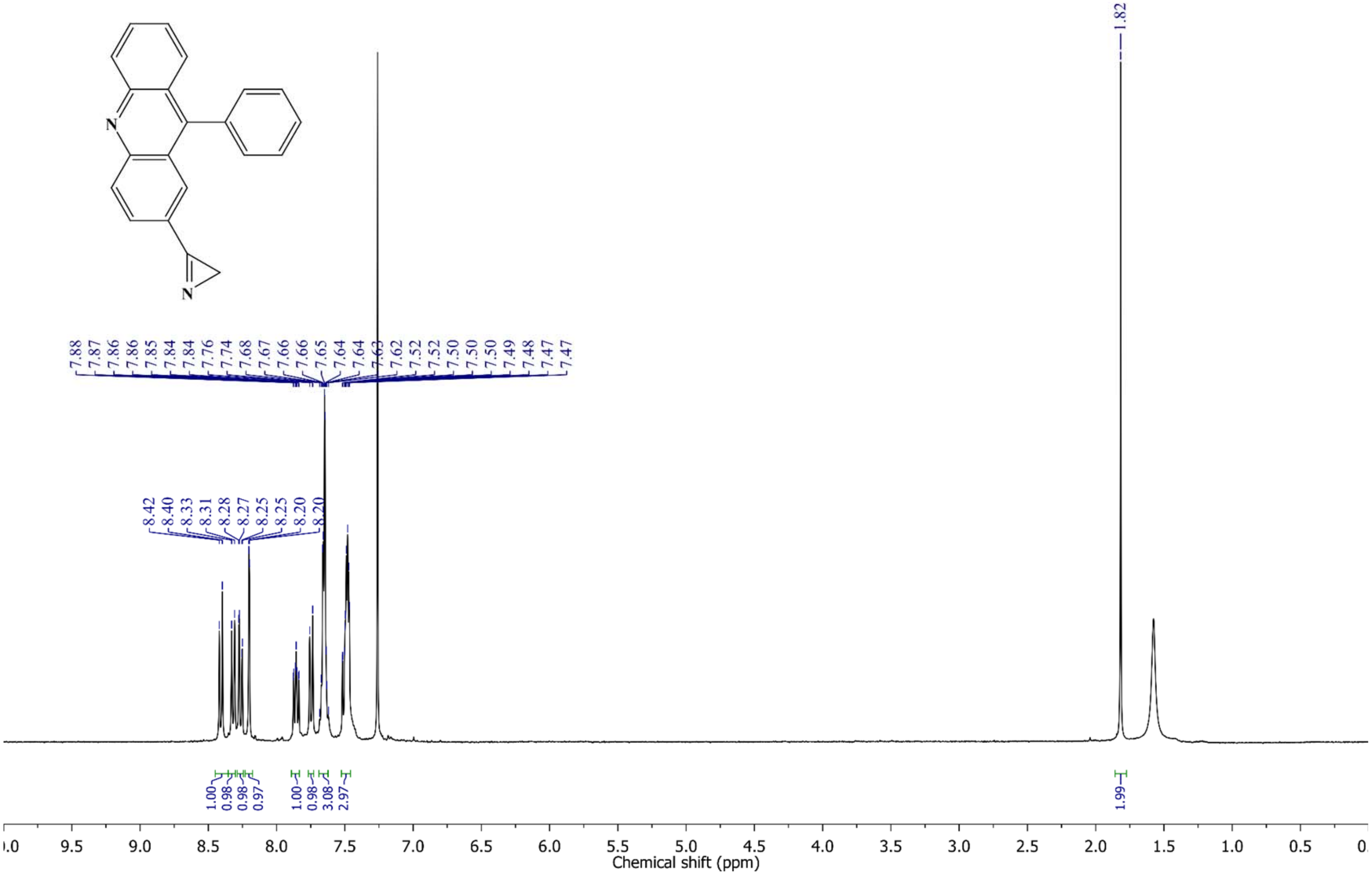

2-(2*H*-Azirin-3-yl)-9-phenylacridine (28), $^{13}C\{^1H\}$ NMR, $CDCl_3$, 100 MHz

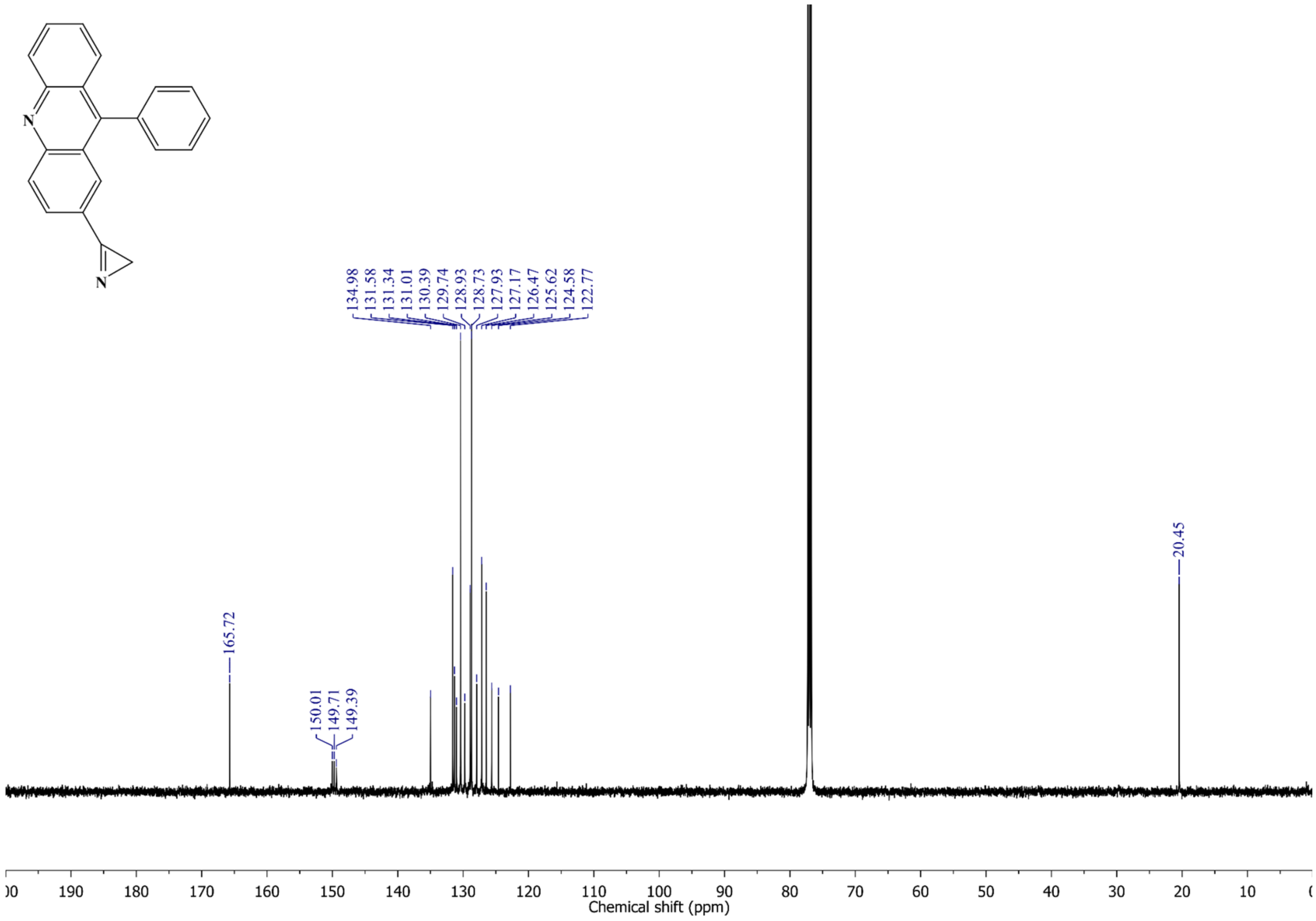

2-(2*H*-Azirin-3-yl)-9-phenylacridine (28), DEPT, $CDCl_3$, 100 MHz

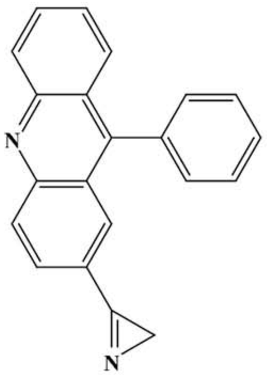

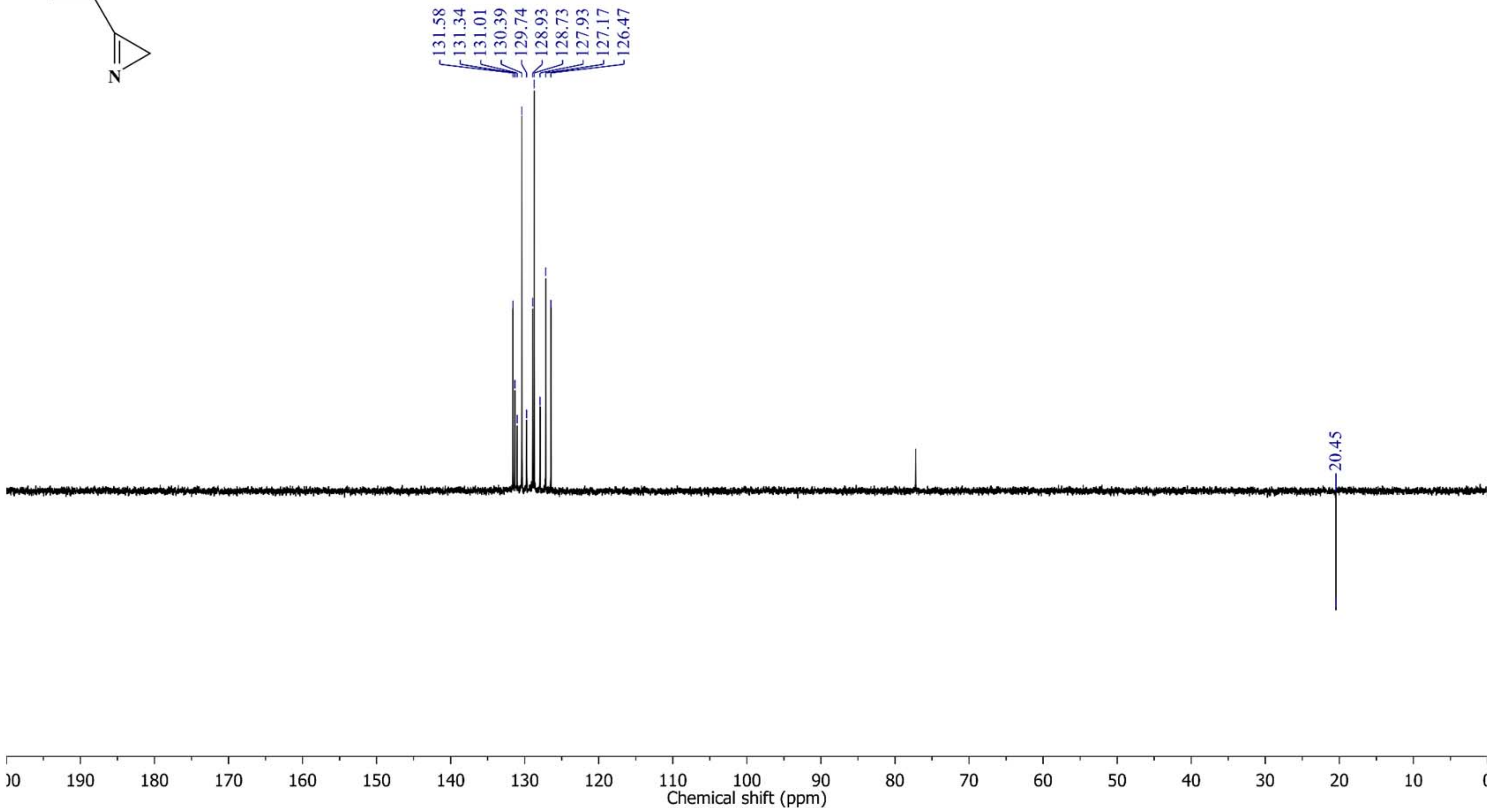

9-(5-(3-Phenyl-2*H*-azirin-2-yl)-1*H*-1,2,3-triazol-1-yl)acridine (32), ^{1}H NMR, $CDCl_3$, 400 MHz

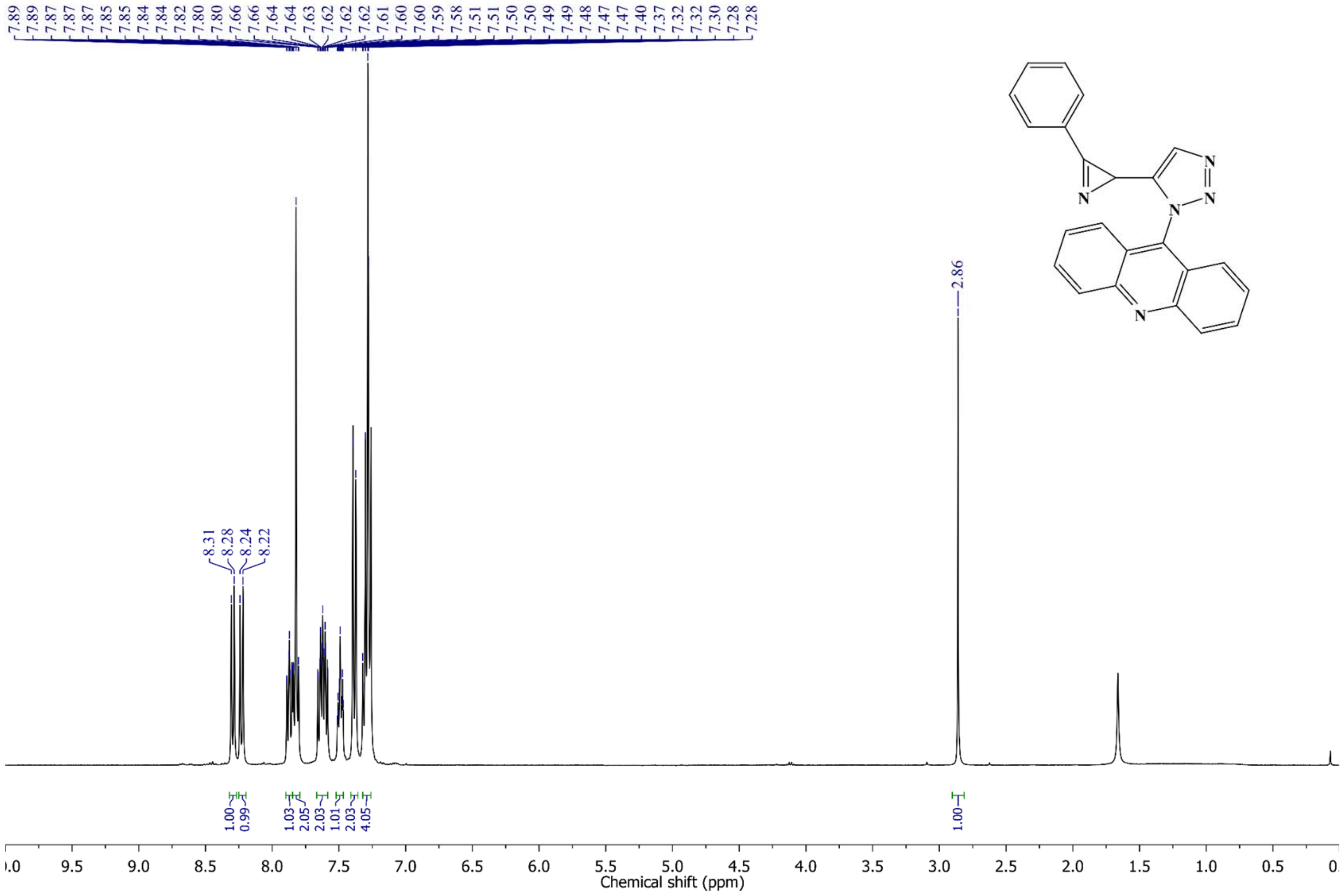

9-(5-(3-Phenyl-2*H*-azirin-2-yl)-1*H*-1,2,3-triazol-1-yl)acridine (32), $^{13}C\{^{1}H\}$ NMR, $CDCl_3$, 100 MHz

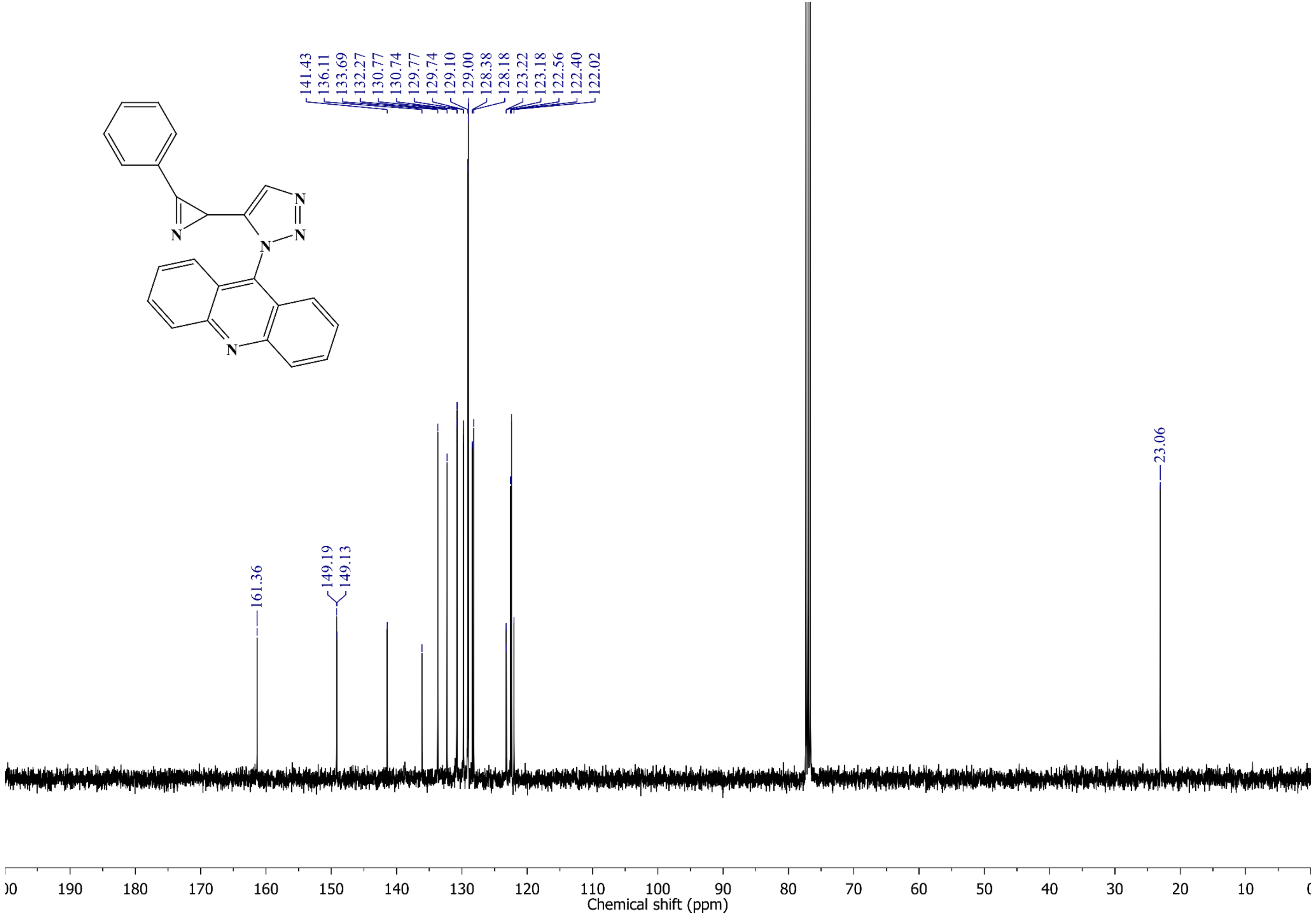

9-(5-(3-Phenyl-2*H*-azirin-2-yl)-1*H*-1,2,3-triazol-1-yl)acridine (32), DEPT, $CDCl_3$, 100 MHz

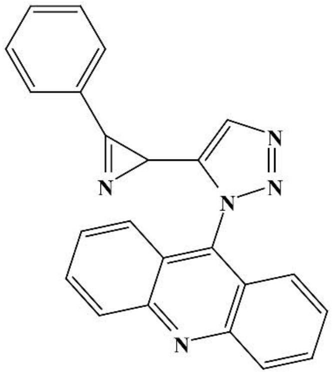

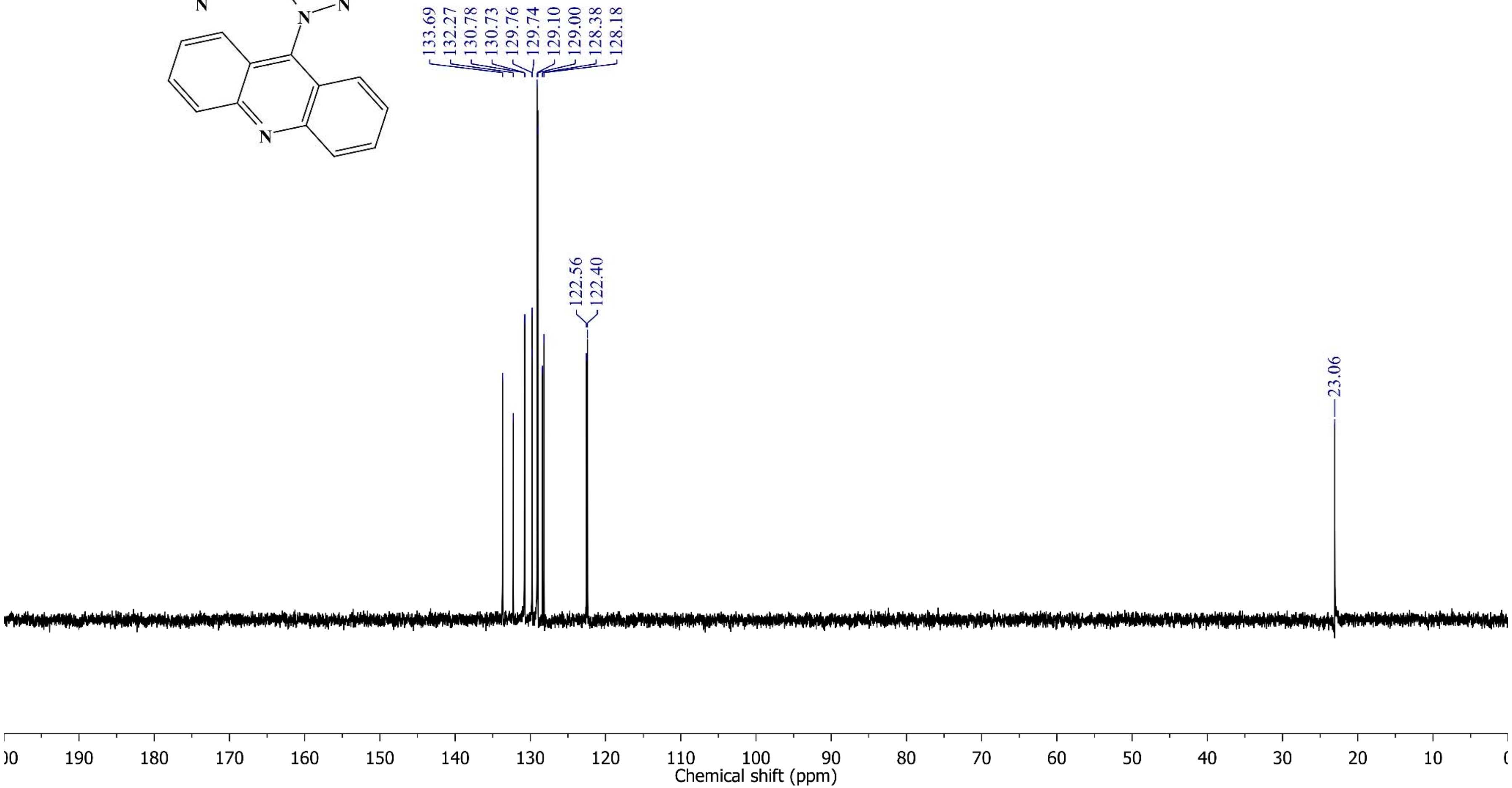

3-(9-Benzyl-9,10-dihydroacridin-9-yl)-5-phenylisoxazole (33a), ^{1}H NMR, $CDCl_3$, 400 MHz

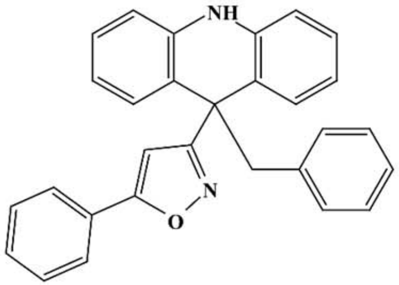

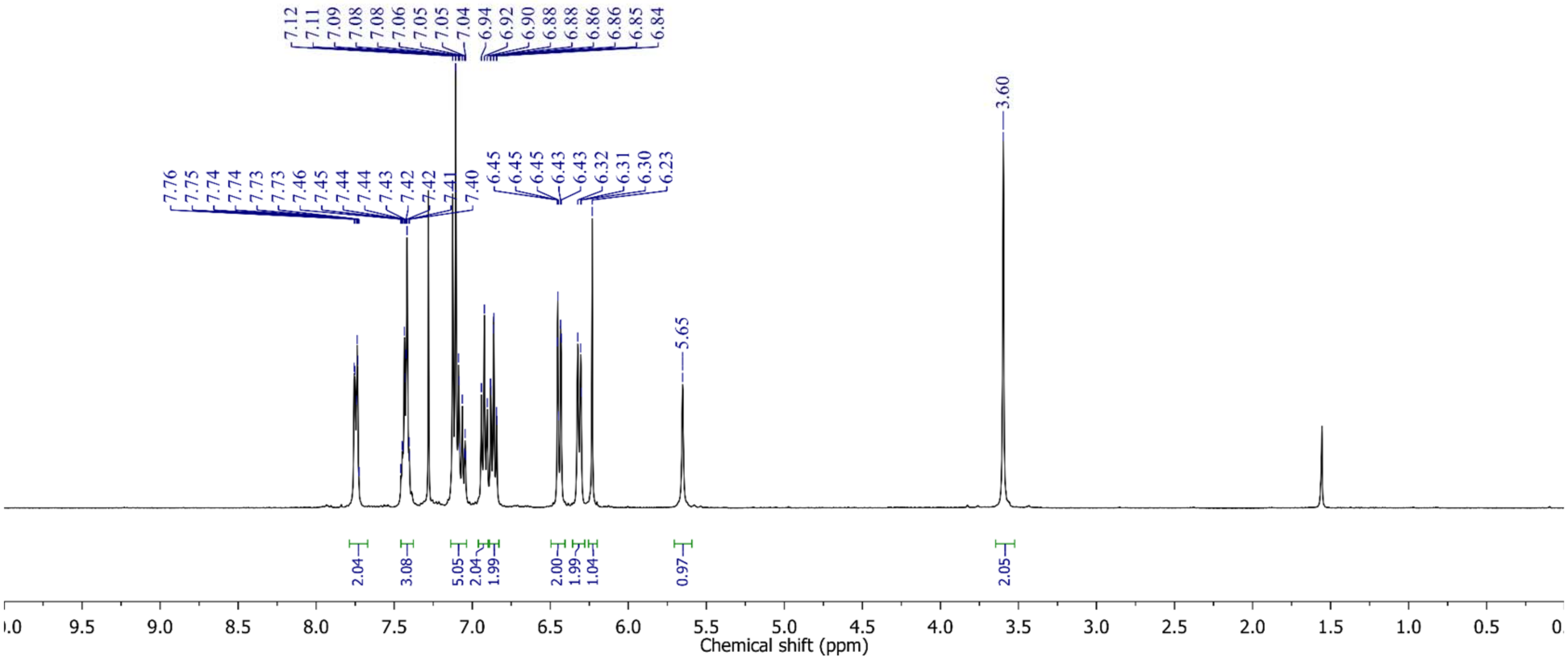

3-(9-Benzyl-9,10-dihydroacridin-9-yl)-5-phenylisoxazole (33a), $^{13}C\{^{1}H\}$ NMR, $CDCl_3$, 100 MHz

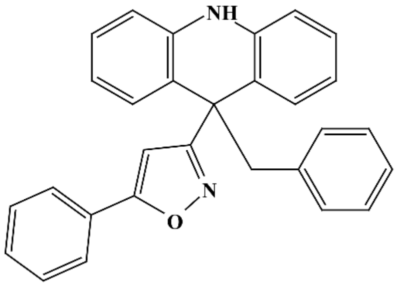

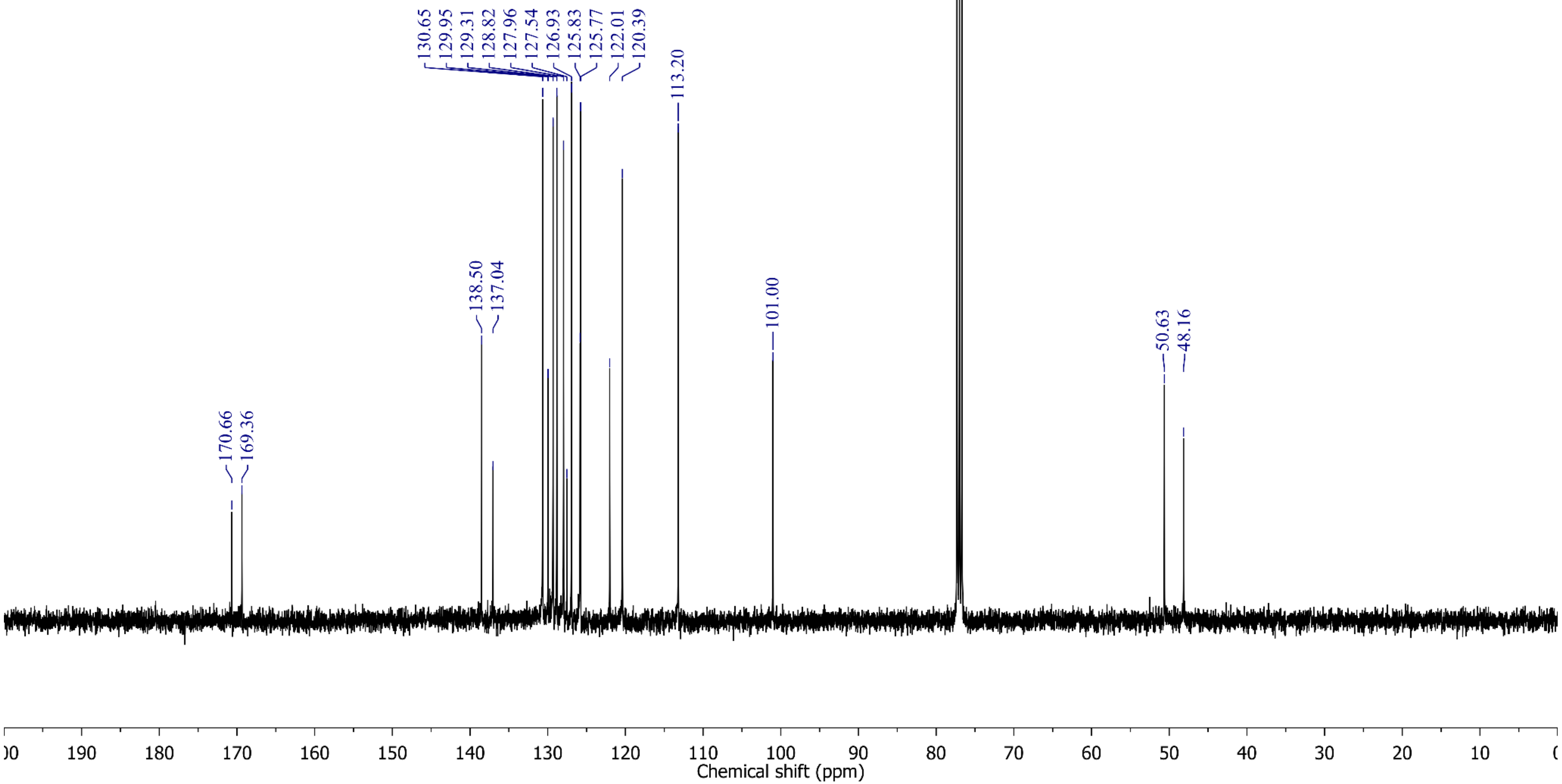

3-(9-Benzyl-9,10-dihydroacridin-9-yl)-5-phenylisoxazole (33a), DEPT, $CDCl_3$, 100 MHz

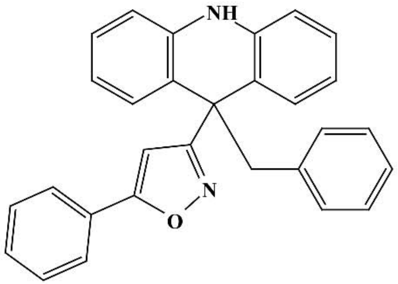

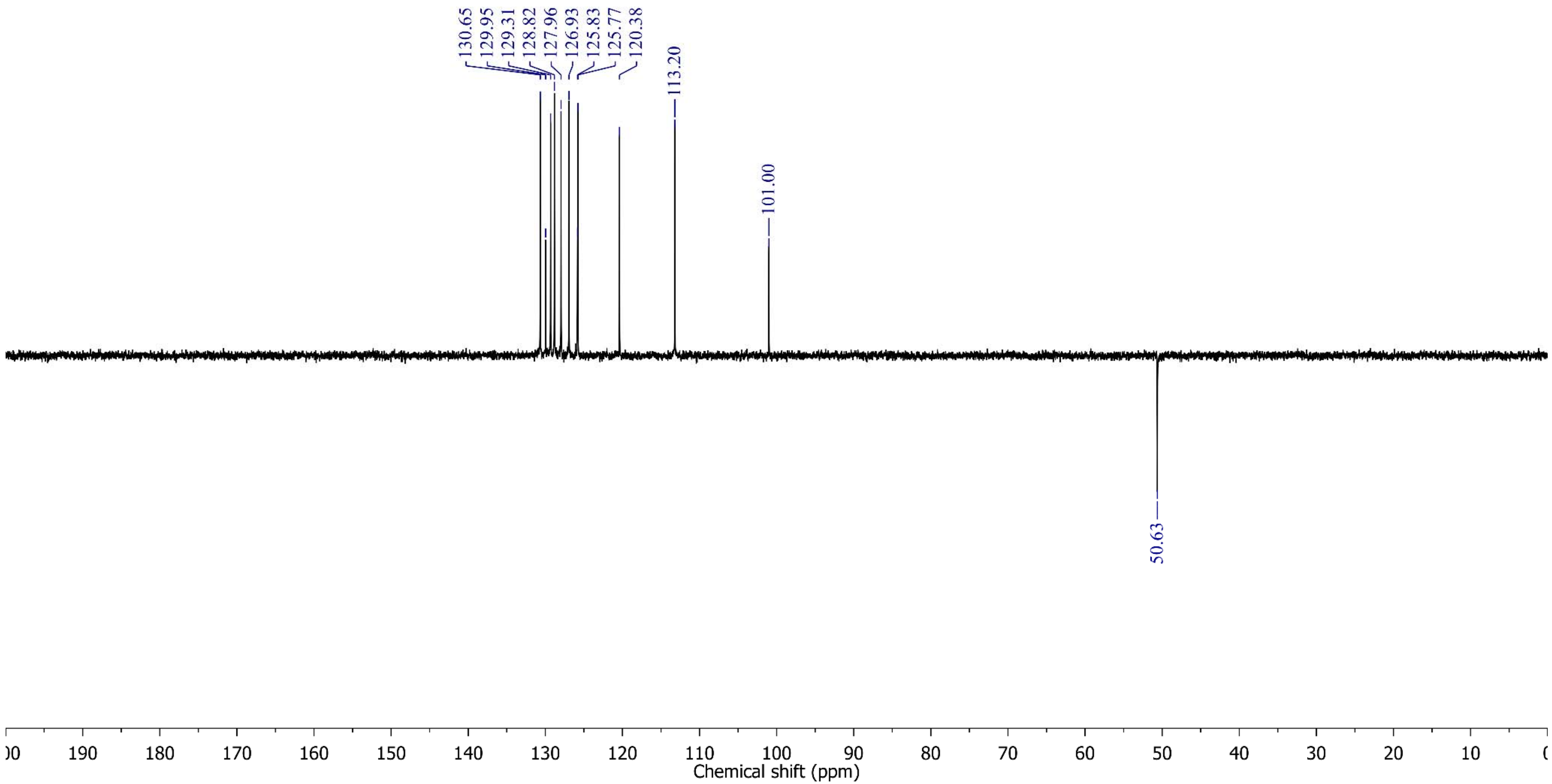

3-(9-(3,5-Dimethylbenzyl)-9,10-dihydroacridin-9-yl)-5-phenylisoxazole (33b), ^{1}H NMR, $CDCl_3$, 400 MHz

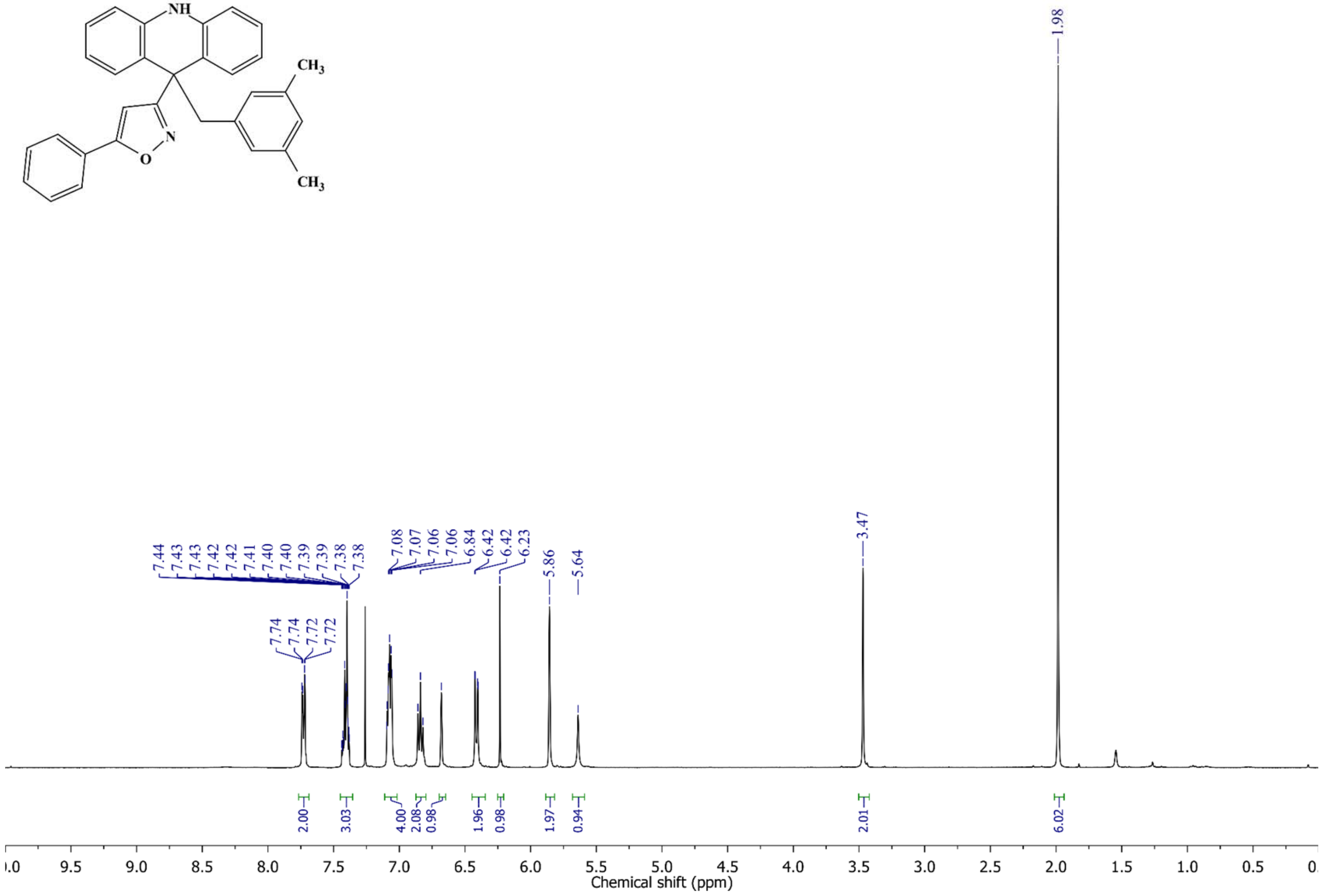

3-(9-(3,5-Dimethylbenzyl)-9,10-dihydroacridin-9-yl)-5-phenylisoxazole (33b), $^{13}C\{^1H\}$ NMR, $CDCl_3$, 100 MHz

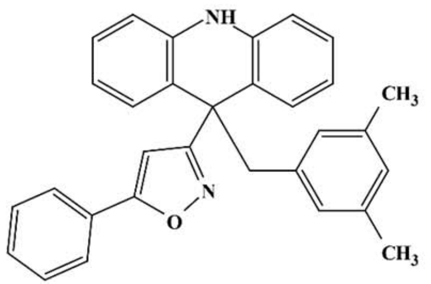

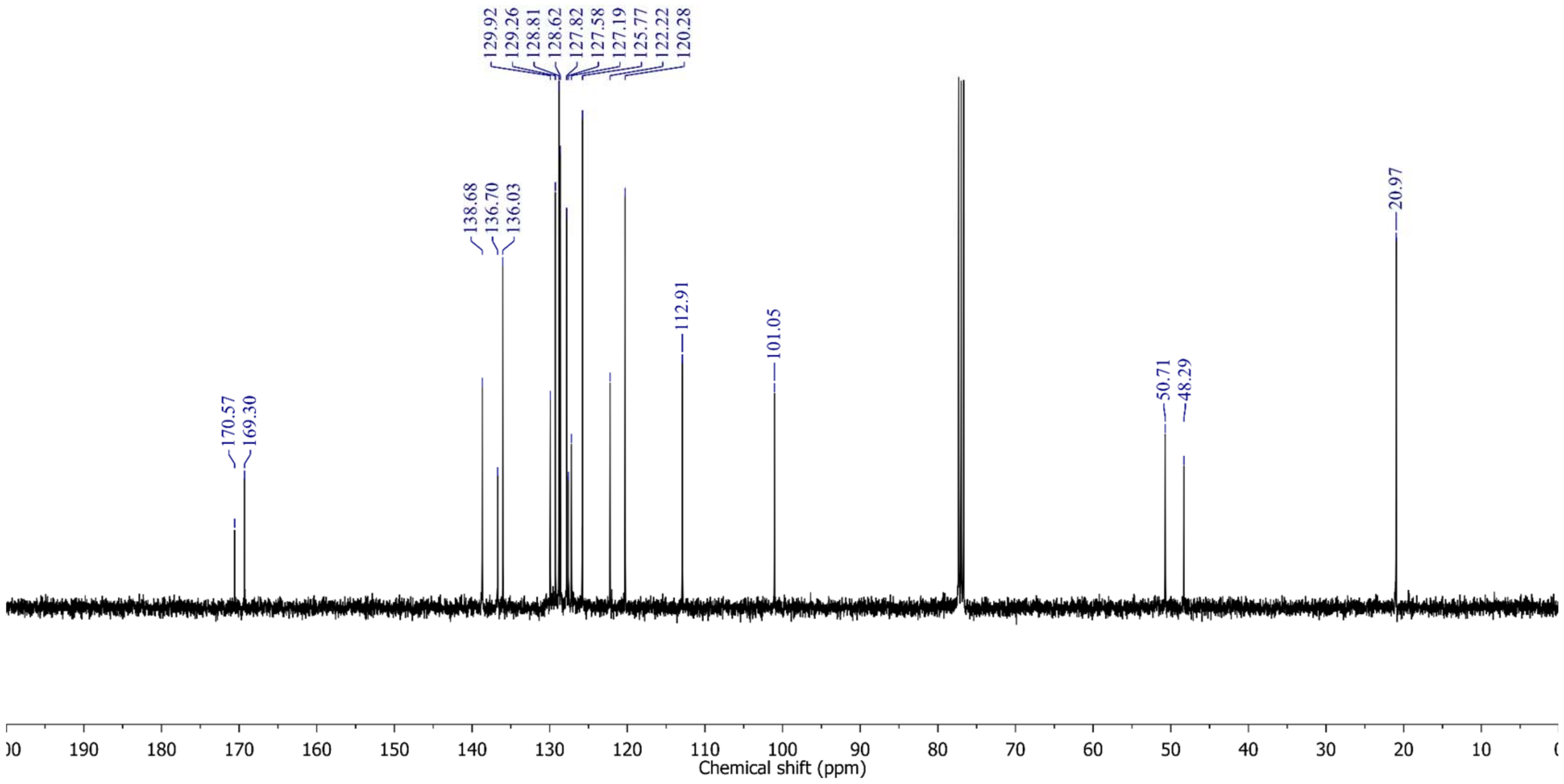

3-(9-(3,5-Dimethylbenzyl)-9,10-dihydroacridin-9-yl)-5-phenylisoxazole (33b), DEPT, $CDCl_3$, 100 MHz

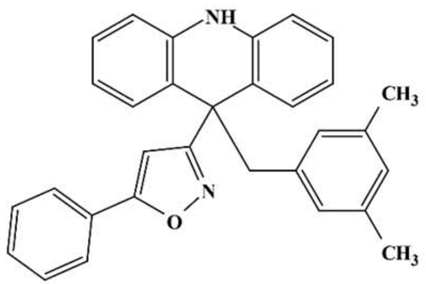

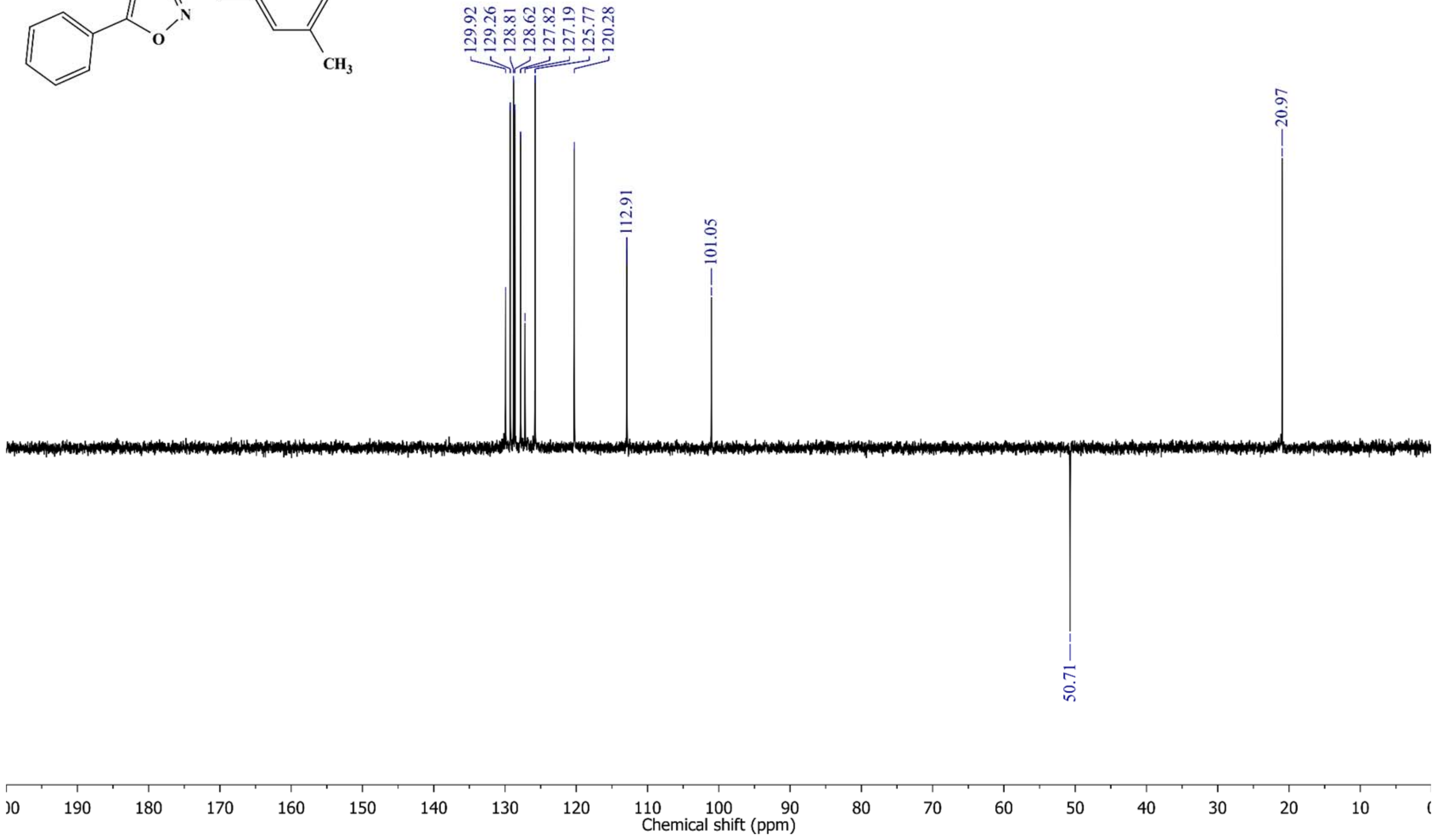

3-(9-(4-Chlorobenzyl)-9,10-dihydroacridin-9-yl)-5-phenylisoxazole (33c), ^{1}H NMR, C_6D_6, 400 MHz

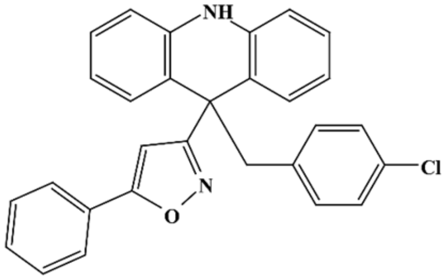

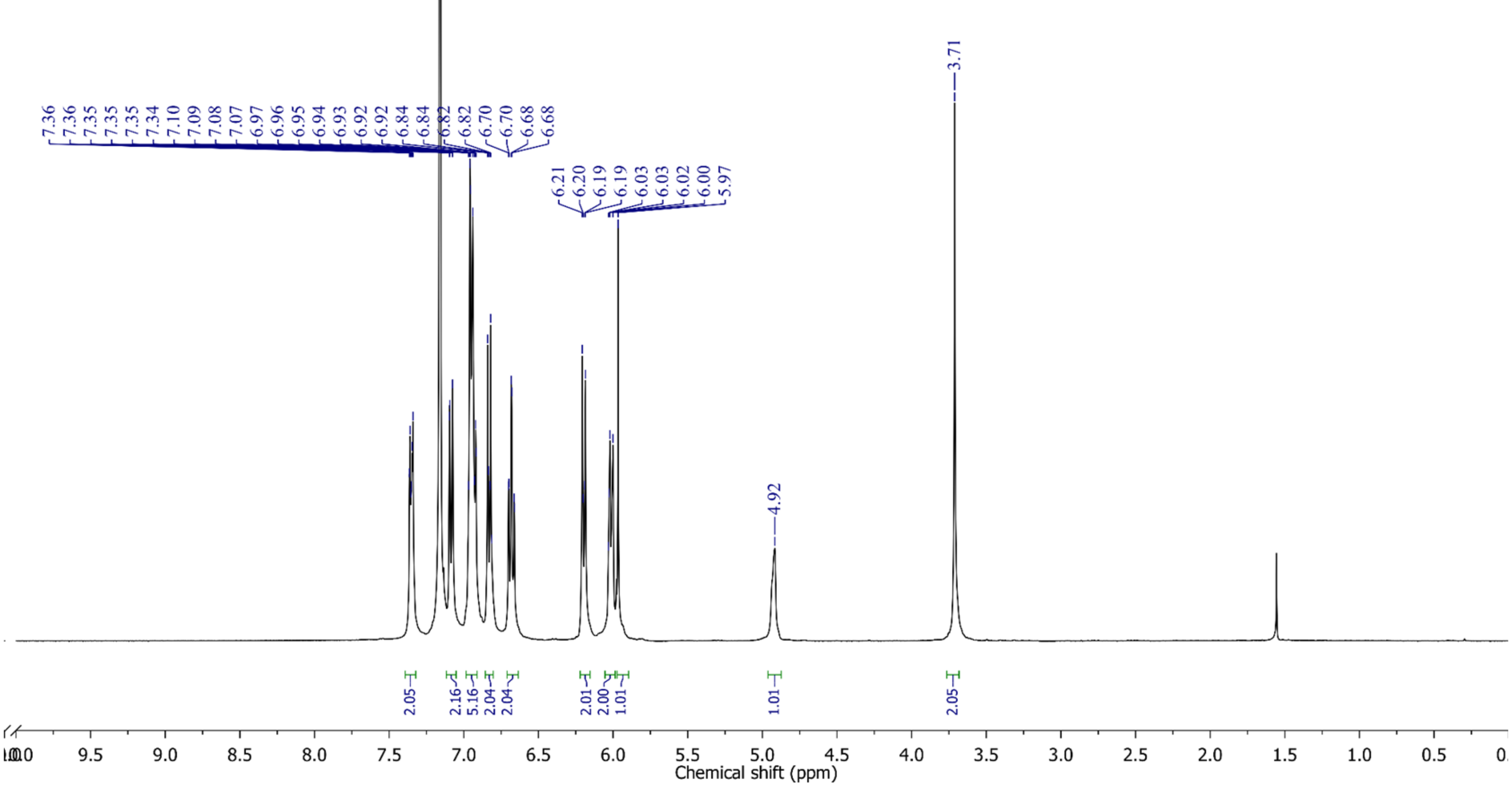

3-(9-(4-Chlorobenzyl)-9,10-dihydroacridin-9-yl)-5-phenylisoxazole (33c), $^{13}C\{^{1}H\}$ NMR, C_6D_6, 100 MHz

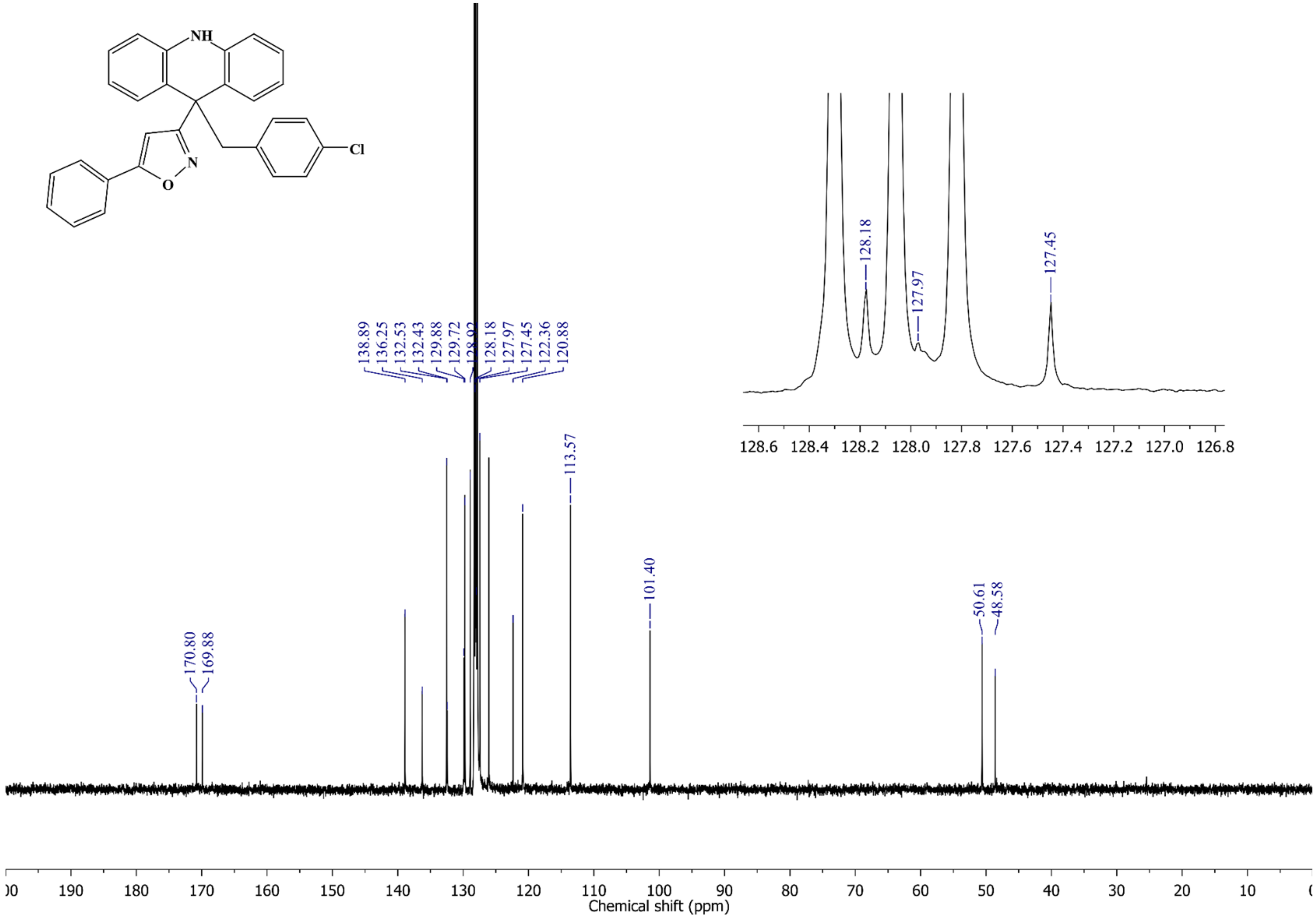

3-(9-(4-Chlorobenzyl)-9,10-dihydroacridin-9-yl)-5-phenylisoxazole (33c), DEPT, C_6D_6, 100 MHz

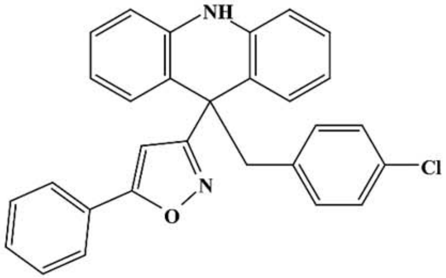

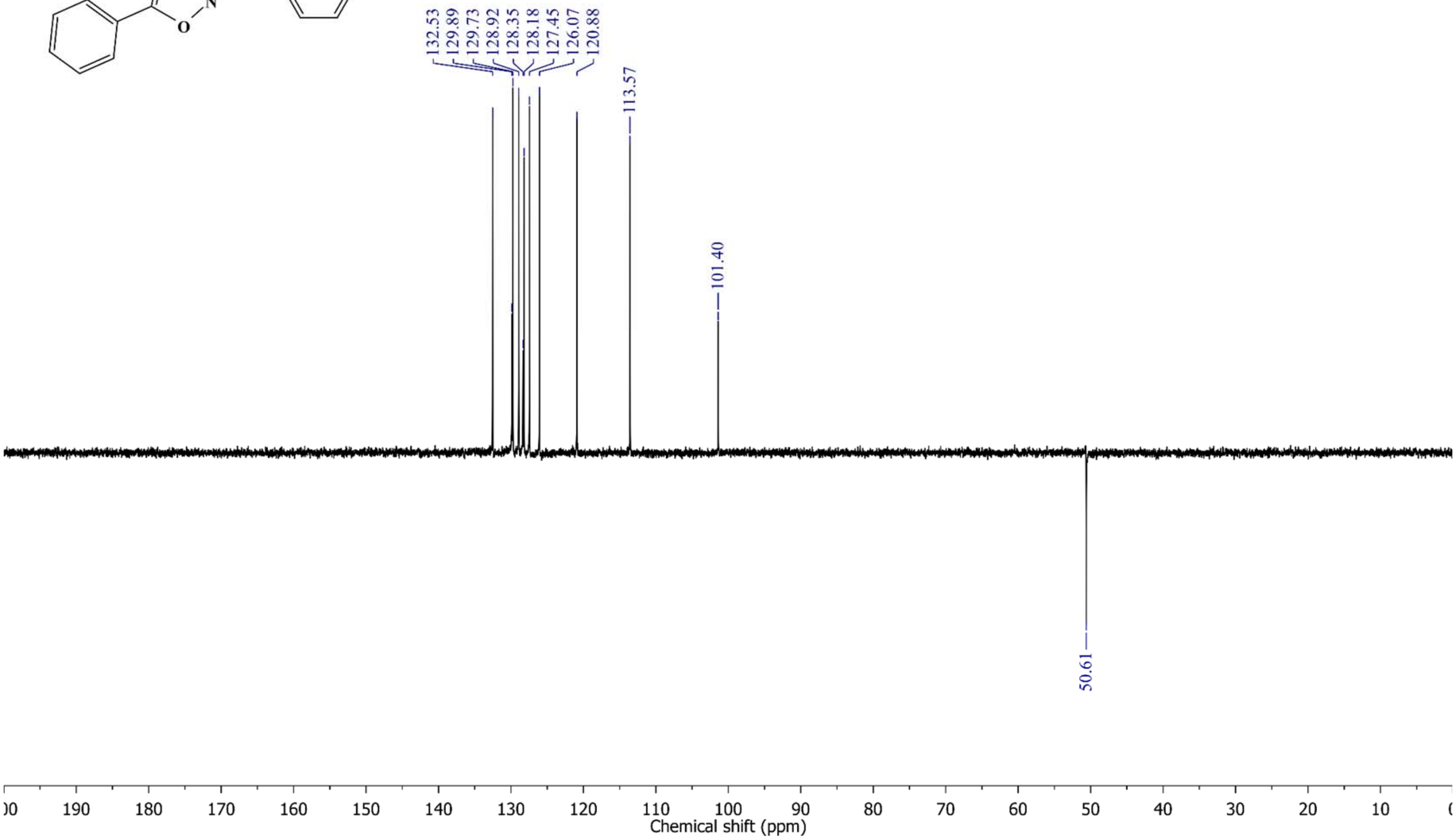

3-(9-(*tert*-Butoxymethyl)-9,10-dihydroacridin-9-yl)-5-phenylisoxazole (33d), ^{1}H NMR, C_6D_6, 400 MHz

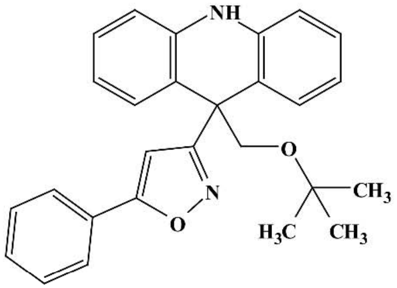

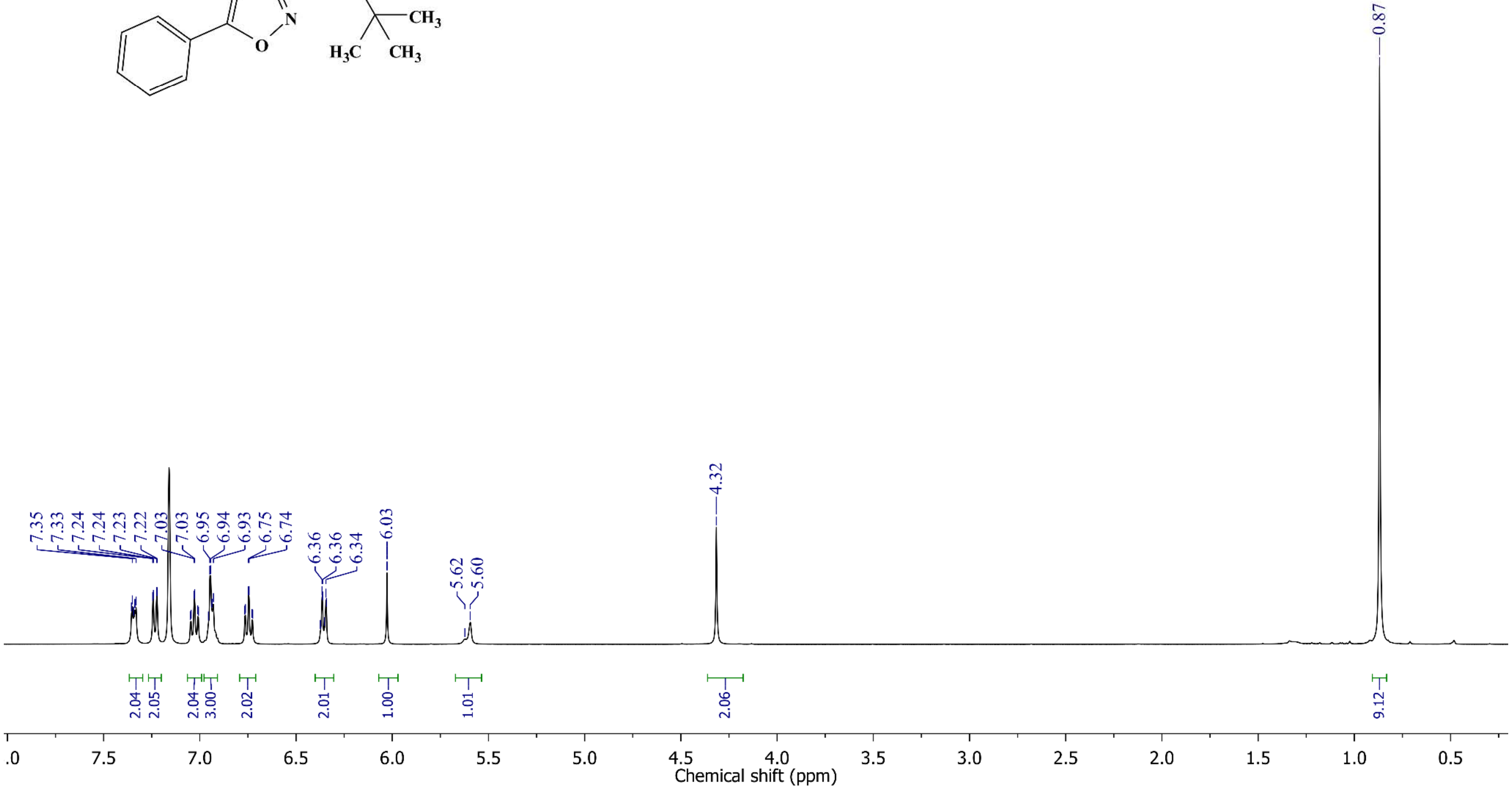

3-(9-(*tert*-Butoxymethyl)-9,10-dihydroacridin-9-yl)-5-phenylisoxazole (33d), $^{13}C\{^1H\}$ NMR, C_6D_6, 100 MHz

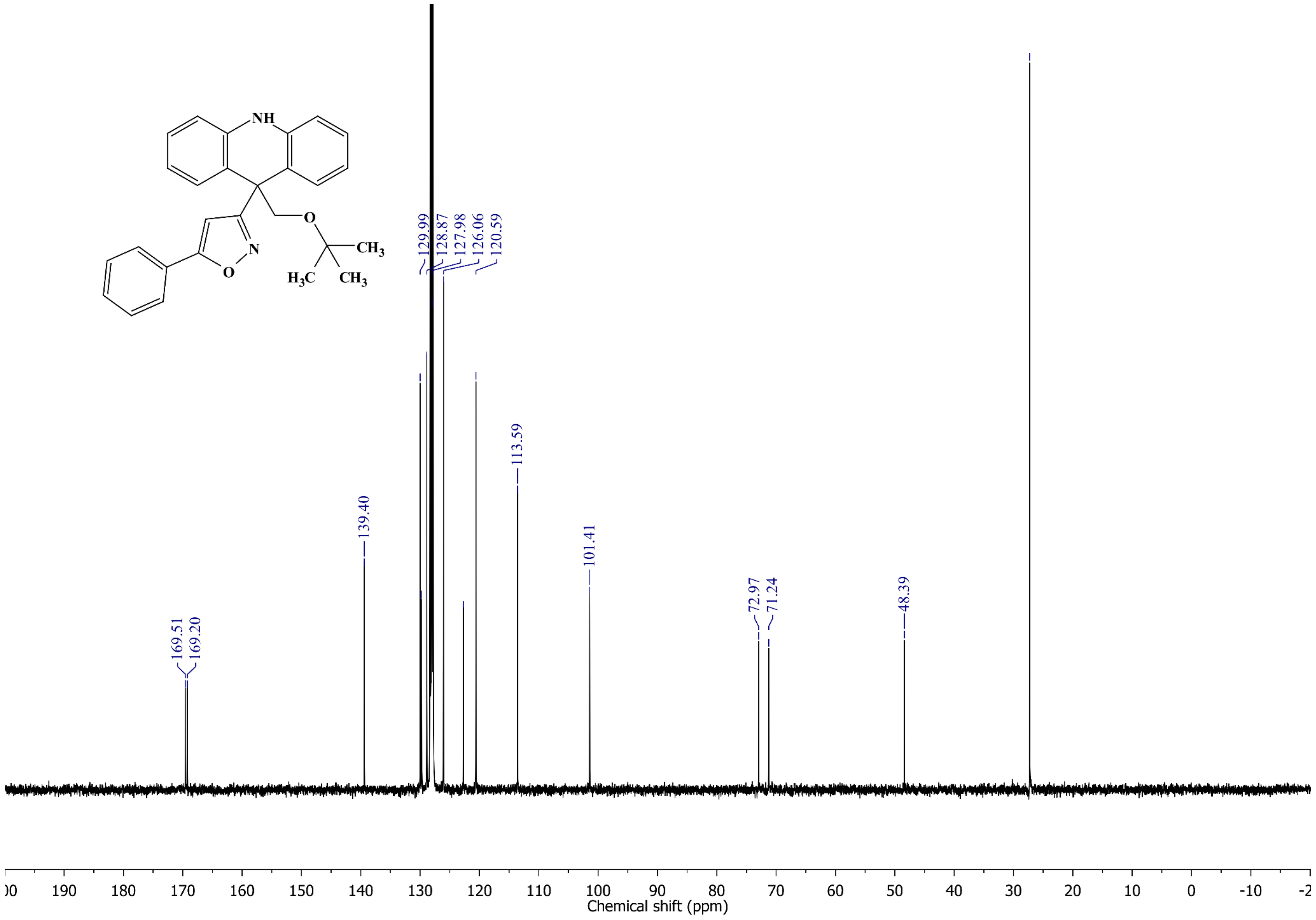

3-(9-(*tert*-Butoxymethyl)-9,10-dihydroacridin-9-yl)-5-phenylisoxazole (33d), DEPT, C_6D_6, 100 MHz

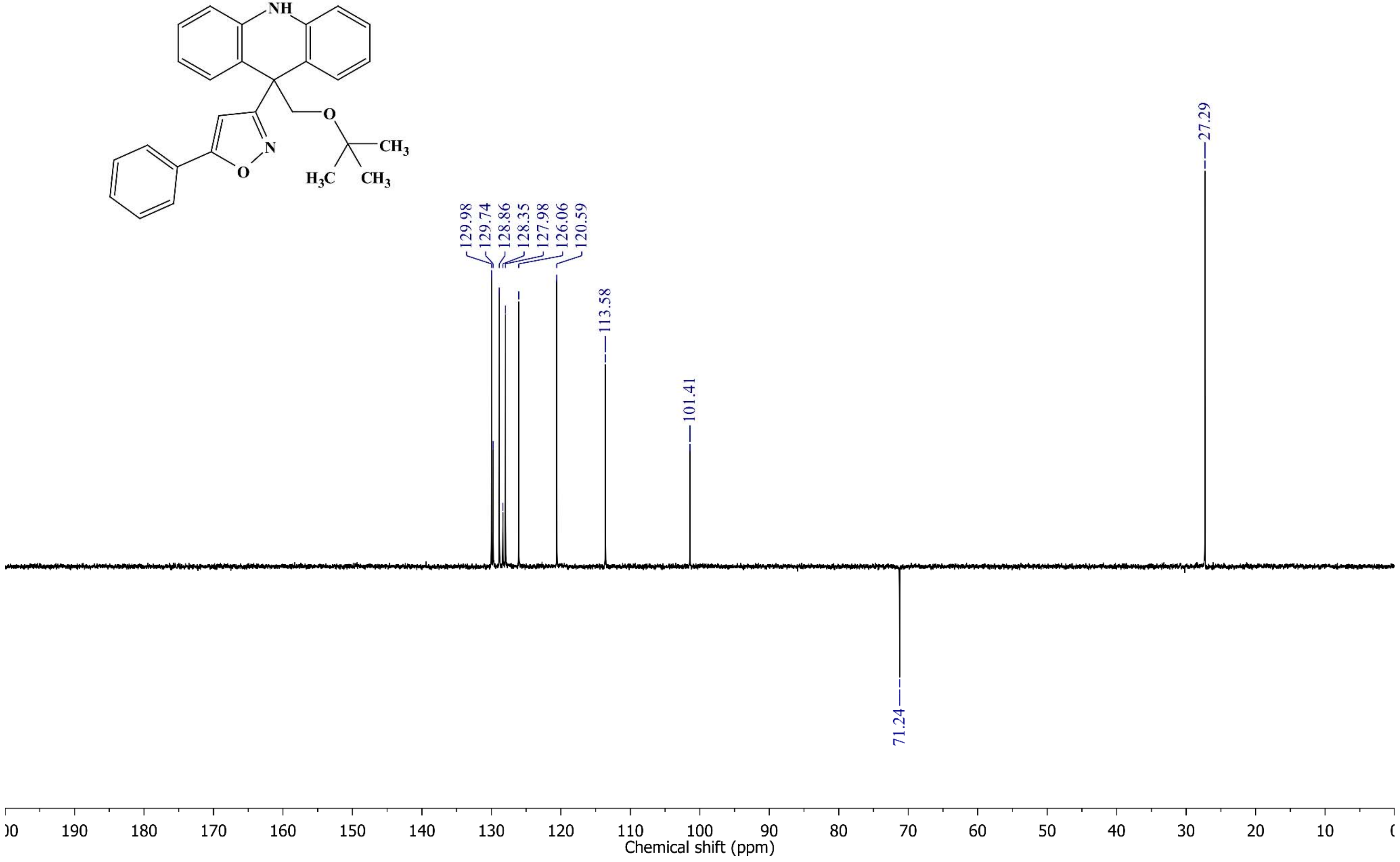

3-(9-(1,4-Dioxan-2-yl)-9,10-dihydroacridin-9-yl)-5-phenylisoxazole (33e), ^{1}H NMR, $CDCl_3$, 400 MHz

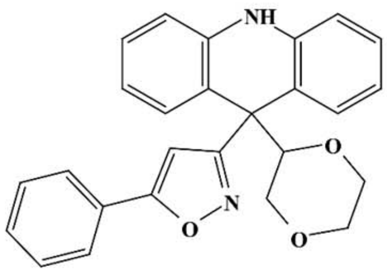

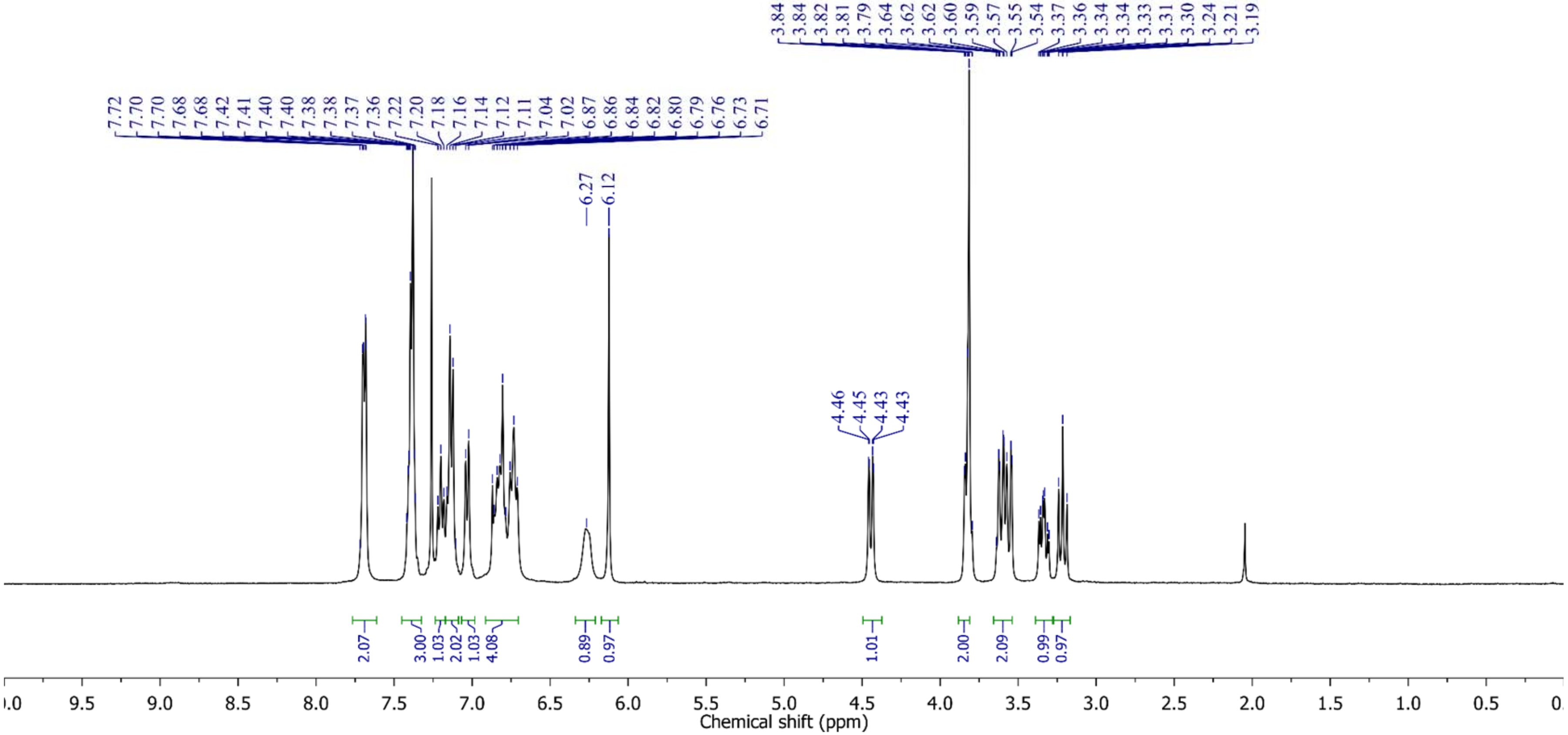

3-(9-(1,4-Dioxan-2-yl)-9,10-dihydroacridin-9-yl)-5-phenylisoxazole (33e), $^{13}C\{^{1}H\}$ NMR, $CDCl_3$, 100 MHz

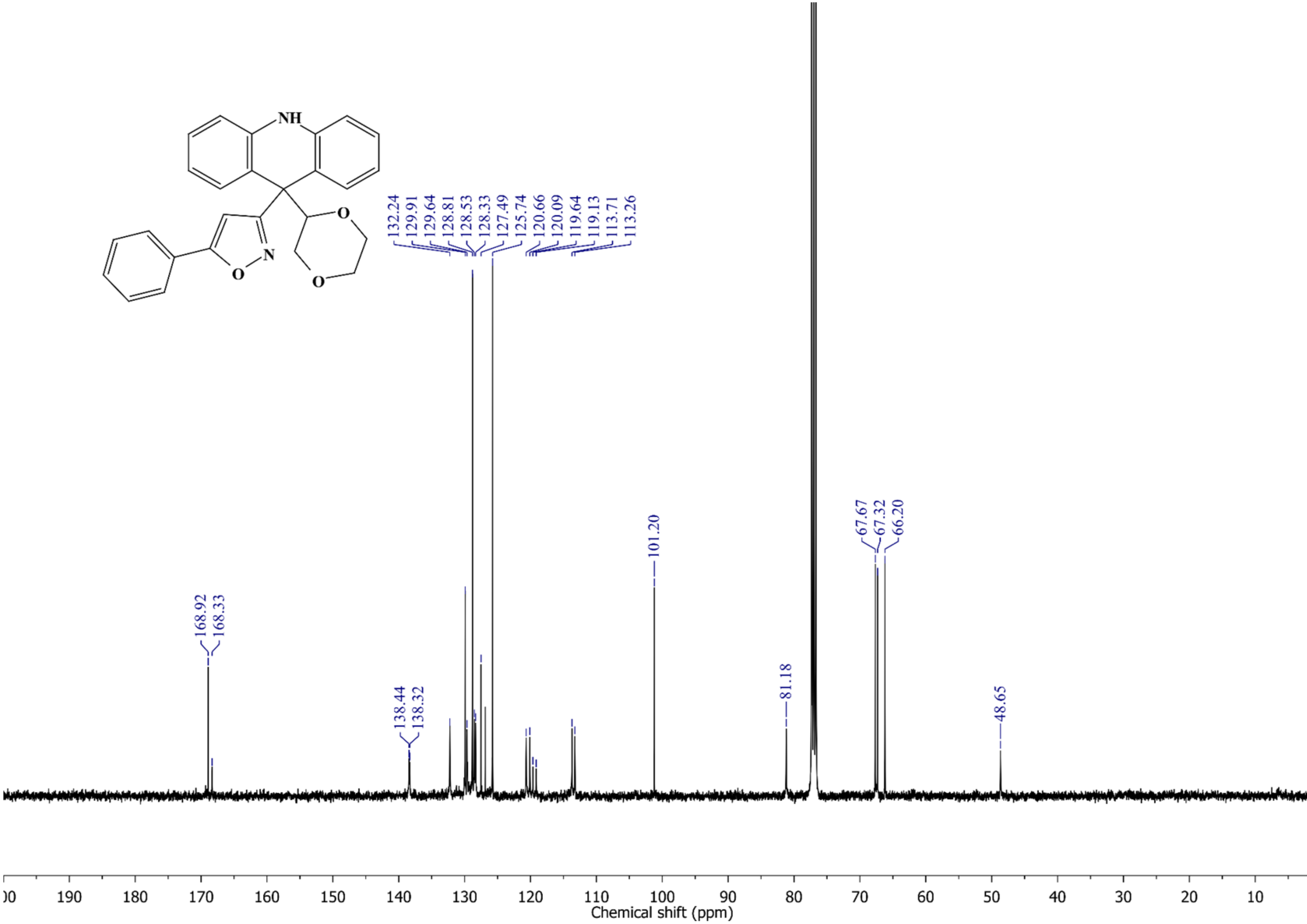

3-(9-(1,4-Dioxan-2-yl)-9,10-dihydroacridin-9-yl)-5-phenylisoxazole (33e), DEPT, $CDCl_3$, 100 MHz

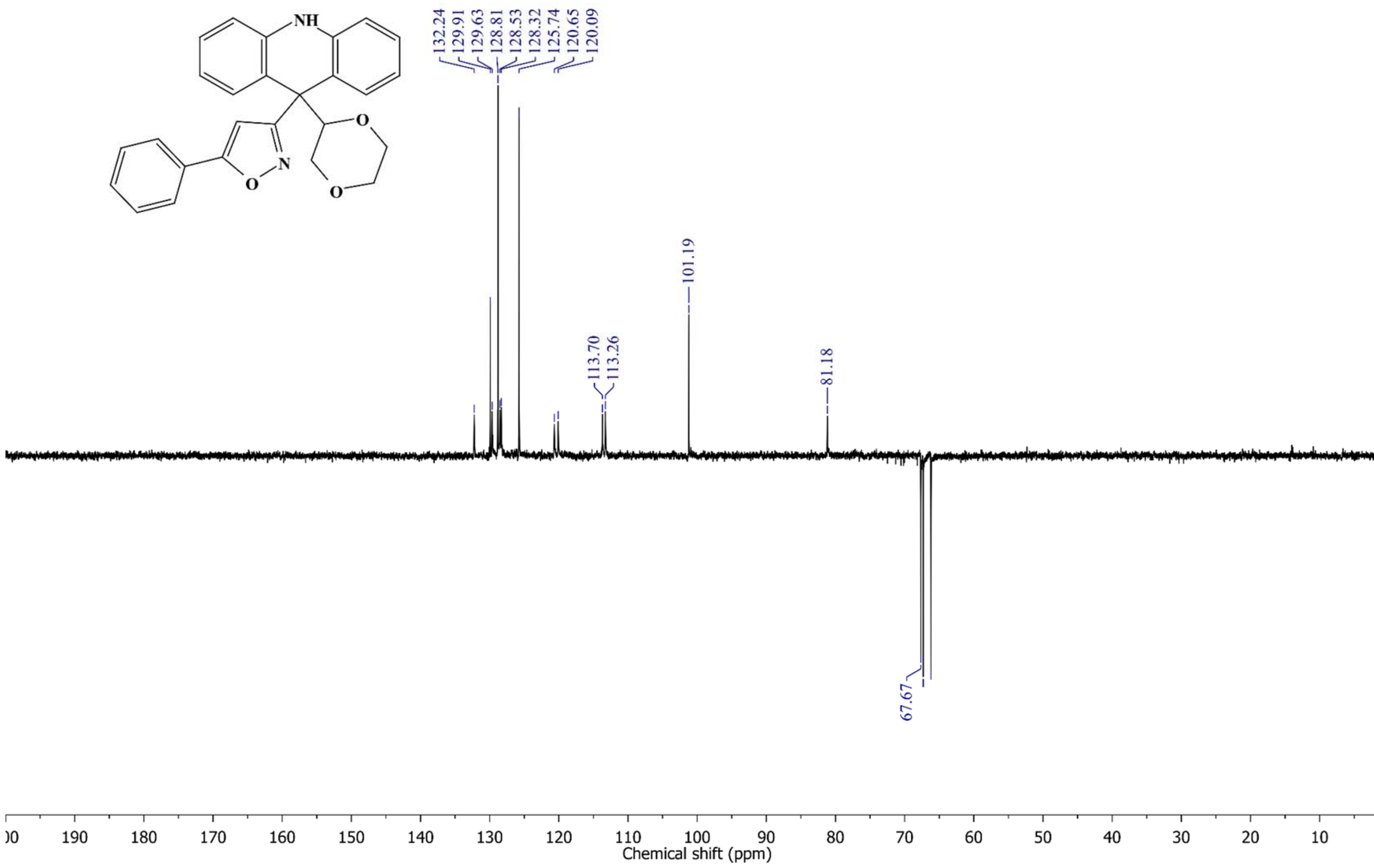

3-(9-(2-Methylbenzyl)-9,10-dihydroacridin-9-yl)-5-(trimethylsilyl)isoxazole (33f), ^{1}H NMR, $CDCl_3$, 400 MHz

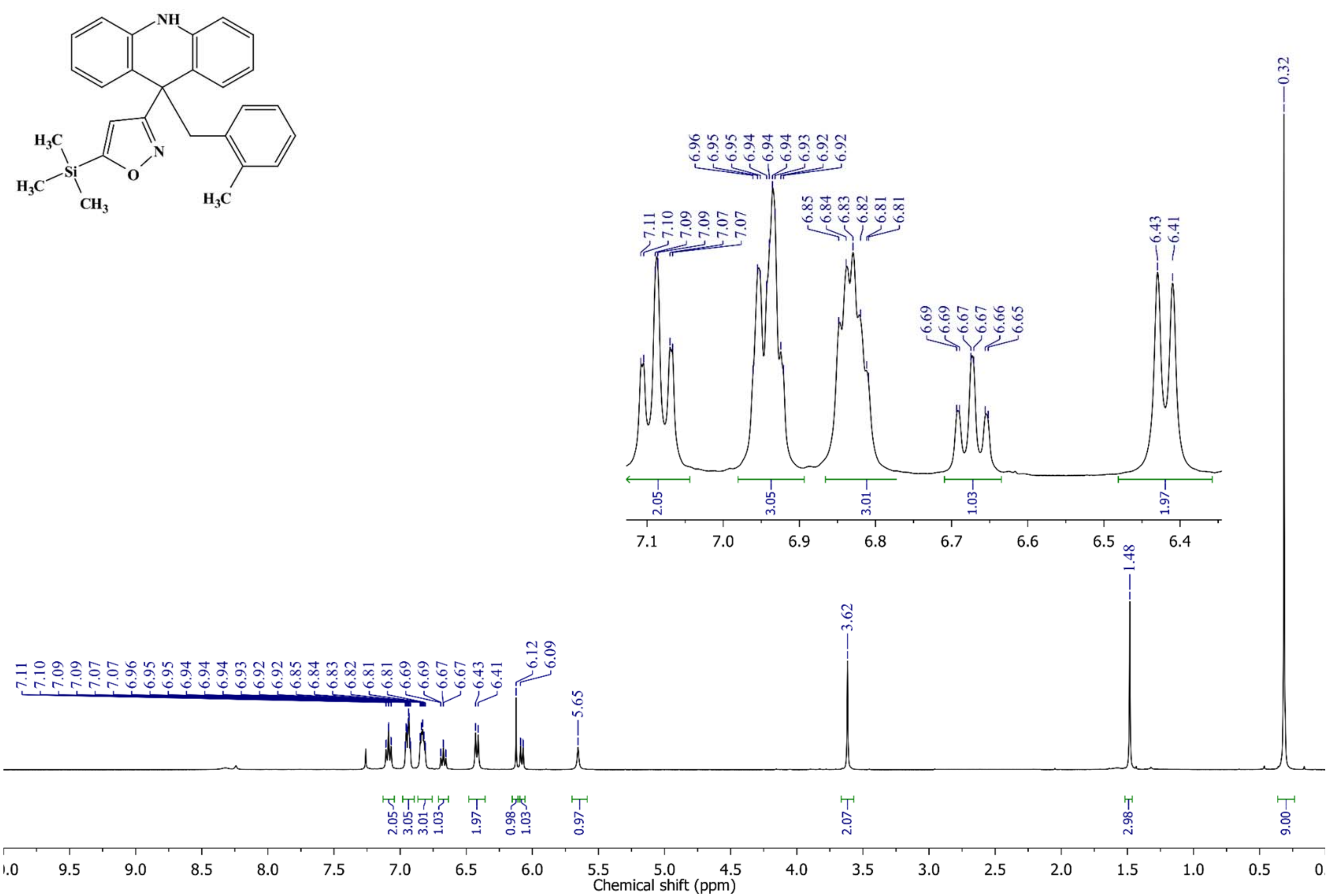

3-(9-(2-Methylbenzyl)-9,10-dihydroacridin-9-yl)-5-(trimethylsilyl)isoxazole (33f), $^{13}C\{^{1}H\}$ NMR, $CDCl_3$, 100 MHz

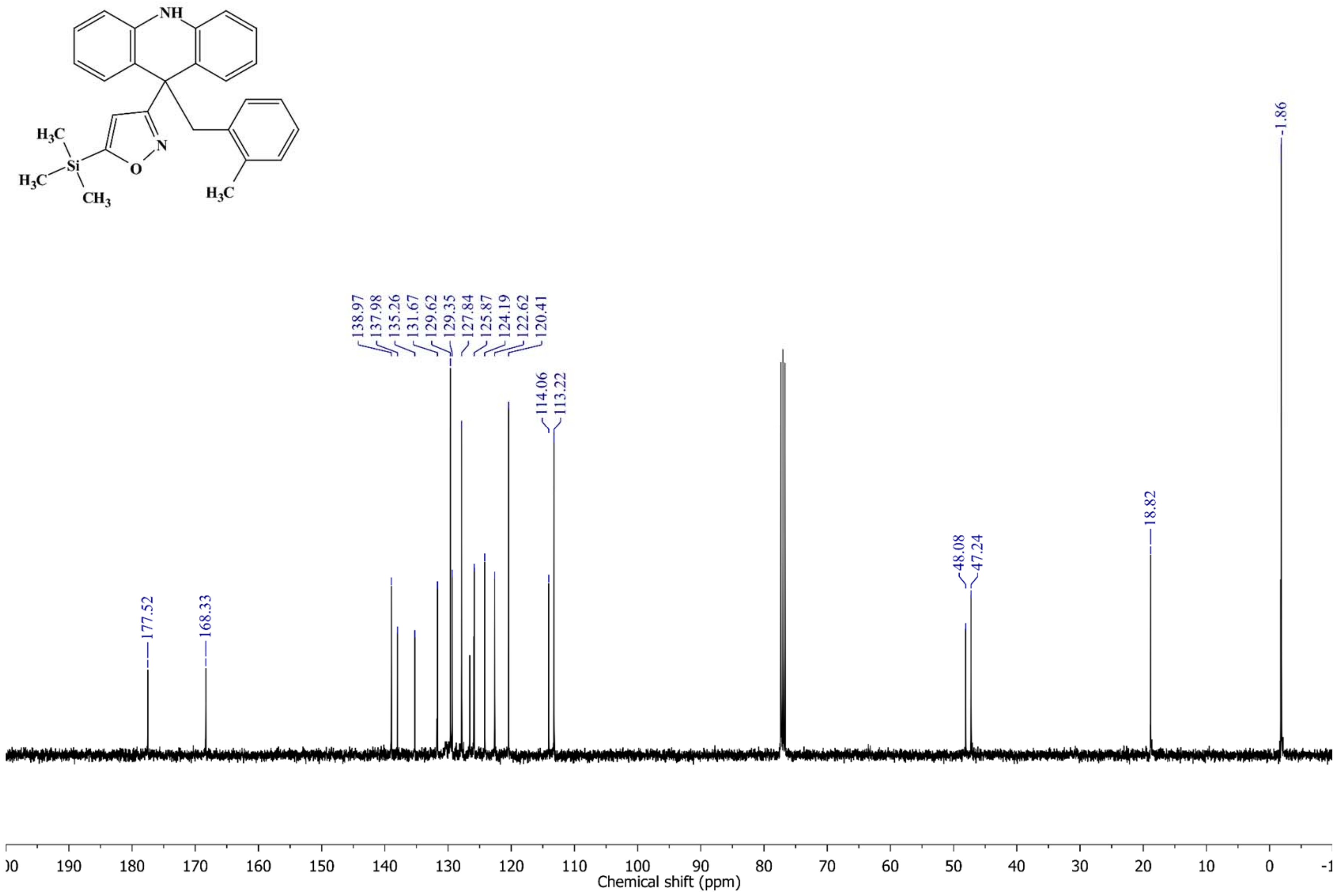

3-(9-(2-Methylbenzyl)-9,10-dihydroacridin-9-yl)-5-(trimethylsilyl)isoxazole (33f), DEPT, $CDCl_3$, 100 MHz

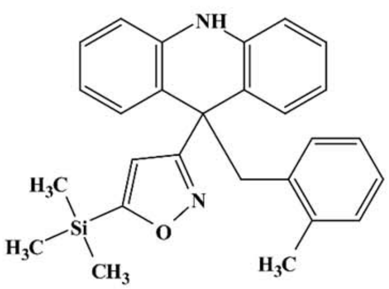

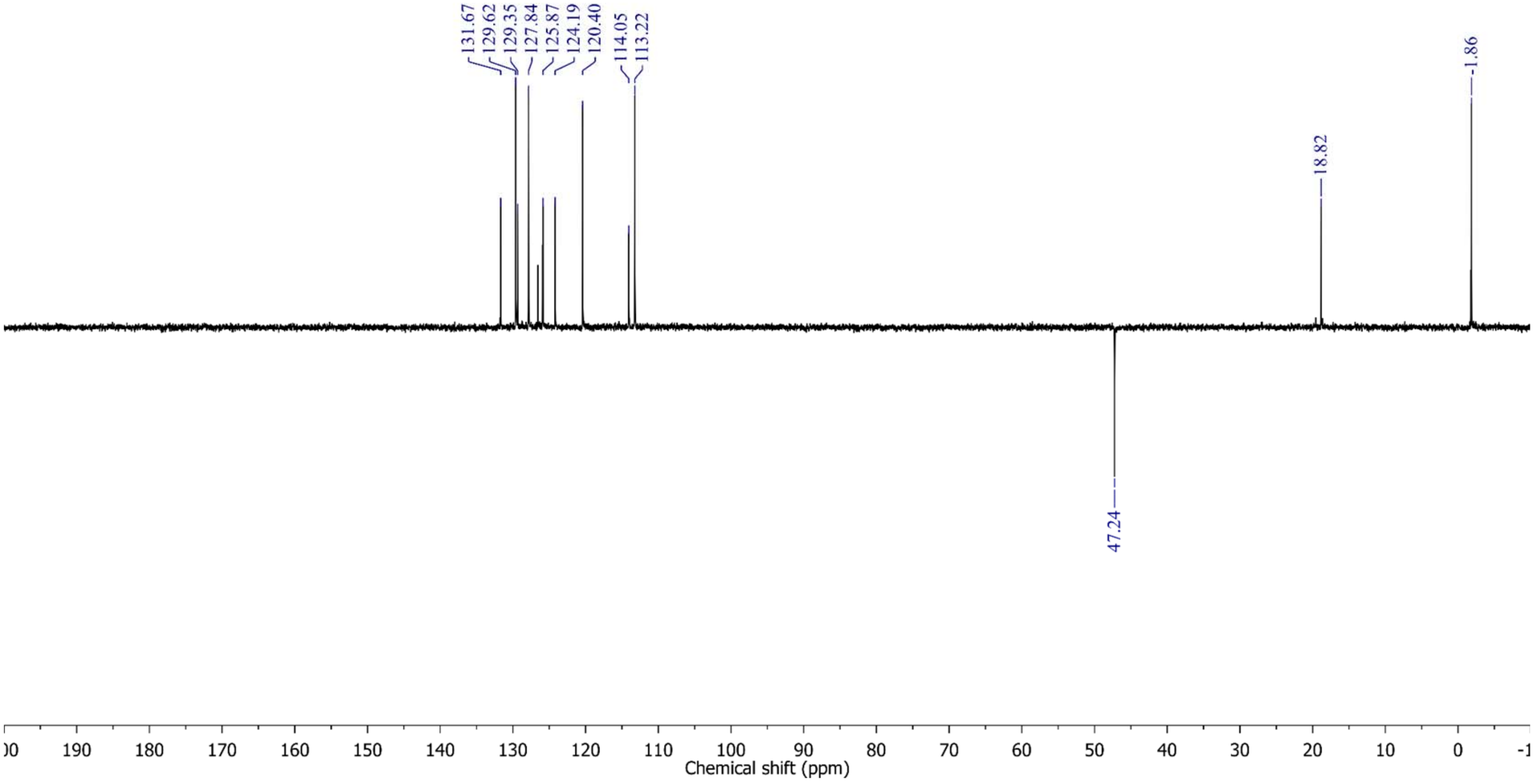

3-(9-(Tetrahydrofuran-2-yl)-9,10-dihydroacridin-9-yl)-5-(trimethylsilyl)isoxazole (33g), ^{1}H NMR, $CDCl_3$, 400 MHz

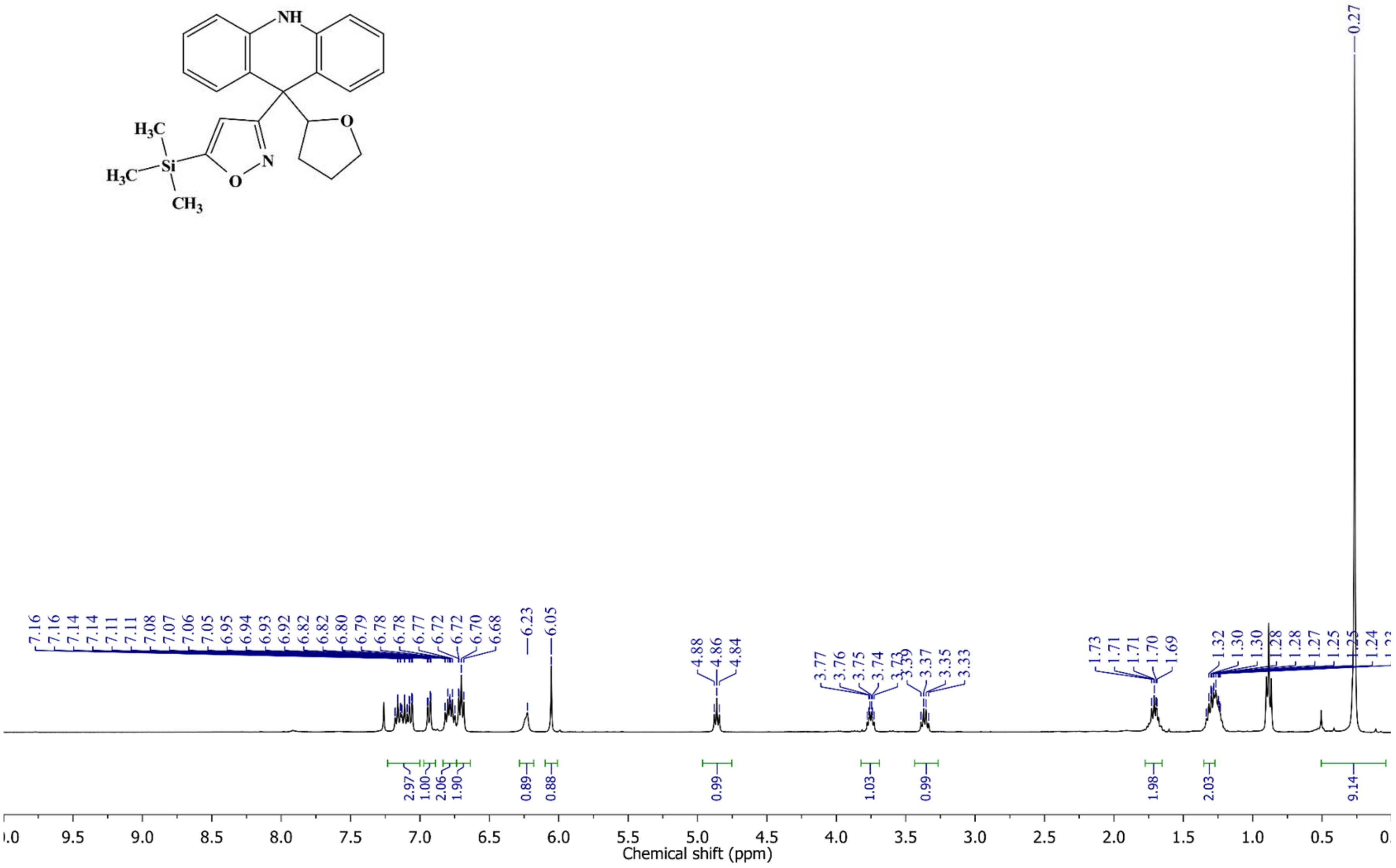

3-(9-(Tetrahydrofuran-2-yl)-9,10-dihydroacridin-9-yl)-5-(trimethylsilyl)isoxazole (33g), $^{13}C\{^{1}H\}$ NMR, $CDCl_3$, 100 MHz

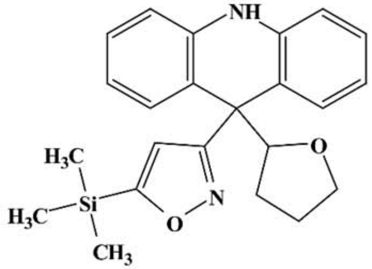

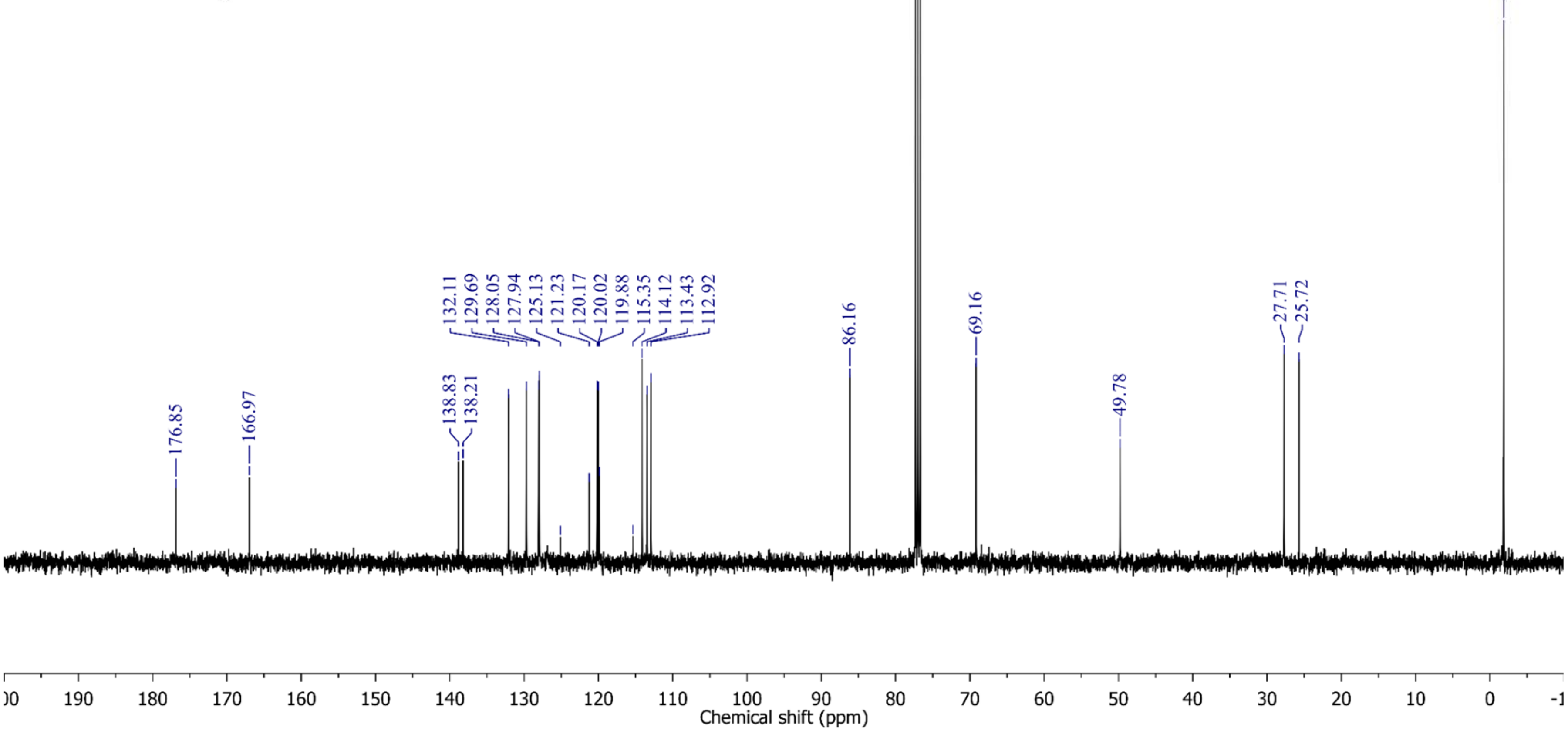

3-(9-(Tetrahydrofuran-2-yl)-9,10-dihydroacridin-9-yl)-5-(trimethylsilyl)isoxazole (33g), DEPT, $CDCl_3$, 100 MHz

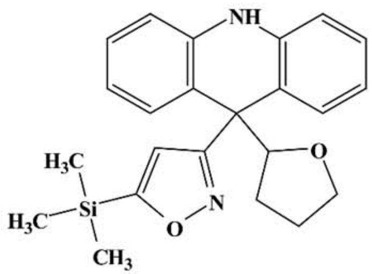

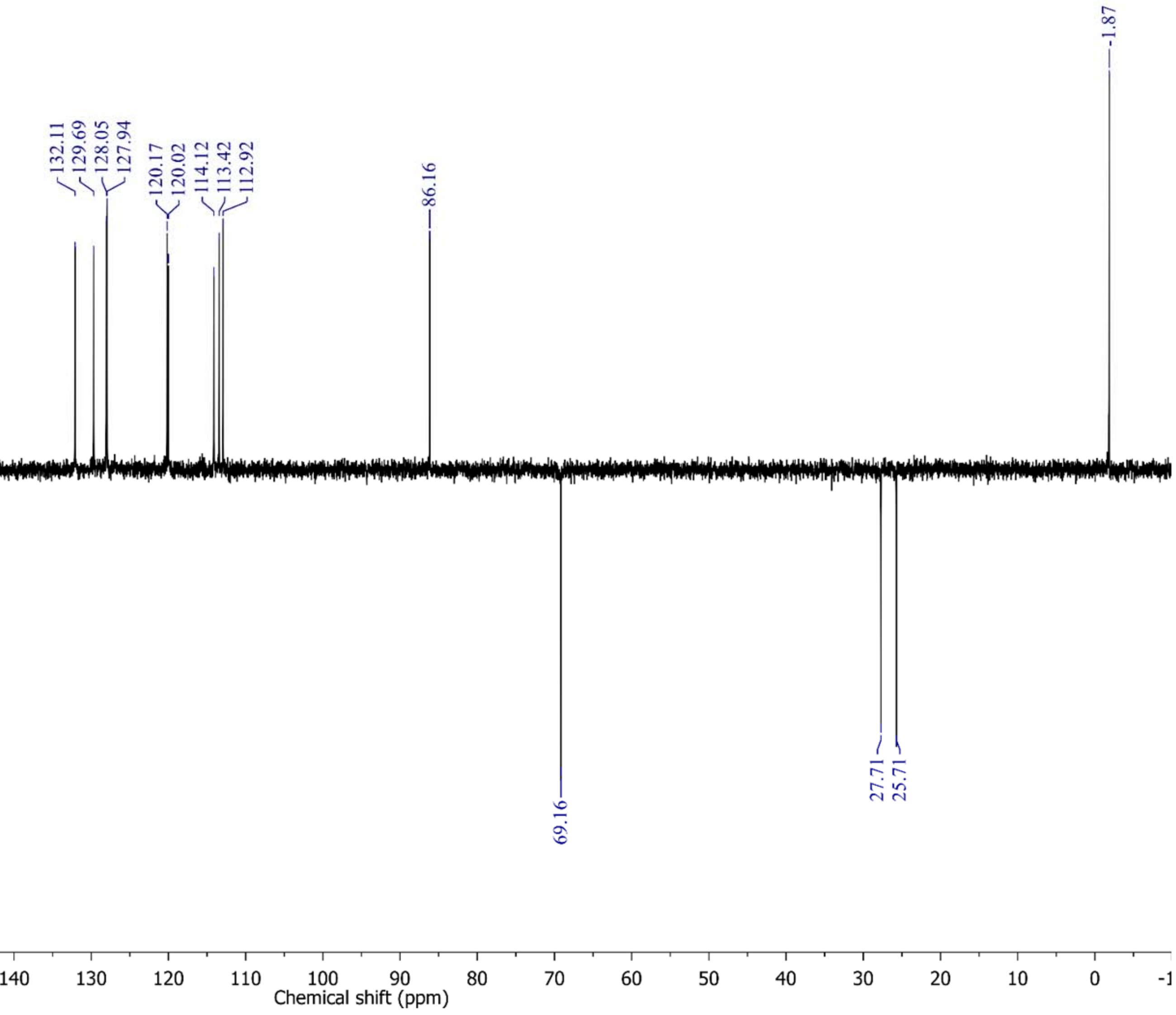

3-(9-Benzyl-9,10-dihydroacridin-9-yl)-5-(*tert*-butoxy)isoxazole (33h), ^{1}H NMR, $CDCl_3$, 400 MHz

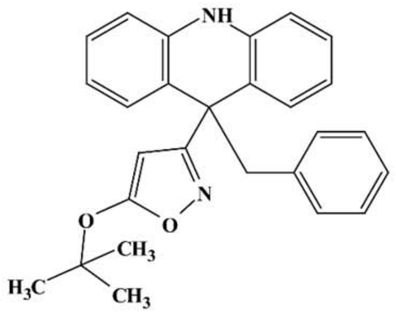

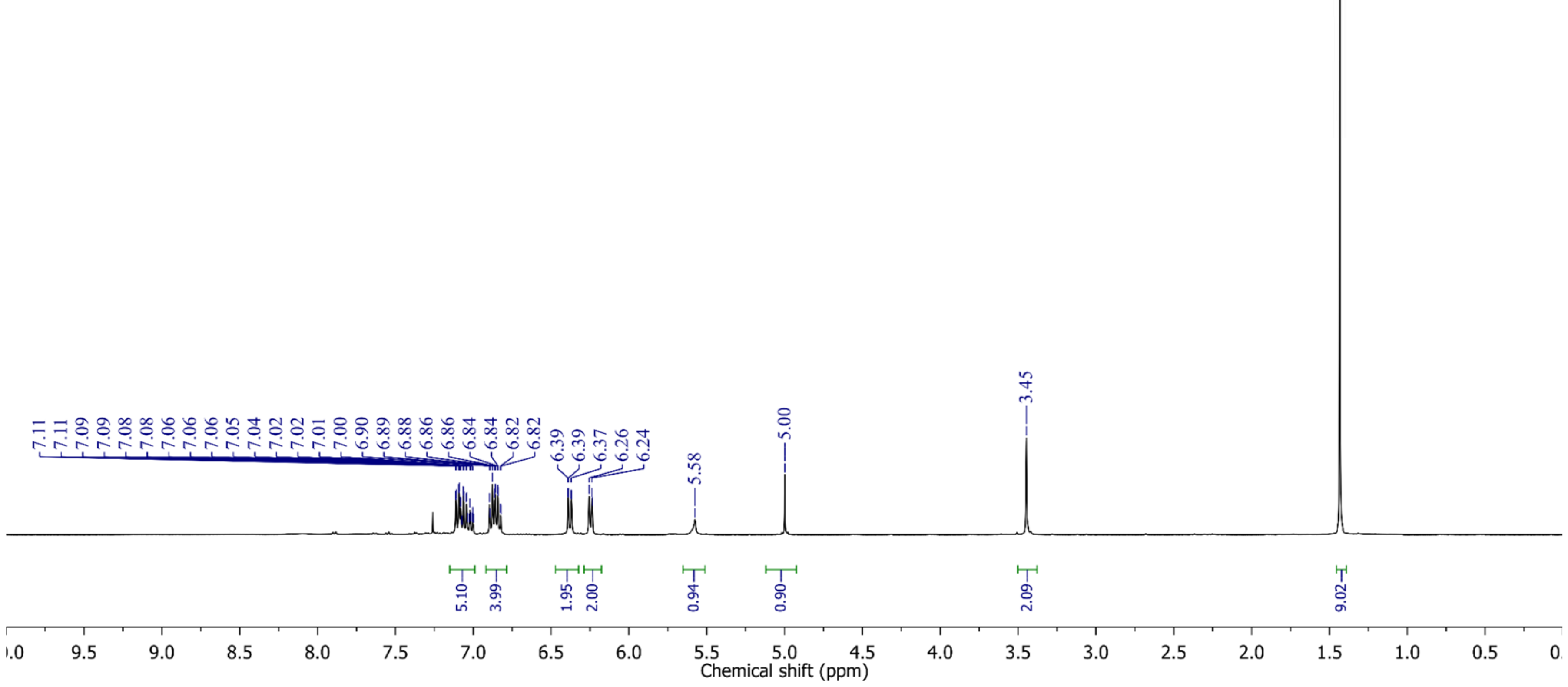

3-(9-Benzyl-9,10-dihydroacridin-9-yl)-5-(*tert*-butoxy)isoxazole (33h), $^{13}C\{^{1}H\}$ NMR, C_6D_6, 100 MHz

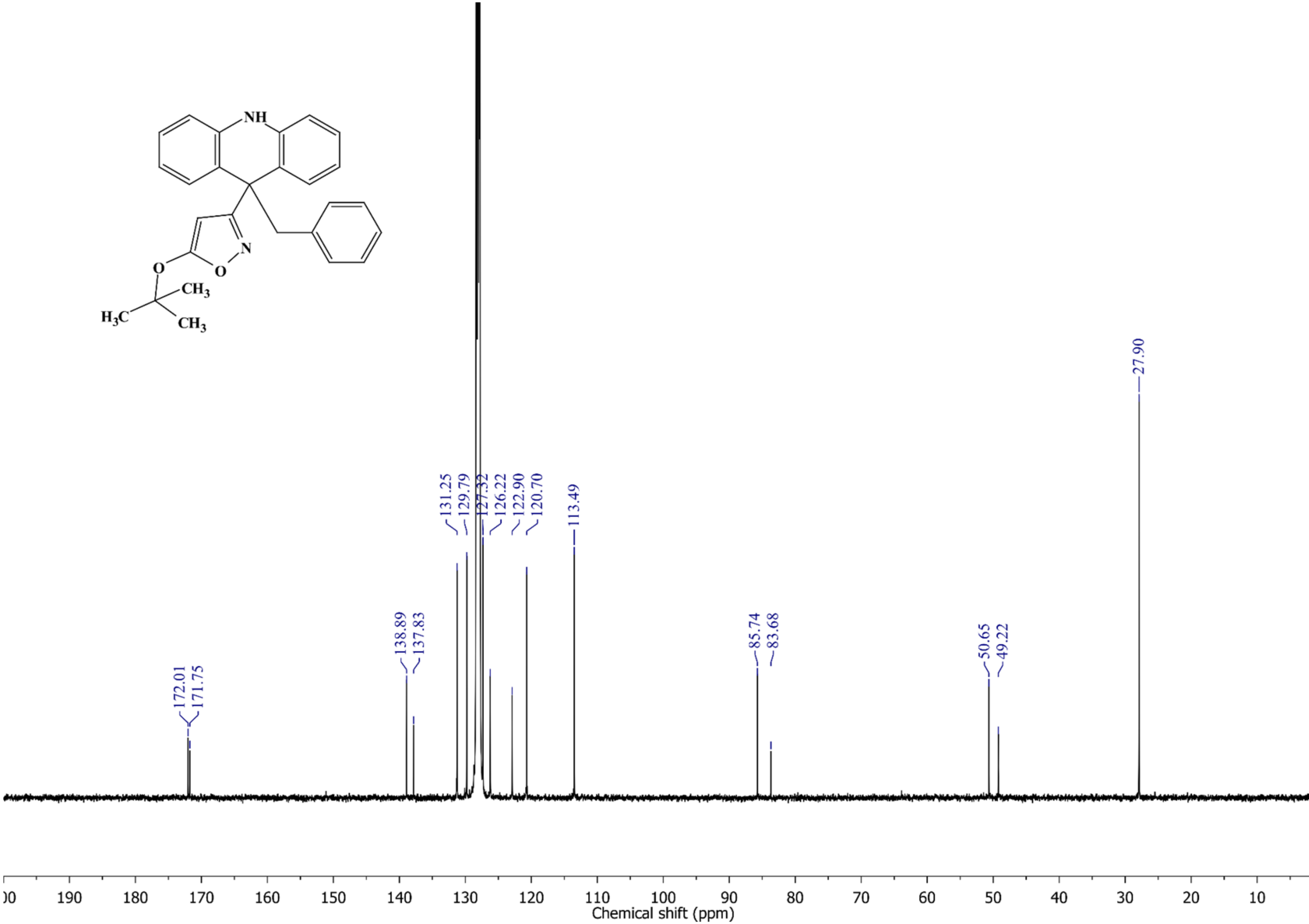

3-(9-Benzyl-9,10-dihydroacridin-9-yl)-5-(*tert*-butoxy)isoxazole (33h), DEPT, C_6D_6, 100 MHz

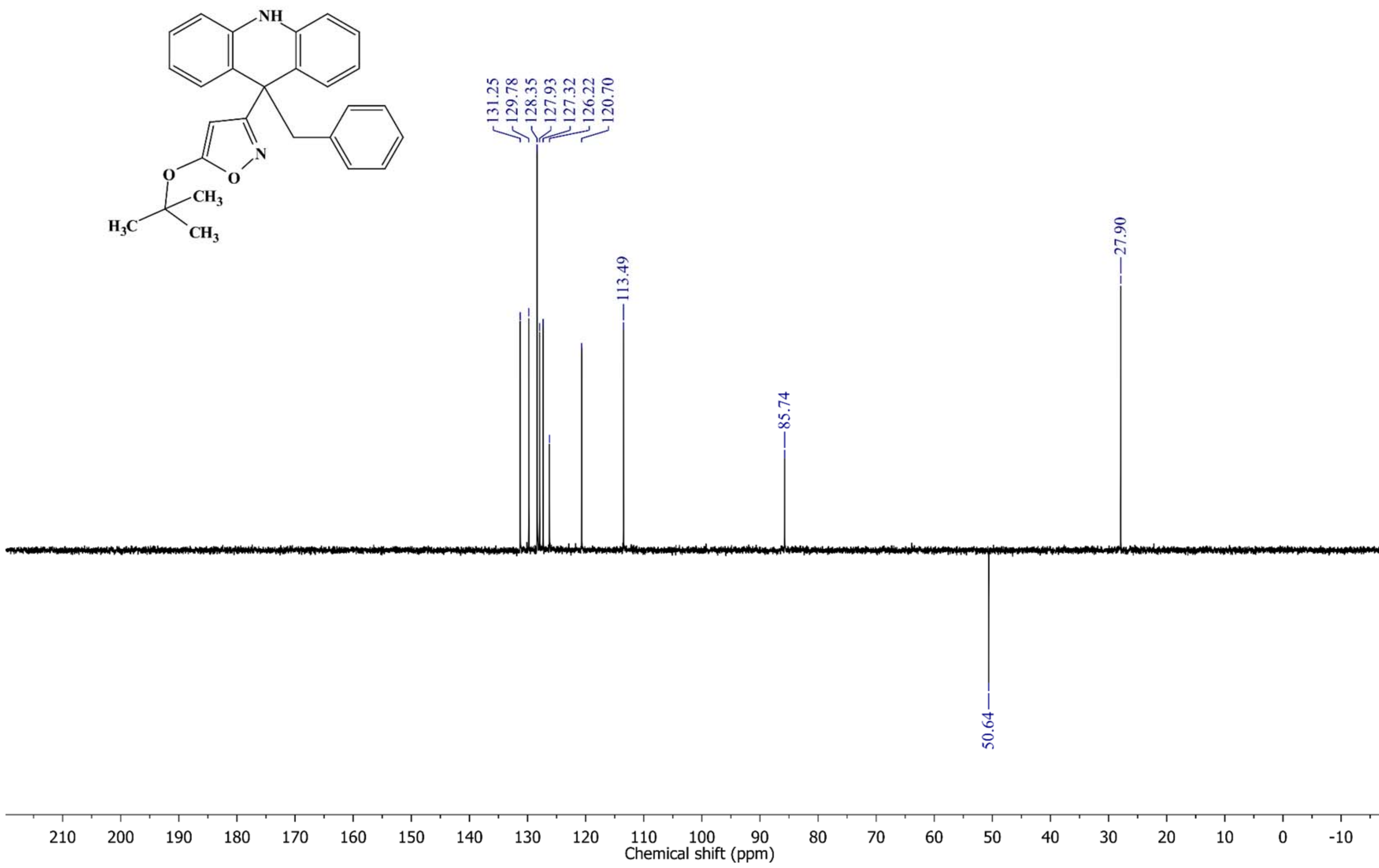

3-(9-(3,5-Dimethylbenzyl)-9,10-dihydroacridin-9-yl)-5-((4-isopropylphenoxy)methyl)isoxazole (33i), ^{1}H NMR, $CDCl_3$, 400 MHz

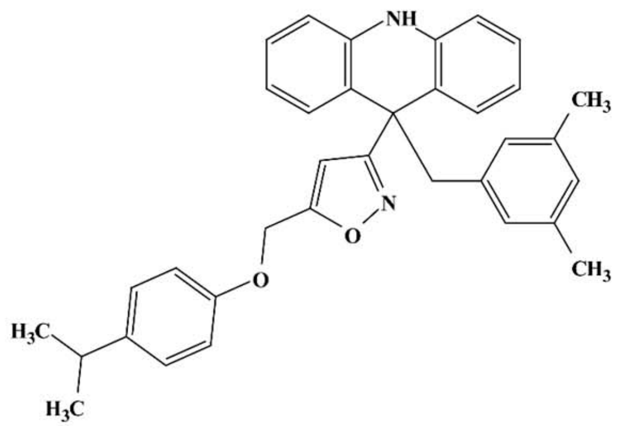

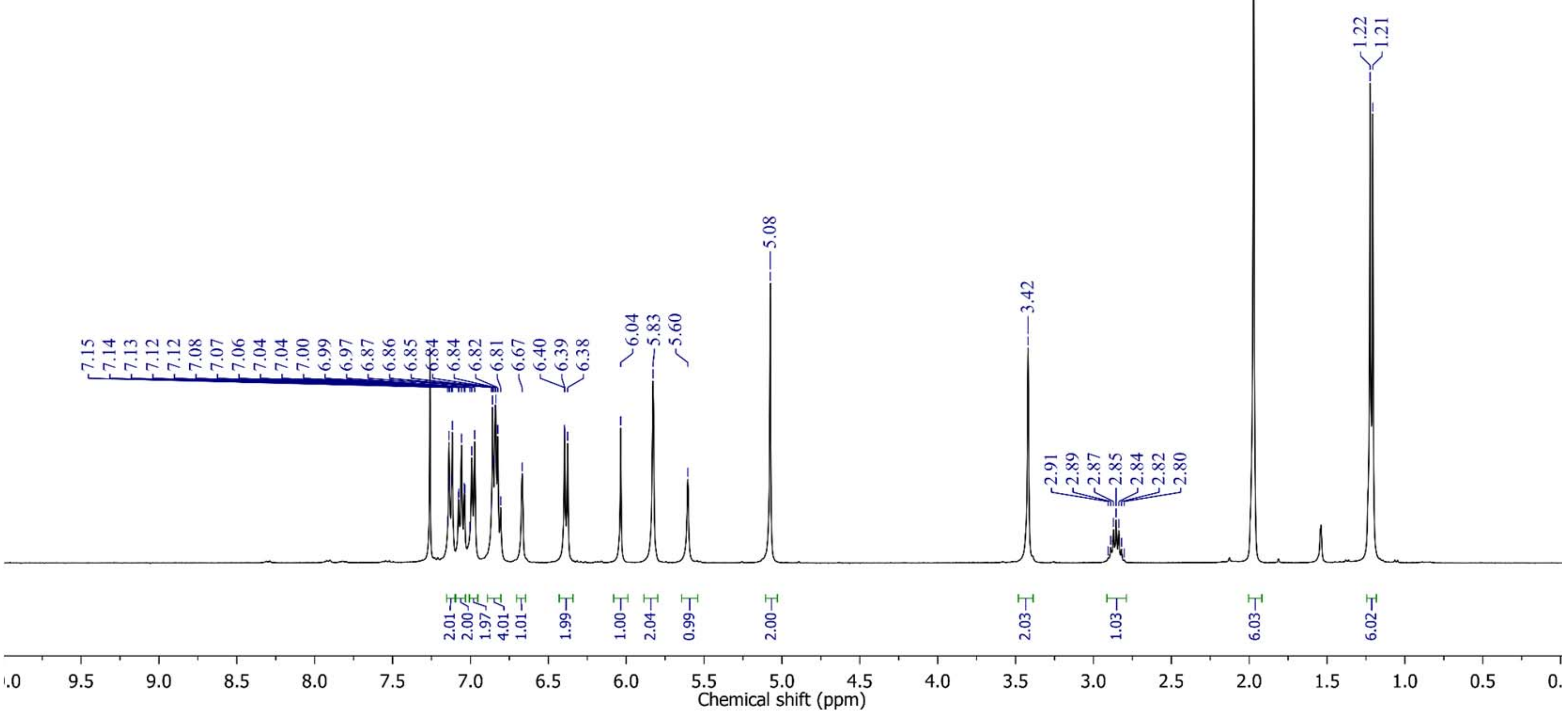

3-(9-(3,5-Dimethylbenzyl)-9,10-dihydroacridin-9-yl)-5-((4-isopropylphenoxy)methyl)isoxazole (33i), $^{13}C\{^{1}H\}$ NMR, $CDCl_3$, 100 MHz

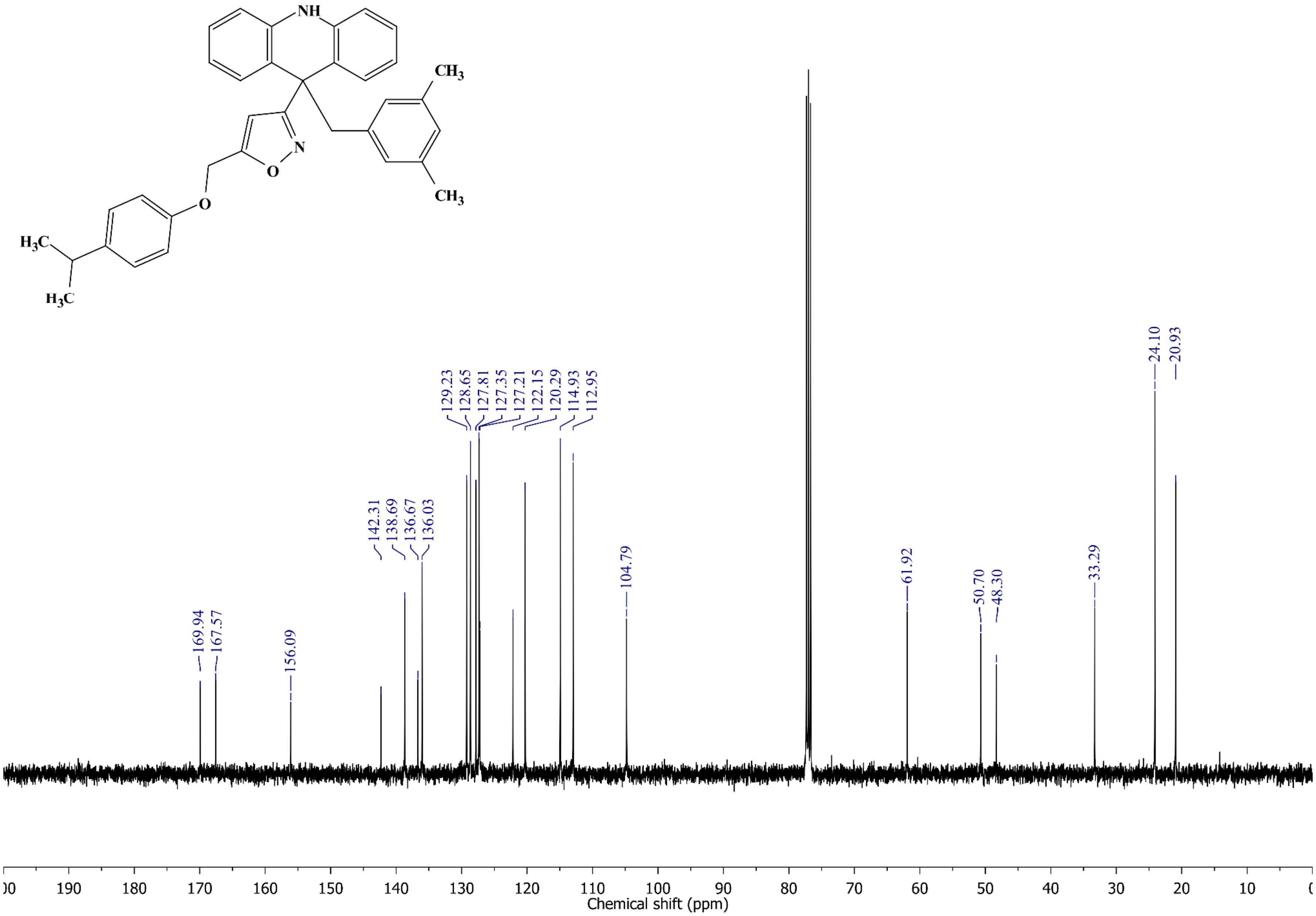

3-(9-(3,5-Dimethylbenzyl)-9,10-dihydroacridin-9-yl)-5-((4-isopropylphenoxy)methyl)isoxazole (33i), DEPT, $CDCl_3$, 100 MHz

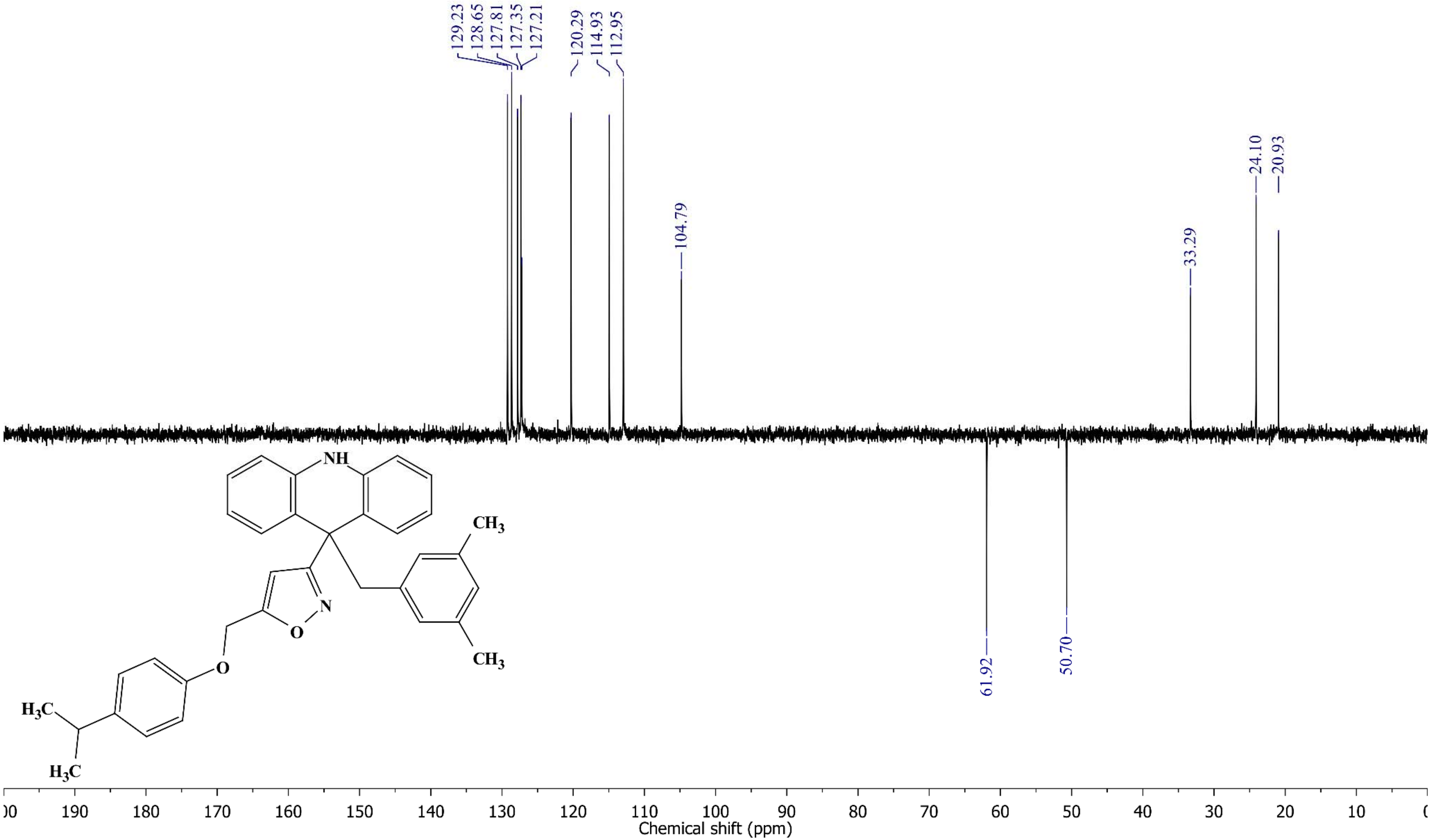

Methyl 3-(9-(1,4-dioxan-2-yl)-2-methyl-9,10-dihydroacridin-9-yl)isoxazole-5-carboxylate (33j), ^{1}H NMR, C_6D_6, 400 MHz

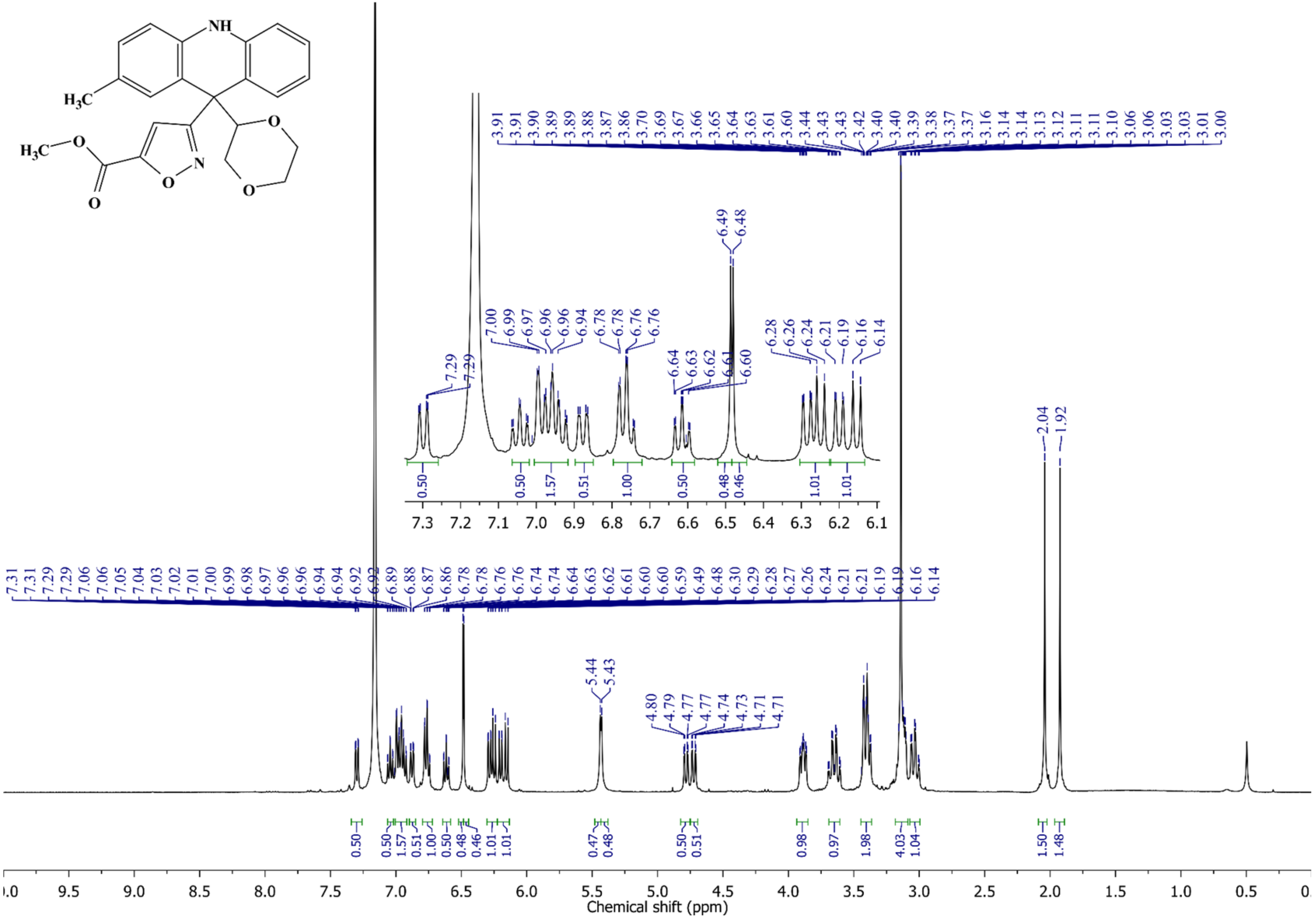

Methyl 3-(9-(1,4-dioxan-2-yl)-2-methyl-9,10-dihydroacridin-9-yl)isoxazole-5-carboxylate (33j), $^{13}C\{^1H\}$ NMR, C_6D_6, 100 MHz

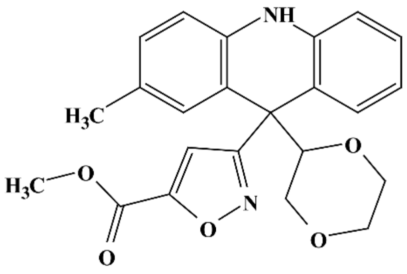

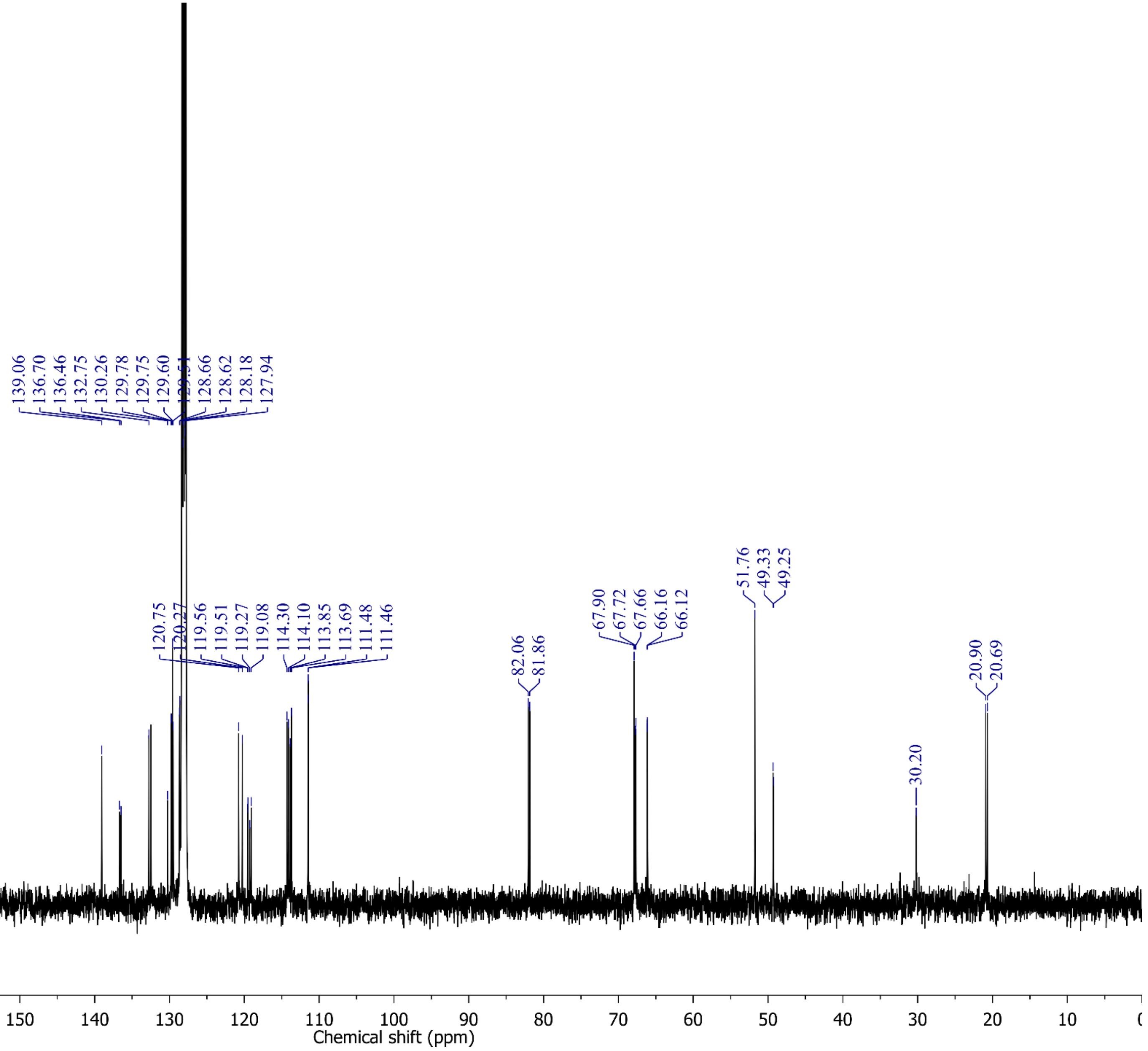

Methyl 3-(9-(1,4-dioxan-2-yl)-2-methyl-9,10-dihydroacridin-9-yl)isoxazole-5-carboxylate (33j), DEPT, C_6D_6, 100 MHz

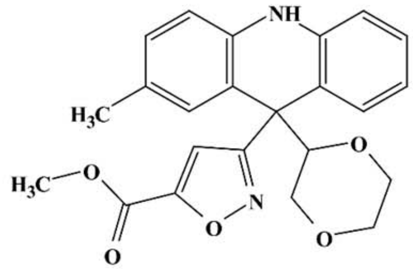

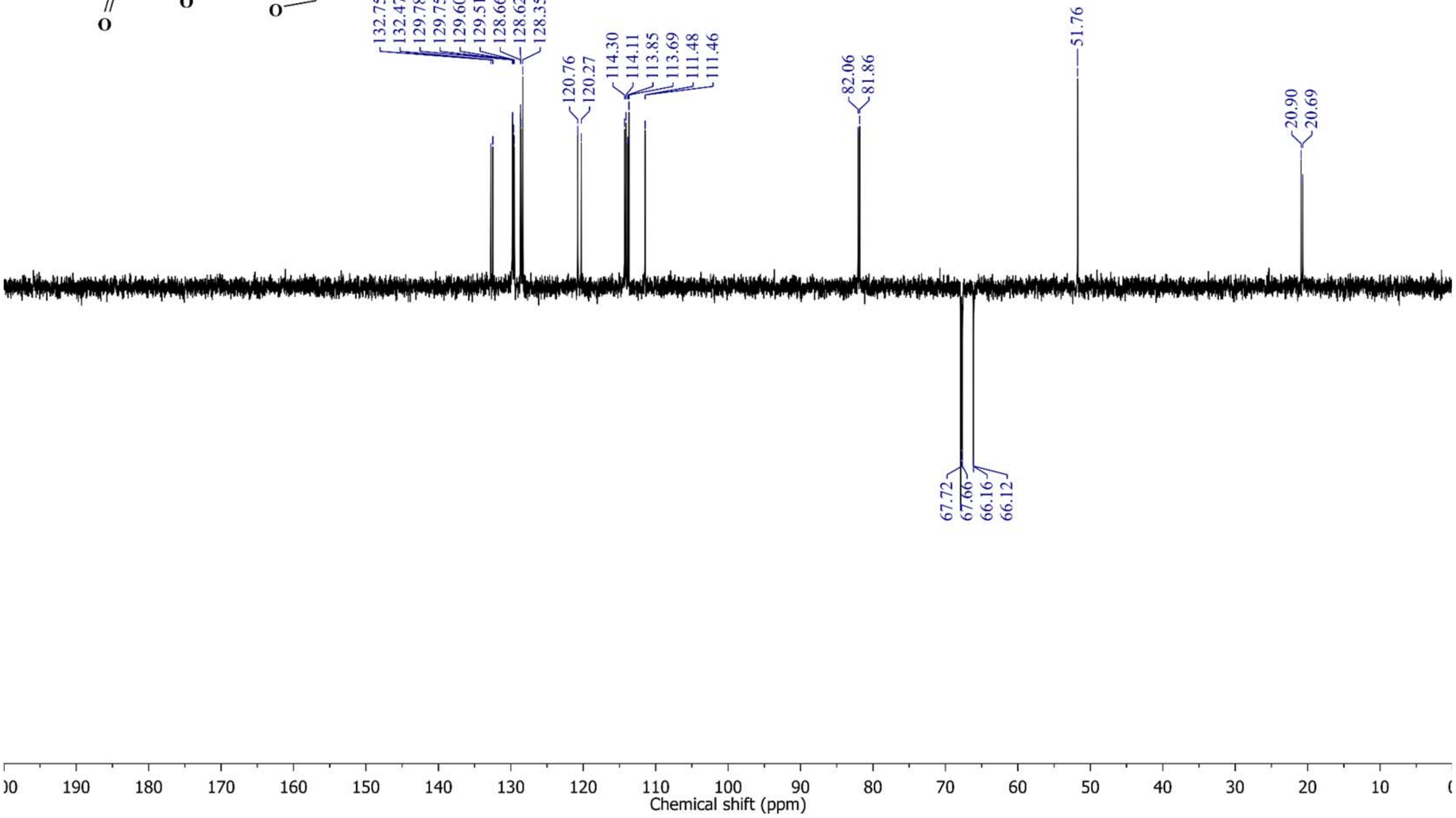

3-(9-(*tert*-Butoxymethyl)-2-methyl-9,10-dihydroacridin-9-yl)-4,5-dihydroisoxazole-5-carbonitrile (33k), ^{1}H NMR, C_6D_6, 400 MHz

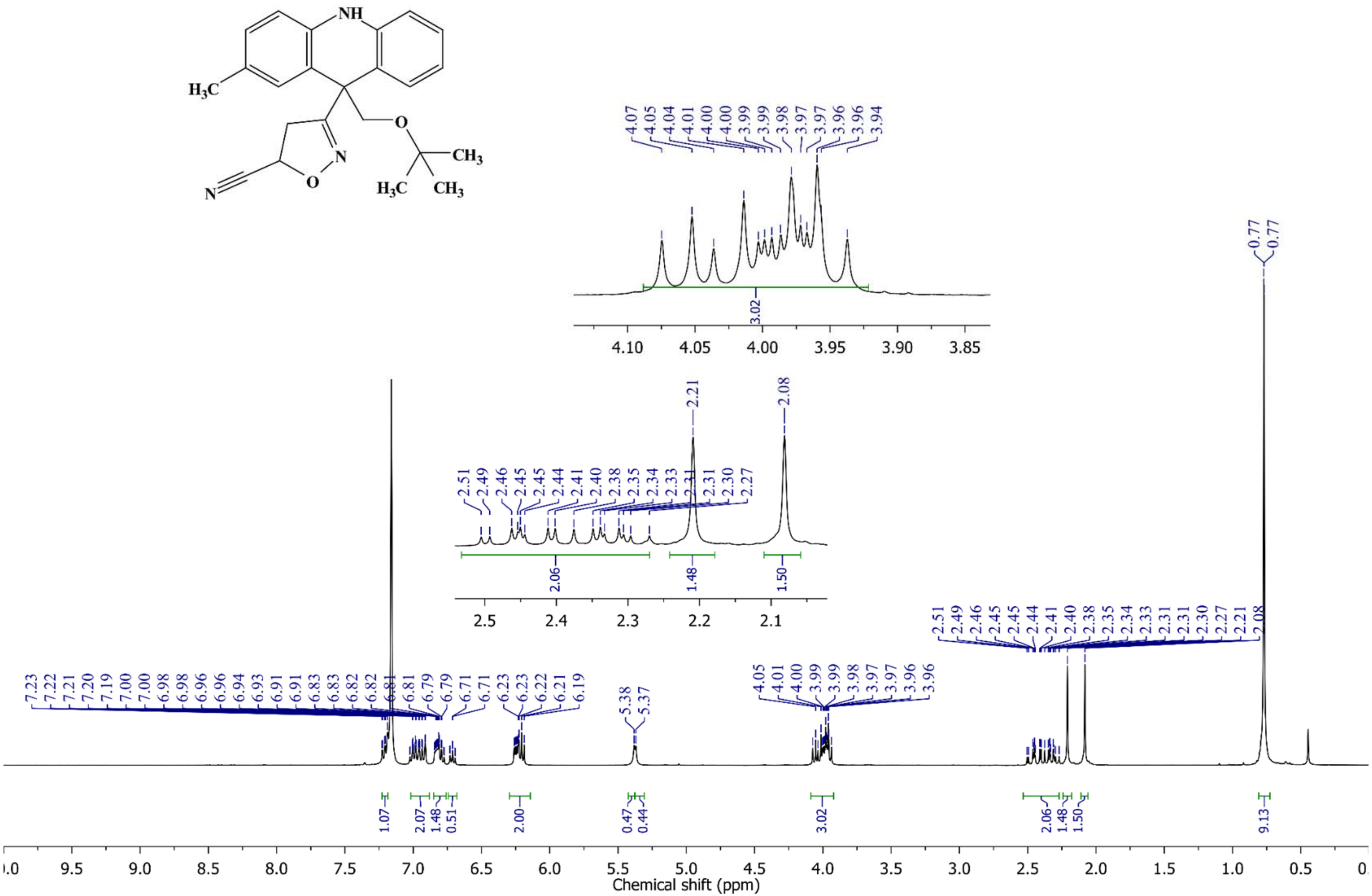

3-(9-(*tert*-Butoxymethyl)-2-methyl-9,10-dihydroacridin-9-yl)-4,5-dihydroisoxazole-5-carbonitrile (33k), $^{13}C\{^{1}H\}$ NMR, C_6D_6, 100 MHz

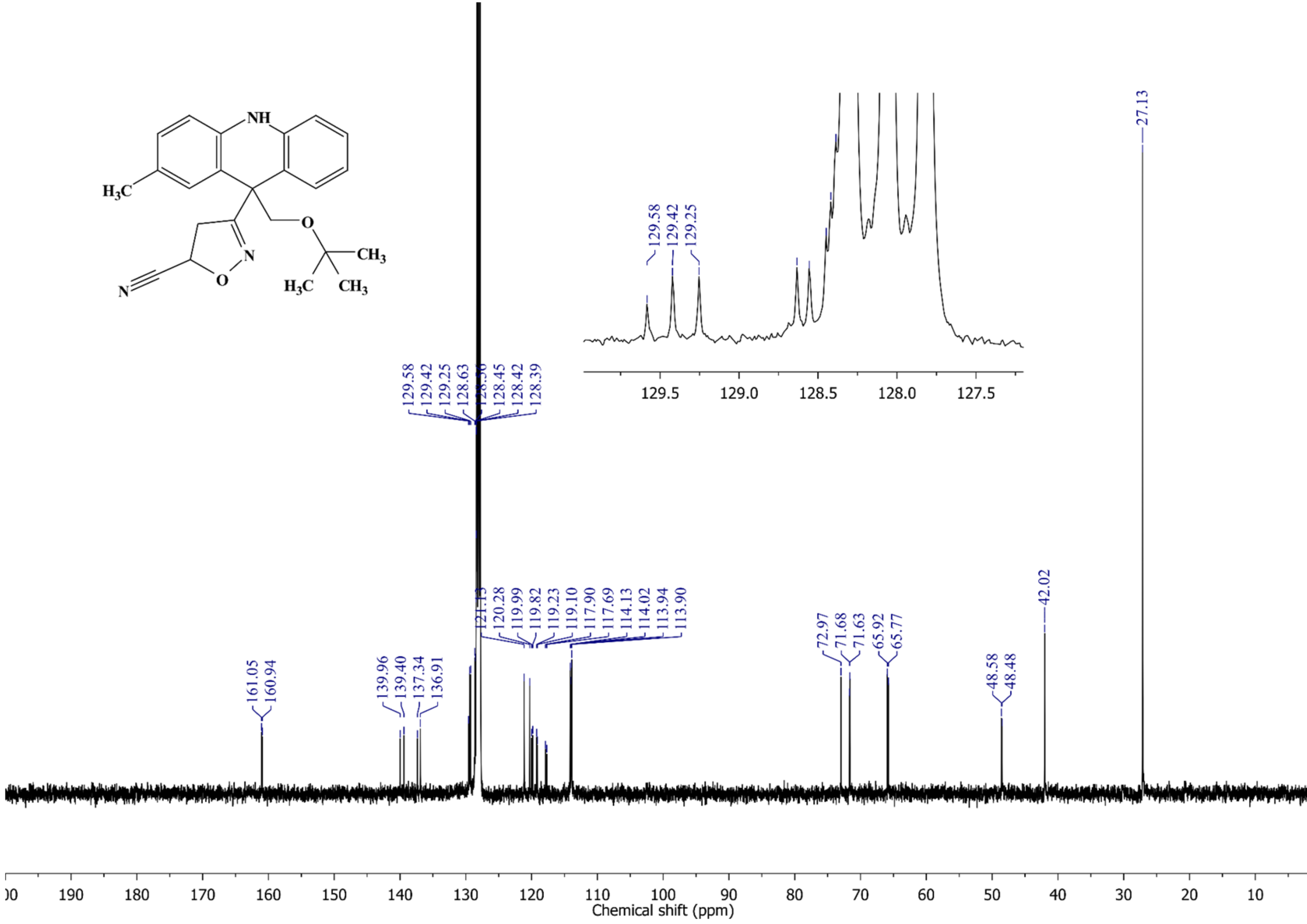

3-(9-(*tert*-Butoxymethyl)-2-methyl-9,10-dihydroacridin-9-yl)-4,5-dihydroisoxazole-5-carbonitrile (33k), DEPT, C_6D_6, 100 MHz

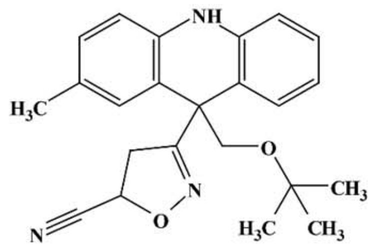

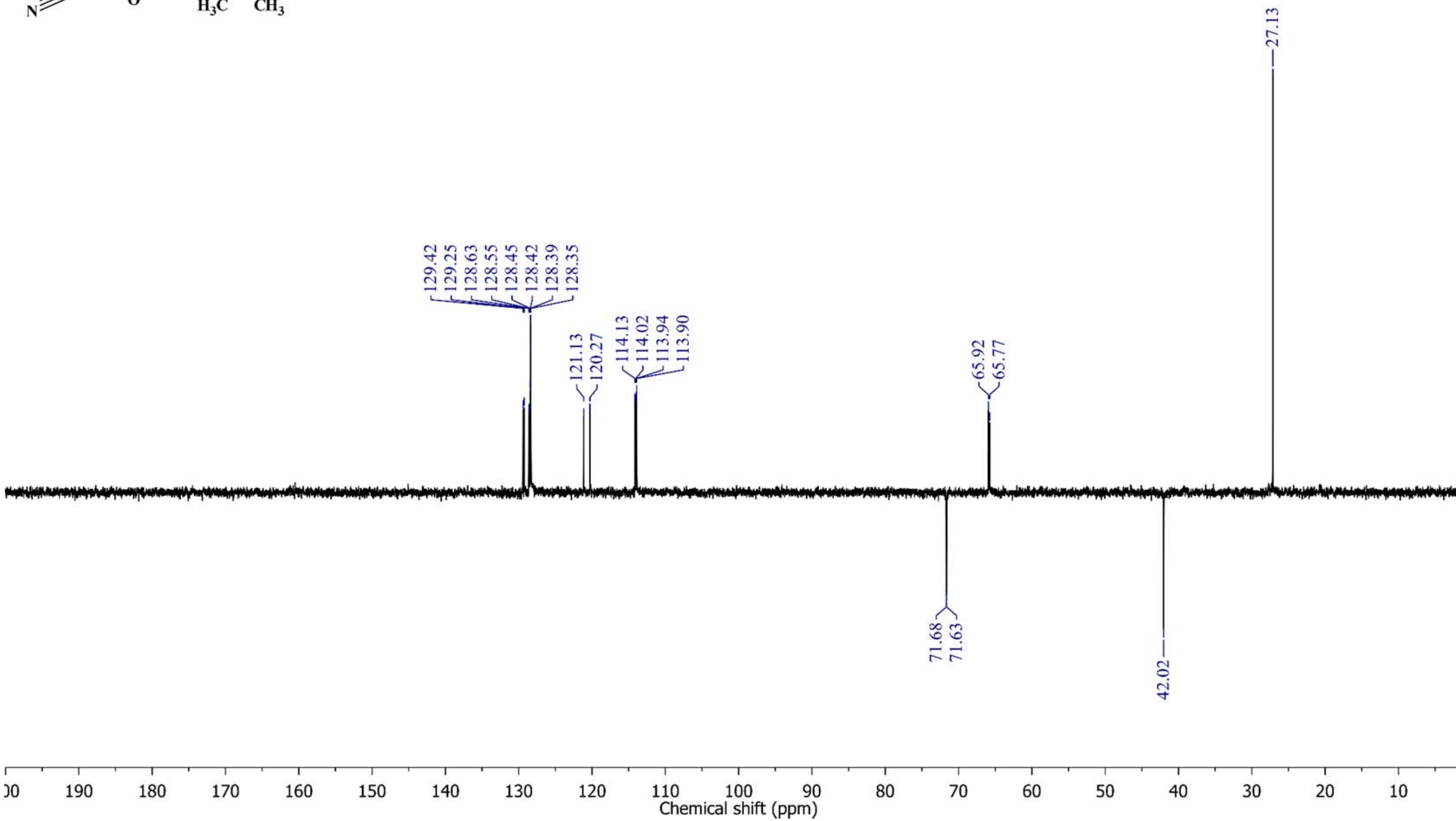

4-((3-(9-Benzyl-2-methyl-9,10-dihydroacridin-9-yl)isoxazol-5-yl)methyl)-5-methoxy-3-(naphthalen-2-yl)isoxazole (33l), ^{1}H NMR, $CDCl_3$, 400 MHz

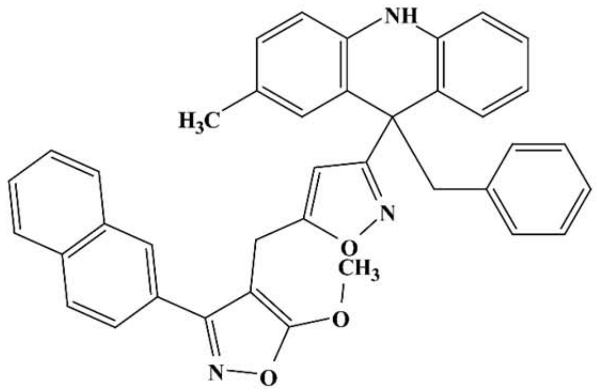

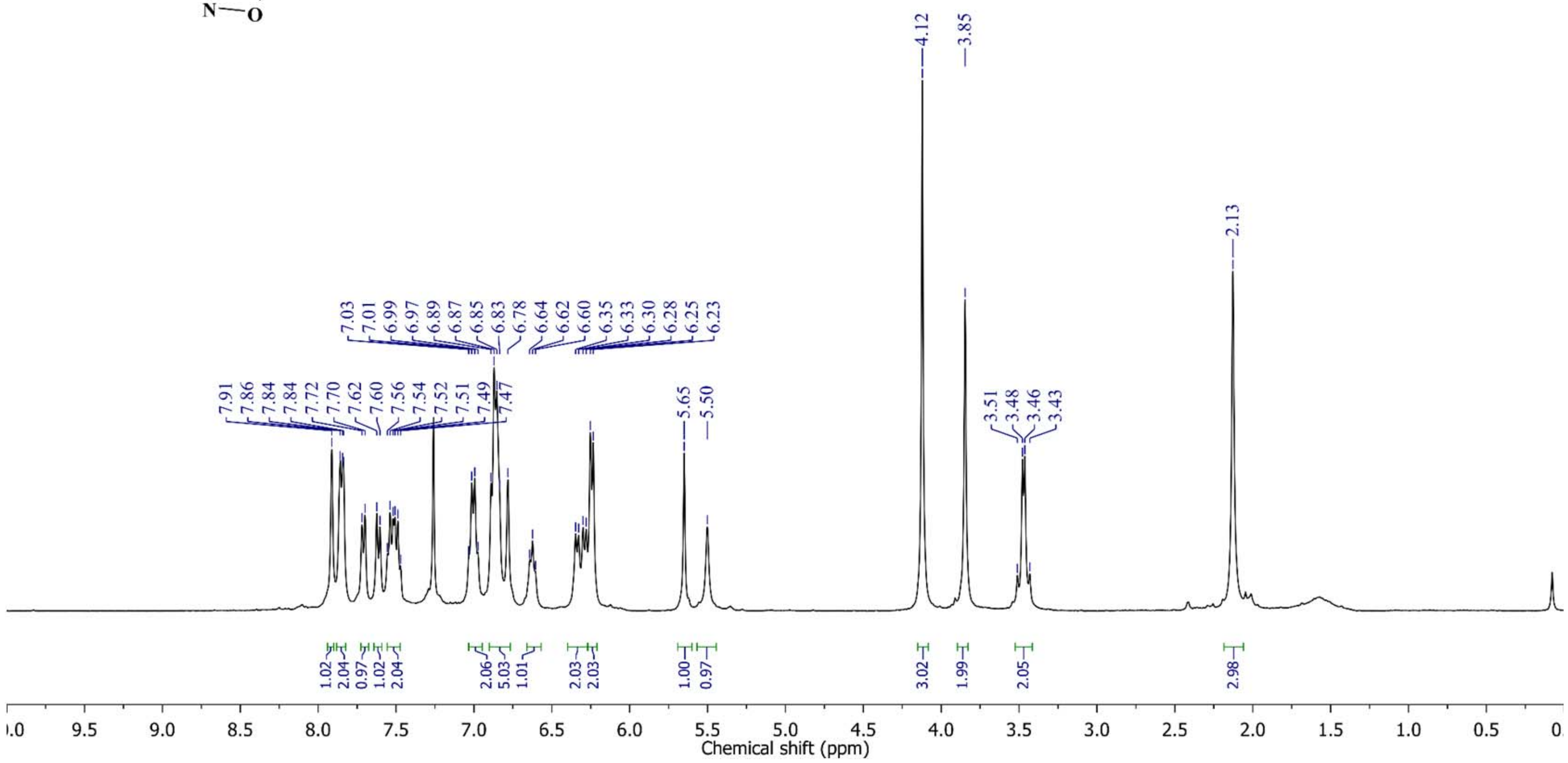

4-((3-(9-Benzyl-2-methyl-9,10-dihydroacridin-9-yl)isoxazol-5-yl)methyl)-5-methoxy-3-(naphthalen-2-yl)isoxazole (33l), ^{1}H NMR, C_6D_6, 400 MHz

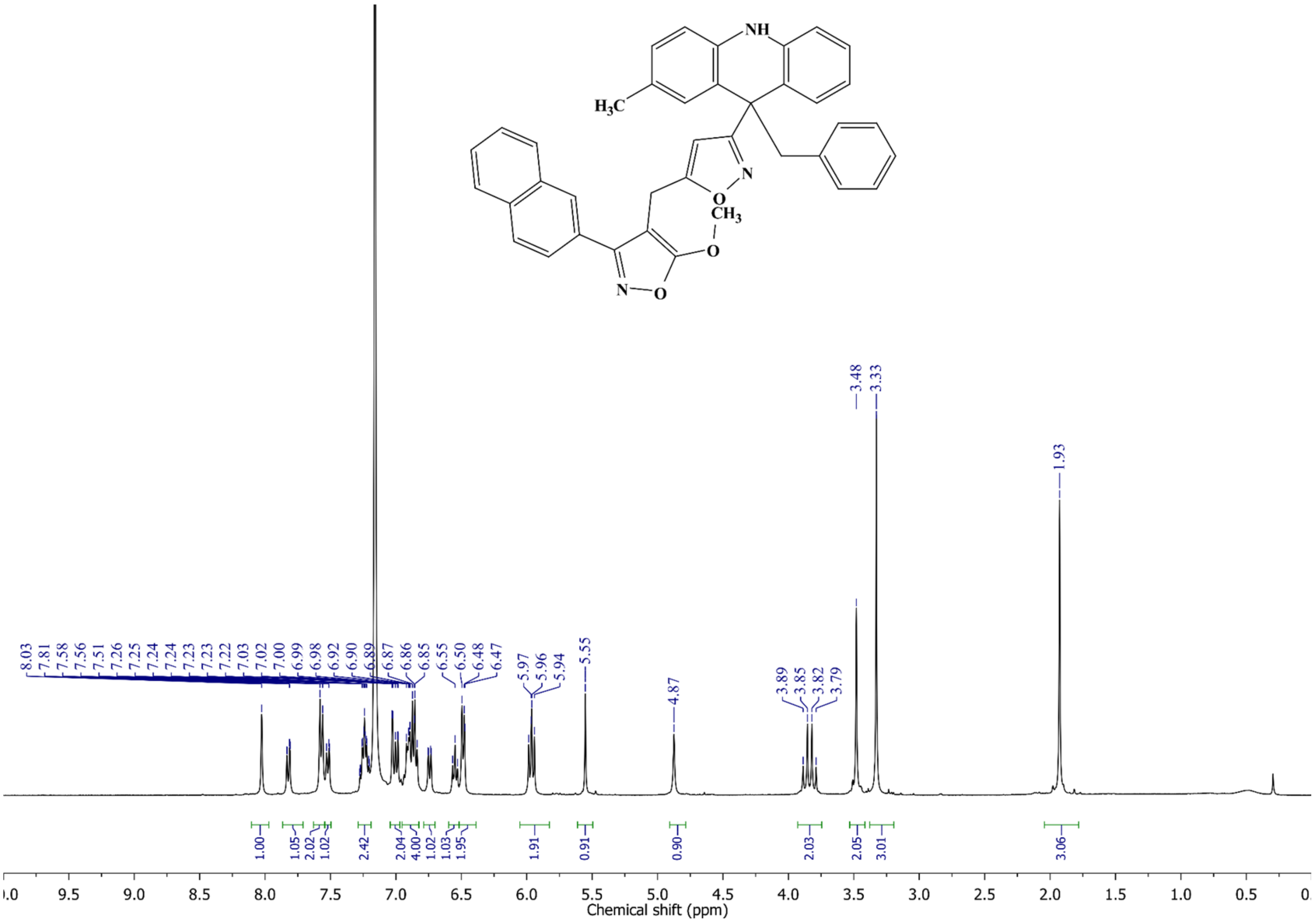

4-((3-(9-Benzyl-2-methyl-9,10-dihydroacridin-9-yl)isoxazol-5-yl)methyl)-5-methoxy-3-(naphthalen-2-yl)isoxazole (33l), $^{13}C\{^{1}H\}$ NMR, C_6D_6, 100 MHz

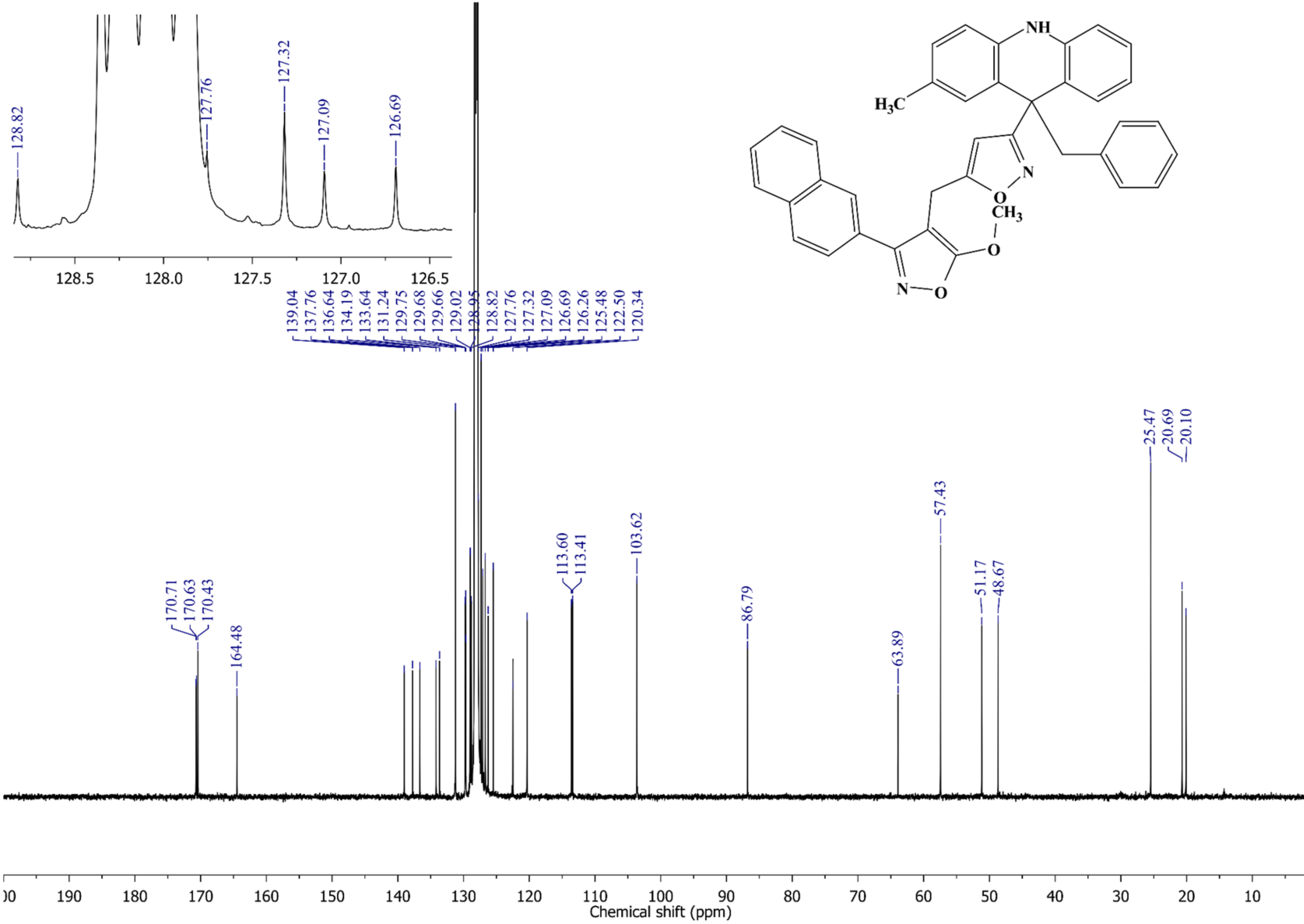

4-((3-(9-Benzyl-2-methyl-9,10-dihydroacridin-9-yl)isoxazol-5-yl)methyl)-5-methoxy-3-(naphthalen-2-yl)isoxazole (33l), DEPT, C_6D_6, 100 MHz

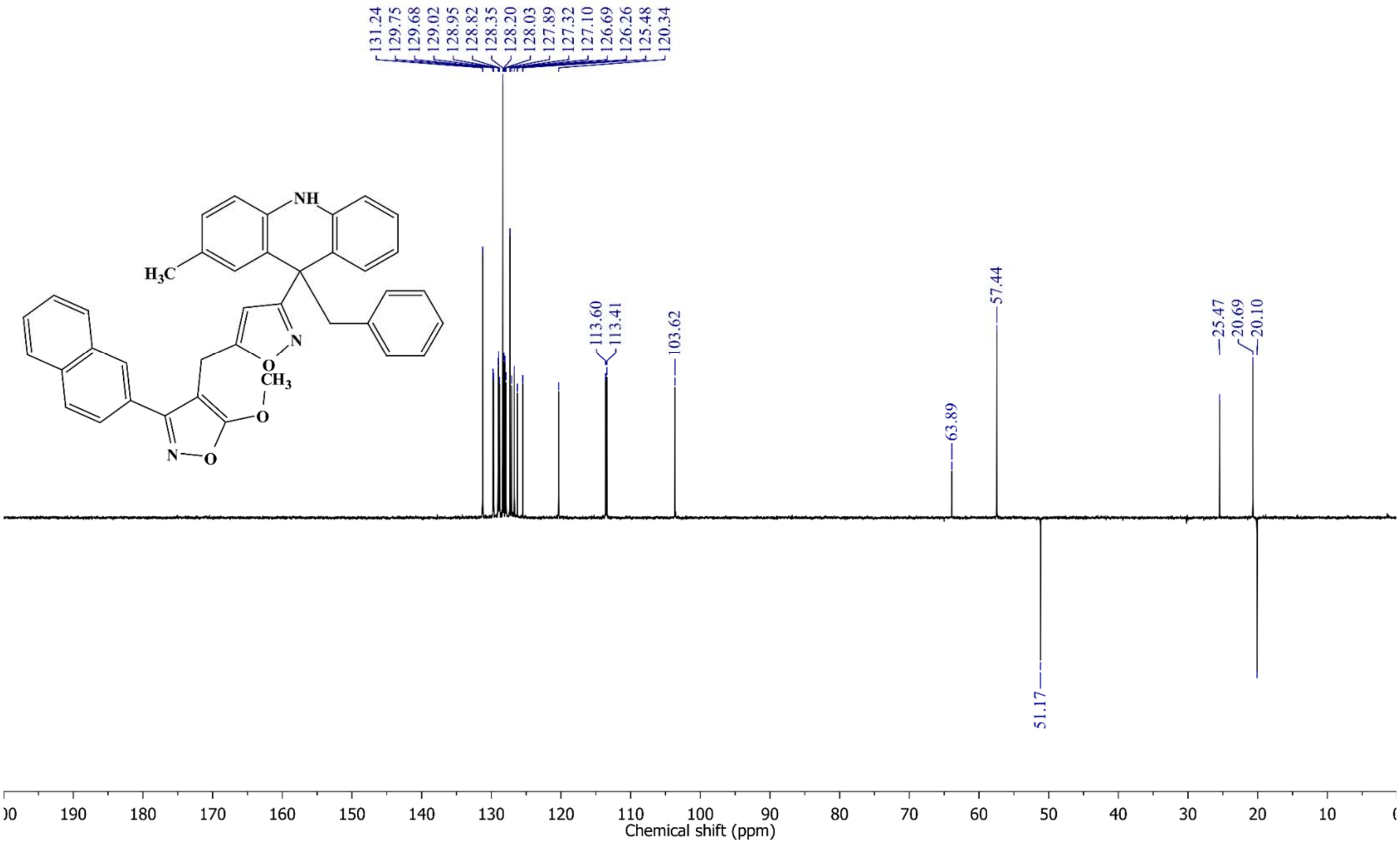

4-((3-(9-(3,5-Dimethylbenzyl)-2-methyl-9,10-dihydroacridin-9-yl)isoxazol-5-yl)methyl)-5-methoxy-3-(naphthalen-2-yl)isoxazole (33m), ^{1}H NMR, C_6D_6, 400 MHz

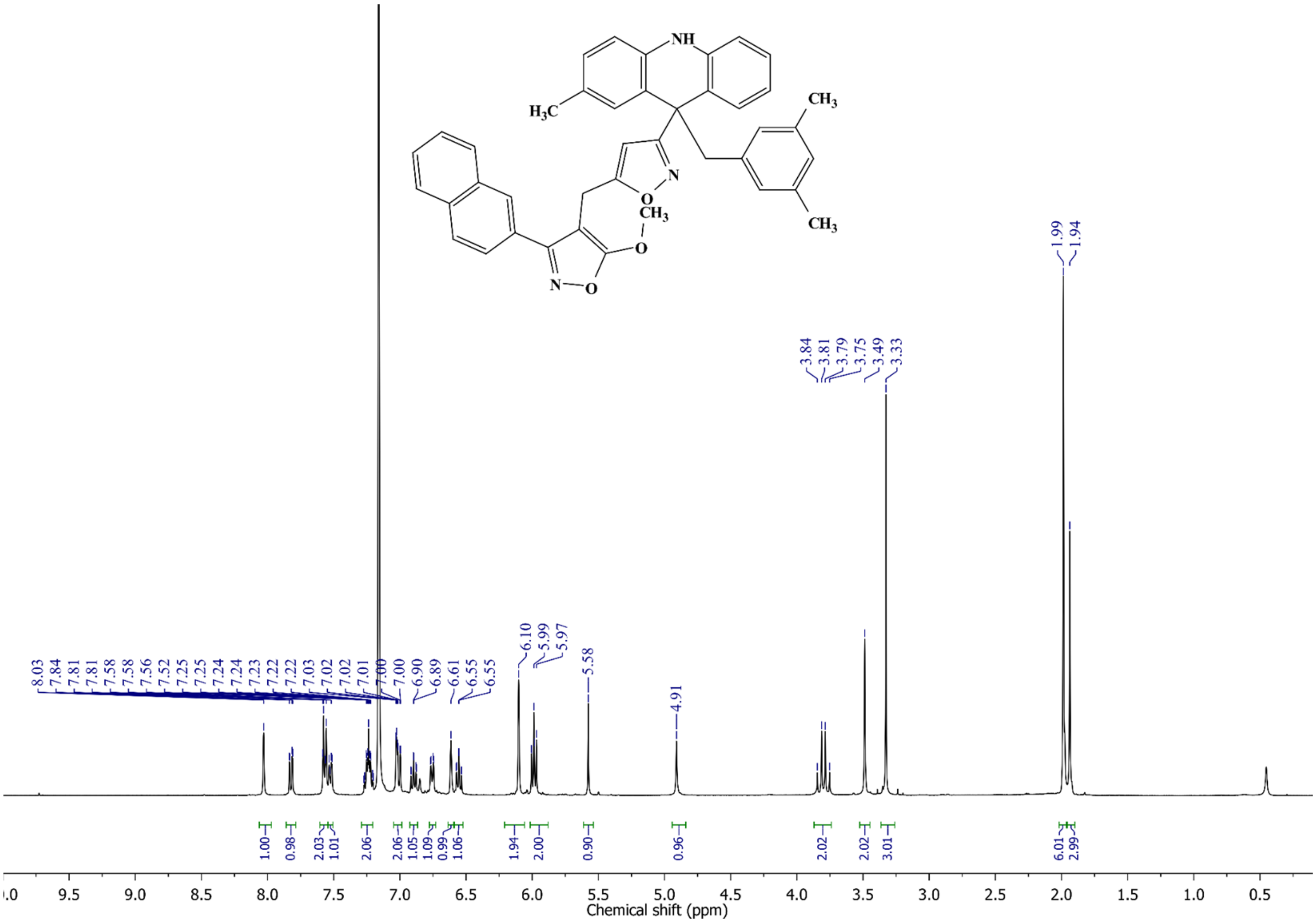

4-((3-(9-(3,5-Dimethylbenzyl)-2-methyl-9,10-dihydroacridin-9-yl)isoxazol-5-yl)methyl)-5-methoxy-3-(naphthalen-2-yl)isoxazole (33m), $^{13}C\{^1H\}$ NMR, C_6D_6, 100 MHz

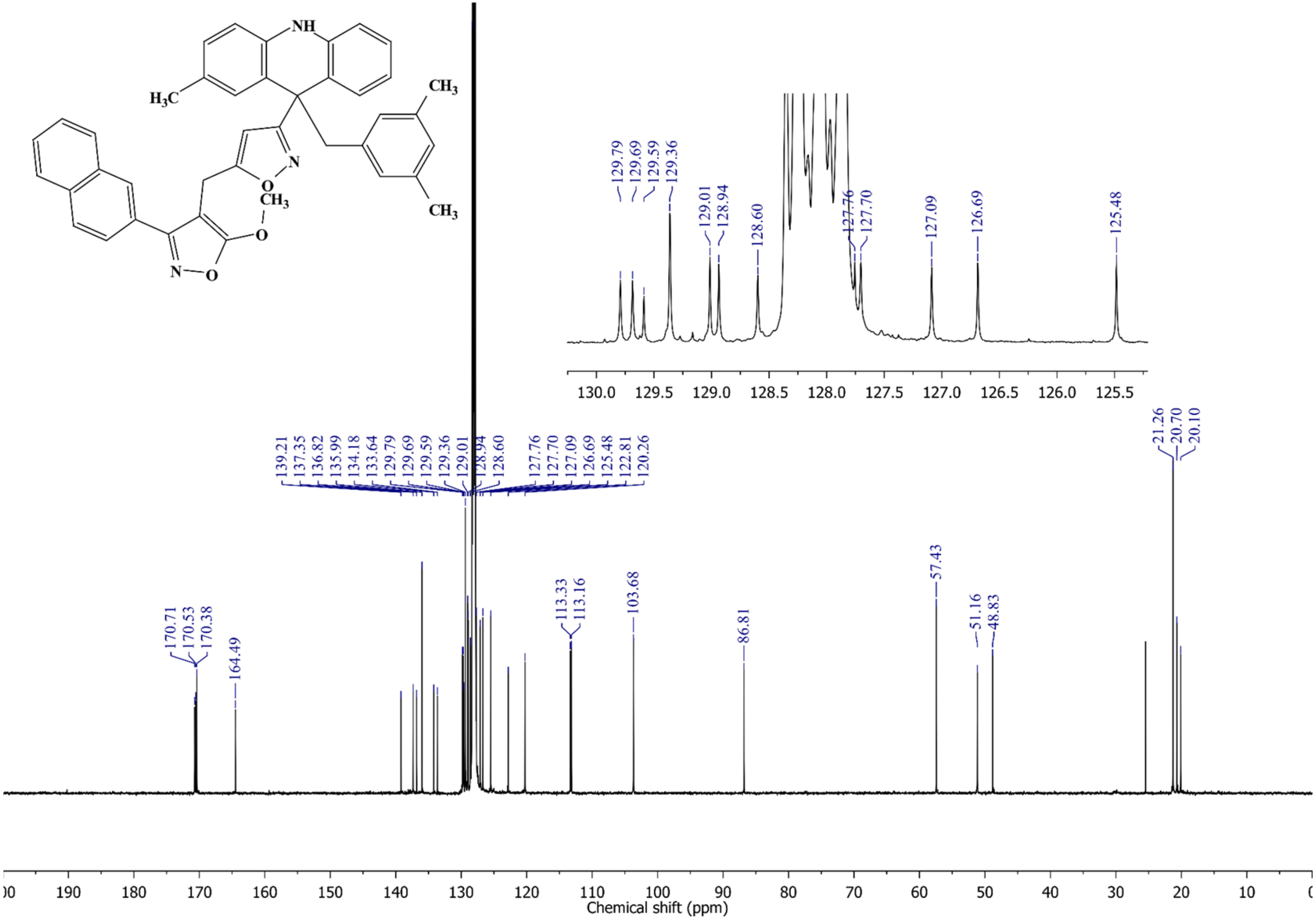

4-((3-(9-(3,5-Dimethylbenzyl)-2-methyl-9,10-dihydroacridin-9-yl)isoxazol-5-yl)methyl)-5-methoxy-3-(naphthalen-2-yl)isoxazole (33m), DEPT, C_6D_6, 100 MHz

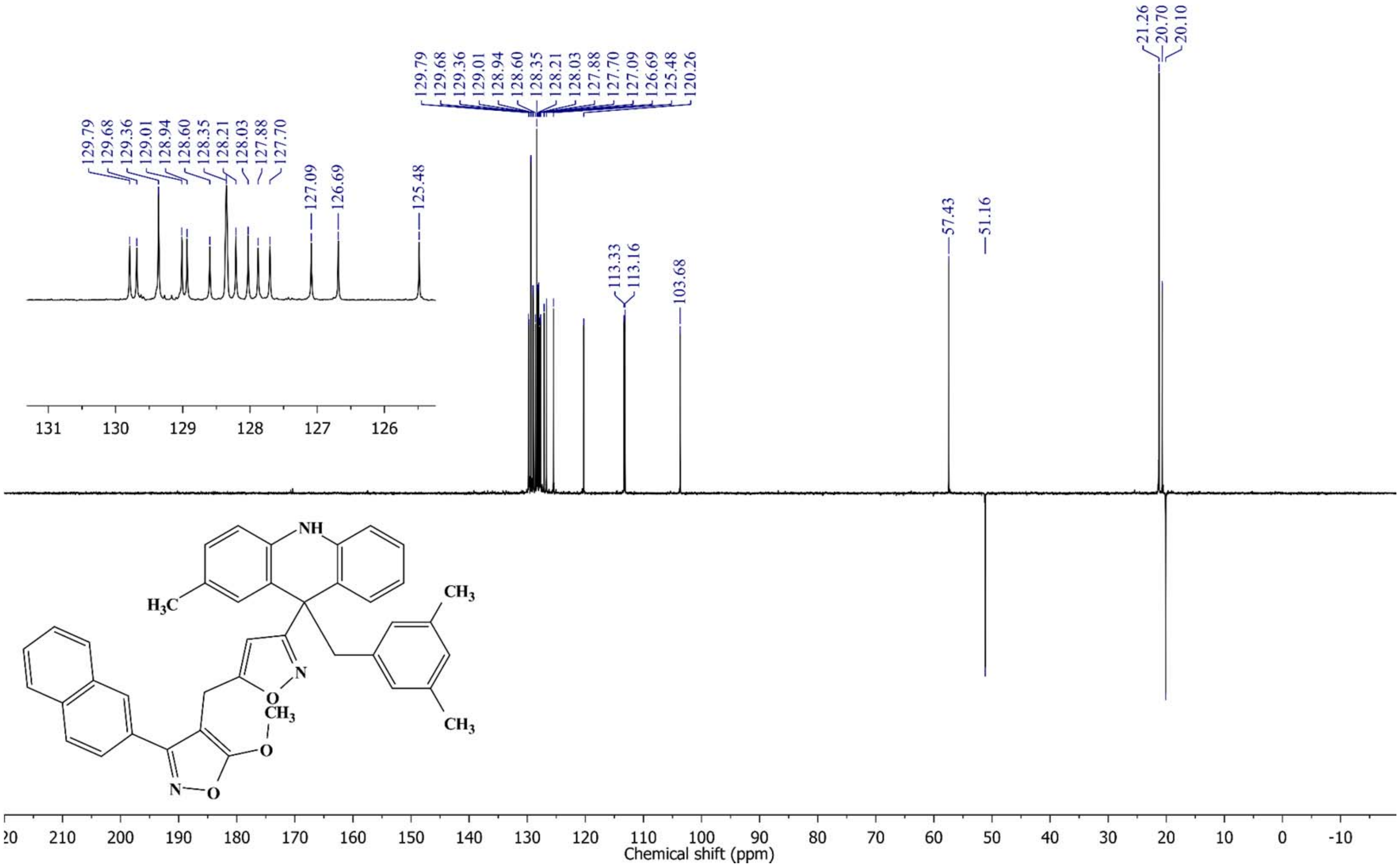

5-(*tert*-Butoxy)-3-(9-(3,5-dimethylbenzyl)-9-phenyl-9,10-dihydroacridin-2-yl)isoxazole (33n), ^{1}H NMR, C_6D_6, 400 MHz

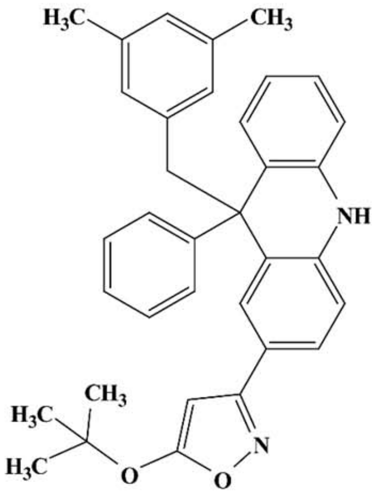

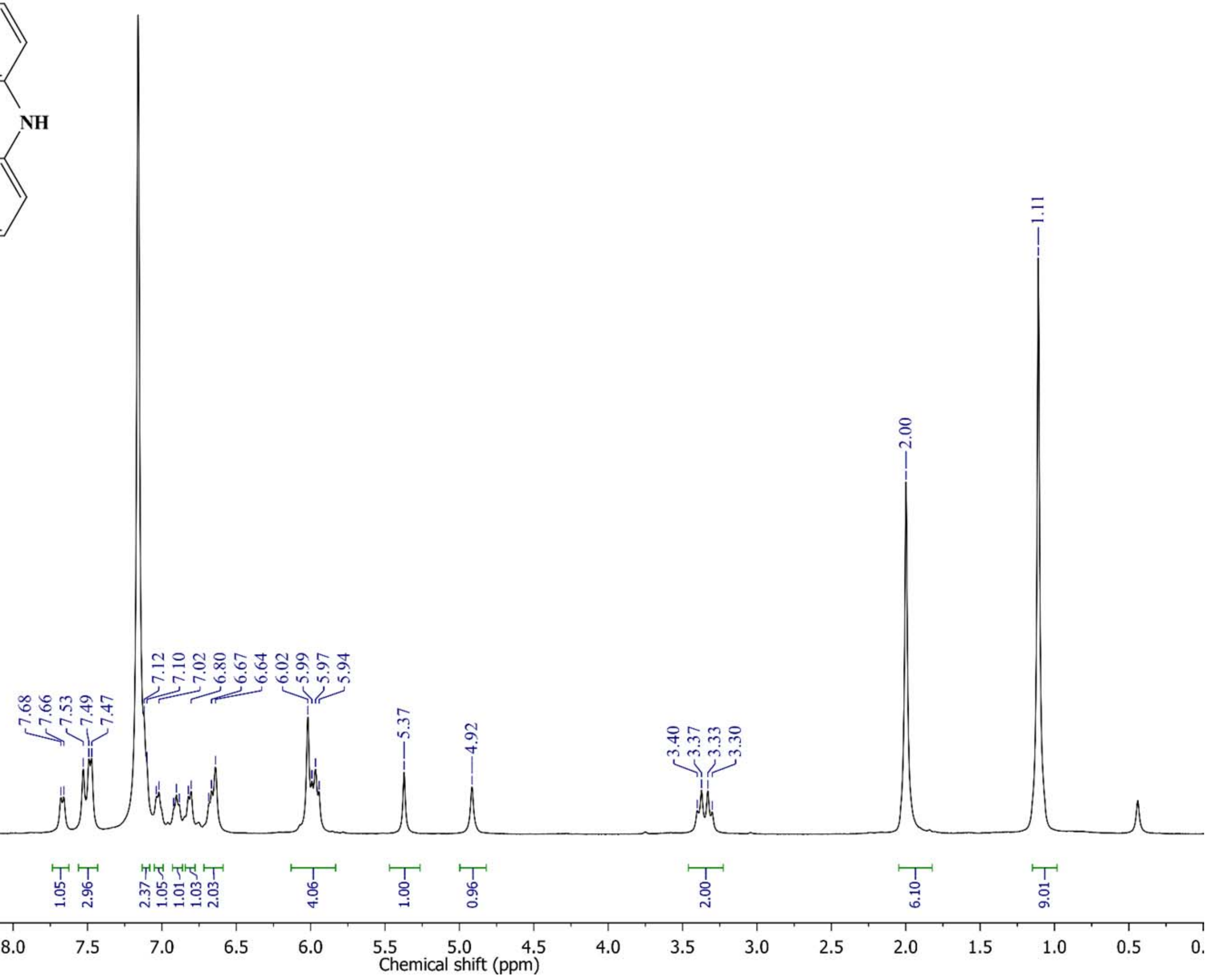

5-(*tert*-Butoxy)-3-(9-(3,5-dimethylbenzyl)-9-phenyl-9,10-dihydroacridin-2-yl)isoxazole (33n), $^{13}C\{^{1}H\}$ NMR, C_6D_6, 100 MHz

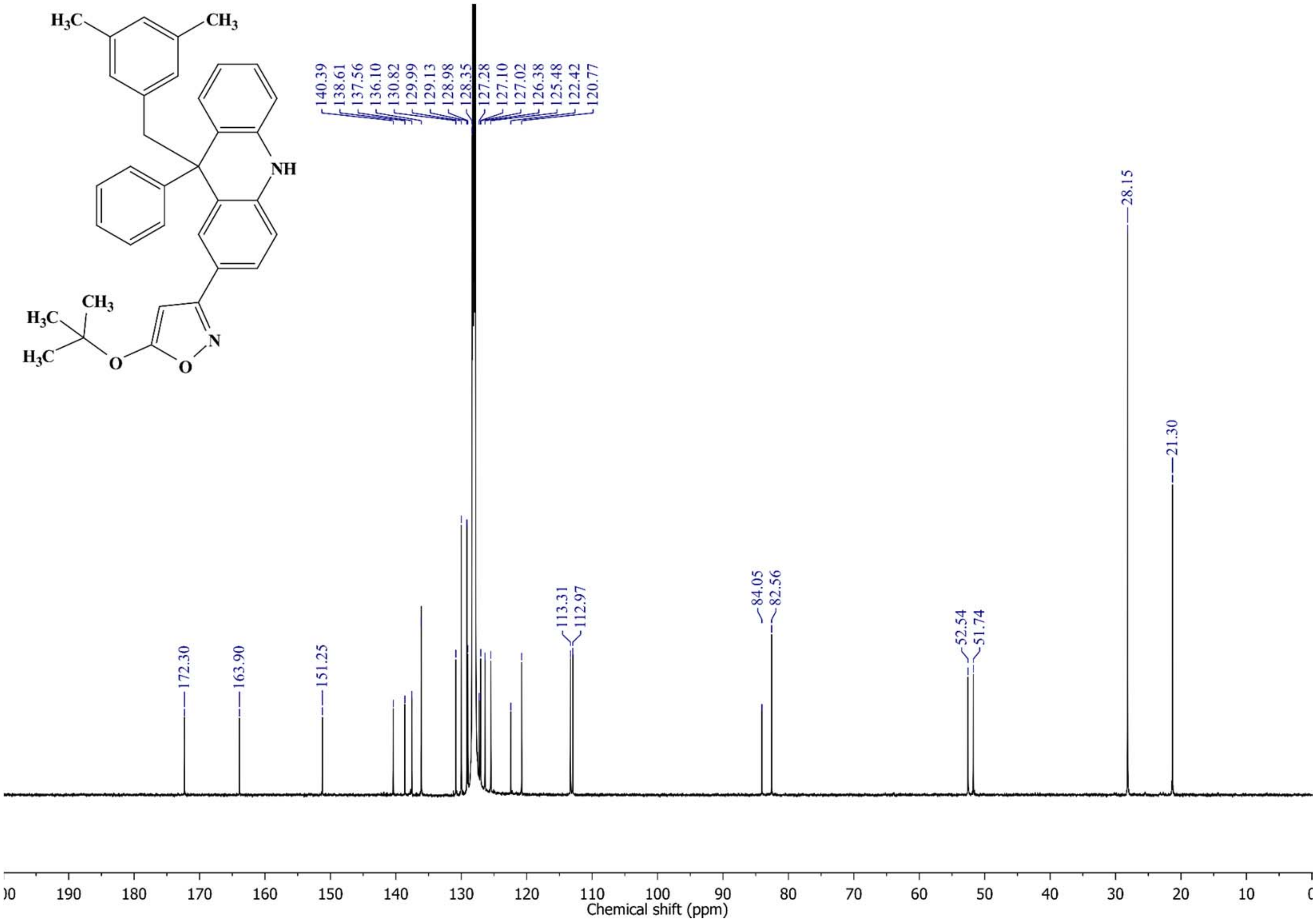

5-(*tert*-Butoxy)-3-(9-(3,5-dimethylbenzyl)-9-phenyl-9,10-dihydroacridin-2-yl)isoxazole (33n), DEPT, C_6D_6, 100 MHz

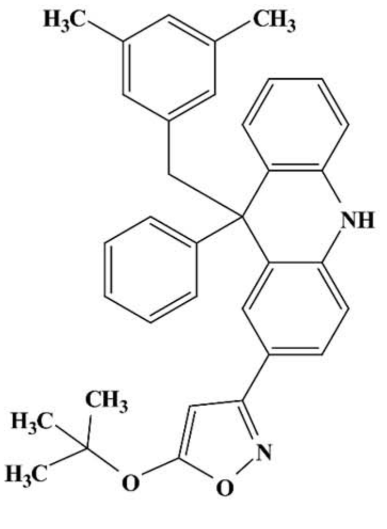

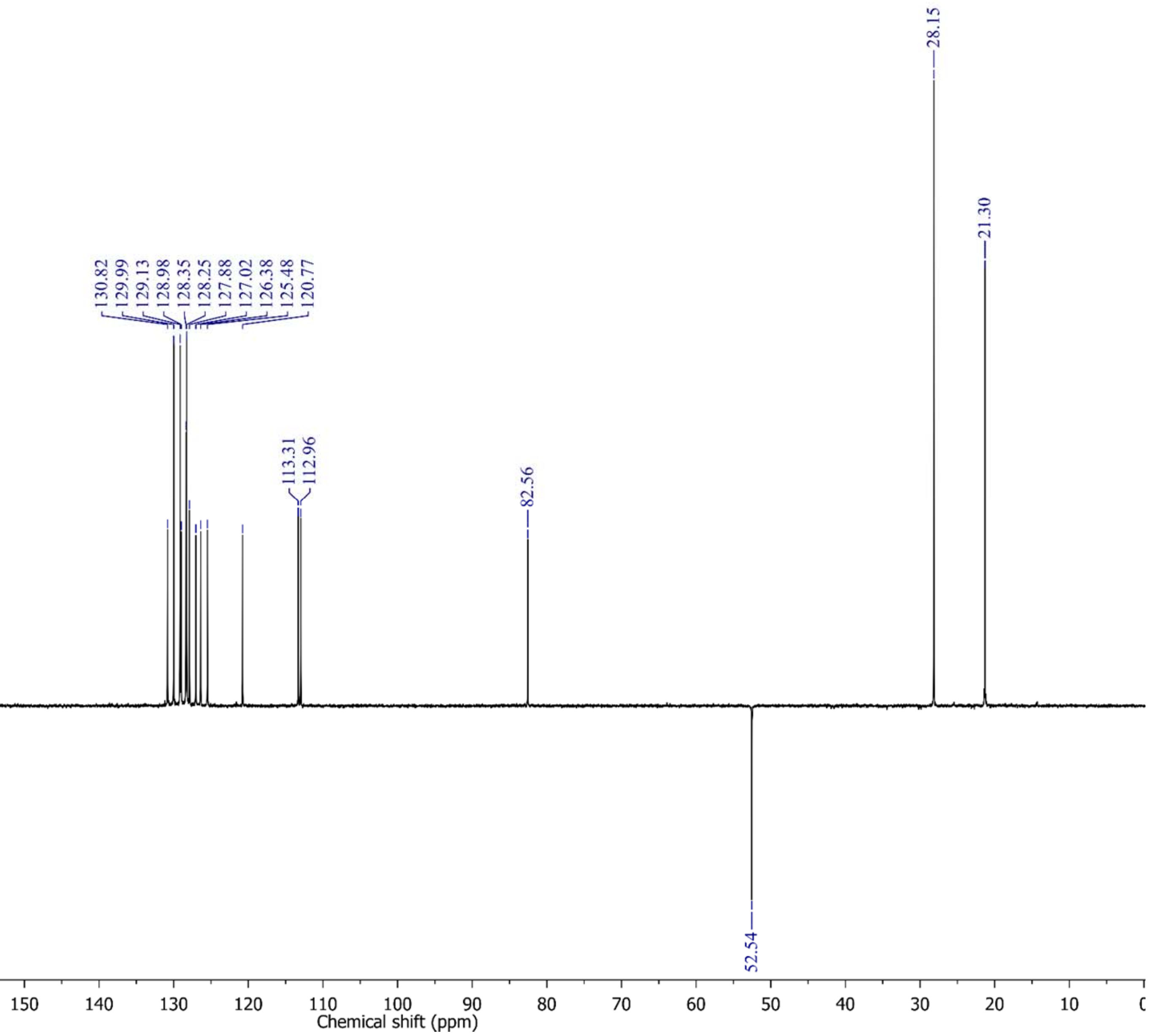

Computational Details

All calculations were performed by using the Gaussian 16 suite of quantum chemical programs[1] at Resource center "Computer center of Saint Petersburg State University". Geometry optimizations of molecules were performed with the B3LYP[2]-D3[3] density functional method and 6-311+G(d,p) basis set using SMD[4] solvent model. Stationary points on the respective potential-energy surfaces were characterized at the same level of theory by evaluating the corresponding Hessian indices. Careful verification of the unique imaginary frequency for the transition state was carried out to check whether the frequency indeed pertains to the desired reaction coordinate.

Table S8. B3LYP-D3/6-311+G(d,p), SMD for MeCN.

Absolute Energies (au), Cartesian Coordinates of stationary points

Molecule **15**

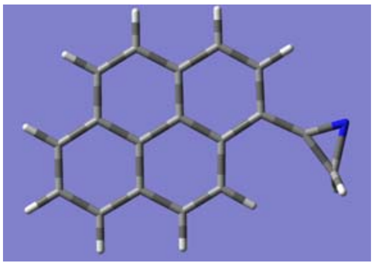

E = -747.496556, H (0K) = -747.264040,
H (373K) = -747.249912,
G (373K) = -747.303758 au.
Imaginary frequency = 0.

C	4.2909650	-1.6199910	0.0005510
N	4.7718550	-0.1583350	-0.0004360
C	3.5268330	-0.3851910	-0.0001150
H	4.4280540	-2.1784230	0.9233100
H	4.4279610	-2.1795800	-0.9215170
C	2.3383680	1.7410360	-0.0001090
C	1.1888790	2.5075490	-0.0000260
C	-0.0783380	1.8956010	0.0000360
C	-0.1647650	0.4708760	0.0000140
C	1.0229320	-0.3255720	-0.0000290
C	2.2777100	0.3357890	-0.0000860
C	-1.2865150	2.6681940	0.0001050
C	-1.4469600	-0.1552090	0.0000130
C	-2.6330740	0.6381690	0.0000550
C	-2.5080500	2.0676500	0.0001170
C	-3.8835670	-0.0005560	0.0000250
H	-4.7842320	0.6040940	0.0000560
C	-3.9724690	-1.3905020	-0.0000490
C	-2.8200850	-2.1733700	-0.0000830
C	-1.5497320	-1.5766310	-0.0000490
C	-0.3412500	-2.3485860	-0.0000700
C	0.8838080	-1.7558490	-0.0000600
H	1.7734830	-2.3705090	-0.0000650
H	-0.4213600	-3.4306320	-0.0000950
H	-1.2043250	3.7497910	0.0001450
H	3.3104860	2.2213290	-0.0001540
H	1.2542140	3.5899880	-0.0000060

TS15-16

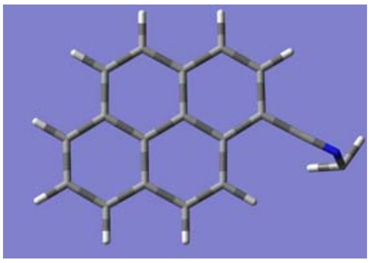

E = -747.410863, H (0K) = -747.183330,
H (373K) = -747.168683,
G (373K) = -747.223862 au.
Imaginary frequency = 1.

C	-4.9089340	-1.2373710	0.9814170
N	-4.4632770	-0.8934870	-0.5160780
C	-3.4375590	-0.3081460	-0.3310230
H	-4.0269570	-1.2084230	1.6382500
H	-5.5991110	-0.4302600	1.2568430
C	-2.2928240	1.8135630	-0.1620940
C	-1.1326530	2.5448680	-0.0537460
C	0.1261580	1.9057000	-0.0065940
C	-0.9972550	-0.2961450	-0.1583820
C	-2.2454460	0.3973100	-0.2158590
C	1.3414910	2.6526220	0.0930580
C	2.6545950	0.5979940	0.0878130
C	2.5523720	2.0266910	0.1367270
C	3.8916920	-0.0648400	0.1365730
H	4.8012790	0.5211700	0.2119480
C	3.9558070	-1.4557540	0.0900370
C	2.7923530	-2.2150510	-0.0041240
C	1.5334830	-1.5934880	-0.0554210
C	0.3157250	-2.3412660	-0.1471790
C	-0.8977380	-1.7211720	-0.1968750
H	-1.8035410	-2.3129460	-0.2639190
H	0.3744730	-3.4239870	-0.1749650
H	1.2794070	3.7344870	0.1330180
H	-3.2549400	2.3090010	-0.2059830
H	-1.1768180	3.6271030	-0.0117780
H	3.4661420	2.6066610	0.2109970
H	4.9192840	-1.9510380	0.1287260

H	-3.4141420	2.6644620	0.0001670
H	-4.9464330	-1.8669880	-0.0000810
H	-2.8948340	-3.2556190	-0.0001400

Molecule **16**

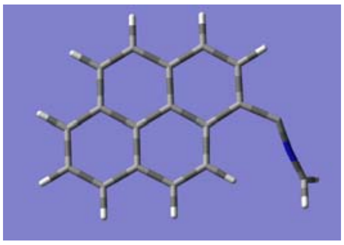

E = -747.479747, H (0K) = -747.249091,
H (373K) = -747.234481,
G (373K) = -747.289443 au.
Imaginary frequency = 0.

C	-4.7255370	-1.7400520	0.6540230
N	-4.0904000	-0.7499150	0.1584780
C	-3.5960760	0.2397560	-0.3585240
H	-4.7324180	-2.6884810	0.1272540
H	-5.2565010	-1.6181330	1.5920120
C	-2.1042240	2.1545210	-0.1973370
C	-0.8685970	2.7609450	-0.0407220
C	0.3028500	1.9909810	0.0240340
C	-1.0744390	-0.0622110	-0.1923900
C	-2.2386410	0.7561210	-0.2397650
C	1.5974180	2.5945880	0.1646580
C	2.6669020	0.4074220	0.1194860
C	2.7277280	1.8392980	0.2106110
C	3.8206820	-0.3903570	0.1671320
H	4.7878870	0.0884480	0.2777720
C	3.7321530	-1.7780120	0.0756860
C	2.4943630	-2.4001180	-0.0630790
C	1.3138330	-1.6409300	-0.1122520
C	0.0221950	-2.2441470	-0.2592030
C	-1.1136910	-1.4945780	-0.2973150
H	-2.0654680	-1.9915110	-0.4223750
H	-0.0362010	-3.3241800	-0.3451910
H	1.6539990	3.6758940	0.2331200
H	-2.9978890	2.7631040	-0.2734450
H	-0.7974780	3.8417180	0.0158270
H	3.7001980	2.3084720	0.3173190
H	4.6347810	-2.3778190	0.1132440
H	2.4300770	-3.4807150	-0.1346470
C	1.3920010	-0.2204190	-0.0207330
C	0.2030510	0.5692940	-0.0693490

H	2.8477550	-3.2977450	-0.0385930
C	1.4587430	-0.1719230	-0.0105000
C	0.1926500	0.4798050	-0.0624970

TS15-18

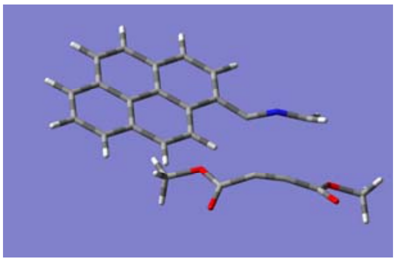

E = -1280.748986, H (0K) = -1280.403054,
H (373K) = -1280.376754,
G (373K) = -1280.460255 au.
Imaginary frequency = 1.

C	-3.6037600	1.5523620	-1.5477260
N	-2.3357250	1.4275670	-1.3198190
C	-1.3634300	0.9525870	-0.7822900
C	-2.6622570	-0.7619290	0.3488710
C	-3.8281020	-0.5414870	0.0356840
C	-5.2417240	-0.5285730	-0.1964580
O	-5.8012570	-1.1603230	-1.0749040
O	-5.8819660	0.2587350	0.6889610
C	-1.5749600	-1.4866950	0.9765620
O	-1.3406930	-2.6595370	0.7631750
O	-0.8687600	-0.7133550	1.8094530
C	0.2790920	-1.3297390	2.4423350
H	-4.1883870	2.1675440	-0.8728680
H	0.7555780	-0.5325960	3.0084980
H	-0.0408500	-2.1325380	3.1086150
H	0.9604310	-1.7211050	1.6863970
H	-3.9911620	1.2856320	-2.5244700
C	-7.3176320	0.3580010	0.5318180
H	-7.6470740	1.0231700	1.3274110
H	-7.5641720	0.7808670	-0.4437200
H	-7.7834550	-0.6227830	0.6395080
C	0.2350440	2.6233270	0.0108290
C	1.5011390	2.9937380	0.4320920
C	2.5812130	2.0990200	0.3407330
C	1.0547900	0.4052150	-0.6231100
C	-0.0050950	1.3371720	-0.5071630
C	3.9024420	2.4564680	0.7708190
C	4.7482790	0.2601510	0.1363140
C	4.9380380	1.5784950	0.6732210
C	5.8008720	-0.6626690	0.0321570
H	6.7912880	-0.3732340	0.3672200
C	5.5823980	-1.9353860	-0.4914270
C	4.3140080	-2.3168620	-0.9221440
C	3.2314690	-1.4266880	-0.8353470
C	1.9097340	-1.7847220	-1.2621790
C	0.8720610	-0.9098960	-1.1646030
H	-0.1160530	-1.2043830	-1.4981550
H	1.7487980	-2.7791900	-1.6647630

H	4.0588680	3.4496400	1.1782730
H	-0.5909970	3.3201770	0.0944020
H	1.6672780	3.9866230	0.8356800
H	5.9321620	1.8623590	1.0024440
H	6.4075690	-2.6351790	-0.5638760
H	4.1503040	-3.3090450	-1.3291230
C	3.4448530	-0.1220270	-0.3012360
C	2.3584180	0.7962910	-0.1987890

Molecule **18**

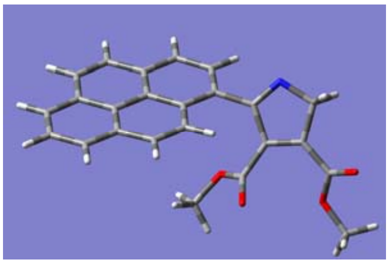

E = -1280.886803, H (0K) = -1280.534327,
H (373K) = -1280.509614,
G (373K) = -1280.589987 au.
Imaginary frequency = 0.

C	-3.4721420	1.8275620	-1.3787290
N	-2.0870600	2.2630780	-1.3180110
C	-1.4278040	1.3945180	-0.6233470
C	-2.2983520	0.2894520	-0.1400610
C	-3.5430080	0.5618880	-0.5843990
C	-4.7996850	-0.1917750	-0.3818340
O	-5.6655950	-0.2739400	-1.2287930
O	-4.8724260	-0.7392780	0.8337620
C	-1.8653680	-0.8944350	0.6526550
O	-2.1502680	-2.0398510	0.3837020
O	-1.0961110	-0.5226230	1.6798040
C	-0.4940510	-1.5858710	2.4585570
H	-4.1338420	2.6058340	-0.9788050
H	0.1102490	-1.0838390	3.2106940
H	-1.2656620	-2.1950960	2.9310490
H	0.1330170	-2.2066720	1.8167720
H	-3.7761510	1.6694000	-2.4208790
C	-6.0411840	-1.5493310	1.1115390
H	-5.9135690	-1.8959430	2.1345410
H	-6.9480950	-0.9497170	1.0212890
H	-6.0824810	-2.3953310	0.4238010
C	0.4026790	2.7371190	0.3385510
C	1.7092790	2.9223800	0.7700480
C	2.6717610	1.9194150	0.5851290
C	0.9562150	0.5374020	-0.5466750
C	0.0073010	1.5537640	-0.2949930
C	4.0228840	2.0653760	1.0511970
C	4.6007240	-0.1395300	0.1905210
C	4.9433010	1.0815950	0.8657680
C	5.5348910	-1.1673510	-0.0109040
H	6.5474310	-1.0392280	0.3571180
C	5.1736650	-2.3386380	-0.6739570
C	3.8770410	-2.5108620	-1.1519020

Molecule **19**

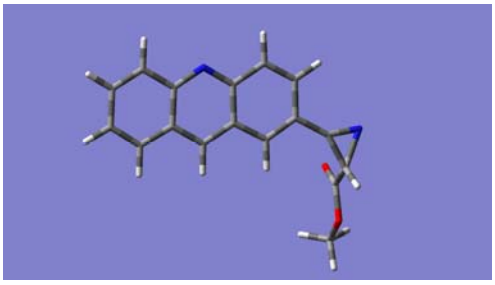

E = -915,242415, H (0K) = -914,992209,
H (373K) = -914,974451,
G (373K) = -915,038821 au.
Imaginary frequency = 0.

C	3.8044030	-0.4026360	-0.8346720
C	4.3982240	0.4911210	0.1980340
O	4.3608940	0.2941930	1.3945400
N	3.6861730	-1.8966370	-0.4561370
C	2.6371700	-1.2062860	-0.4909830
C	1.2139310	-1.2394000	-0.2873880
C	0.5716080	-2.4653290	0.0883470
C	-0.7759120	-2.5037750	0.2898030
C	-1.5763010	-1.3259780	0.1314940
C	-0.9245770	-0.0945870	-0.2479200
C	0.4818990	-0.0857260	-0.4510650
N	-2.8964650	-1.4086330	0.3411710
C	-3.6476990	-0.3008770	0.1916280
C	-3.0897870	0.9758500	-0.1870970
C	-1.7129900	1.0482400	-0.4010500
C	-5.0541720	-0.3877060	0.4143250
C	-5.8502600	0.7178340	0.2690930
C	-5.2982460	1.9772180	-0.1056380
C	-3.9539400	2.1035490	-0.3275930
O	4.9800860	1.5565660	-0.3726590
C	5.5743970	2.5260130	0.5236910
H	4.0327270	-0.1726040	-1.8694520
H	1.1748470	-3.3576070	0.2101100
H	-1.2745520	-3.4230570	0.5746240
H	0.9727510	0.8395110	-0.7330970
H	-1.2543670	1.9899230	-0.6857630
H	-5.4684320	-1.3481920	0.6991590
H	-6.9181550	0.6399330	0.4409900
H	-5.9536070	2.8339710	-0.2126050
H	-3.5206880	3.0563470	-0.6127090
H	5.9755980	3.3066910	-0.1191360
H	4.8177690	2.9366580	1.1942790
H	6.3738190	2.0640900	1.1052640

C	2.9092810	-1.5100620	-0.9698880
C	1.5684890	-1.6434950	-1.4612610
C	0.6414180	-0.6669430	-1.2665720
H	-0.3499980	-0.7923490	-1.6841030
H	1.3051860	-2.5435290	-2.0069460
H	4.2935140	2.9844580	1.5603490
H	-0.3383470	3.5072830	0.5180030
H	1.9890750	3.8421530	1.2722150
H	5.9596800	1.2035430	1.2254690
H	5.9094100	-3.1216100	-0.8207400
H	3.6028050	-3.4223260	-1.6724620
C	3.2669370	-0.3092500	-0.2890930
C	2.2961250	0.7165660	-0.0851970

TS19-20

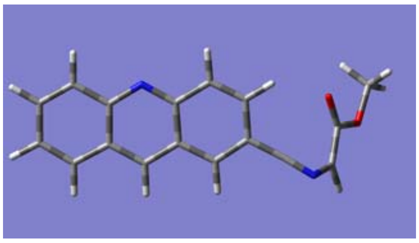

E = -915.171460, H (0K) = -914.925334,
H (373K) = -914.907245,
G (373K) = -914.972135 au.
Imaginary frequency = 1.

C	4.2492290	1.3689780	0.0846170
C	4.4980620	0.0066930	0.3030560
O	4.0989730	-0.9688510	-0.3550740
N	3.4553500	1.7993290	-1.1088410
C	2.4379800	1.1772790	-0.9671790
C	1.2050840	0.5532950	-0.8678500
C	1.0563550	-0.8195660	-1.2832500
C	-0.1567850	-1.4253120	-1.1827650
C	-1.2923650	-0.7286610	-0.6518770
C	-1.1300370	0.6443870	-0.2234680
C	0.1269800	1.2676600	-0.3460920
N	-2.4603330	-1.3694020	-0.5683370
C	-3.5281690	-0.7146750	-0.0666350
C	-3.4665890	0.6570100	0.3858950
C	-2.2449920	1.3172650	0.2955950
C	-4.7723690	-1.4006840	0.0221170
C	-5.8793680	-0.7673630	0.5259670
C	-5.8184600	0.5846680	0.9713790
C	-4.6427900	1.2802430	0.9037460
O	5.3452100	-0.1656070	1.3853980
C	5.7637820	-1.5115550	1.6460240
H	4.6006200	2.1805800	0.7001610
H	1.9274810	-1.3411970	-1.6544290
H	-0.2926640	-2.4546940	-1.4927520
H	0.2501000	2.2984910	-0.0346540
H	-2.1517280	2.3475840	0.6231950
H	-4.8162960	-2.4289710	-0.3174570
H	-6.8214500	-1.3005390	0.5889620
H	-6.7119760	1.0555290	1.3643070

Molecule **20**

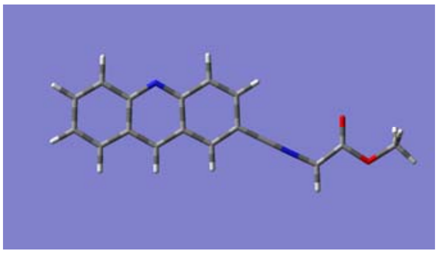

E = -915.233191, H (0K) = -914.984562,
H (373K) = -914.966127,
G (373K) = -915.032786 au.
Imaginary frequency = 0.

C	4.5503520	1.1367270	0.0008470
C	5.5627160	0.1285220	0.0004090
O	5.3881990	-1.0875960	-0.0007690
N	3.2761350	0.7542110	0.0000320
C	2.1673130	0.4024960	-0.0019950
C	0.8297810	-0.0276630	-0.0013460
C	0.5622880	-1.4428760	-0.0009140
C	-0.7219900	-1.8957650	-0.0003410
C	-1.8295700	-0.9883870	-0.0002100
C	-1.5538260	0.4288100	-0.0006880
C	-0.2096970	0.8849910	-0.0013250
N	-3.0721700	-1.4866990	0.0003460
C	-4.1138930	-0.6334260	0.0004440
C	-3.9439490	0.8002440	-0.0000290
C	-2.6434210	1.3054190	-0.0005780
C	-5.4390430	-1.1622330	0.0010160
C	-6.5226980	-0.3238610	0.0011010
C	-6.3539980	1.0915030	0.0006190
C	-5.0998400	1.6391000	0.0000710
O	6.8070980	0.6912950	0.0014200
C	7.9162630	-0.2248390	0.0009400
H	4.7562620	2.1961920	0.0018810
H	1.3944320	-2.1361710	-0.0010050
H	-0.9323200	-2.9590290	0.0000380
H	-0.0119010	1.9503720	-0.0017770
H	-2.4764170	2.3779180	-0.0009430
H	-5.5595660	-2.2397280	0.0013770
H	-7.5255120	-0.7365150	0.0015390
H	-7.2294620	1.7308830	0.0006890

H	-4.5794470	2.3098020	1.2387630
H	6.3815560	-1.4582380	2.5426900
H	4.9079620	-2.1672120	1.8255000
H	6.3539610	-1.9129380	0.8176980

H	-4.9595040	2.7148330	-0.0003000
H	8.8093440	0.3986470	0.0018600
H	7.9015770	-0.8569400	0.8917270
H	7.9022210	-0.8552120	-0.8910760

TS20-21

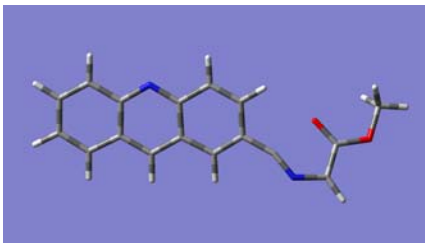

E = -915,216518, H (0K) = -914,968080,
H (373K) = -914,950719,
G (373K) = -915,013878 au.
Imaginary frequency = 1.

C	4.5780420	1.5455590	-0.3571190
C	4.8350350	0.2574710	0.1726820
O	3.9115250	-0.5889350	0.2415580
N	3.2890720	1.6216060	-0.7617150
C	2.4441000	0.7633110	-0.6699290
C	1.1585480	0.2061420	-0.5147210
C	0.9465170	-1.1968400	-0.7500490
C	-0.3034640	-1.7288100	-0.6603670
C	-1.4379120	-0.9069630	-0.3592450
C	-1.2229110	0.5024510	-0.1312890
C	0.0935320	1.0299200	-0.1916510
N	-2.6494070	-1.4724110	-0.3008780
C	-3.7143480	-0.6982370	-0.0141620
C	-3.6022900	0.7190360	0.2361430
C	-2.3336450	1.2959390	0.1693230
C	-5.0064590	-1.2989890	0.0463550
C	-6.1120020	-0.5429970	0.3366510
C	-6.0000730	0.8558090	0.5845440
C	-4.7784290	1.4708520	0.5362270
O	6.0950410	-0.0015020	0.5690430
C	6.3396720	-1.3094950	1.1279510
H	5.2841110	2.3393840	-0.5272210
H	1.8018640	-1.8155360	-0.9882170
H	-0.4707700	-2.7866560	-0.8270670
H	0.2510050	2.0846720	0.0014590
H	-2.2080200	2.3588540	0.3499400
H	-5.0847590	-2.3636340	-0.1429030
H	-7.0894810	-1.0105170	0.3801170
H	-6.8919960	1.4282440	0.8123960
H	-4.6800400	2.5349390	0.7232000
H	7.3961480	-1.3182520	1.3906350
H	5.7294790	-1.4682530	2.0195090
H	6.1287820	-2.0890630	0.3934230

TS20-22

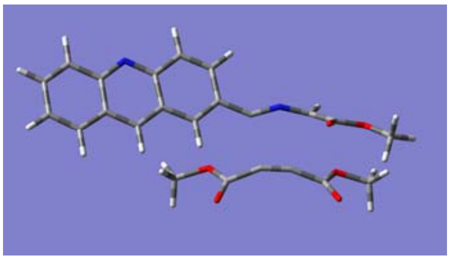

E = -1448.497276, H (0K) = -1448.133042,
H (373K) = -1448.103069,
G (373K) = -1448.197437au.
Imaginary frequency = 1.

C	3.2136820	-1.3481670	0.5095640
C	4.0057780	-1.8561390	-0.6151770
O	3.5675520	-2.2057550	-1.6943410
N	1.9025230	-1.3604840	0.4248650
C	0.8714730	-0.7553220	0.3228960
C	-0.5451810	-0.9082680	0.4773340
C	-1.0529310	-1.7638590	1.5153350
C	-2.3964100	-1.9002320	1.6995980
C	-3.3287760	-1.2070300	0.8629180
C	-2.8135090	-0.3578820	-0.1833870
C	-1.4099920	-0.2184430	-0.3436490
N	-4.6402280	-1.3696370	1.0803190
C	-5.5136160	-0.7134670	0.2927970
C	-5.0961220	0.1598010	-0.7789840
C	-3.7276190	0.3183600	-0.9954550
C	-6.9110480	-0.8840760	0.5236970
C	-7.8289170	-0.2310300	-0.2559980
C	-7.4153670	0.6308130	-1.3132630
C	-6.0848200	0.8215250	-1.5685030
C	1.9541120	1.3386120	0.0309440
C	3.1578360	1.0820770	0.0630200
C	4.5887460	1.2079040	-0.0646870
O	5.1645050	1.2629300	-1.1337980
O	5.1993650	1.2409960	1.1277910
C	6.6468460	1.3033950	1.0988780
C	0.7913370	2.1874730	-0.1235900
O	0.4078170	2.6266160	-1.1893810
O	0.1816820	2.4054120	1.0480590
C	-1.0233530	3.2113460	1.0050170
O	5.3141040	-1.8292540	-0.2978710
C	6.2391120	-2.1705660	-1.3536040
H	3.6596520	-1.2422690	1.4907070
H	-0.3493630	-2.2922930	2.1474350
H	-2.7890970	-2.5404560	2.4811800
H	-1.0347870	0.4269820	-1.1282700
H	-3.3714890	0.9661530	-1.7901500
H	-7.2198730	-1.5404580	1.3293130

H	-8.8886320	-0.3679060	-0.0707970
H	-8.1653360	1.1335710	-1.9130550
H	-5.7562930	1.4742140	-2.3702660
H	6.9540500	1.2958490	2.1424180
H	7.0487140	0.4350900	0.5752330
H	6.9787980	2.2210030	0.6108960
H	-1.3795520	3.2455940	2.0321010
H	-0.7942460	4.2157760	0.6461580
H	-1.7668920	2.7432300	0.3587350
H	7.2299520	-2.0615650	-0.9176060
H	6.1193780	-1.4903070	-2.1976910
H	6.0811830	-3.1998520	-1.6805150

Molecule **21**

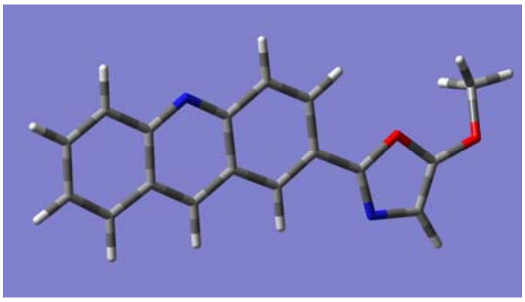

E = -915,259761, H (0K) = -915,007823,
H (373K) = -914,990810,
G (373K) = -915,053207 au.
Imaginary frequency = 0.

C	-4.4305900	-1.7621410	0.0078120
C	-4.7995880	-0.4569400	-0.0904990
O	-3.6672450	0.3075370	-0.1303670
N	-3.0445160	-1.8230090	0.0212980
C	-2.6304290	-0.5922950	-0.0525570
C	-1.2723010	-0.0700910	-0.0467280
C	-1.0434380	1.3451650	-0.0867380
C	0.2237070	1.8530360	-0.0824000
C	1.3618010	0.9895210	-0.0410460
C	1.1326180	-0.4348870	-0.0023860
C	-0.1985640	-0.9316660	-0.0054530
N	2.5899610	1.5288460	-0.0413470
C	3.6567820	0.7099470	-0.0057190
C	3.5334020	-0.7283070	0.0342780
C	2.2496680	-1.2746370	0.0353800
C	4.9658450	1.2802570	-0.0078680
C	6.0750040	0.4774610	0.0267280
C	5.9516560	-0.9424790	0.0664360
C	4.7155630	-1.5292850	0.0701550
O	-5.9805160	0.1292960	-0.2202840
C	-6.1323980	1.4396960	0.3891200
H	-5.0602220	-2.6347090	0.0627000
H	-1.8925110	2.0165280	-0.1191940
H	0.3946510	2.9230980	-0.1114050
H	-0.3561020	-2.0037080	0.0232730
H	2.1171740	-2.3516660	0.0650720
H	5.0521980	2.3606600	-0.0381170
H	7.0642450	0.9218330	0.0242020

Molecule **22**

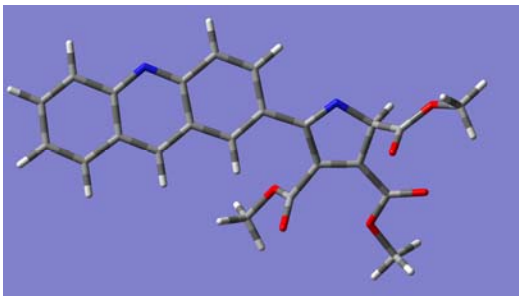

E = -1448.621608, H (0K) = -1448.251158,
H (373K) = -1448.222693,
G (373K) = -1448.312304 au.
Imaginary frequency = 0.

C	-3.2293580	-1.1928440	-0.5293190
C	-3.9670760	-1.9480270	0.5881280
O	-3.7464430	-1.7908590	1.7647020
N	-1.8684320	-1.7066430	-0.6265310
C	-1.0524050	-0.7185470	-0.4425450
C	0.4089220	-0.8864760	-0.5156640
C	0.9429060	-1.9545790	-1.3111950
C	2.2879950	-2.1599010	-1.4083110
C	3.2109970	-1.3187680	-0.7111430
C	2.6785460	-0.2511210	0.0975430
C	1.2693610	-0.0628980	0.1710370
N	4.5260080	-1.5487710	-0.8341160
C	5.3864640	-0.7526810	-0.1723940
C	4.9517080	0.3394060	0.6656850
C	3.5795970	0.5668290	0.7821410
C	6.7873620	-0.9944770	-0.3003570
C	7.6924790	-0.2055860	0.3591070
C	7.2615470	0.8723460	1.1861570
C	5.9274550	1.1380000	1.3354840
C	-1.7768230	0.5618110	-0.2057670
C	-3.0910870	0.2778530	-0.2615560
C	-4.2635120	1.1615270	-0.1207400
O	-5.3963970	0.7360910	-0.0141700
O	-3.9341970	2.4556990	-0.1291400
C	-5.0162600	3.4070390	0.0277550
C	-1.1562230	1.9016820	0.0311760
O	-1.0215230	2.3958400	1.1266990
O	-0.7451430	2.4425050	-1.1130470

H	6.8470220	-1.5530840	0.0932290
H	4.6094570	-2.6085760	0.1000500
H	-7.1824510	1.6957960	0.2657240
H	-5.8762610	1.3915990	1.4503570
H	-5.5056560	2.1725730	-0.1214160

C	-0.0854490	3.7338500	-1.0190710
O	-4.8421320	-2.8150160	0.0827120
C	-5.5817710	-3.6179540	1.0397240
H	-3.7496330	-1.3717330	-1.4757620
H	0.2538520	-2.5938600	-1.8489700
H	2.6877760	-2.9606390	-2.0200720
H	0.9051440	0.7309810	0.8111000
H	3.2131320	1.3796830	1.4014390
H	7.1086330	-1.8159220	-0.9308320
H	8.7544270	-0.3990690	0.2544810
H	8.0008340	1.4796810	1.6957980
H	5.5868070	1.9555010	1.9621240
H	-4.5438670	4.3859520	-0.0090220
H	-5.7343340	3.2972590	-0.7862070
H	-5.5121310	3.2569950	0.9877600
H	0.1746260	3.9965720	-2.0416720
H	-0.7674130	4.4711570	-0.5940370
H	0.8112450	3.6504600	-0.4037070
H	-6.2320090	-4.2514150	0.4406070
H	-6.1708150	-2.9725340	1.6925510
H	-4.8948710	-4.2241640	1.6316590

Molecule **23**

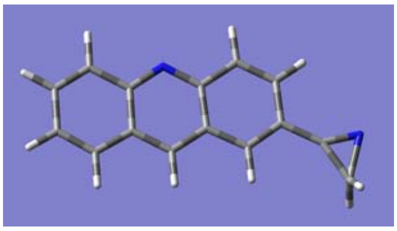

E = -687,282658, H (0K) = -687,074959,
H (373K) = -687,061649,
G (373K) = -687,114072 au.
Imaginary frequency = 0.

C	4.9833490	-1.4162230	0.0002880
N	5.2117340	0.1142230	0.0011930
C	4.0288200	-0.3243860	-0.0001950
C	2.6585790	0.1236290	-0.0004620
C	2.3626070	1.5270760	-0.0004030
C	1.0710900	1.9636760	-0.0002530
C	-0.0183490	1.0332000	-0.0001510
C	0.2822940	-0.3788610	-0.0003100
C	1.6402810	-0.8014730	-0.0004970
N	-1.2723450	1.5046890	0.0000640
C	-2.2949950	0.6284830	0.0001160
C	-2.0972560	-0.8014620	-0.0000790
C	-0.7857940	-1.2786760	-0.0002770
C	-3.6312380	1.1289820	0.0003380
C	-4.6965730	0.2674800	0.0003580
C	-4.4997170	-1.1440220	0.0001600
C	-3.2342890	-1.6648960	-0.0000520
H	5.2175100	-1.9398850	0.9234420
H	3.1839850	2.2347390	-0.0004880
H	0.8363090	3.0219550	-0.0002310
H	1.8646280	-1.8628550	-0.0006850

TS23-24

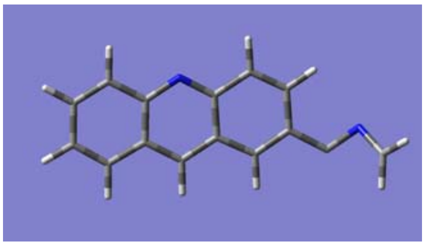

E = -687,206665, H (0K) = -687,002014,
H (373K) = -686,988706,
G (373K) = -687,041348 au.
Imaginary frequency = 1.

C	5.7022580	-0.6252040	0.5803700
N	4.9913880	0.0556990	-0.3448320
C	3.9495700	-0.7291640	-0.2309570
C	2.6199400	-0.1383430	-0.1448830
C	2.3924520	1.2839440	-0.0904570
C	1.1301000	1.7949160	-0.0363110
C	-0.0129500	0.9306640	-0.0285940
C	0.2053890	-0.4957290	-0.0775520
C	1.5347000	-0.9937940	-0.1284010
N	-1.2364760	1.4710770	0.0276810
C	-2.3073190	0.6527400	0.0368610
C	-2.1926640	-0.7855130	-0.0124310
C	-0.9113540	-1.3349830	-0.0690540
C	-3.6114090	1.2267820	0.0968880
C	-4.7237330	0.4261620	0.1065650
C	-4.6089790	-0.9931490	0.0566970
C	-3.3758730	-1.5839550	-0.0011850
H	6.2946010	-0.0452030	1.2882020
H	3.2495680	1.9463730	-0.0928160
H	0.9568410	2.8646280	0.0045440
H	1.6941340	-2.0662670	-0.1595080

H	-0.5949780	-2.3473020	-0.0003940
H	-3.7750320	2.2035430	0.0004860
H	-5.7076990	0.6594110	0.0005230
H	-5.3619210	-1.8010430	0.0001720
H	-3.0705820	-2.7373160	-0.0002100
H	5.2192190	-1.9388000	-0.9229140

H	-0.7805430	-2.4118900	-0.1061050
H	-3.6937510	2.3071020	0.1341130
H	-5.7101720	0.8743250	0.1525420
H	-5.5071030	-1.5999480	0.0651280
H	-3.2733250	-2.6631950	-0.0393470
H	5.7446020	-1.7056000	0.6279630

Molecule **24**

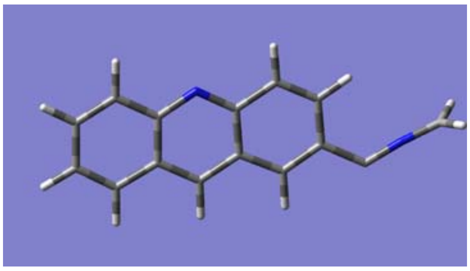

E = -687,267586, H (0K) = -687,061637,
H (373K) = -687,047866,
G (373K) = -687,101531 au.
Imaginary frequency = 0.

C	6.0867510	0.1740070	0.0000340
N	4.9433160	-0.3831970	0.0000750
C	3.8906520	-1.0180710	0.0000020
C	2.5716560	-0.3722840	-0.0000560
C	2.4221850	1.0577700	-0.0000490
C	1.1888420	1.6400610	0.0000000
C	0.0010790	0.8401470	-0.0000150
C	0.1442590	-0.5956750	-0.0000340
C	1.4483740	-1.1671270	-0.0000460
N	-1.1931360	1.4484500	0.0000160
C	-2.3063850	0.6905770	0.0000190
C	-2.2678360	-0.7522580	0.0000210
C	-1.0164600	-1.3712980	-0.0000130
C	-3.5793900	1.3357010	0.0000060
C	-4.7335100	0.5973210	-0.0000030
C	-4.6937110	-0.8272680	0.0000110
C	-3.4933080	-1.4847750	0.0000300
H	6.5737390	0.4020930	-0.9432370
H	3.3132320	1.6765590	-0.0000780
H	1.0762250	2.7185810	0.0000230
H	1.5483890	-2.2471360	-0.0000710
H	-0.9453150	-2.4545010	0.0000140
H	-3.6029750	2.4196610	-0.0000030
H	-5.6950200	1.0988390	-0.0000220
H	-5.6230540	-1.3852700	0.0000050
H	-3.4494190	-2.5687880	0.0000440
H	6.5737530	0.4022280	0.9432520

TS24-25

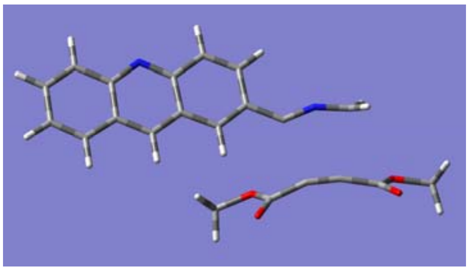

E = -1220.532982, H (0K) = -1220.212027,
H (373K) = -1220.186389,
G (373K) = -1220.269708 au.
Imaginary frequency = 1.

C	3.6834730	-2.0911750	-0.6125950
N	2.4279710	-1.7878080	-0.5381770
C	1.4917330	-1.0393980	-0.3691140
C	0.0707460	-1.1876420	-0.1687910
C	-0.4600340	-2.3798650	0.4354600
C	-1.7987430	-2.5095190	0.6598700
C	-2.7106590	-1.4693700	0.2904290
C	-2.1783350	-0.2781550	-0.3251320
C	-0.7765340	-0.1635590	-0.5294690
N	-4.0184270	-1.6370450	0.5292090
C	-4.8715850	-0.6547190	0.1821320
C	-4.4363050	0.5743740	-0.4382710
C	-3.0718450	0.7344850	-0.6819630
C	-6.2647330	-0.8305920	0.4361380
C	-7.1621320	0.1476090	0.0975220
C	-6.7308620	1.3603960	-0.5146150
C	-5.4038890	1.5686990	-0.7756380
C	2.9759980	0.8899530	-0.0946650
C	4.1069100	0.4388820	-0.2355930
C	5.5198570	0.2474330	-0.4032230
O	6.1103320	0.3850320	-1.4586930
O	6.1148150	-0.0945490	0.7537670
C	1.8986050	1.8320670	0.0941790
O	1.4712880	2.5637860	-0.7780200
O	1.4237730	1.7957010	1.3472160
C	0.3168080	2.6843230	1.6406880
H	4.1916700	-2.4122860	0.2901660
H	0.2243770	-3.1724200	0.7148790
H	-2.2040170	-3.4040770	1.1190470
H	-0.3877420	0.7396730	-0.9839360
H	-2.7036170	1.6437870	-1.1464730
H	-6.5867710	-1.7550130	0.9020830
H	-8.2184680	0.0029390	0.2957370
H	-7.4641890	2.1162520	-0.7713020
H	-5.0625530	2.4870550	-1.2414860

H	0.0688620	2.4952650	2.6828270
H	0.6177780	3.7238770	1.5027440
H	-0.5348120	2.4538860	0.9994550
H	4.1172760	-2.2926910	-1.5860230
C	7.5432990	-0.3243030	0.6930720
H	7.8336310	-0.5874270	1.7080470
H	7.7673130	-1.1443200	0.0087100
H	8.0621610	0.5801700	0.3716080

Molecule **25**

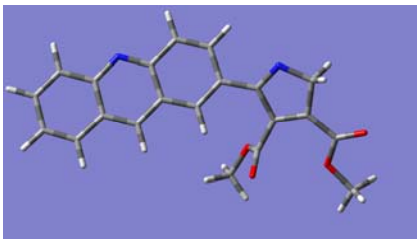

E = -1220.673899, H (0K) = -1220.346189,
H (373K) = -1220.322289,
G (373K) = -1220.401091 au.
Imaginary frequency = 0.

C	3.6338880	-2.2758700	-0.4305570
N	2.2180920	-2.5718160	-0.3221170
C	1.5843120	-1.4537360	-0.1628430
C	0.1190990	-1.3946780	0.0002260
C	-0.5580500	-2.5192770	0.5784870
C	-1.9119770	-2.5207230	0.7487500
C	-2.7026320	-1.3988800	0.3474470
C	-2.0282780	-0.2704010	-0.2436900
C	-0.6131550	-0.3028190	-0.4004250
N	-4.0301250	-1.4343720	0.5344720
C	-4.7660300	-0.3747060	0.1512120
C	-4.1850680	0.7995900	-0.4550930
C	-2.8018490	0.8218450	-0.6412360
C	-6.1794410	-0.4080050	0.3495400
C	-6.9603340	0.6520100	-0.0284690
C	-6.3850920	1.8105220	-0.6275090
C	-5.0343850	1.8825770	-0.8352350
C	2.5115360	-0.2928950	-0.1380420
C	3.7561250	-0.7920090	-0.2893190
C	5.0538130	-0.0899510	-0.3016190
O	6.0923010	-0.6149500	-0.6499890
O	4.9531800	1.1758490	0.1214770
C	2.1263690	1.1402380	0.0323330
O	2.0804310	1.9427560	-0.8720950
O	1.8020720	1.3973740	1.2994660
C	1.3549520	2.7474010	1.5913190
H	4.1925780	-2.8129010	0.3458650
H	0.0313650	-3.3722430	0.8915430
H	-2.4177110	-3.3684560	1.1969930
H	-0.1429940	0.5512700	-0.8726760
H	-2.3269950	1.6879570	-1.0917720
H	-6.6107900	-1.2920310	0.8055740
H	-8.0328430	0.6155050	0.1279520
H	-7.0277240	2.6342890	-0.9166520

DMAD

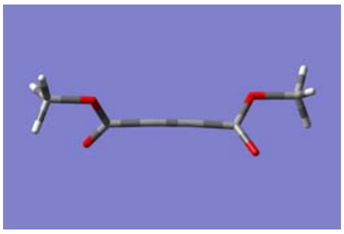

E = -533.260867, H (0K) = -533.146859,
H (373K) = -533.134909,
G (373K) = -533.185637 au.
Imaginary frequency = 0.

C	0.5992530	-0.1888750	0.0596870
C	-0.5992370	-0.1886750	-0.0589870
C	-2.0302570	-0.2509510	-0.2734080
O	-2.5414860	-1.0055350	-1.0706390
O	-2.6741490	0.6124630	0.5077140
C	-4.1236680	0.6372860	0.3836350
C	2.0304540	-0.2525050	0.2726580
O	2.5425750	-1.0126180	1.0639950
O	2.6734270	0.6164960	-0.5031050
C	4.1231760	0.6397780	-0.3816040
H	-4.4542190	1.3957180	1.0888860
H	-4.4061250	0.9073540	-0.6344020
H	-4.5366450	-0.3378190	0.6445160
H	4.4526560	1.4038710	-1.0812220
H	4.4078230	0.9013430	0.6380340
H	4.5352540	-0.3332650	-0.6514250

H	-4.5847650	2.7589070	-1.2901450	
H	1.1283170	2.7503110	2.6549470	
H	2.1477950	3.4615830	1.3658290	
H	0.4625760	2.9798190	1.0084530	
H	4.0230740	-2.6339560	-1.3916700	
C	6.1655440	1.9671030	0.1118410	
H	5.8697750	2.9488890	0.4748890	
H	6.9114660	1.5240280	0.7733280	
H	6.5611420	2.0380940	-0.9025140	

References

(1) Gaussian 16, Revision A.03, Frisch, M. J.; Trucks, G. W.; Schlegel, H. B.; Scuseria, G. E.; Robb, M. A.; Cheeseman, J. R.; Scalmani, G.; Barone, V.; Petersson, G. A.; Nakatsuji, H.; Li, X.; Caricato, M.; Marenich, A. V.; Bloino, J.; Janesko, B. G.; Gomperts, R.; Mennucci, B.; Hratchian, H. P.; Ortiz, J. V.; Izmaylov, A. F.; Sonnenberg, J. L.; Williams-Young, D.; Ding, F.; Lipparini, F.; Egidi, F.; Goings, J.; Peng, B.; Petrone, A.; Henderson, T.; Ranasinghe, D.; Zakrzewski, V. G.; Gao, J.; Rega, N.; Zheng, G.; Liang, W.; Hada, M.; Ehara, M.; Toyota, K.; Fukuda, R.; Hasegawa, J.; Ishida, M.; Nakajima, T.; Honda, Y.; Kitao, O.; Nakai, H.; Vreven, T.; Throssell, K.; Montgomery, J. A., Jr.; Peralta, J. E.; Ogliaro, F.; Bearpark, M. J.; Heyd, J. J.; Brothers, E. N.; Kudin, K. N.; Staroverov, V. N.; Keith, T. A.; Kobayashi, R.; Normand, J.; Raghavachari, K.; Rendell, A. P.; Burant, J. C.; Iyengar, S. S.; Tomasi, J.; Cossi, M.; Millam, J. M.; Klene, M.; Adamo, C.; Cammi, R.; Ochterski, J. W.; Martin, R. L.; Morokuma, K.; Farkas, O.; Foresman, J. B.; Fox, D. J. Gaussian, Inc., Wallingford CT, 2016.

(2) (a) Becke, A. D. *J. Chem. Phys*. **1993**, *98*, 5648–5652. (b) Becke, A. D. *Phys. Rev. A* **1988**, *38*, 3098–3100. (c) Lee, C.; Yang, W.; Parr, R. G. *Phys. Rev. B* **1988**, *37*, 785–789.

(3) (a) Grimme, S.; Antony, J.; Ehrlich, S.; Krieg, H. *J. Chem. Phys*. **2010**, *132*, 1054104. (b) Grimme, S.; Ehrlich, S.; Goerigk, L. *J. Comput. Chem.* **2011**, *32*, 1456–1465.

(4) Marenich, A. V.; Cramer, C. J.; Truhlar, D. G. *J. Phys. Chem. B*, **2009**, *11* , 6378-6396.
